# Chemo‐ and Regioselective Multiple C(sp^2^)−H Insertions of Malonate Metal Carbenes for Late‐Stage Functionalizations of Azahelicenes

**DOI:** 10.1002/anie.202210798

**Published:** 2022-09-01

**Authors:** Yana Nikolova, Bibiana Fabri, Pau Moneva Lorente, Alejandro Guarnieri‐Ibáñez, Adiran de Aguirre, Yoshiki Soda, Gennaro Pescitelli, Francesco Zinna, Céline Besnard, Laure Guénée, Dimitri Moreau, Lorenzo Di Bari, Eric Bakker, Amalia I. Poblador‐Bahamonde, Jérôme Lacour

**Affiliations:** ^1^ Department of Organic Chemistry University of Geneva Quai Ernest Ansermet 30 1211 Geneva 4 Switzerland; ^2^ Department of Inorganic and Analytical Chemistry University of Geneva Quai Ernest Ansermet 30 1211 Geneva 4 Switzerland; ^3^ Dipartimento di Chimica e Chimica Industriale University of Pisa Via G. Moruzzi 13 56124 Pisa Italy; ^4^ Laboratory of Crystallography University of Geneva Quai Ernest Ansermet 24 1211 Geneva 4 Switzerland; ^5^ Department of Biochemistry University of Geneva Quai Ernest Ansermet 24 1211 Geneva 4 Switzerland

**Keywords:** C−H Insertion, Carbenes, Helicenes, Late-Stage Functionalization, Photophysical Properties

## Abstract

Chiral quinacridines react up to four times, step‐by‐step, with α‐diazomalonates under Ru^II^ and Rh^II^ catalysis. By selecting the catalyst, [CpRu(CH_3_CN)_3_][PF_6_] (Cp=cyclopentadienyl) or Rh_2_(oct)_4_, chemo and regioselective insertions of derived metal carbenes are achieved in favor of mono‐ or bis‐functionalized malonate derivatives, respectively, (r.r.>49 : 1, up to 77 % yield, 12 examples). This multi‐introduction of malonate groups is particularly useful to tune optical and chemical properties such as absorption, emission or Brønsted acidity but also cellular bioimaging. Density‐functional theory further elucidates the origin of the carbene insertion selectivity and also showcases the importance of conformations in the optical response.

## Graphical abstract

Chiral quinacridines react chemo‐ and regioselectively with α‐diazomalonates under Ru^II^/Rh^II^ catalysis, up to four times, achieving a stepwise late‐stage multi‐functionalization of the [4]helicene scaffold. Absorption, emission or Brønsted acidity but also cellular bioimaging are readily tuned. Density‐functional theory elucidates the carbene insertion selectivity and clarifies the importance of conformations in the optical response.

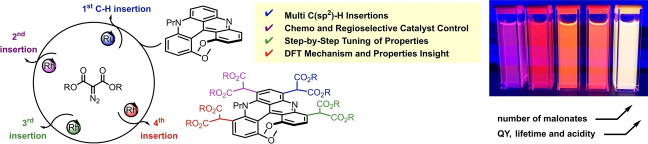

## Introduction

Helicenes, chiral *ortho*‐condensed polyaromatic derivatives, are molecules of general scientific interest in view of their applications in analytics, biochemistry, catalysis and synthesis, physics and surface sciences.[^1^, ^2^] To manipulate these helical moieties, and their absorption, emission, electronic circular dichroism (ECD) and circularly polarized luminescence (CPL) properties in particular, chemists often rely on the introduction of substituents at their periphery.^[3]^ In that regard, late‐stage functionalizations (LSF) of core structures are particularly attractive.^[4]^ With (hetero)helicenes, chemo and regioselectivity, however, remain a challenge as these molecules often contain a large number of “equivalent” C−H aromatic sites. LSF rely then essentially on directing groups or on the use of skeletons pre‐activated at certain positions.^[5]^ This approach, while efficient, tends to favor specific reactivities at single site only.

**Figure 1:**
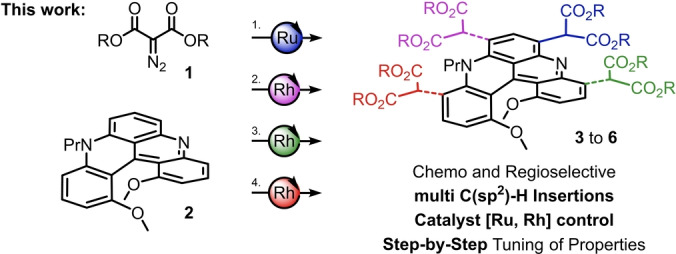
Step‐by‐step synthesis of mono, bis, tris and tetra malonate functionalized [4]helicenes **3** to **6** by catalyst‐controlled C(sp^2^)−H insertions of electrophilic metal carbenes (Ru, Rh) onto diaza [4]helicene **2**.

Herein, in a new development, it is shown that metal‐catalyzed decompositions of α‐diazomalonates **1** can be used, step‐by‐step, to multi‐functionalize chiral diaza [4]helicene **2** on the outer rim (Figure 1). By selecting the catalyst, [CpRu(CH_3_CN)_3_][PF_6_] (Cp=cyclopentadienyl) or Rh_2_(oct)_4_, chemo‐ and regioselective insertions of derived metal carbenes can be achieved in favor of mono‐ or bis‐functionalized malonate derivatives, respectively, (up to 77 % isolated yield, 12 examples). Furthermore, the higher reactivity of rhodium carbenes can be used for the controlled formation of products of tris‐ and tetra‐carbene insertions. This multi‐introduction of malonate functional group (FG), rare in traditional LSF approaches, is particularly useful to induce a gradual and measured evolution of important properties of [4]helicene **2** such as absorption (Δ*λ*=−44 nm), emission (quantum yield 5→22 % and lifetime 3.0→8.4 ns, CH_3_CN) and Brønsted acidity (p*K*_a_ **2**•H^+^ to **6**•H^+^, 6.63→−0.42 and lower).

## Results and Discussion

**Figure 2:**
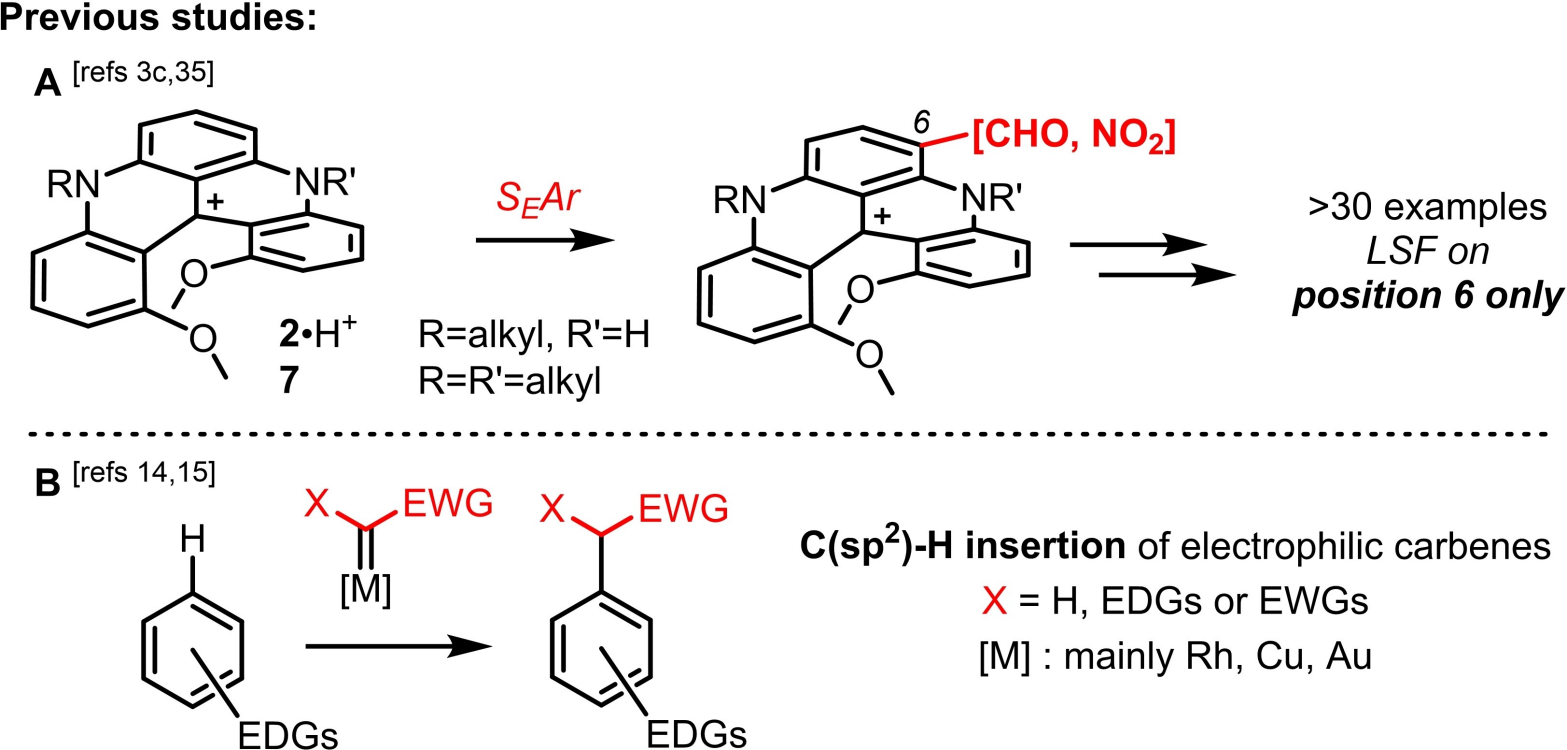
Section **A**: Single‐site S_*E*_Ar LSF of cationic helicenes **2•H^+^** and **7**. Section **B**: Direct C(sp^2^)−H insertions of electrophilic metal carbenes into electron‐rich aromatics. EDGs: electron‐donating groups and EWGs: electron‐withdrawing groups.

Absorption, fluorescence, ECD and CPL are important properties attached to helicenes and, for purely organic derivatives, these features appear usually in the blue range of the visible spectrum.^[6]^ Targeting the red spectral region (620–750 nm) is essential for applications in microscopy and chemical biology but it remains a global challenge.^[7]^ Cationic [4], [5] and [6]helicenes, which beneficiate from the extended delocalization provided by the triarylcarbenium framework, are welcome exceptions.^[8]^ DMQA (DiMethoxyQuinAcridinium) **7**, and related quinacridine derivative **2**, are classical representatives of this class of heteroaromatics.[^9^, ^10^] These moieties are remarkably nucleophilic despite their cationic and heteroaromatic characters. In fact, they react readily under Vilsmeier–Haack (VH) or nitration conditions to afford the corresponding mono‐carbaldehyde or nitro derivatives.^[3c]^ However, both CHO and NO_2_ substituents are strong electron‐withdrawing groups (EWGs) that deactivate the aromatic nuclei and hence limit dramatically additional S_E_Ar reactivity (Figure 2, section **A**). For that matter, controlled bis, tris or tetra VH or nitration reactions of **2**•H^+^ and **7** have never been achieved and possible benefits from multiple FG substitution never accessed.

**Scheme 1:**
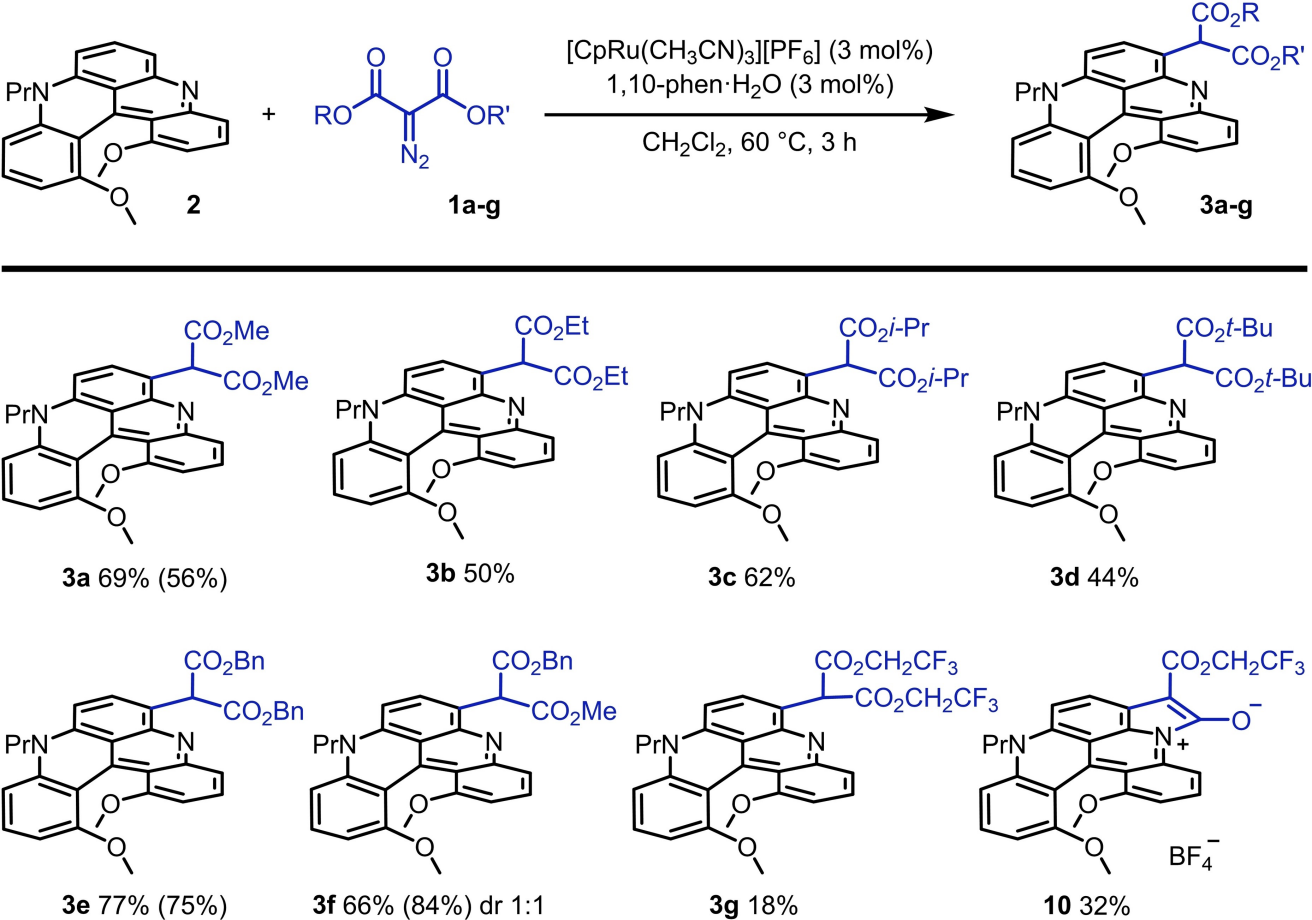
Reaction conditions: **2** (0.1 mmol), **1 a**–**g** (2 equiv), [CpRu(CH_3_CN)_3_][PF_6_] (3 mol %), 1,10‐phen⋅H_2_O (3 mol %), CH_2_Cl_2_, 60 °C, 3 h. Isolated yields. In parentheses, reactions at 1 mmol scale.

**Scheme 2:**
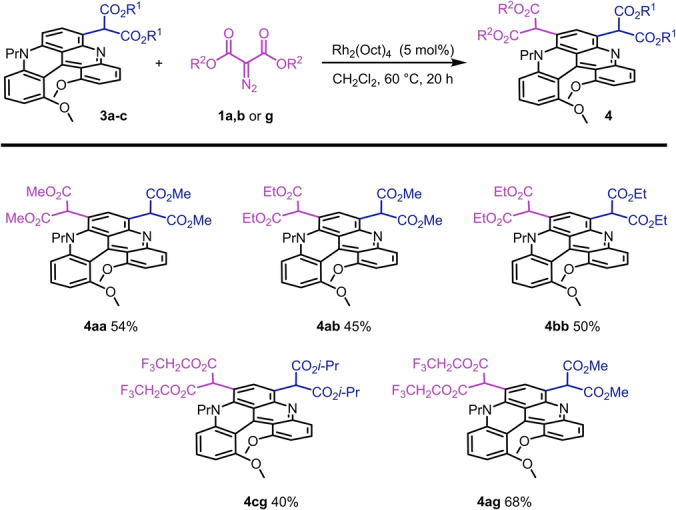
Reaction conditions: **3 a**–**c** (0.1 mmol), **1 a**, **1 b** or **1 g** (2 equiv), Rh_2_(oct)_4_ (5 mol %), CH_2_Cl_2_, 60 °C, 22 h.

To achieve polysubstitutions, the reactivity of electrophilic metal carbenes was considered.^[11]^ Such intermediates undergo a remarkably large range of synthetically useful transformations including C−H insertions.^[12]^ Usually, C(sp^3^)−H reactivity is preferred over C(sp^2^)−H insertions, which occur nevertheless with electron‐rich aromatic structures (Figure 2, section **B**).^[13]^ With such substrates, different mechanistic pathways can be considered, including electrophilic aromatic substitutions via Wheland‐type intermediates.^[14]^ In these examples, anilines, anisoles and indoles are predominant starting materials and most reactions involve donor‐acceptor diazo derivatives as carbene precursors.^[15]^ With this background in hand, quinacridine **2** and DMQA **7** were tested with α‐diazo arylacetates **8** (Scheme S1), β‐ketoesters or phosphonate **9** (Scheme S2) or malonate reagents **1** (Table 1, Schemes 1 and 2) in the presence of metal salts or complexes.

**Table 1:** Functionalization of quinacridine **2** by metal‐catalyzed decomposition of dimethyl 2‐diazomalonate **1 a**. Optimization studies for chemo‐ and regioselective mono C(sp^2^)−H insertion.

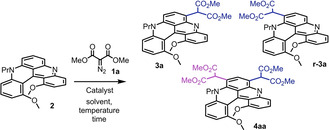						
				Yield [%]^[a]^		
Entry	Catalyst (mol %)	*T* [°C]	*t* [h]	**3 a**	**r‐3 a**	**4 aa**
1	CuI (10)^[b]^	100	16	0	0	0
2	CuTC (10)^[b]^	80	22	0	0	0
3	Rh_2_(OAc)_4_ (5)	60	16	12	25	7
4	Rh_2_(oct)_4_ (5)	60	16	18	18	29
5	Rh_2_(TFA)_4_ (5)	60	16	30	19	5
6	Rh_2_(esp)_2_ (5)	60	16	29	16	4
7	Rh_2_(piv)_4_ (5)	60	16	48	4	5
8	[CpRu(CH_3_CN)_3_][PF_6_] (3)	60	24	26	0	0
9	[CpRu(CH_3_CN)_3_][PF_6_] (3)^[c]^	60	16	45^[d]^	0	0
10	[CpRu(CH_3_CN)_3_][PF_6_] (3)^[c]^	60	3	69^[d]^	0	0
11	[CpRu(CH_3_CN)_3_][BAr_F_] (3)^[c]^	60	3	18^[d]^	0	0
12	No catalyst, no ligand	60	16	0	0	0

[a] NMR yields by ^1^H NMR analysis of crude reaction mixtures using 1,3,5‐trimethoxybenzene as internal standard. [b] 1,2‐Dichloroethane as solvent. [c] With additional 1,10‐phenanthroline monohydrate (3 mol %) as ligand. [d] Isolated yield.

While the classical quinacridinium **7** was inactive under all conditions, **2** was found to react principally with aryl diazoacetates and α‐diazo malonates.^[16]^ With reagents **8**, each insertion of the corresponding metal carbene onto quinacridine **2** created a new stereogenic center and complex (diastereomeric) crude mixtures were obtained. To simplify, optimization studies were thus performed with dimethyl diazomalonate **1 a** exclusively. Such acceptor‐acceptor diazo reagents **1**, while being amongst the most stable diazo derivatives,^[17]^ still decompose readily under metal catalysis. Initial experiments with CuI and CuTc were unproductive (entries 1 and 2). With Rh_2_(OAc)_4_, but also Rh_2_(oct)_4_, Rh_2_(TFA)_4_ and Rh_2_(esp)_2_,^[18]^ products of mono carbene addition, **3 a** and regioisomer **r‐3 a**, but also product of double addition **4 aa** were observed in the crude mixtures; all structures being confirmed by X‐ray diffraction analysis (Figure S21).[^19^, ^20^] In entries 3 to 7 (Table 1), different proportions among products **3 a**, **r‐3 a** and **4 aa** were obtained which are difficult to rationalize based on catalyst structure. Nevertheless, Rh_2_(oct)_4_ appears to be particularly active, favoring the formation of bis adduct **4 aa** preferentially (entry 4). Bulky Rh_2_(piv)_4_ afforded the most selective reaction (entry 7) of the dirhodium series. Isolation of **3 a** from **r‐3 a** and **4 aa** by chromatography remained somewhat cumbersome and optimization studies were further pursued. [CpRu(CH_3_CN)_3_][PF_6_] was tested as decomposition catalyst (entry 8), but also its 1 : 1 combination with 1,10‐phenanthroline as ligand (entry 9). Both reactions afforded **3 a** as a single product in 26 % and 45 % yields, respectively. Using a reaction time of 3 h instead of 16 h, full conversion of **2** and a higher isolated yield of **3 a** (69 %) were achieved (entry 10).^[21]^ Finally, salt [CpRu(CH_3_CN)_3_][BAr_F_] was tested (entry 11)^[22]^ and a lower yield was observed (18 %).^[23]^ Of note, in term of reactivity, the formation of pyridinium ylide products was never observed despite the presence of the Lewis basic nitrogen atom.^[24]^ Conditions detailed in entry 10, affording perfect regio‐ and chemoselectivity for the mono C−H insertion, were selected for the remainder of the study.

As a rule, acyclic diazomalonate reagents afforded effective C(sp^2^)−H insertion and formation of products **3** in low to good yields (18–77 %). For this first malonate addition step, reactivity is relatively insensitive to steric hindrance as methyl, ethyl, but also *iso*‐propyl and *tert*‐butyl side‐chains are compatible.^[25]^ The highest yield was achieved with dibenzyl diazomalonate **1 e** affording **3 e** in 77 % isolated yield. Similarly, unsymmetrical diazo **1 f** afforded corresponding [4]helicene **3 f** in 66 % yield (1 : 1 diastereomeric ratio). Interestingly, in these two reactions involving benzyl ester diazo derivatives, products of intramolecular Buchner additions could not be detected.^[26]^ Scaling up reactions to 1 mmol scale was readily achieved; compounds **3 a** (Me), **3 e** (Bn) and **3 f** (BnMe) being isolated with 56 %, 75 % and 84 % yields, respectively. Finally, with bis(2,2,2‐trifluoroethyl) diazomalonate **1 g**, C−H insertion also occurred effectively but isolation of corresponding product **3 g** was achieved with low yield only (18 %) as most of the product reacted in situ to form betaine **10** (32 %). Formation of **10** probably involves a ketene intermediate followed by an intramolecular trapping of the electrophilic species by the N‐atom. Mechanistic details provided by DFT calculations are depicted in Figure S22.

To afford the targeted multi‐LSF, metal carbenes derived from dirhodium catalysts were looked for. In fact, preliminary results had established that Rh_2_(oct)_4_ was particularly effective to generate product **4** directly from unfunctionalized **2** (Table 1, entry 4). This metal complex was thus used for the development of the double functionalization protocol. In practice, treatment of mono‐adducts **3** with 2‐diazomalonates **1** (2 equiv) in the presence of Rh_2_(oct)_4_ (5 mol %) afforded derivatives **4** in moderate to good yields (Scheme 2). While the transformation proceeded relatively well to generate helicenes **4 aa** (54 %), **4 bb** (50 %) and mixed **4 ab** (45 % by addition of **1 b** onto **3 a**), it was less proficient with hindered reagents or substrates. In fact, using **3 a** and diazomalonates with bulkier chains like **1 c** and **1 e**, reactions proceeded poorly.

Similar results were obtained with substrates **3 c** or **3 e** carrying hindered malonate groups (R^2^=Bn, *i*‐Pr; Scheme S3). An increased electrophilicity of the metal carbene was necessary in these latter cases and it was achieved using bis(2,2,2‐trifluoroethyl) diazomalonate reagent **1 g**. In fact, reaction of hindered **3 c** proceeded now smoothly in favor of **4 cg** (40 %) and the highest yield was provided in the reaction of **3 a** and the consecutive formation of **4 ag** (68 %). Practically, it is possible to combine both sets of Ru‐ and Rh‐catalyzed conditions and realize, in one‐pot, the tandem C−H insertions of diazomalonate **1 a** onto helicene **2**. This direct formation affords an isolated yield of 60 % of **4 aa** (54 % on 1 mmol scale, Figure S1), quite higher than the combined yield of the two isolated steps (37 % overall).

**Scheme 3:**
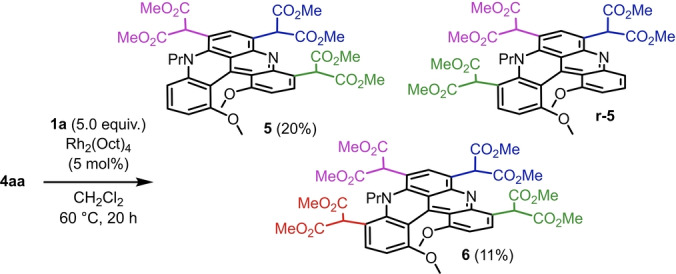
Reaction conditions: a) **4 aa** (0.1 mmol), **1 a** (5 equiv), Rh_2_(oct)_4_ (5 mol %), CH_2_Cl_2_, 60 °C, 20 h.

In addition, thanks to the higher reactivity of rhodium malonate carbenes, triple and quadruple C(sp^2^)−H insertions could be achieved (Scheme 3). Treatment of **4 aa** (1.0 equiv) with **1 a** (5.0 equiv) under Rh_2_(oct)_4_ catalysis (5 mol %) afforded products **5** and **6** in 20 % and 11 % isolated yields, respectively, after isolation and purification by chromatography. While the regioselectivity was excellent for the formation of tetra‐functionalized derivative **6** (regioisomeric ratio, r.r.>20 : 1), a mixture of tri‐substituted **5** and **r‐5** was observed (r.r. 1:0.6). Only **5** and **6** were isolated in analytically pure forms. Despite many experiments, conditions to improve isolated yields and selectivity between **5**, **r‐5** and **6** could not be found. Each addition of a malonate moiety introduces a strong EWG that deactivates effectively subsequent electrophilic aromatic substitutions and thus reduces the probability for further C−H insertion.^[27]^

**Figure 3:**
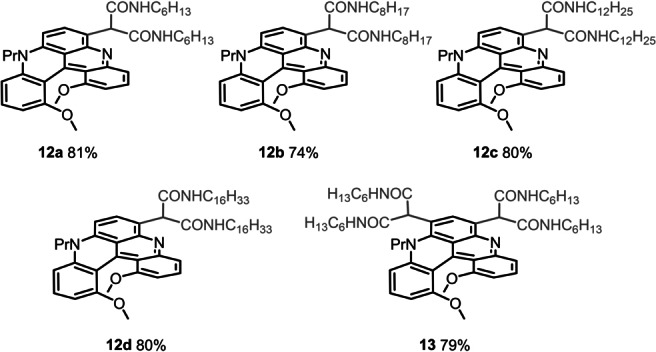
Mono **12** and bis**13** malonate amides carrying linear alkyl side chains, from C_6_ to C_16_.

Finally, with derivatives **3** and **4** as major adducts, further late‐stage functionalizations were considered, and the formation of polyamide derivatives **12** and **13** in particular (Figure 3).^[28]^ Syntheses were easily performed by heating precursors **3 a** and **4 aa** at 90 °C with primary amines as solvent (RNH_2_, R=C_6_H_13_, C_8_H_17_, C_12_H_25_, C_16_H_33_). Each malonate group was transformed into the corresponding bis(amides) without evidence of decarboxylation.^[29]^ Good overall yields were afforded for compounds **12 a**–**12 d** (74–81 %, Figure 3) and **13** (79 %).

**Figure 4:**
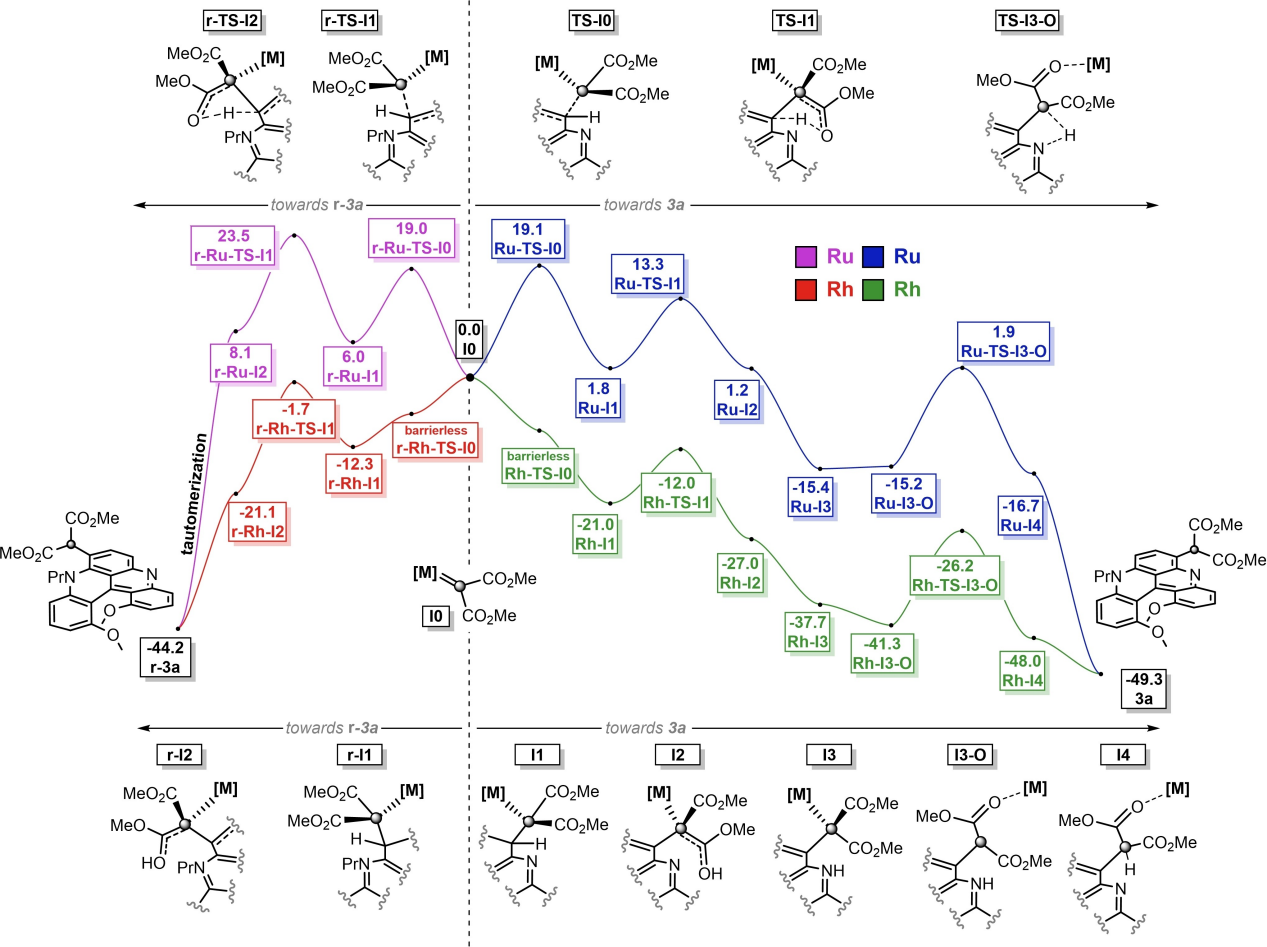
Computed Gibbs energy profiles for the formation of **3 a** (right) and **r‐3 a** (left) under CpRu and dirhodium catalysis. All energies in kcal mol^−1^.

DFT calculations were then conducted to rationalize the distinct reactivity of Ru or Rh catalysts. To ensure a full mechanistic picture, the formation of **3 a**, **r‐3 a** and **4 aa** products was calculated with both catalytic conditions. The results for the formation of **r‐3 a** and **3 a** are summarized in Figure 4, left and right panels, respectively, using malonate carbene complexes **I0** as starting points.^[30]^ The same mechanism applies for both Ru and Rh mediated reactions although with important energetic differences. In the case of **3 a**, four key series of steps are observed: (1) a metal‐carbene addition to the C(sp^2^)−H bond, (2) two subsequent proton transfer steps (CH→OH and then OH→NH), (3) a migration of the metal from the reactive carbon to an O‐coordination, and (4) a final NH→CH proton transfer step to deliver target **3 a** after catalyst regeneration. Precise information on all the elementary steps is detailed in the Supporting Information (pages S39 and S40). With both Ru and Rh sets of data in hand, several considerations can be extracted. According to the calculations, and as observed experimentally, the CpRu‐catalyzed reaction generates **3 a** exclusively. In fact, this is the result of a Curtin–Hammett situation that can be traced to the ΔΔ*G* ^≠^ of more than 10.0 kcal mol^−1^ between **r‐TS‐I1** and **TS‐I1**. Intermediates **r‐I1** and **I1** are in equilibrium via **r‐TS‐I0** and **TS‐I0** of similar energies. This reversible **r‐I1**⇌**I1** process is then shifted towards the formation of **3 a** by the considerably faster kinetics of **I1** (**TS‐I1**) towards **I2**. For the dirhodium system, a different energetic situation is observed. The barrierless nature of the first transition states, **r‐TS‐I0** and **TS‐I0**, requires the consideration of the subsequent steps. Then, in this case, the low ΔΔ*G* ^≠^ (<2.0 kcal mol^−1^) between **r‐TS‐I1** and **TS‐I1** indicates that both pathways are productive, in accordance with the experimental results that yield a mixture of **3 a** and **r‐3 a**.

Concerning the formation of bis‐malonate product **4 aa**, DFT calculations for the initial steps are also provided in the Supporting Information and justify the formation of the adduct under Rh‐catalysis only (Figure S23 and related information).

Then, with **3 a**, **4 aa**, **5** and **6** in hand, the stereoelectronic influence of the malonate FGs and their corresponding effects on the physico‐chemical properties was investigated. First, the electronic character of **3 a** to **6** was assessed by cyclic voltammetry and the results are compared to parent compound **2** (Figure S7 and Table S1). Each additional electron‐withdrawing contribution of the malonate moieties renders reduction more facile. In oxidation mode, a similar trend was observed since non‐reversible signatures were found at +0.13, +0.25 and +0.47 V for **2**, **3 a** and **4 aa**, respectively. With **5** and **6**, two reversible waves were found at +0.48 and +0.72 V vs Fc^+^/Fc, respectively. This could indicate that, with three or four malonate groups at the periphery that shield the central core, radical cationic species are less inclined to react with the external medium. Finally, electronic gap values (Table S1) increase from **2** to **6**; a trend that will be compared with the optical data.

**Figure 5:**
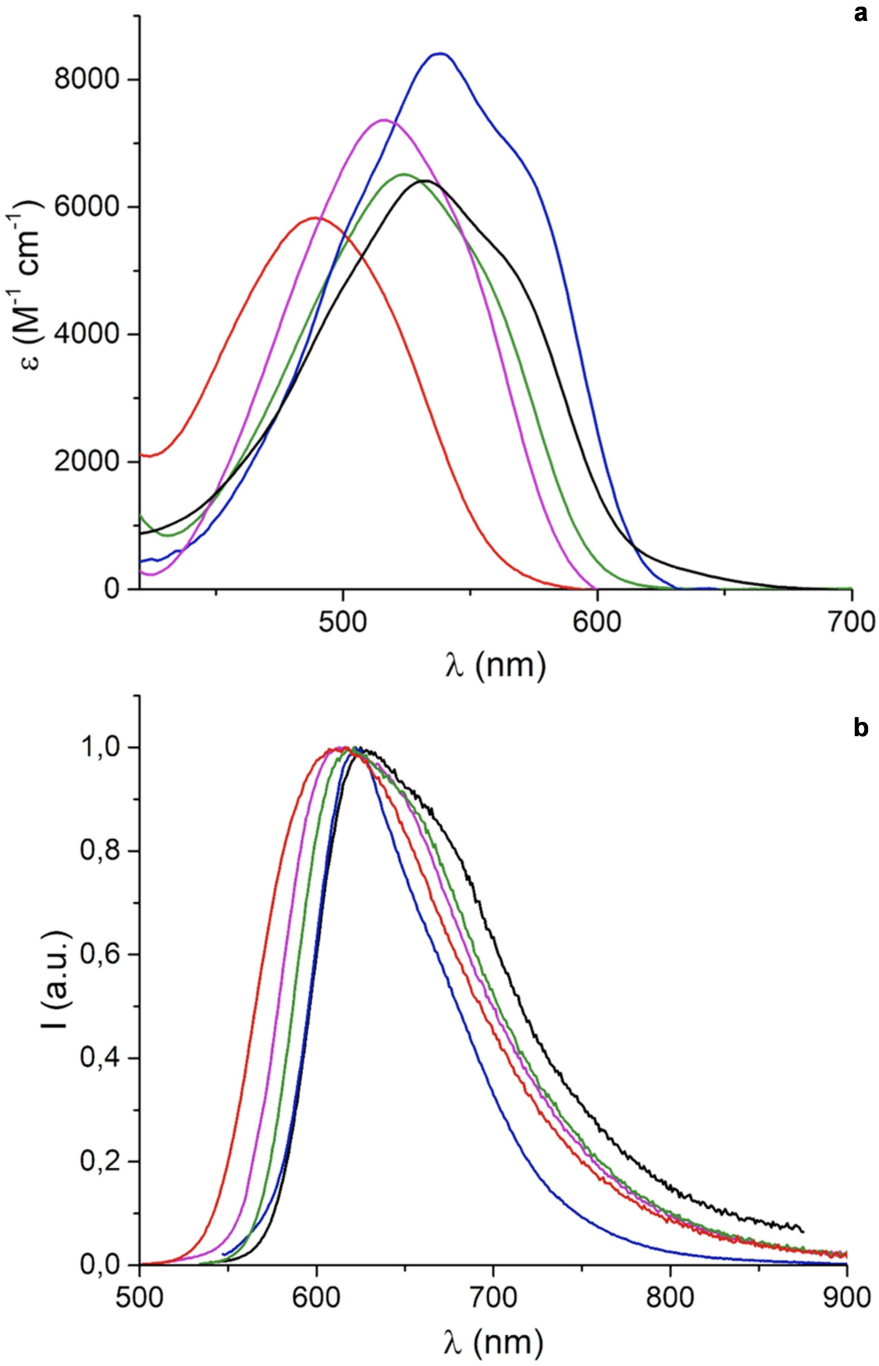
a) Absorption and b) normalized emission spectra of compound **2** (black), **3 a** (blue), **4 aa** (magenta), **5** (green) and **6** (red) in air‐equilibrated acetonitrile solution (C ca. 10^−5^ M) at 293 K.

Absorption and fluorescence properties were then studied on the neutral (Figure 5) and protonated (Figures S8–S18) helicenes in acetonitrile (Table 2). The core structure **2** presents a relatively broad absorption maximum at 533 nm and a weak emission at 625 nm with fluorescence quantum yield of 5 %. Upon the introduction of the malonate groups, a general hypsochromic shift in the lowest energy absorption band was observed corresponding to an overall blue‐shift of 44 nm from **2** to **6**. However, this global trend needs to be examined in detail as each introduction of a malonate moiety provokes either a red or a blue‐shift with amplitude depending on the degree of substitution. In fact, while compound **3 a** is slightly red‐shifted compared to compound **2** (*λ*_max_ 533→537 nm), compound **4 aa** is blue‐shifted with respect to **3 a** (*λ*_max_ 537→516 nm). The same fluctuation is noticed for compounds **5** and **6** as the third added malonate moiety brings about a red‐shift compared to **4 aa** (*λ*_max_ 516→524 nm) and the fourth substitution a rather strong blue‐shift (*λ*_max_ 524→489 nm). Analogous variations are observed for the emission maxima as shown in Table 2. Consequently, the optical energy gap does not increase with each addition of a malonate moiety; values of 2.10, 2.09, 2.17, 2.13 and 2.26 eV are measured for **2**, **3 a**, **4 aa**, **5** and **6**, respectively. This contrasts with the trend for the electronic energy gap (see above and Table S1).

**Table 2:** Photophysical properties of neutral and protonated malonate‐functionalized [4]helicenes in acetonitrile.

Compound	*λ*_max_ [nm]	*ϵ* [M^−1^ cm^−1^]	*λ*_em_ [nm]	Stokes shift [cm^−1^]	Φ_F_ [%]	τ [ns]^[b]^
**2**	533	6410	625	2762	5^[a]^	3.0^[c]^
**3 a**	537	8400	631	2774	5^[a]^	3.0^[c]^
**4 aa**	516	7400	613	3067	17^[a]^	7.5
**5**	524	6510	621	2981	18^[a]^	7.7
**6**	489	5730	616	4216	22^[a]^	8.4
**2**•**H^+^**	622	8560	674	1240	1^[d]^	1.4
**3 a**•**H^+^**	616	9660	666	1219	2^[d]^	1.9
**4 aa**•**H^+^**	612	8280	673	1481	3^[d]^	2.6
**5**•**H^+^**	614	8400	677	1516	3^[d]^	2.4
**6**•**H^+^**	599	5880	684	2074	2^[d]^	2.1

[a] Reference 4‐(dicyanomethylene)‐2‐methyl‐6‐(p‐dimethylaminostyryl)‐4*H*‐pyran (Φ=43,5 % in ethanol), estimated error=±10 %. [b] Excitation at 470 nm. [c] A component with a time constant around 10 ns and relative amplitude<2 has been omitted. [d] Reference oxazine 1 (Φ=11 % in ethanol), estimated error=±10 %.

**Figure 6:**
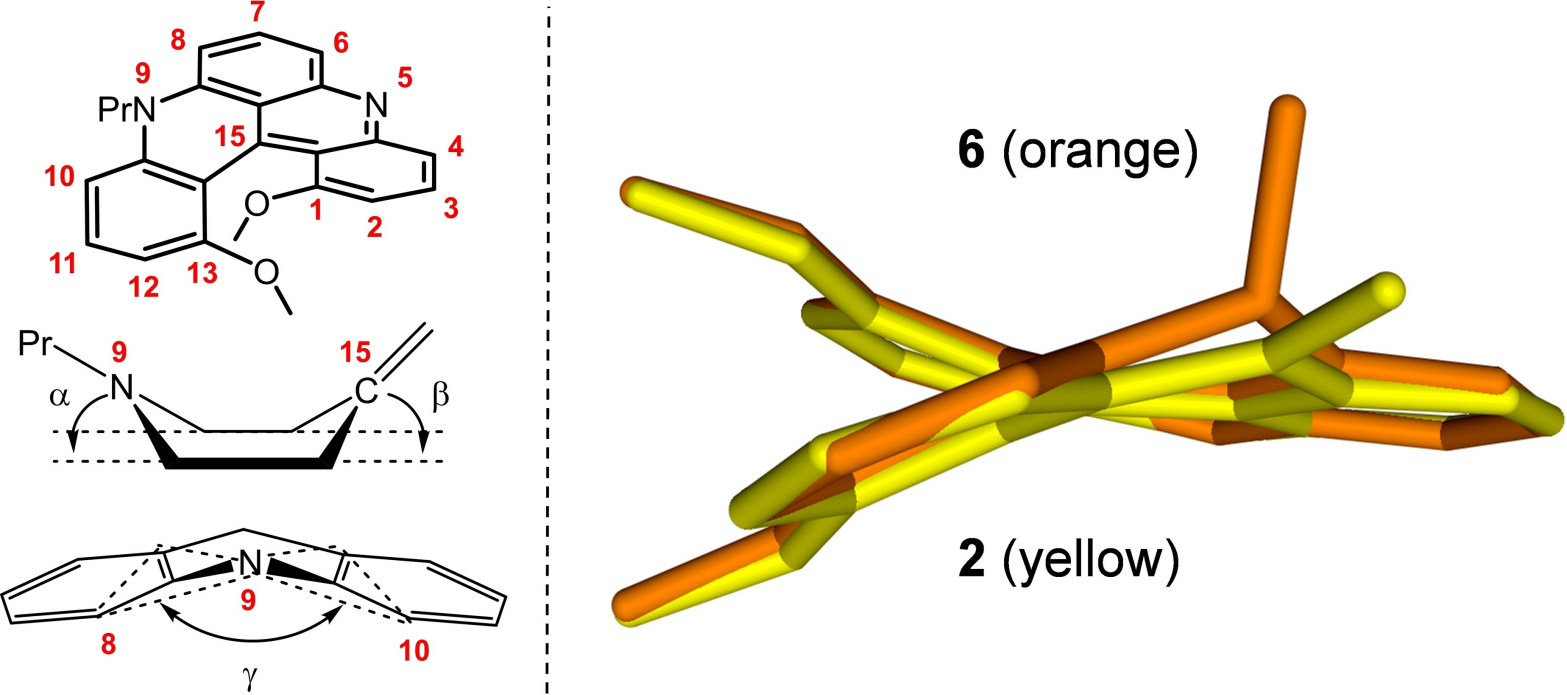
(Left) Representative skeletal deformations upon the introduction of malonate groups at positions adjacent to the *N*‐propyl side chain (atom N9). *α* and *β* [°] are the dihedral angles of the envelope at N9 and C15, respectively. *γ* [°] is the bent angle corresponding to the folding of the central ring along the N9⋅⋅⋅C15 line. (Right) Overlay between DFT‐optimized geometries of **2** and **6**. Only the C, N and O atoms of the helicene cores are showed, for clarity. A more complete picture with the malonate chains of **6** is shown in Figure S25.

To elucidate this peculiar behavior, time‐dependent density‐functional theory (TDDFT) calculations were performed and they are in excellent agreement with the trend of experimental optical data (Figure 6, Table S5). Of importance, it should be noticed that small red‐shifts correspond to the introduction of malonate groups next to the unsubstituted nitrogen atom, at positions C6 (**3 a**) and C4 (**5**), while larger blue‐shifts occur when the EWGs are positioned adjacent to the *N*‐propyl moiety, on C8 (**4 aa**) and C10 (**6**). For **3 a** and **5**, the decrease of the optical energy gap upon the functionalization of the less‐hindered helicene side corresponds to a classical influence of electron‐withdrawing groups on polyaromatic substrates.^[31]^

However, when malonate FGs are introduced next to the propyl‐substituted N atom (compounds **4 aa** and **6**), strong interactions occur that (i) reduce the conformation freedom of the *n*‐propyl and malonate groups (see below) and (ii) induce a puckering of the six‐membered aza cycle. In fact, as shown recently for alkynyl derivatives,^[32]^ *n*‐propyl chains are impacted strongly by neighboring substituents, in consequence, these alkyl residues adopt *gauche* conformations that fold above the helical core. Experimental characterization of this effect is observed in the solid‐state structures of **r‐3 a** and **4 aa** (Figure S21). For the malonate group(s), the equivalent of an allylic A(1,3)‐strain interaction tends to position the C−H bonds of the bifunctional FG almost periplanar to the adjacent aromatic skeleton.^[33]^ Further structural deformations of the quinacridinium ring occur (Figure 6) as the strain results in “out‐of‐plane” displacements for atoms N9 and C15 (*α* 28.2–30.7°, *β* 16.2–19.2°) and in a bending of the aromatic skeleton (*γ* angle, 152.7–147.5°). Results of the computational data are indicated in Table S5. For instance, for compound **6**, the six‐membered aza cycle assumes a boat‐like conformation. This out‐of‐plane distortion for the N‐atom decreases the overall conjugation and hence the hypsochromic shifts observed upon the malonate introduction in positions C8 and C10.^[34]^ In brief, in term of optical properties, a fine interplay between electronic and steric effects is demonstrated upon the addition of the malonate moieties.

Finally, an enhancement of the fluorescence quantum yields is observed as values increase up to 22 % from **2** to **6** (Table 2). The introduction of malonate groups next to the hindered nitrogen atom seems, again, to have a stronger influence in relation probably with the increase of rigidity for the related structures (**4 aa** and **6**). Fluorescence lifetimes follow a similar trend up to 8.4 ns for **6** (Figure S19). These values are rather high for organic emitters in the red domain of the visible spectrum and hence useful for time‐gated fluorescent measurements.^[35]^ The protonated conjugates of the series, from **2**•**H^+^** to **6**•**H^+^**, present strongly red‐shifted absorption and emission spectra and their fluorescence quantum yields are significantly lower (Table 2 and Figures S8, S10, S13, S15, S17).

**Figure 7:**
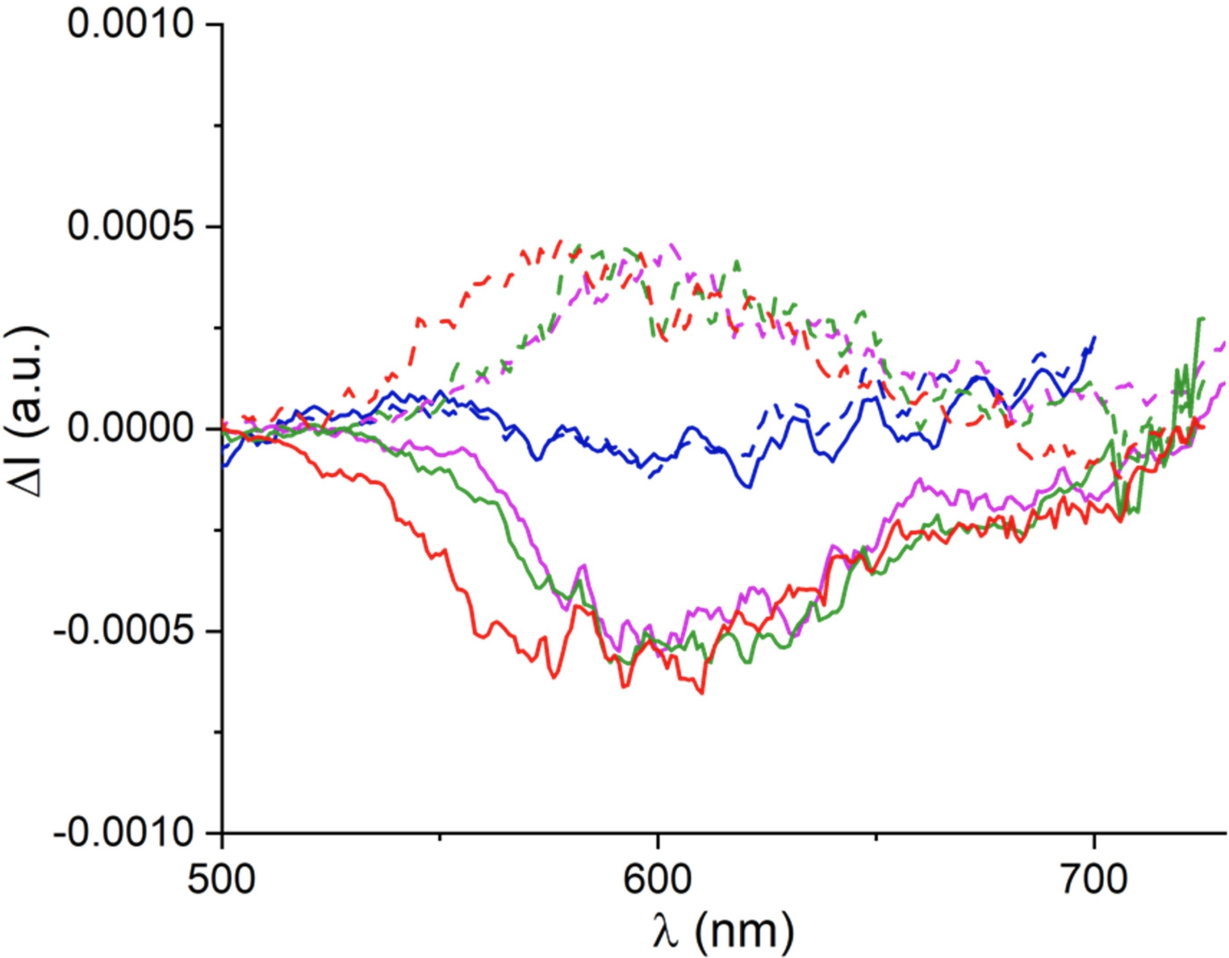
CPL spectra (*M* full and *P* dashed lines) of compounds **3 a** (blue), **4 aa** (magenta), **5** (green) and **6** (red) in air‐equilibrated acetonitrile solution (C ca. 10^−5^ M) at 293 K.

At this stage, the left‐ and right‐handed enantiomers of compounds **2** to **6** of *M* and *P* configurations, respectively, were separated using chiral stationary phase (CSP) HPLC (Figures S2–S6) and their chiroptical properties studied in acetonitrile. The UV/Vis electronic circular dichroism (ECD) spectra are reported in the Supporting Information (Figures S9, S11, S14, S16, S18) and the circularly polarized luminescence (CPL) in Figure 7. All compounds of the series present pronounced ECD bands in the UV domain around 270 nm. The sign of the Cotton effects is fully conserved for the levo‐ and dextrorotatory enantiomers indicative of identical absolute configurations for the (−) and (+)‐enantiomers in the series. The absolute configuration assignment was then achieved by ECD comparison with the unsubstituted compound **2** ^[10a]^ matching, for instance, dextrorotatory characters with *P* configuration for compounds **2** to **6**. For the lowest energy transition, only compounds **4 aa**, **5** and **6** display a Cotton effect (|Δ*ϵ*|≈5 M^−1^ cm^−1^), while no signal is observable for the parent compound **2** and the mono‐substituted **3 a**. In all likelihood, the occurrence of Cotton effects at low energy is related to the conformational changes occurring with the introduction of malonate groups at the immediate proximity of the alkyl‐substituted N9 atom in **4 aa**, **5** and **6** (see above). Then, unsurprisingly, no CPL signal could be measured for compounds **2** and **3 a**.^[36]^ While, at the emission maxima, the *g*_lum_ values are +0.0005/−0.0005, +0.0004/−0.0005 and +0.0004/−0.0005 for the *P*/*M* enantiomers of **4 aa**, **5** and **6**, respectively. These values correspond to a CPL brightness (*B*_CPL_) of around 0.3 M^−1^ cm^−1^.^[37]^ Although the measured CPL signals of the polysubstituted compounds **4 aa**, **5** and **6** remain in the 10^−4^ to 10^−3^ range, in line with values typical for most helicene derivatives,^[37]^ this constitutes a strong improvement in comparison to core substrate **2** and mono‐substituted derivatives.[^3c^, ^38^]

Care was also taken to study the Brønsted acidity of the derivatives, i.e. the proton transfer ability of the corresponding salts **3 a**•H^+^ to **6**•H^+^, in comparison with that of core structure **2**•H^+^. Due to the water immiscibility of the materials, a recent method able to determine p*K*_a_ values of hydrophobic chromogenic pH sensitive probes was selected (Figure S26).^[39]^ As described by E. Bakker and co‐workers, p*K*_a_ values can be obtained by measuring the degree of dye deprotonation induced by ion exchange between H^+^ and Na^+^, followed by measuring the relative lipophilicities of H^+^ and Na^+^ by using a solvatochromic dye as a reference ion, embedded in emulsified nanosensors containing the dye of interest and an ion exchanger. While the p*K*_a_ of the unfunctionalized helicene **2**•H^+^ was determined to be 6.63±0.08, the functionalized derivatives from **3 a**•H^+^ to **5**•H^+^ exhibited more acidic p*K*_a_ values, 4.23±0.01, 1.89±0.01, −0.42±0.01, respectively. The p*K*_a_ value of **6**•H^+^ could not be obtained as the acidity of the compound was too high to be fully protonated. Therefore, a stepwise increase in acidity, or a proportional decrease in p*K*_a_ values (2.4 per substitutional group) has been observed. Clearly, upon the progressive addition of electron‐withdrawing malonate FGs from mono to tetra‐substitution, a rather strong electron‐withdrawing effect occurs for each malonate insertion.

**Figure 8:**
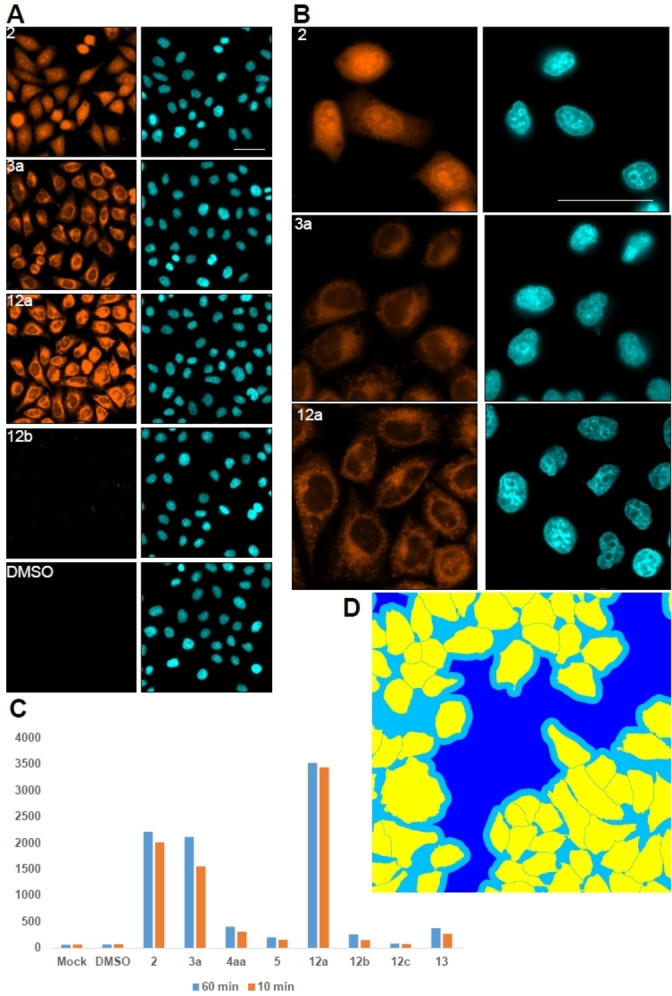
Microscopy images of Hela‐MZ cells treated for 60 min with indicated compounds. Blue (Hoechst staining) Orange (compounds **2**, **3 a**, **12 a** or **12 b**). A) 20X water immersion widefield image used for signal quantification. B) 60X water immersion images for the compounds of interest. C) Quantification of average intensity Txred signal for all tested compounds for 10 or 60 min. D) Segmentation mask example generated on A, Yellow (Cell), Light blue (image), Dark blue (Background). Images for signal intensity quantification. Scale bar 50 μm.

Finally, cellular imaging studies were considered as the optical properties of compounds **2** to **6**, but also **12** and **13**, display absorption and fluorescence from the visible red to near‐infrared domains, which are close to the transparency window of biological media. Previously, similar derivatives have been used as effective probes for different cellular sub‐components (late endosomes, mitochondria, chloroplasts).[^35c^, ^40^] Live and fixed cell (PFA) imaging procedures (see Supporting Information) were thus performed by treatment of Hela‐MZ cells with DMSO solutions of **2**, esters **3 a**, **4 aa**, **5** and amides **12 a**, **12 b**, **12 c** and **13** (Figures 8 and S28).^[41]^ Two different concentrations were used (10 μM and 1 μM) and cells were exposed for either 10 or 60 min, without detectable signs of cytotoxicity. Signal localization between live and fixed conditions showed little differences demonstrating an effective fast entry of the compounds inside the cell, most likely by passive diffusion and some endocytosis. Overall, compound **2** shows integration into the endoplasmic reticulum (ER) and nucleus; the derivative crossing the nuclei envelope to give signals similar to DNA binding derivatives like Hoechst. Of all the malonate esters, only fluorophore **3 a** showed an intense labeling of the ER and of vesicles, most probably endosomes; compounds **4 aa** and **5** displaying the same preference but with a (very) weak intensity (Figure 8, **C**). Finally, in the amide series, a very strong discrimination occurs among the derivatives. Only **12 a**, containing two *n*‐hexyl amide side chains, enters the cells and integrates in the ER, more than **3 a** (Figure 8, **C**). Compounds **12 b** and **12 c**, with *n*‐octyl and *n*‐dodecyl residues, show instead little or no labeling. A similar result is observed for **13** demonstrating that cell permeation is sensitive not only to the length of the amide alkyl chains but also to the number/position of them.

## Conclusion

Using pH‐sensitive diaza [4]helicene **2** as an original heterohelicene scaffold, it was possible to select four out of nine C(sp^2^)−H bonds for step‐by‐step chemo‐ and regioselective insertions of malonate metal carbenes. By a careful choice of catalyst, [CpRu(CH_3_CN)_3_][PF_6_] vs Rh_2_(oct)_4_, mono‐ or bis‐functionalized malonate helicenes could be prepared in good yields (up to 77 % yield, 12 examples) and excellent regioisomeric ratios (>49 : 1). Mechanism of formation and origin of selectivity were elucidated based on DFT calculations. Tris‐ and tetra‐malonate insertions could be further accomplished thanks to the higher reactivity of rhodium carbenes; these additional functional groups being beneficial for chiroptical properties. In fact, the multi‐introduction of malonate groups is particularly useful to tune properties of the [4]helicene core. The electron‐withdrawing groups blue‐shift the absorptions (Δ*λ* −44 nm) and entail improved emission properties (quantum yield 5→22 % and lifetime 3.0→8.4 ns in CH_3_CN). Brønsted acidity of the conjugated acids are strongly modified (p*K*_a_ **2**•H^+^ to **6**•H^+^, 6.63→−0.42 and lower), which could help develop selective intracellular probes.^[40b]^ Cellular imaging studies also evidenced strong discrimination among the various malonate esters or amides in the labelling of Hela‐MZ cells. All in all, LSF with strongly electrophilic yet tunable metal carbenes was pursued and provided ease of synthesis and improved properties of the derivatives. Applications of stepwise multi‐functionalization strategies ought to be considered in any field looking for quick synthetic access and manipulations of properties.

**Supporting Information** contains experimental conditions, full characterizations of all new compounds (PDF); CSP‐HPLC traces, p*K*_a_ determination, UV/Vis, ECD, CPL and fluorescence spectra; computational details. In addition, the dataset for this article can be found at the following DOI: 10.26037/yareta:py3o7ztvrnfdtcatka6yfrz27e. It will be preserved for 10 years.

## Conflict of interest

The authors declare no conflict of interest.

1

## Supporting information

As a service to our authors and readers, this journal provides supporting information supplied by the authors. Such materials are peer reviewed and may be re‐organized for online delivery, but are not copy‐edited or typeset. Technical support issues arising from supporting information (other than missing files) should be addressed to the authors.

Supporting Information

Supporting Information

## Data Availability

The data that support the findings of this study are openly available in yareta.unige.ch at https://doi.org/10.26037/yareta:py3o7ztvrnfdtcatka6yfrz27e, reference number 4008.
