# Supplementary material for: Chemo‐ and Regioselective Multiple C(sp2)−H Insertions of Malonate Metal Carbenes for Late‐Stage Functionalizations of Azahelicenes
Source: Angew Chem Int Ed Engl. 2022 Sep 1;61(41):e202210798. doi: 10.1002/anie.202210798 (PMC9825994; doi:10.1002/anie.202210798)
Supplement: Supplementary file 2 — Supporting Information [file ANIE-61-0-s002.pdf]

Angewandte Chemie
Eine Zeitschrift der Gesellschaft Deutscher Chemiker

Supporting Information

Chemo- and Regioselective Multiple C(sp^2)–H Insertions of Malonate Metal Carbenes for Late-Stage Functionalizations of Azahelicenes

*Y. Nikolova, B. Fabri, P. Moneva Lorente, A. Guarnieri-Ibáñez, A. de Aguirre, Y. Soda, G. Pescitelli, F. Zinna, C. Besnard, L. Guénée, D. Moreau, L. Di Bari, E. Bakker, A. I. Poblador-Bahamonde, J. Lacour**

Contents

General remarks and analysis conditions S4

Synthesis and characterization of new compounds S7

General procedures for synthesis of α-diazo malonates S7

Catalysts - structures S7

General procedure for synthesis of mono functionalized quinacridines **3** S7

Procedure for synthesis and characterization of **r-3a** S12

General procedure for synthesis of bis functionalized quinacridines **4** S13

Synthetic procedure for one-pot tandem preparation of quinacridine **4aa** S15

Synthetic procedure for preparation of quinacridines **5** and **6** S16

General procedure for synthesis of mono **12** and bis amides **13** S17

Chiral Stationary Phase (CSP) HPLC resolution S20

Resolution and specific rotation of compound **2** S20

Resolution and specific rotation of compound **3a** S21

Resolution and specific rotation of compound **4aa** S22

Resolution and specific rotation of compound **5** S23

Resolution and specific rotation of compound **6** S24

Electrochemical properties S25

General Figure S25

Electrochemical data of compounds **2**, **3a**, **4aa**, **5**, **6** S25

Optical and chiroptical properties S26

Absorption, emission and ECD spectra of compounds **2** and **2•H$^+$** S26

Absorption, emission and ECD spectra of compounds **3a** and **3a•H$^+$** S27

Absorption and emission spectra of compound **r-3a** S28

Absorption, emission and ECD spectra of compounds **4aa** and **4aa•H$^+$** S28

Absorption, emission and ECD spectra of compounds **5** and **5•H$^+$** S29

Absorption, emission and ECD spectra of compounds **6** and **6•H$^+$** S30

Lifetime measurements S31

X-ray structure determination S33

Comparison S33

Compound **3a** (CCDC 2161999) S33

Comments on the model: S34

Asymmetric unit with displacement ellipsoids at 50 percent probability. S34

Compound **r-3a** (CCDC 2162000) S35

Comments on the model: S35
Asymmetric unit with displacement ellipsoids at 50 percent probability. S36
Compound **4aa** (CCDC 2162001)........ S37
Asymmetric unit with displacement ellipsoids at 50 percent probability. S38
Computational results........ S39
Formation of betaine-type product **10** S39
Details concerning the formation of mono-malonate products **3a** and **r-3a** S39
Formation of bis-malonate product **4aa** S40
TDDFT calculation of optical properties........ S41
pK_a Determination - nanoparticle-based method [26]........ S43
General Figure........ S43
Particle preparation S43
Buffer preparation S43
Absorbance measurements S43
Cell imaging S45
^{1}H, ^{13}C, ^{19}F NMR spectra, IR spectra and HRMS reports of new compounds........ S46
XYZ Coordinates S111
References........ S156

General remarks and analysis conditions

Reagents

Unless otherwise stated, reagents were purchased from commercial sources and used without further purification. All reactions involving air sensitive compounds were carried out under N_2 *via* an inert gas/vacuum double manifold line and standard Schlenk techniques using dry solvents. Reactions involving oxygen sensitive reagents were performed using degassed solvents.

Chromatography

Analytical thin layer chromatography (TLC) and **retardation factors** (R_f) were performed with Silica gel 60 F_{254} aluminium plates purchased from Merck. **Flash column chromatograph** was performed with Silica SiliaFlash P60, 40-63 µm (230-400 mesh) and with Combi*Flash*® *R*f 200 on SiO_2 4 g, 12 g and 24 g cartridges. Enantiopure materials were obtained by chiral stationary phase **HPLC** resolution on an Agilent 1260 Infinity II apparatus (quaternary pump, auto sampler, column thermostat and diode array detector) using a semi-preparative CHIRALPAK® IG column (250 x 10 mm, 5 µm), CHIRALPAK® IH column (250 x 10 mm, 5 µm) and HPLC grade solvents.

Nuclear Magnetic Resonance

NMR spectra were recorded on a Bruker Avance III 500 MHz, Bruker Avance III HD-*NanoBay* 400 MHz and Bruker Avance III HD-*NanoBay* 300 MHz spectrometers at room temperature. **^{1}H NMR** chemical shifts are given in ppm relative to Me_4Si with solvent resonances used as internal standards (CD_2Cl_2 δ = 5.32 ppm). Data are reported as follows: chemical shift (ppm) on the *δ* scale, multiplicity, coupling constant (Hz) and integration. **^{13}C NMR** chemicals shifts are given in ppm relative to Me_4Si with solvent resonances used as internal standards (CD_2Cl_2 δ = 53.84 ppm). **^{19}F NMR** chemicals shifts are given in ppm.

Infrared Spectroscopy

IR spectra were recorded with a Perkin-Elmer 100 FT-IR spectrometer using a diamond ATR Golden Gate sampling and are reported in wavenumbers (cm^{-1}).

Mass Spectrometry

LRMS spectra were obtained in methanol solutions on an API 150EX (AB/MDS Sciex) spectrometer in positive polarity. **HRMS** spectra were obtained in methanol solutions on a Waters Xevo G2 Tof (TOF) spectrometer in positive polarity by the Department of Mass Spectroscopy at the University of Geneva.

Electrochemistry

Cyclic voltammetry (CV) data were recorded using a CH instrument potentiostat. All the experiments were conducted under a nitrogen atmosphere in a one-compartment, three-electrode electrochemical cell. All measurements were performed in anhydrous acetonitrile with tetra-n-butylammonium hexafluorophosphate ([TBA][PF_6]) as supporting electrolytes (10^{-1} M). All electrodes were purchased from BAS Inc. The platinum (Ø = 3 mm) working electrode was polished with 0.05 µm alumina paste before each recording. An Ag/$AgNO_3$ (10^{-2} M + [TBA][PF_6] 10^{-1} M) electrode was used as a pseudo-reference during the measurements. All potential values were then corrected using ferrocene (Fc) as reference and all half-wave and peak potentials are reported *vs* Fc^+/Fc.

(Chir)Optical properties

Optical rotations (OR) were measured on a Perkin Elmer 241 polarimeter at room temperature using a Hg lamp (365 nm). All compounds under study in this work absorb strongly at wavelenght used to measure the optical rotation (365 nm), therefore the reported OR values might be affected by big errors. **UV-Vis-NIR absorption spectra** were recorded on a JASCO V-650 spectrophotometer at room temperature. Measurements were performed in air-equilibrated analytical grade acetonitrile at concentrations *ca*. 10^{-5} M. **Electronic Circular dichroism (ECD)** spectra were recorded on a JASCO J-815 spectrophotometer at 20 °C. Measurement were performed in air-equilibrated analytical grade acetonitrile at concentrations *ca*. 10^{-5} M, with parameters as follows: scan speed – 200 nm/min, slit width – 1 nm, integration time – 1 sec, accumulations – 10. All spectra were baseline corrected by subtraction of the solvent spectrum. **Steady-state fluorescence** spectra were

measured using a FluoroMax+ spectrofluorometer from Horiba Scientific. All fluorescence spectra were corrected for the wavelength-dependent sensitivity of the detection. Fluorescence quantum yields Φ were determined by comparison with a standard of known quantum yield (Table 2) using the following equation:

$$\Phi = \Phi_r \frac{I\, A_r\, n^2}{I_r\, A\, n_r^2}$$

where *A* is the absorbance at the excitation wavelength (λ), *n* the refractive index and *I* the integrated emission intensity; "r" stands for reference. Diluted solutions with absorption lower than 0.1 were employed. Excitations of reference and sample compounds were performed at the same wavelength. **Fluorescence lifetimes** on the nanosecond timescale were measured by a time-correlated single photon counting (TCSPC) setup. Excitation was performed at 470 nm using ~60 ps pulse at 10 MHz or 20 MHz produced by a laser diode (PicoQuant, LDH-P-C-470). The fluorescence decay was followed at wavelenght 610 nm and 690 nm using band-pass filters for the neutral and protonated derivatives, respectively. The full width at half-maximum (fwhm) of the instrument response function (IRF) was around 200 ps. The fluorescence time profiles were analysed with the deconvolution of the experimental IRF and an exponential function. Diluted solutions with absorption lower than 0.1 were employed. **Circularly polarized luminescence (CPL)** spectra were recorded using a home-built spectrofluoropolarimeter[1] under 365 nm irradiation from a commercial LED-source, using a 90° geometry between the excitation and detection direction. All the spectra were recorded in air-equilibrated analytical grade acetonitrile at concentrations *ca*. 10^{-5} M, using the following parameters: scan-speed – 2 nm/sec, integration time – 4 sec, photomultiplier tube driving voltage – 500 V, accumulations – 8. All (chir)optical measurements were performed in 1 cm optical quartz cells.

Computational details

All calculations were carried out with Gaussian09 package (D01 version)[2] and Spartan'20 (1.1.4 version, Wavefunction, Irvine, CA, USA, 2022).The B3LYP functional,[3] together with D3 empirical dispersion correction[4] was used as level of theory. Two different basis sets were used for the calculations. In set (A) we used 6-31G* basis set for light atoms[5] and the LANL2DZ and its corresponding pseudopotential for the Ru and Rh atoms.[6] This set was used for the structure optimization of those species shown in Figure 4 and Figure S23 (mechanisms to form products **r-3a**, **3a** and **4aa**). Potential energies were further refined with a larger basis set 6-311++G**[7] for light atoms and LANL2TZ(f)[8] for the Ru and Rh atoms. In set (B) we used 6-31+G** basis set for all atoms (only light atoms were used in these calculations). This set was used for the calculations of the thermodynamic betaine-type product (compound **10**) formation. This set was necessary because we were not able to find some important intermediates in the reaction path with a smaller basis set. All geometry optimizations were carried out without symmetry constrains and solvent contributions were included via SMD model (dichloromethane, Ɛ = 8.93).[9] All optimized geometries were identified as minima or transition state trough frequency calculations. All reported energies are Gibbs free energies at 298 K and 1 atm, in kcal·mol^{-1}.

For the estimation of S0-S1 transition energies, the structures of compounds **2**, **3a**, **4aa**, **5** and **6** were built in Spartan'20 and their conformational ensemble was explored by conformational searches using Merck Molecular Force Field (MMFF) by varying all rotatable bonds and inverting the N(Pr) atom. The search generated a large number of conformers for compounds **4aa**, **5** and **6**, which were screened through a series consecutive steps similar to the procedure employed by Hehre et al. for NMR calculations of flexible compounds.[10] The steps consisted of: (1) single-point energy calculations at HF/3-21G level; (2) single-point energy calculations at B3LYP-D3/6-31G* level; (3) geometry optimizations at B3LYP-D3/6-31G* level; (4) single-point energy calculations at B3LYP-D3/6-31+G** level; (5) geometry optimizations at B3LYP-D3/6-31+G** level; (6) single-point energy calculations at B3LYP-D3/6-31+G** level including SMD solvent model for acetonitrile (Ɛ = 35.688); (7) final geometry optimizations at B3LYP-D3/6-31+G** level including SMD solvent model for acetonitrile (Ɛ = 35.688). After each step, all conformers within an energy window of 5 kcal/mol were retained (10 kcal/mol for the HF step). TD-DFT calculations were run on a representative set of 2-4 low-energy minima for each compound. The discussed results refer to the lowest-energy structure; variations up to 2-3 nm for the transition energies were found for the other investigated structures. TD-DFT calculations were run with B3LYP and CAM-B3LYP [11] functional, the def2-TZVP basis set, [12] and IEF-PCM solvent model for acetonitrile.[13]

p*K*a Determination - Nanoparticle-based method

Absorbance measurements were performed by TECAN Infinite M Nano$^+$ microplate reader (Tecan, Zürich, Switzerland) with 96 well plates sourced from Eppendorf (Hamburg, Germany). 827 pH lab (Metrohm, Herisau, Switzerland) was used to measure pH in buffer preparation. Reagents dioctyl sebacate (DOS), Pluronic® F-127 (F127), Sodium tetrakis[3,5-

bis(trifluoromethyl)phenyl]borate (NaTFPB), tetrahydrofuran (THF) of Selectophore^{TM} grade were purchased from MilliporeSigma (Burlington, MA, USA).

Crystallography

All data were collected on an Agilent Supernova using Cu Kα1 radiation. Refinements were carried out in the SHELXL[14] program, within the olex2[15] graphical software. Details for the refinement for each structure can be found below with, for each structure, a representation of the cation and of the content of the asymmetric units shown as displacement ellipsoids drawn at 50 percent probability. For disordered structures, comments on the modelling of the disorder are included.

Cell imaging

HeLa-MZ cells, a line of HeLa cells selected to be amiable to imaging, were provided by Prof. Lucas Pelkmans (University of Zurich). HeLa cells are not on the list of commonly misidentified cell lines maintained by the International Cell Line Authentication Committee.

The HeLa-MZ cells of use were authenticated by Microsynth (Balgach, Switzerland), which revealed 100% identity to the DNA profile of the cell line HeLa (ATCC: CCL-2) and 100% identity over all 15 autosomal STRs to the Microsynth's reference DNA profile of HeLa.[16]

HeLa-MZ cells were seeded in a µ-Plate 96 Well Black (IBIDI cat 89626) at concentration of 1.2 x 10^4 cell/well with FluoroBrite™ DMEM (cat. A18967-01 Thermo-Fisher). The day after cells were treated with 1 µM or 10 µM compound solutions at different time point (120-60-30-10 min), a DMSO and mock control were always present in the experiment.

After live cell wash with an automated plate washer (BioTek EL406), cells were fixed in 3% paraformaldehyde (PFA) for 15 min and then nuclei stained by Hoechst (1:2000; 10mg/ml stock). Cells were imaged by ImageXpress® Micro Confocal microscope (Molecular Devices LLC; Sunnyvale, CA) with 20X Nikon water immersion objective in widefield mode using DAPI filter (ex. 359 em. 461) for nuclei stain and TexasRed filter (ex. 595 em. 613) for compound imaging. Images were quantified using MetaXpress Custom Module editor software: cell-masks were generated, and then applied on the fluorescent images to extract relevant measurements (more details in cited reference,[17] image analysis cell count reveal that more than 5000 cells have been analyzed for each condition).

Synthesis and characterization of new compounds

General procedures for synthesis of α-diazo malonates

Important note: *Diazo compounds are high energy materials and should be handled with caution. Although α-diazo malonates are stable at room temperature, it is advisable to carry out reactions behind a blast shield.*

All diazo derivatives, used in the current study, were prepared according to reported procedures.[18]

Catalysts - structures

**CuTC**
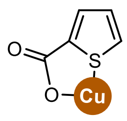

**$Rh_2(OAc)_4$**	**$Rh_2(oct)_4$**	**$Rh_2(TFA)_4$**	**$Rh_2(esp)_2$**	**$Rh_2(piv)_4$**
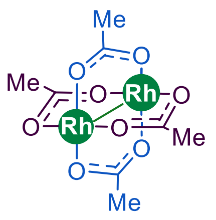	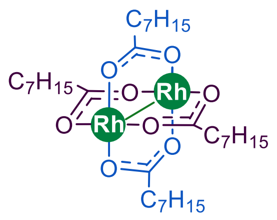	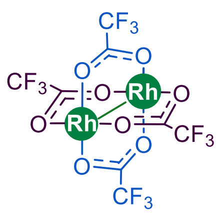	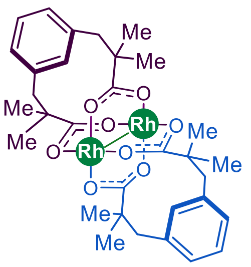	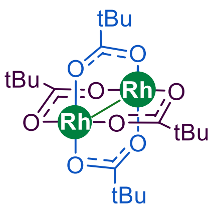

**$[CpRu(CH_3CN)_3][PF_6]$**	**$[CpRu(CH_3CN)_3][BAr_F]$**
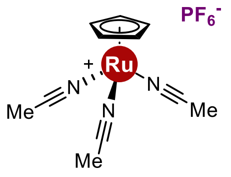	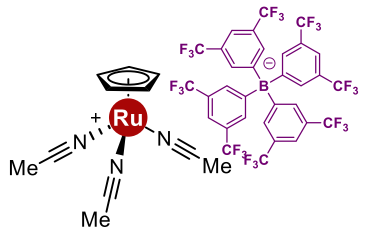

General procedure for synthesis of mono functionalized quinacridines 3

$[CpRu(CH_3CN)_3][PF_6]$ (3 mol%) and 1,10-phenanthroline monohydrate (3 mol%) were dissolved in dry CH_2Cl_2 (0.5 M) and the resulting mixture was allowed to stir for 15 min. Then, quinacridine **2** [19] (0.1 mmol) was added and the corresponding α-diazomalonate (2 equiv) at the end. The vial was sealed and allowed to stir at 60 °C for 3 h. After complete consumption of the starting quinacridine **2** (monitored by ESI-MS), the crude reaction mixture was allowed to cool to room temperature. The solvent was then removed under reduced pressure and the residue was purified by flash chromatography (Si_2O, CH_2Cl_2/MeOH gradient from 100:0 to 99:1) affording the titled quinacridines **3** as dark purple solids.

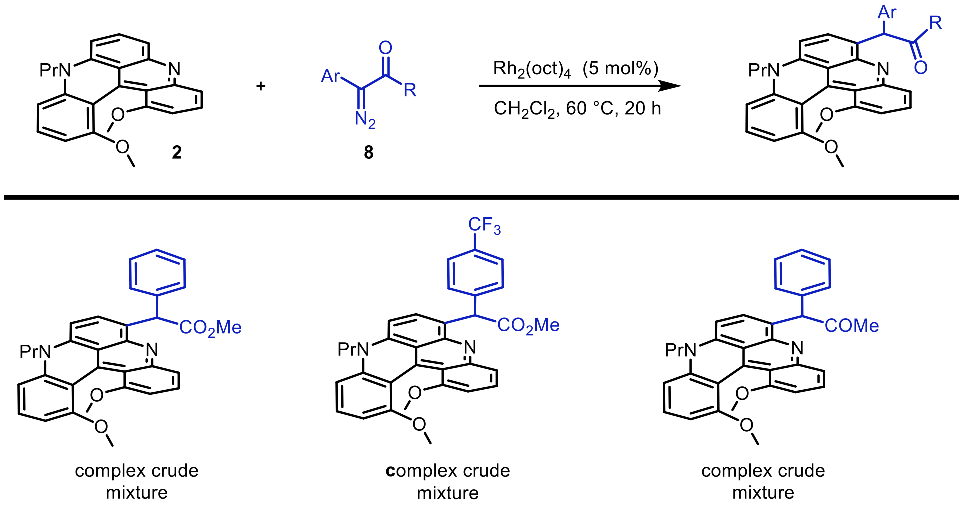

Scheme S1. Scope of α-diazo arylacetates **8** – unsuccessful attempts

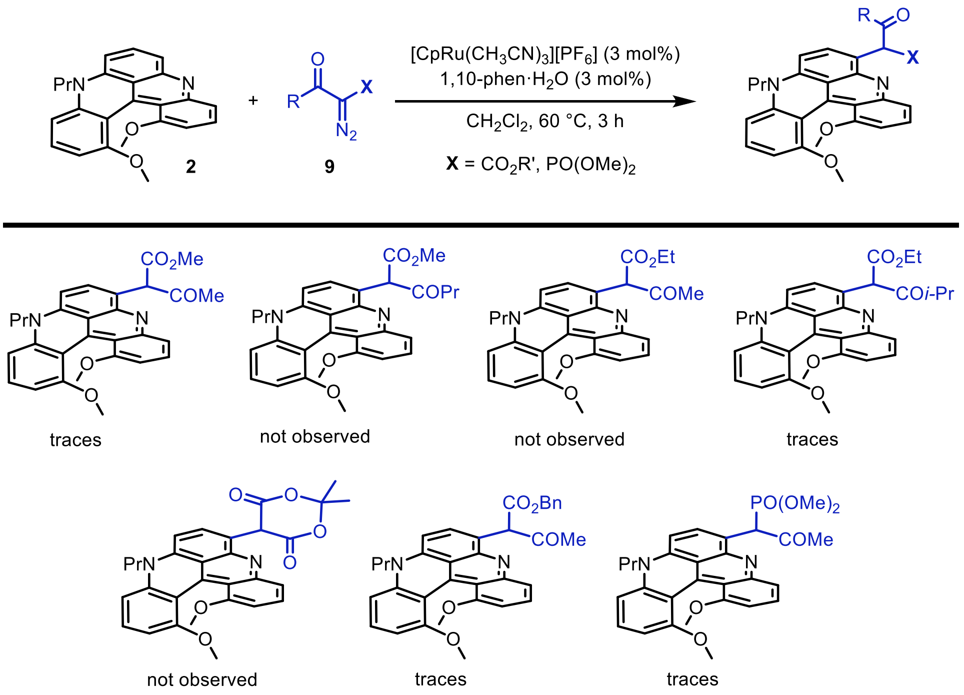

Scheme S2. Scope of α-diazodicarbonyles and phosphonate **9** – unsuccessful attempts

Dimethyl 2-(1,13-dimethoxy-5-propyl-5*H*-quinolino[2,3,4-*kl*]acridin-8-yl)malonate (3a)

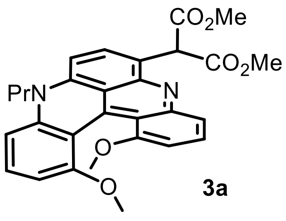

3a

Compound **3a** is prepared according to the general procedure, using 40 mg (0.1 mmol) quinacridine **2**, 1.41 mg (3.2 µmol) [CpRu(CH_3CN)$_3$][PF_6], 0.58 mg (3.2 µmol) 1,10-phenanthroline·H_2O and 25 µL (0.2 mmol) dimethyl 2-diazomalonate in 0.22 mL dry CH_2Cl_2. The desired compound was isolated as a dark purple solid (38 mg, 69%).

***R*f** (SiO_2, CH_2Cl_2/MeOH, 97:3) = 0.42. **^{1}H NMR (500 MHz, CD_2Cl_2):** δ = 7.70 (d, *J* = 8.1 Hz, 1H, CH), 7.58-7.54 (m, 2H, CH), 7.49 (t, *J* = 8.3 Hz, 1H, CH), 6.93 (d, *J* = 8.5 Hz, 1H, CH), 6.68 (d, *J* = 8.1 Hz, 1H, CH), 6.63-6.59 (m, 2H, CH), 6.19 (s, 1H, CH), 4.26-4.18 (m, 1H, CH_2), 3.96-3.88 (m, 1H, CH_2), 3.76 (s, 3H, OCH_3), 3.75 (s, 3H, OCH_3), 3.73 (s, 3H, OCH_3), 3.66 (s, 3H, OCH_3), 2.12-2.02 (m, 1H, CH_2), 2.01-1.90 (m, 1H, CH_2), 1.16 (t, *J* = 7.4 Hz, 3H, CH_3) ppm. **^{13}C NMR (126 MHz, CD_2Cl_2):** δ = 170.55 (CO_2Me), 170.53 (CO_2Me), 159.85 (C), 158.31 (C), 150.98 (C), 146.42 (C), 143.17 (C), 139.22 (C), 133.94 (C), 132.22 (CH), 130.43 (CH), 130.39 (CH), 121.19 (CH), 120.46 (C), 118.48 (C), 114.90 (C), 112.73 (C), 106.23 (CH), 101.96 (CH), 100.85 (CH), 98.90 (CH), 55.40 (OCH_3), 55.36 (OCH_3), 52.91 (OCH_3), 52.88 (OCH_3), 51.73 (CH), 50.10 (CH_2), 18.92 (CH_2), 11.34 (CH_3) ppm. **IR (neat, cm^{-1}):** ν = 2957, 1729, 1600, 1574, 1539, 1461, 1433, 1389, 1347, 1247, 1160, 1110, 1072, 1043, 824, 780, 752, 592. **HRMS (ESI)** calculated for [M+]: 501.2021, Found 501.2016.

Diethyl 2-(1,13-dimethoxy-5-propyl-5*H*-quinolino[2,3,4-*kl*]acridin-8-yl)malonate (3b)

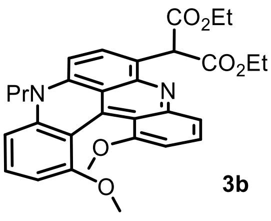

3b

Compound **3b** is prepared according to the general procedure, using 40 mg (0.1 mmol) quinacridine **2**, 1.41 mg (3.2 µmol) [CpRu(CH_3CN)$_3$][PF_6], 0.58 mg (3.2 µmol) 1,10-phenanthroline·H_2O and 36 µL (0.2 mmol) diethyl 2-diazomalonate in 0.22 mL dry CH_2Cl_2. The desired compound was isolated as a dark purple solid (29 mg, 50%).

***R*f** (SiO_2, CH_2Cl_2/MeOH, 97:3) = 0.42. **^{1}H NMR (500 MHz, CD_2Cl_2):** δ = 7.70 (d, *J* = 8.1 Hz, 1H, CH), 7.59-7.53 (m, 2H, CH), 7.49 (t, *J* = 8.3 Hz, 1H, CH), 6.93 (d, *J* = 8.2 Hz, 1H, CH), 6.68 (d, *J* = 8.2 Hz, 1H, CH), 6.63-6.58 (m, 2H, CH), 6.10 (s, 1H, CH), 4.27-4.18 (m, 5H, CH_2), 3.97-3.88 (m, 1H, CH_2), 3.73 (s, 3H, OCH_3), 3.66 (s, 3H, OCH_3), 2.13-2.02 (m, 1H, CH_2), 2.02-1.90 (m, 1H, CH_2), 1.30-1.25 (m, 6H, CH_3), 1.17 (t, *J* = 7.4 Hz, 3H, CH_3) ppm. **^{13}C NMR (126 MHz, CD_2Cl_2):** δ = 170.10 (CO_2Et), 170.06 (CO_2Et), 159.85 (C), 158.32 (C), 150.86 (C), 146.42 (C), 143.20 (C), 139.12 (C), 133.92 (C), 132.20 (CH), 130.35 (2 x CH), 121.13 (CH), 120.44 (C), 118.71 (C), 114.89 (C), 112.74 (C), 106.22 (CH), 101.94 (CH), 100.83 (CH), 98.92 (CH), 61.85 (CH_2), 61.81 (CH_2), 55.40 (OCH_3), 55.36 (OCH_3), 52.29 (CH), 50.10 (CH_2), 18.92 (CH_2), 14.36 (2 x CH_3), 11.33 (CH_3) ppm. **IR (neat, cm^{-1}):** ν = 2931, 1727, 1600, 1574, 1539, 1461, 1389, 1367, 1347, 1311, 1247, 1214, 1159, 1111, 1073, 1035, 940, 824, 778, 749, 591, 537. **HRMS (ESI)** calculated for [M+]: 529.2334, Found 529.2332.

Diisopropyl 2-(1,13-dimethoxy-5-propyl-5*H*-quinolino[2,3,4-*kl*]acridin-8-yl)malonate (3c)

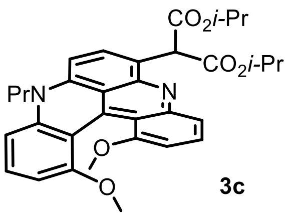

3c

Compound **3c** is prepared according to the general procedure, using 40 mg (0.1 mmol) quinacridine **2**, 1.41 mg (3.2 µmol) [CpRu(CH_3CN)$_3$][PF_6], 0.58 mg (3.2 µmol) 1,10-phenanthroline·H_2O and 43 µL (0.2 mmol) diisopropyl 2-diazomalonate in 0.22 mL dry CH_2Cl_2. The desired compound was isolated as a dark purple solid (37 mg, 62%).

***R*f** (SiO_2, CH_2Cl_2/MeOH, 97:3) = 0.5. **^{1}H NMR (500 MHz, CD_2Cl_2):** δ = 7.69 (d, *J* = 8.7 Hz, 1H, CH), 7.57-7.54 (m, 2H, CH), 7.48 (t, *J* = 8.3 Hz, 1H, CH), 6.93 (d, *J* = 8.8 Hz, 1H, CH), 6.68 (d, *J* = 8.1 Hz, 1H, CH), 6.63-6.58 (m, 2H, CH), 6.01 (s, 1H, CH), 5.14-5.04 (m, 2H, CH), 4.26-4.18 (m, 1H, CH_2), 3.95-3.88 (m, 1H, CH_2), 3.73 (s, 3H, OCH_3), 3.66 (s, 3H, OCH_3), 2.12-2.03 (m, 1H, CH_2), 2.01-1.91 (m, 1H, CH_2), 1.30-1.26 (m, 12H, CH_3), 1.17 (t, *J* = 7.5 Hz, 3H, CH_3) ppm. **^{13}C NMR (126 MHz, CD_2Cl_2):** δ = 169.56 (CO_2*i*-Pr), 169.52 (CO_2*i*-Pr), 159.92 (C), 158.31 (C), 150.89 (C), 146.54 (C), 143.22 (C), 139.02 (C), 133.80 (C), 132.13 (CH), 130.25 (CH), 130.14 (CH), 121.21 (CH), 120.45 (C), 119.08 (C), 114.88 (C), 112.73 (C), 106.20 (CH), 101.89 (CH), 100.79 (CH), 98.87 (CH), 69.18 (CH), 69.13 (CH), 55.39 (OCH_3), 55.35 (OCH_3), 52.77 (CH), 50.09 (CH_2), 21.87 (2 x CH_3), 21.84 (2 x CH_3), 18.90 (CH_2), 11.34 (CH_3) ppm. **IR (neat, cm^{-1}):** ν = 3674, 2974, 1723, 1600, 1574, 1539, 1459, 1386, 1249, 1161, 1073, 939, 902, 825, 750, 590. **HRMS (ESI)** calculated for [M+]: 557.2660, Found 557.2664.

Di-*tert*-butyl 2-(1,13-dimethoxy-5-propyl-5*H*-quinolino[2,3,4-*kl*]acridin-8-yl)malonate (3d)

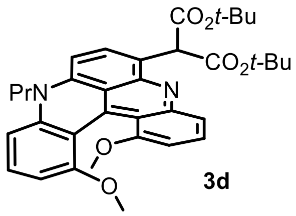

3d

Compound **3d** is prepared according to the general procedure, using 40 mg (0.1 mmol) quinacridine **2**, 1.41 mg (3.2 µmol) [CpRu(CH_3CN)$_3$][PF_6], 0.58 mg (3.2 µmol) 1,10-phenanthroline·H_2O and 66 µL (0.2 mmol) di-*tert*-butyl 2-diazomalonate in 0.22 mL dry CH_2Cl_2. The desired compound was isolated as a dark purple solid (28 mg, 44%). ***R*$_f$** (SiO_2, CH_2Cl_2/MeOH, 97:3) = 0.38. **^{1}H NMR (400 MHz, CD_2Cl_2):** δ = 7.72 (d, *J* = 8.2 Hz, 1H, CH), 7.58-7.53 (m, 2H, 2 x CH), 7.48 (t, *J* = 8.4 Hz, 1H, CH), 6.92 (d, *J* = 8.7 Hz, 1H, CH), 6.68 (d, *J* = 8.1 Hz, 1H, CH), 6.64-6.52 (m, 2H, 2 x CH), 5.87 (s, 1H, CH), 4.26-4.18 (m, 1H, CH_2), 3.96-3.88 (m, 1H, CH_2), 3.73 (s, 3H, OCH_3), 3.66 (s, 3H, OCH_3), 2.11-2.02 (m, 1H, CH_2), 2.02-1.91 (m, 1H, CH_2), 1.51 (s, 9H, 3 x CH_3), 1.49 (s, 9H, 3 x CH_3), 1.17 (t, *J* = 7.5 Hz, 3H, CH_3) ppm. **^{13}C NMR (126 MHz, CD_2Cl_2):** δ = 169.27 (CO_2*t*-Bu), 169.21 (CO_2*t*-Bu), 159.85 (C), 158.32 (C), 150.98 (C), 146.71 (C), 143.28 (C), 138.84 (C), 133.34 (C), 132.07 (CH), 130.14 (CH), 129.97 (CH), 121.35 (CH), 120.49 (C), 119.74 (C), 114.87 (C), 112.74 (C), 106.18 (CH), 101.83 (CH), 100.75 (CH), 98.91 (CH), 81.58 (C), 81.47 (C), 55.39 (OCH_3), 55.35 (OCH_3), 54.04 (CH), 50.08 (CH_2), 28.16 (3 x CH_3), 28.14 (3 x CH_3), 18.90 (CH_2), 11.34 (CH_3) ppm. **IR (neat, cm^{-1}):** ν = 2923, 1722, 1600, 1576, 1539, 1460, 1391, 1367, 1250, 1158, 1074, 940, 848, 825, 772, 743, 621, 592, 563. **HRMS (ESI)** calculated for [M+]: 585.2959, Found 585.2962.

Dibenzyl 2-(1,13-dimethoxy-5-propyl-5*H*-quinolino[2,3,4-*kl*]acridin-8-yl)malonate (3e)

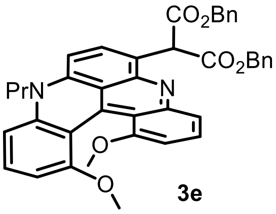

3e

Compound **3e** is prepared according to the general procedure, using 40 mg (0.1 mmol) of quinacridine **2**, 1.41 mg (3.2 µmol) [CpRu(CH_3CN)$_3$][PF_6], 0.58 mg (3.2 µmol) 1,10-phenanthroline·H_2O and 67 mg (0.2 mmol) dibenzyl 2-diazomalonate in 0.22 mL dry CH_2Cl_2. The desired compound was isolated as a dark purple solid (54 mg, 77%). ***R*$_f$** (SiO_2, CH_2Cl_2/MeOH, 99:1) = 0.33. **^{1}H NMR (500 MHz, CD_2Cl_2):** δ = 7.67 (d, *J* = 8.1 Hz, 1H, CH), 7.59-7.46 (m, 3H,3 x CH), 7.39-7.23 (m, 10H, 2 x Ph), 6.94 (d, *J* = 8.5 Hz, 1H, CH), 6.70-6.56 (m, 3H, CH), 6.25 (s, 1H, CH), 5.22 (d, *J* = 13.8 Hz 4H, 2 x CH_2), 4.22 (ddd, *J* = 15.1, 11.6, 5.3 Hz, 1H, CH_2), 3.92 (ddd, *J* = 15.6, 11.4, 5.0 Hz, 1H, CH_2), 3.73 (s, 3H, OCH_3), 3.66 (s, 3H, OCH_3), 2.10-2.02 (m, 1H, CH_2), 2.01-1.91 (m, 1H, CH_2), 1.16 (t, *J* = 7.4 Hz, 3H, CH_3) ppm. **^{13}C NMR (126 MHz, CD_2Cl_2):** δ = 169.85, (CO_2Bn), 169.80 (CO_2Bn), 159.85 (C), 158.30 (C), 150.95 (C), 146.46 (C), 143.17 (C), 139.25 (C), 136.38 (C), 133.86 (C), 132.17 (CH), 130.40 (CH), 130.33 (CH), 128.82 (CH), 128.80 (CH), 128.42 (2 x CH), 128.38 (2 x CH), 128.31 (2 x CH), 128.22 (2 x CH), 121.26 (CH), 120.44 (C), 120.12 (C), 118.43 (C), 114.91 (C), 112.75 (C), 106.22 (CH), 101.95 (CH), 100.84 (CH), 98.81 (CH), 67.40 (CH_2), 67.33 (CH_2), 55.40 (OCH_3), 55.35 (OCH_3), 52.42 (CH), 50.07 (CH_2), 18.91 (CH_2), 11.34 (CH_3) ppm. **IR (neat, cm^{-1}):** ν = 2926, 1731, 1600, 1574, 1539, 1456, 1377, 1249, 1160, 1111, 1073, 824, 778, 735, 695, 592, 515. **HRMS (ESI)** calculated for [M+]: 653.2646, Found 653.2670.

1-Benzyl 3-methyl 2-(1,13-dimethoxy-5-propyl-5*H*-quinolino[2,3,4-*kl*]acridin-8-yl)malonate (3f)

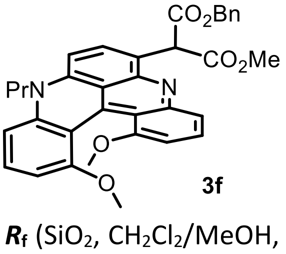

3f

Compound **3f** is prepared according to the general procedure, using 40 mg (0.1 mmol) quinacridine **2**, 1.41 mg (3.2 µmol) [CpRu(CH_3CN)$_3$][PF_6], 0.58 mg (3.2 µmol) 1,10-phenanthroline·H_2O and 51 mg (0.2 mmol) 1-benzyl 3-methyl 2-diazomalonate in 0.22 mL dry CH_2Cl_2. The desired compound was isolated as a dark purple solid (41 mg, 66%) in a 1:1 ratio (diastereomeric ratio was determined by ^{1}H NMR analysis of the crude reaction mixture, the two diastereoisomers are assigned with indices *a* and *b*).

***R*$_f$** (SiO_2, CH_2Cl_2/MeOH, 98:2) = 0.45. **^{1}H NMR (500 MHz, CD_2Cl_2):** δ = 7.68 (dd, *J* = 8.1, 2.0 Hz, 1H, CH), 7.59-7.52 (m, 2H, CH), 7.48 (t, *J* = 8.3 Hz, 1H, CH) 7.38-7.24 (m, 5H, CH), 6.93 (d, *J* = 8.8 Hz, 1H, CH), 6.66 (d, *J* = 8.1 Hz, 1H, CH), 6.63-6.58 (m, 2H, CH), 6.23 (s, 0.45H, CH_a), 6.22 (s, 0.45H, CH_b), 5.23 (d, *J* = 13.1 Hz, 2H, CH_2), 4.21(ddd, *J* = 15.1, 11.6, 5.3 Hz, 1H, CH_2), 3.91 (ddd, *J* = 15.8, 11.6, 5.0 Hz, 1H, CH_2), 3.77 (s, 1.50H, OCH_{3a}), 3.75 (s, 1.50H, OCH_{3b}), 3.73 (s, 3H, OCH_3), 3.66 (s, 3H, OCH_3), 2.10-2.02 (m, 1H, CH_2), 2.00-1.90 (m, 1H, CH_2), 1.16 (t, *J* = 7.4 Hz, 3H, CH_3) ppm. **^{13}C NMR (126 MHz, CD_2Cl_2):** δ = 170.53, (0.5C, CO_2Me), 170.48 (0.5C, CO_2Me), 169.88, (0.5C, CO_2Bn), 169.85

(0.5C, CO_2Bn), 159.84 (C), 158.30 (C), 150.99 (C), 146.47 (C), 143.16 (C), 139.24 (C), 136.45 (C), 133.84 (C), 132.17 (CH), 130.39 (0.5C, CH), 130.35 (0.5C, CH), 130.32 (CH), 128.81 (CH), 128.79 (CH), 128.40 (CH), 128.37 (CH), 128.24 (0.5C, CH), 128.17 (0.5C, CH), 121.27 (0.5C, CH), 121.25 (0.5C, CH), 120.44 (C), 118.50 (C), 114.93 (0.5C, C), 114.91 (0.5C, C), 112.73 (C), 106.22 (CH), 101.95 (CH), 100.84 (0.5C, CH), 100.82 (0.5C, CH), 98.82 (CH), 67.34 (0.5C, CH_2), 67.27 (0.5C, CH_2), 55.39 (OCH_3), 55.35 (OCH_3), 52.89 (0.5C, OCH_3), 52.87 (0.5C, OCH_3), 52.15 (0.5C, CH), 52.13 (0.5C, CH), 50.07 (CH_2), 18.91 (CH_2), 11.34 (CH_3) ppm. **IR (neat, cm^{-1}):** ν = 2929, 1730, 1601, 1573, 1539, 1486, 1456, 1433, 1385, 1346, 1314, 1248, 1214, 1143, 1111, 1072, 1042, 824, 779, 750, 697, 591, 549. **HRMS (ESI)** calculated for [M+]: 577.2334, Found 577.2330.

Di (2,2,2-trifluoroethyl) 2-(1,13-dimethoxy-5-propyl-5*H*-quinolino[2,3,4-*kl*]acridin-8-yl)malonate (3g)

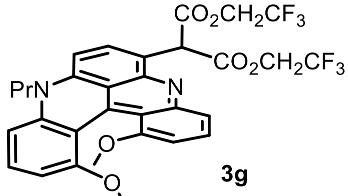

Compound **3g** is prepared according to the general procedure, using 40 mg (0.1 mmol) quinacridine **2**, 1.41 mg (3.2 µmol) [$CpRu(CH_3CN)_3$][PF_6], 0.58 mg (3.2 µmol) 1,10-phenanthroline·H_2O and 34 µL (0.2 mmol) bis (2,2,2-trifluoroethyl) 2-diazomalonate in 0.22 mL dry CH_2Cl_2. The desired compound was isolated as a dark purple solid (12 mg, 18%).

***R*f** (SiO_2, CH_2Cl_2/MeOH, 97:3) = 0.31. **^{1}H NMR (500 MHz, CD_2Cl_2):** δ = 7.75-7.67 (m, 2H, 2 x CH), 7.66-7.58 (m, 2H, 2 x CH), 7.49 (t, *J* = 8.3 Hz, 1H, CH), 7.10 (dd, *J* = 8.4, 0.9 Hz, 1H, CH), 6.75-6.70 (m, 1H, CH), 6.66 (dd, *J* = 8.1, 0.9 Hz, 1H, CH), 5.37 (s, 1H, CH), 4.80-4.74 (m, 1H, CH_2CF_3), 4.70-4.64 (m, 1H, CH_2CF_3), 4.60-4.55 (m, 1H, CH_2CF_3), 4.52-4.43 (m, 2H, 2 x CH_2), 3.76 (s, 3H, OCH_3), 3.66 (s, 3H, OCH_3), 3.63-3.56 (m, 1H, CH_2), 1.54-1.49 (m, 1H, CH_2), 1.47-1.41 (m, 1H, CH_2), 0.48 (t, *J* = 7.4 Hz, 3H, CH_3) ppm. **^{13}C NMR (126 MHz, CD_2Cl_2):** δ = 167.25 ($CO_2CH_2CF_3$), 166.61 ($CO_2CH_2CF_3$), 159.09 (C), 158.32 (C), 150.74 (C), 146.49 (C), 145.50 (C), 141.77 (C), 132.11 (CH), 131.65 (CH), 131.13 (CH), 123.98 (C), 123.15 (q, *J* = 277.4, CF_3), 123.01 (q, *J* = 277.4, CF_3), 121.36 (C), 120.21 (CH), 120.05 (CH), 118.52 (C), 115.63 (C), 111.85 (CH), 110.44 (C), 103.54 (CH), 102.17 (CH), 61.85 (q, *J* = 36.9 Hz, CH_2), 61.74 (q, *J* = 36.8 Hz, CH_2), 59.53 (CH_2), 55.72 (OCH_3), 55.47 (OCH_3), 53.46 (CH), 21.58 (CH_2), 10.81 (CH_3) ppm. **^{19}F NMR** (282 MHz, CD_2Cl_2): δ = −74.07 ppm. **IR (neat, cm^{-1}):** ν = 2968, 1750, 1690, 1637, 1563, 1499, 1463, 1408, 1341, 1265, 1150, 1087, 968, 832, 780, 753, 699, 646, 551, 532. **HRMS (ESI)** calculated for [M+]: 637.1768, Found 637.1758.

1,14-dimethoxy-10-propyl-7-((2,2,2-trifluoroethoxy)carbonyl)-10*H*-pyrrolo[3,2,1-*de*]quinolino[4,3,2-*mn*]acridin-14*b*-ylium-6-olate tetrafluoroborate (10)

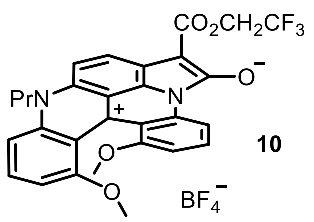

Compound **10** is isolated as a dark green solid (22 mg, 32%).

***R*f** (SiO_2, CH_2Cl_2/MeOH, 95:5) = 0.32. **^{1}H NMR (500 MHz, CD_2Cl_2):** δ = 9.51 (dd, *J* = 8.7, 1.0 Hz, 1H, CH), 8.56 (d, *J* = 8.4 Hz, 1H, CH), 7.90-7.81 (m, 2H, 2 x CH), 7.55 (d, *J* = 8.5 Hz, 1H, CH), 7.44 (d, *J* = 9.0 Hz, 1H, CH), 6.92 (dd, *J* = 8.1, 1.0 Hz, 1H, CH), 6.80 (d, *J* = 7.9 Hz, 1H, CH), 4.77 (qd, *J* = 9.0, 3.7 Hz, 2H, CH_2), 4.71 (ddd, *J* = 16.3, 10.2, 5.2 Hz, 1H, CH_2), 4.57 (ddd, *J* = 15.1, 10.1, 5.4 Hz, 1H, CH_2), 3.79 (s, 3H, OCH_3), 3.78 (s, 3H, OCH_3), 2.19-2.08 (m, 2H, CH_2), 1.24 (t, *J* = 7.4 Hz, 3H, CH_3) ppm. **^{13}C NMR (126 MHz, CD_2Cl_2):** δ = 165.48 ($CO_2CH_2CF_3$), 164.26 (CO), 160.57 (C), 159.86 (C), 141.24 (C), 140.99 (C), 140.72 (C), 135.94 (CH), 135.59 (CH), 134.69 (C), 132.47 (C), 127.56 (CH), 124.88 (q, *J* = 277.5, CF_3), 121.57 (C), 119.71 (C), 113.92 (C), 113.53 (C), 113.13 (C), 109.56 (CH), 107.63(CH), 104.41 (CH), 104.04 (CH), 101.72 (CH), 59.07 (q, *J* = 35.3 Hz, CH_2), 55.92 (OCH_3), 55.75 (OCH_3), 51.37 (CH_2), 20.87 (CH_2), 11.44 (CH_3) ppm. **^{19}F NMR** (282 MHz, CD_2Cl_2): δ = −153.18, −153.12, −73.95 ppm. **IR (neat, cm^{-1}):** ν =3106, 2966, 1718, 1643, 1602, 1583, 1555, 1504, 1448, 1427, 1404, 1359, 1339, 1297, 1257, 1216, 1146, 1112, 1072, 1044, 1012, 972, 946, 892, 837, 800, 769, 746, 728, 654, 628, 586, 561. **HRMS (ESI)** calculated for [M+]: 536.1554, Found 536.1543.

Procedure for synthesis and characterization of **r-3a**

To a solution of substrate **2** (0.1 mmol) and $Rh_2(oct)_4$ (5 mol%) in dry CH_2Cl_2 (0.5 M) dimethyl 2-diazomalonate (25 µL, 0.2 mmol) **1a** was added. The vial was sealed and allowed to stir at 60 °C for 9 h. After complete consumption of the starting quinacridine **2** (monitored by ESI-MS), the crude reaction mixture was allowed to cool to room temperature. The solvent was then removed under reduced pressure and the residue was purified by flash chromatography (Si_2O, CH_2Cl_2/MeOH gradient from 100:0 to 99:1). Derivative **r-3a** was isolated as a dark red solid (18 mg, 33%).[20]

Dimethyl 2-(1,13-dimethoxy-5-propyl-5H-quinolino[2,3,4-kl]acridin-6-yl)malonate (r-3a)

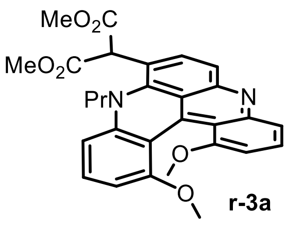

r-3a

R_f (SiO_2, CH_2Cl_2/MeOH, 96:4) = 0.36. **^{1}H NMR (500 MHz, CD_2Cl_2):** δ = 7.73 (d, *J* = 9.1 Hz, 1H, CH), 7.68-7.56 (m, 3H, CH), 7.44 (t, *J* = 8.2 Hz, 1H, CH), 7.06 (dd, *J* = 8.5, 0.9 Hz, 1H, CH), 6.69 (dd, *J* = 7.1, 1.5 Hz, 1H, CH), 6.62 (dd, *J* = 8.2, 0.9 Hz, 1H, CH), 5.14 (s, 1H, CH), 4.38 (ddd, *J* = 13.4, 8.5, 4.6 Hz, 1H, CH_2), 3.86 (s, 3H, OCH_3), 3.75 (s, 3H, OCH_3), 3.66 (s, 3H, OCH_3), 3.65 (s, 3H, OCH_3), 3.63-3.57 (m, 1H, CH_2), 1.55-1.44 (m, 1H, CH_2), 1.41-1.31 (m, 1H, CH_2), 0.50 (t, *J* = 7.4 Hz, 3H, CH_3) ppm. **^{13}C NMR (126 MHz, CD_2Cl_2):** δ = 169.69 (CO_2Me), 169.26 (CO_2Me), 158.96 (C), 158.25 (C), 151.44 (C), 147.53 (C), 145.99 (C), 140.94 (C), 132.68 (C), 132.20 (CH), 131.04 (CH), 130.19 (CH), 124.36 (CH), 121.23 (C), 120.41 (C), 118.56 (C), 115.75 (C), 112.46 (CH), 111.84 (CH), 103.26 (CH), 101.83 (CH), 59.19 (CH_2), 55.66 (OCH_3), 55.40 (OCH_3), 54.22 (CH), 53.32 (OCH_3), 53.26 (OCH_3), 21.54 (CH_2), 10.99 (CH_3) ppm. **IR (neat, cm^{-1}):** ν = 2947, 1759, 1737, 1599, 1574, 1558, 1339, 1431, 1331, 1246, 1143, 1102, 1083, 1023, 831, 785, 756, 740, 710, 645. **HRMS (ESI)** calculated for [M+]: 501.2021, Found 501.2043.

General procedure for synthesis of bis functionalized quinacridines **4**

To a solution of mono functionalized quinacridines **3a-c** (0.1 mmol) and $Rh_2(oct)_4$ (5 mol%) in dry CH_2Cl_2 (0.5 M) the corresponding α-diazomaloate (2 equiv) was added. The vial was sealed and allowed to stir at 60 °C for 22 h. After complete consumption of the starting quinacridines **3a-c** (monitored by ESI-MS), the crude reaction mixture was allowed to cool to room temperature. The solvent was then removed under reduced pressure and the residue was purified by flash chromatography (Si_2O, CH_2Cl_2/MeOH gradient from 100:0 to 99:1) affording the titled quinacridines **4** as dark red solids.

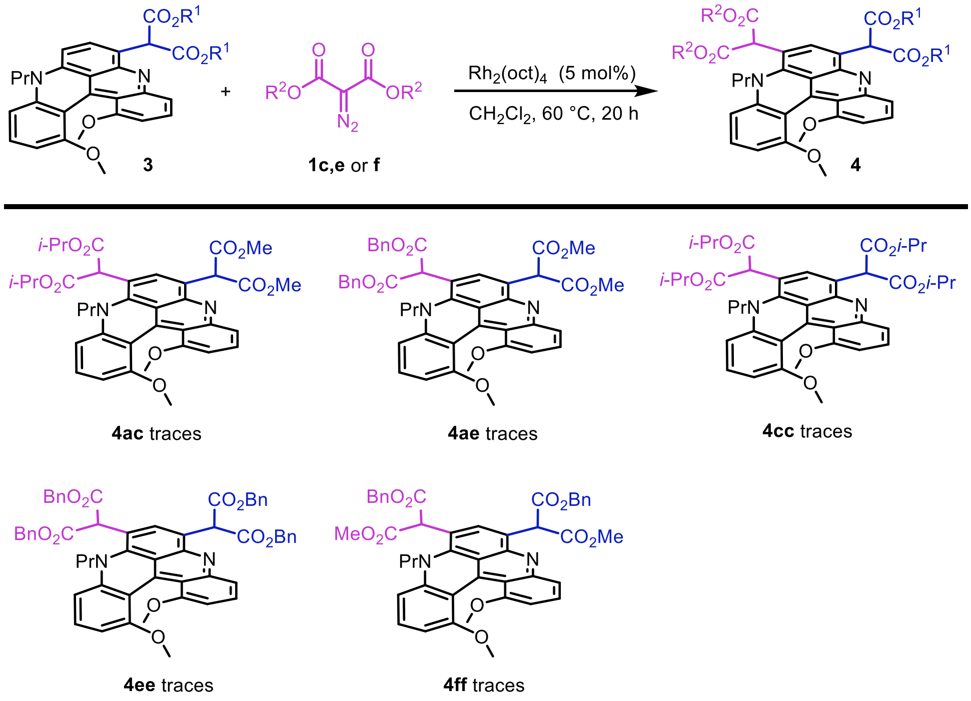

Scheme S3. Scope of α-diazo malonates **1** for bis substitution – unsuccessful attempts

Tetramethyl 2,2'-(1,13-dimethoxy-5-propyl-5*H*-quinolino[2,3,4-*kl*]acridine-6,8-diyl)dimalonate (4aa)

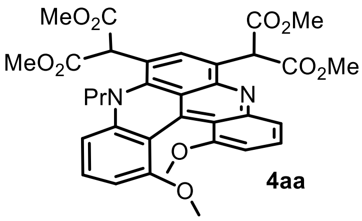

Compound **4aa** is prepared according to the general procedure, using 25 mg (0.05 mmol) of quinacridine **3a**, 1.95 mg (2.5 µmol) of $Rh_2(oct)_4$ and 11 µL (0.10 mmol) of dimethyl 2-diazomalonate in 0.1 mL of dry CH_2Cl_2. The desired compound was isolated as a dark red solid (17 mg, 54%).

***R*$_f$** (SiO_2, CH_2Cl_2/MeOH, 99:1) = 0.33. **^{1}H NMR (500 MHz, CD_2Cl_2):** δ = 7.80 (s, 1H, CH), 7.66-7.58 (m, 2H, 2 x CH), 7.45 (t, *J* = 8.3 Hz, 1H, CH), 7.06 (d, *J* = 9.4 Hz, 1H, CH), 6.70 (dd, *J* = 6.9, 1.8 Hz, 1H, CH), 6.62 (d, *J* = 8.1 Hz, 1H, CH), 6.18 (s, 1H, CH), 5.13 (s, 1H, CH), 4.41(ddd, *J* = 13.5, 8.7, 4.5 Hz, 1H, CH_2), 3.87 (s, 3H, OCH_3), 3.78 (s, 3H, OCH_3), 3.76 (s, 3H, OCH_3), 3.74 (s, 3H, OCH_3), 3.67 (s, 3H, OCH_3), 3.65-3.61 (m, 1H, CH_2), 3.63 (s, 3H, OCH_3), 1.60-1.52 (m, 1H, CH_2), 1.43-1.35 (m, 1H, CH_2), 0.54 (t, *J* = 7.4 Hz, 3H, CH_3) ppm. **^{13}C NMR (126 MHz, CD_2Cl_2):** δ = 170.06 (CO_2Me), 169.99 (CO_2Me), 169.46 (CO_2Me), 169.08 (CO_2Me), 158.97 (C), 158.19 (C), 150.68 (C), 145.33 (C), 144.95 (C), 141.05 (C), 133.22 (C), 132.35 (CH), 131.17 (CH), 130.44 (CH), 124.03 (C), 122.50 (C), 121.34 (CH), 118.32 (C), 115.73 (C), 111.49 (CH, C), 103.24 (CH), 102.10 (CH), 58.87 (CH_2), 55.69 (OCH_3), 55.40 (OCH_3), 54.35 (CH), 53.35 (OCH_3), 53.26 (OCH_3), 52.93 (2 x OCH_3), 51.86 (CH), 21.63 (CH_2), 10.95 (CH_3) ppm. **IR (neat, cm^{-1}):** ν = 2953, 1733, 1597, 1566, 1472, 1433, 1313, 1258, 1198, 1144, 1112, 1090, 1049, 1024, 973, 931, 826, 784, 754, 732, 690, 592, 511. **HRMS (ESI)** calculated for [M+]: 631.2287, Found 631.2261.

Diethyl 2-(8-(1,3-dimethoxy-1,3-dioxopropan-2-yl)-1,13-dimethoxy-5-propyl-5*H*-quinolino[2,3,4-*kl*]-acridin-6-yl)malonate (4ab)

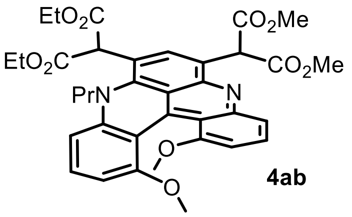

4ab

Compound **4ab** is prepared according to the general procedure, using 25 mg (0.05 mmol) of quinacridine **3a**, 1.95 mg (2.5 µmol) of $Rh_2(oct)_4$ and 12 µL (0.10 mmol) of diethyl 2-diazomalonate in 0.1 mL of dry CH_2Cl_2. The desired compound was isolated as a dark red solid (15 mg, 45%).
***R*f** (SiO_2, CH_2Cl_2/MeOH, 99:1) = 0.33. **^{1}H NMR (500 MHz, CD_2Cl_2):** δ 7.81 (s, 1H, CH), 7.66-7.59 (m, 2H, 2 x CH), 7.44 (t, *J* = 8.3 Hz, 1H, CH), 7.06 (dd, *J* = 8.4, 1.0 Hz, 1H, CH), 6.70 (dd, *J* = 6.9, 1.8 Hz, 1H, CH), 6.62 (d, *J* = 8.1 Hz, 1H, CH), 6.17 (s, 1H, CH), 5.08 (s, 1H, CH), 4.41 (ddd, *J* = 13.5, 8.7, 4.5 Hz, 1H, CH_2), 4.32 (qd, *J* = 7.1, 5.2 Hz, 2H, CH_2), 4.14 (q, *J* = 7.1 Hz, 2H, CH_2), 3.77 (s, 3H, OCH_3), 3.75 (s, 3H, OCH_3), 3.74 (s, 3H, OCH_3), 3.70-3.65 (m, 1H, CH_2), 3.64 (s, 3H, OCH_3), 1.56-1.53 (m, 1H, CH_2), 1.42-1.37 (m, 1H, CH_2), 1.32 (t, *J* = 7.1 Hz, 3H, CH_3), 1.18 (t, *J* = 7.2 Hz, 3H, CH_3), 0.54 (t, *J* = 7.4 Hz, 3H, CH_3) ppm. **^{13}C NMR (126 MHz, CD_2Cl_2):** δ = 170.09 (CO_2Me), 169.98 (CO_2Me), 169.03 (CO_2Et), 168.61 (CO_2Et), 158.98 (C), 158.19 (C), 150.68 (C), 145.43 (C), 145.00 (C), 140.97 (C), 133.17 (C), 132.36 (CH), 131.12 (CH), 130.36 (CH), 124.09 (C), 122.41 (C), 121.37 (CH), 118.34 (C), 115.72 (C), 111.79 (C), 111.52 (CH), 103.18 (CH), 102.07 (CH), 62.46 (CH_2), 62.43 (CH_2), 58.84 (CH_2), 55.70 (OCH_3), 55.40 (OCH_3), 54.75 (CH), 52.90 (OCH_3), 52.88 (OCH_3), 51.91 (CH), 21.65 (CH_2), 14.32 (CH_3), 14.12 (CH_3), 10.98 (CH_3) ppm. **IR (neat, cm^{-1}):** ν = 2965, 1718, 1643, 1601, 1583, 1556, 1505, 1448, 1405, 1360, 1339, 1297, 1217, 1145, 1112, 1072, 1045, 1013, 972, 946, 892, 851, 837, 800, 770, 747, 728, 654, 560, 548, 525. **HRMS (ESI)** calculated for [M+]: 659.2600, Found 659.2564.

Tetraethyl 2,2'-(1,13-dimethoxy-5-propyl-5*H*-quinolino[2,3,4-*kl*]acridine-6,8-diyl)dimalonate (4bb)

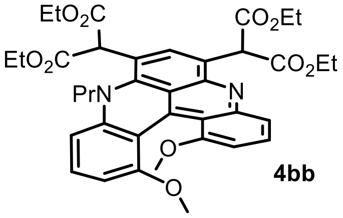

4bb

Compound **4bb** is prepared according to the general procedure, using 26 mg (0.05 mmol) of quinacridine **3b**, 1.95 mg (2.5 µmol) of $Rh_2(Oct)_4$ and 12 µL (0.10 mmol) of diethyl 2-diazomalonate in 0.1 mL of dry CH_2Cl_2. The desired compound was isolated as a dark red solid (17 mg, 50%).
***R*f** (SiO_2, CH_2Cl_2/MeOH, 99:1) = 0.37. **^{1}H NMR (500 MHz, CD_2Cl_2):** δ 7.82 (s, 1H, CH), 7.65-7.58 (m, 2H, 2 x CH), 7.44 (t, *J* = 8.2 Hz, 1H, CH), 7.06 (dd, *J* = 8.4, 1.0 Hz, 1H, CH), 6.70 (dd, *J* = 7.0, 1.7 Hz, 1H, CH), 6.62 (dd, *J* = 8.2, 1.0 Hz, 1H, CH), 6.09 (s, 1H, CH), 5.08 (s, 1H, CH), 4.41 (ddd, *J* = 13.3, 8.6, 4.5 Hz, 1H, CH_2), 4.32 (qd, *J* = 7.0, 1.8 Hz, 2H, CH_2), 4.27-4.20 (m, 4H, CH_2), 4.13 (qd, *J* = 7.1, 3.1 Hz, 2H, CH_2), 3.74 (s, 3H, OCH_3), 3.71-3.66 (m, 1H, CH_2), 3.64 (s, 3H, OCH_3), 1.60-1.56 (m, 1H, CH_2), 1.41-1.37 (m, 1H, CH_2), 1.34-1.27 (m, 9H, CH_3), 1.18 (t, *J* = 7.2 Hz, 3H, CH_3), 0.53 (t, *J* = 7.4 Hz, 3H, CH_3) ppm. **^{13}C NMR (126 MHz, CD_2Cl_2):** δ = 169.60 (CO_2Et), 169.50 (CO_2Et), 169.05 (CO_2Et), 168.60 (CO_2Et), 158.99 (C), 158.20 (C), 150.65 (C), 145.50 (C), 145.12 (C), 140.85 (C), 133.10 (C), 132.08 (CH), 131.06 (CH), 130.27 (CH), 124.11 (C), 122.79 (C), 121.42 (CH), 118.40 (C), 115.72 (C), 111.83 (C), 111.55 (CH), 103.17 (CH), 102.05 (CH), 62.42 (CH_2), 62.39 (CH_2), 61.83 (2 x CH_2), 58.87 (CH_2), 55.70 (OCH_3), 55.40 (OCH_3), 54.70 (CH), 52.46 (CH), 21.64 (CH_2), 14.32 (3 x CH_3), 14.13 (CH_3), 10.98 (CH_3) ppm. **IR (neat, cm^{-1}):** ν = 2930, 2040, 1728, 1599, 1566, 1541, 1469, 1366, 1342, 1303, 1240, 1144, 1111, 1093, 1029, 977, 937, 859, 828, 783, 751, 659, 617, 586, 561, 546, 529. **HRMS (ESI)** calculated for [M+]: 687.2912, Found 687.2895.

Diisopropyl 2-(6-(1,3-dioxo-1,3-bis(2,2,2-trifluoroethoxy)propan-2-yl)-1,13-dimethoxy-5-propyl-5*H*-quinolino[2,3,4-*kl*]acridin-8-yl)malonate (4cg)

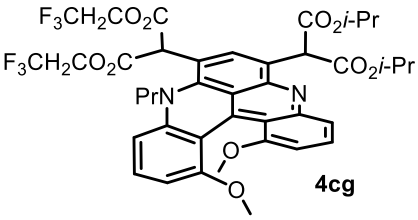

4cg

Compound **4cg** is prepared according to the general procedure, using 28 mg (0.05 mmol) of quinacridine **3c**, 1.95 mg (2.5 µmol) of $Rh_2(Oct)_4$ and 17 µL (0.10 mmol) of bis (2,2,2-trifluoroethyl) 2-diazomalonate in 0.1 mL of dry CH_2Cl_2. The desired compound was isolated as a dark red solid (16 mg, 40%).
***R*f** (SiO_2, CH_2Cl_2/MeOH, 99:1) = 0.55. **^{1}H NMR (500 MHz, CD_2Cl_2):** δ 7.79 (s, 1H, CH), 7.67-7.54 (m, 2H, 2 x CH), 7.46 (t, *J* = 8.3 Hz, 1H, CH), 7.08 (dd, *J* = 8.3, 1.0

Hz, 1H, CH), 6.71 (dd, J = 6.8, 1.8 Hz, 1H, CH), 6.64 (d, J = 8.1 Hz, 1H, CH), 6.04 (s, 1H, CH), 5.36 (s, 1H, CH), 5.16-5.04 (m, 2H, CH), 4.79-4.72 (m, 1H, CH_2), 4.68-4.59 (m, 2H, CH_2), 4.51-4.38 (m, 2H, CH_2), 3.75 (s, 3H, OCH_3), 3.64 (s, 3H, OCH_3), 3.62-3.56 (m, 1H, CH_2), 1.59-1.54 (m, 1H, CH_2), 1.45-1.40 (m, 1H, CH_2), 1.30-1.26 (m, 12H, CH_3), 0.50 (t, J = 7.3 Hz, 3H, CH_3) ppm. **^{13}C NMR (126 MHz, CD_2Cl_2):** δ = 168.88 (CO_2Me), 168.80 (CO_2Me), 167.19 ($CO_2CH_2CF_3$), 166.54 ($CO_2CH_2CF_3$), 158.98 (C), 158.18 (C), 150.83 (C), 145.32 (C), 145.06 (C), 141.62 (C), 133.15 (C), 131.20 (CH), 131.04 (CH), 130.54 (CH), 123.91 (C), 123.17 (q, J = 277.4 Hz, CF_3), 123.05 (q, J = 277.4 Hz, CF_3), 121.44 (C), 118.53 (C), 115.77 (C), 111.69 (C, CH), 109.44 (CH), 103.49 (CH), 102.21 (CH), 69.34 (CH), 69.29 (CH), 61.83 (q, J = 36.7 Hz, CH_2), 61.74 (q, J = 37.0 Hz, CH_2), 59.35 (CH_2), 55.71 (OCH_3), 55.43 (OCH_3), 53.35 (CH), 52.81(CH), 21.79 (3 x CH_3), 21.73 (CH_3), 21.57 (CH_2), 10.80 (CH_3) ppm. **^{19}F NMR (282 MHz, CD_2Cl_2):** δ = −74.04, −74.02 ppm. **IR (neat, cm^{-1}):** ν = 2984, 2925, 1730, 1600, 1570, 1540, 1469, 1414, 1375, 1275, 1163, 1100, 1059, 979, 905, 830, 787, 753, 663, 616, 583, 542. **HRMS (ESI)** calculated for [M+]: 823.2660, Found 823.2652.

Dimethyl 2-(6-(3-(2-fluoroacetoxy)-1-oxo-1-(2,2,2-trifluoroethoxy)-3l2-propan-2-yl)-1,13-dimethoxy-5-propyl-5H-quinolino[2,3,4-*kl*]acridin-8-yl)malonate (4ag)

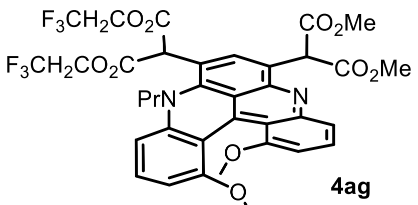

4ag

Compound **4ag** is prepared according to the general procedure, using 25 mg (0.05 mmol) of quinacridine **3a**, 1.95 mg (2.5 µmol) of $Rh_2(oct)_4$ and 17 µL (0.10 mmol) of bis (2,2,2-trifluoroethyl) 2-diazomalonate in 0.1 mL of dry CH_2Cl_2. The desired compound was isolated as a dark red solid (26 mg, 68%). ***R*f** (SiO_2, CH_2Cl_2/MeOH, 99:1) = 0.55. **^{1}H NMR (500 MHz, CD_2Cl_2):** δ 7.77 (s, 1H, CH), 7.68-7.60 (m, 2H, 2 x CH), 7.47 (t, J = 8.2 Hz, 1H, CH), 7.08 (dd, J = 8.4, 0.9 Hz, 1H, CH), 6.72 (dd, J = 5.9, 2.7 Hz, 1H, CH), 6.65 (dd, J = 8.1, 0.9 Hz, 1H, CH), 6.18 (s, 1H, CH), 5.35 (s, 1H, CH), 4.81-4.74 (m, 1H, CH_2), 4.70-4.63 (m, 1H, CH_2), 4.62-4.56 (m, 1H, CH_2), 4.50-4.42 (m, 2H, CH_2), 3.76 (s, 3H, OCH_3), 3.75 (s, 6H, OCH_3), 3.64 (s, 3H, OCH_3), 3.62-3.56 (m, 1H, CH_2), 1.61-1.57 (m, 1H, CH_2), 1.45-1.40 (m, 1H, CH_2), 0.51 (t, J = 7.4 Hz, 3H, CH_3) ppm. **^{13}C NMR (126 MHz, CD_2Cl_2):** δ = 169.94 (CO_2Me), 169.85 (CO_2Me), 167.15 ($CO_2CH_2CF_3$), 166.57 ($CO_2CH_2CF_3$), 158.96 (C), 158.19 (C), 150.83 (C), 145.09 (C), 144.96 (C), 141.81 (C), 133.32 (C), 131.55 (CH), 131.31 (CH), 130.71 (CH), 123.88 (C), 123.17 (q, J = 277.1 Hz, CF_3), 123.04 (q, J = 277.4 Hz, CF_3), 121.31 (C), 118.45 (C), 115.77 (C), 111.64 (C, CH), 109.40 (CH), 103.51 (CH), 102.26 (CH), 61.87 (q, J = 37.3 Hz, CH_2), 61.78 (q, J = 37.0 Hz, CH_2), 59.30 (CH_2), 55.72 (OCH_3), 55.43 (OCH_3), 53.52 (CH), 52.93 (2 x OCH_3), 51.80 (CH), 21.60 (CH_2), 10.79 (CH_3) ppm. **^{19}F NMR (282 MHz, CD_2Cl_2):** δ = −74.04, −74.02 ppm. **IR (neat, cm^{-1}):** ν = 2922, 2851, 1751, 1600, 1568, 1471, 1435, 1414, 1277, 1257, 1159, 1056, 978, 828, 784, 754, 664, 588, 554. **HRMS (ESI)** calculated for [M+]: 767.2034, Found 767.2060.

Synthetic procedure for one-pot tandem preparation of quinacridine **4aa**

$[CpRu(CH_3CN)_3][PF_6]$ (1.41 mg, 0.003 mmol) and 1,10-phenanthroline monohydrate (0.58 mg, 0.003 mmol) were dissolved in 0.2 mL dry CH_2Cl_2 (0.5 M) and the resulting mixture was allowed to stir for 15 min. Then, quinacridine **2** (40 mg, 0.1 mmol) was added and dimethyl 2-diazomalonate (25 µL, 0.2 mmol) **1a** at the end. The vial was sealed and allowed to stir at 60 °C for 3 h. After complete consumption of the starting quinacridine **2** (monitored by ESI-MS), the crude reaction mixture was allowed to cool to room temperature. Then $Rh_2(oct)_4$ (4.21 mg, 0.005 mmol) and dimethyl 2-diazomalonate (25 µL, 0.2 mmol) **1a** were added to the crude. The vial was sealed again and the reaction mixture was heated at 60 °C for 20 h (monitored by ESI-MS). After this time the solvent was removed under reduced pressure and the residue was purified by flash chromatography (Si_2O, CH_2Cl_2/MeOH gradient from 100:0 to 99:1) affording quinacridine **4aa** as a dark red solid (41 mg, 60 %).

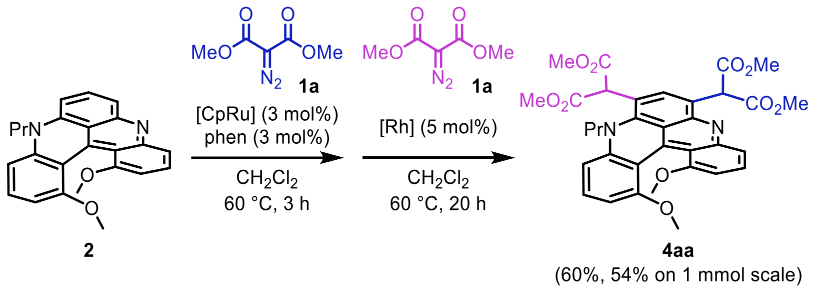

Figure S1. One-pot (tandem) double C–H insertions of diazomalonate **1a** onto helicene **2**.

Synthetic procedure for preparation of quinacridines 5 and 6

Dimethyl 2-diazomalonate (55 µL, 0.5 mmol) was added to a solution of quinacridine **4aa** (63 mg, 0.1 mmol) and $Rh_2(oct)_4$ (4 mg, 0.005 mmol) in 0.5 mL dry DCM (0.2 M) in a microwave vial. The vial was then sealed and the resulting mixture was heated to 60 °C and monitored by ESI-MS. Upon reaction completion (20 h), the solution was allowed to cool down to room temperature and then washed with 1M NaOH and deionized water and dried over anhydrous Na_2SO_4. The solvent was removed under reduced pressure to provide a dark red crude solid from which malonate derivatives **5** and **6** were separated by flash chromatography (silica gel, CH_2Cl_2/MeOH gradient from 100:0 to 99.5:0.5). The product **5** is obtained as a purple solid (15 mg, 20 %) and the product **6** as a red solid (9.8 mg, 11 %).

Hexamethyl 2,2',2''-(1,13-dimethoxy-5-propyl-5H-quinolino[2,3,4-*kl*]acridine-6,8,10-triyl)trimalonate (5)

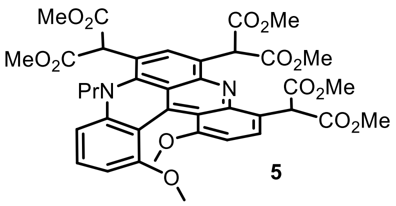

***R*f** (SiO_2, CH_2Cl_2/MeOH, 99:1) = 0.27. **^{1}H NMR (500 MHz, CD_2Cl_2):** δ 7.81 (s, 1H, CH), 7.62 (dd, *J* = 7.9, 0.6 Hz, 1H, CH), 7.46 (t, *J* = 8.2 Hz, 1H, CH), 7.07 (dd, *J* = 8.4, 0.9 Hz, 1H, CH), 6.70 (d, *J* = 8.0 Hz, 1H, CH), 6.63 (dd, *J* = 8.3, 0.9 Hz, 1H, CH), 6.09 (s, 1H, CH), 5.99 (s, 1H, CH), 5.12 (s, 1H, CH), 4.43 (ddd, *J* = 13.4, 8.6, 4.5 Hz, 1H, CH_2), 3.86 (s, 3H, OCH_3), 3.80 (s, 3H, OCH_3), 3.77-3.74 (m, 12H, CH_3), 3.67 (s, 3H, OCH_3), 3.66 (s, 3H, OCH_3), 3.59-3.63 (m, 1H, CH_2), 1.56-1.51 (m, 1H, CH_2), 1.42-1.36 (m, 1H, CH_2), 0.53 (t, *J* = 7.4 Hz, 3H, CH_3) ppm. **^{13}C NMR (126 MHz, CD_2Cl_2):** δ = 170.42 (CO_2Me), 170.29 (CO_2Me), 169.95 (CO_2Me), 169.80 (CO_2Me), 169.39 (CO_2Me), 169.01 (CO_2Me), 158.99 (C), 158.23 (C), 147.93 (C), 145.33 (C), 144.21 (C), 140.89 (C), 134.02 (C), 132.65 (CH), 131.46 (CH), 130.36 (CH), 123.92 (C), 123.22 (C), 122.68 (C), 118.10 (C), 115.39 (C), 111.73 (C), 111.50 (CH), 103.21 (CH), 101.35 (CH), 58.82 (CH_2), 55.68 (OCH_3), 55.46 (OCH_3), 54.33 (CH), 53.38 (OCH_3), 53.28 (OCH_3), 52.96 (OCH_3), 52.91 (2 x OCH_3), 52.88 (OCH_3), 52.82 (CH), 52.24 (CH), 21.65 (CH_2), 10.96 (CH_3) ppm. **IR (neat, cm^{-1}):** ν = 2922, 2851, 1731, 1600, 1572, 1523, 1470, 1434, 1241, 1196, 1144, 1114, 1095, 1053, 1025, 929, 827, 785, 751, 665, 586, 529, 515, 500. **HRMS (ESI)** calculated for [M+]: 761.2553, Found 761.2572.

Octamethyl 2,2',2'',2'''-(1,13-dimethoxy-5-propyl-5H-quinolino[2,3,4-*kl*]acridine-4,6,8,10-tetrayl)tetra-malonate (6)

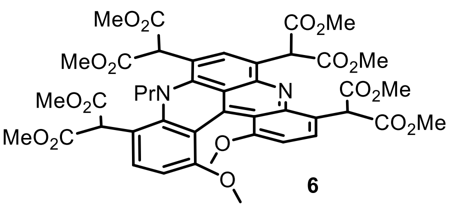

***R*f** = 0.30 (SiO_2, CH_2Cl_2/MeOH, 99:1). **^{1}H NMR (500 MHz, CD_2Cl_2):** δ 7.87 (s, 1H, CH), 7.68 (d, *J* = 8.0 Hz, 1H, CH), 7.61 (d, *J* = 8.8 Hz, 1H, CH), 6.84 (d, *J* = 8.9 Hz, 1H, CH), 6.77 (d, *J* = 8.0 Hz, 1H, CH), 6.12 (s, 1H, CH), 6.05 (s, 1H, CH), 5.53 (s, 1H, CH), 5.51 (s, 1H, CH), 3.89 (s, 3H, OCH_3), 3.84 (s, 3H, OCH_3), 3.82 (s, 3H, OCH_3), 3.81 (s, 3H, OCH_3), 3.78 (s, 3H, OCH_3), 3.76 (s, 3H, OCH_3), 3.73 (s, 6H, 2 x OCH_3), 3.67 (s, 3H, OCH_3), 3.65 (s, 3H, OCH_3), 3.59-3.55 (m, 1H, CH_2), 3.42 (ddd, *J* = 13.8, 11.3, 5.1 Hz, 1H, CH_2), 1.18-1.13 (m, 1H, CH_2), 0.70-0.63 (m, 1H, CH_2),

0.44 (t, J = 7.3 Hz, 3H, CH_3) ppm. **^{13}C NMR (126 MHz, CD_2Cl_2):** δ = 170.30 (CO_2Me), 170.15 (CO_2Me), 169.68 (2 x CO_2Me), 169.47 (CO_2Me), 169.32 (CO_2Me), 169.23 (CO_2Me), 168.62 (CO_2Me), 158.24 (C), 157.66 (C), 148.15 (C), 145.63 (C), 144.14 (C), 141.22 (C), 134.96 (C), 133.16 (CH), 131.54 (CH), 130.65 (CH), 126.15 (C), 125.75 (C), 123.45 (C), 123.19 (C), 119.96 (C), 118.58 (C), 116.60 (C), 107.54 (CH), 101.89 (CH), 64.08 (CH_2), 55.83 (OCH_3), 55.65 (OCH_3), 53.35 (2 x OCH_3), 53.27 (OCH_3), 53.18 (OCH_3), 53.02 (OCH_3), 52.99 (OCH_3), 52.97 (2 x OCH_3), 52.72 (CH), 52.63 (CH), 52.28 (CH), 52.00 (CH), 19.22 (CH_2), 11.06 (CH_3) ppm. **IR (neat, cm^{-1}):** ν = 3750, 2955, 2921, 2851, 1732, 1573, 1539, 1458, 1435, 1233, 1196, 1146, 1112, 1095, 1057, 1026, 988, 924, 829, 797, 750, 666, 593, 567, 547, 521. **HRMS (ESI)** calculated for [M+]: 891.2819, Found 891.2757.

General procedure for synthesis of mono 12 and bis amides 13

Quinacridine **3a** or **4aa** (0.06 mmol) was added to the corresponding primary amine (100 equiv) and the resulting mixture was heated at 90 °C in an open to air vial. After complete consumption of the starting quinacridine (monitored by ESI-MS), the crude reaction mixture was allowed to cool down to room temperature. Then it was sequentially washed with saturated solutions of citric acid, $NaHCO_3$ and finally with water. The residue was purified by flash chromatography (Si_2O, CH_2Cl_2/MeOH gradient from 100:0 to 91:9) affording the titled quinacridines **12** or **13** as dark purple solids.

2-(1,13-dimethoxy-5-propyl-5*H*-quinolino[2,3,4-*kl*]acridin-8-yl)-*N*^{1},*N*^{3}-dihexylmalonamide (12a)

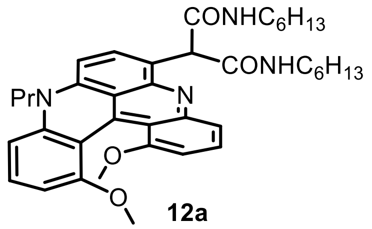

12a

Compound **12a** is prepared according to the general procedure, using 30 mg (0.06 mmol) of quinacridine **3a** and 0.8 mL of *n*-hexylamine. The reaction mixture was heated for 4 h. The desired compound was isolated as a dark purple solid (31 mg, 81%).

^{1}H NMR (500 MHz, CD_2Cl_2): δ = 8.64 (t, J = 5.7 Hz, 2H, NH_2), 7.69 (d, J = 8.1 Hz, 1H, CH), 7.64-7.55 (m, 2H, 2 x CH), 7.50 (t, J = 8.3 Hz, 1H, CH), 6.94 (d, J = 8.6 Hz, 1H, CH), 6.68 (d, J = 8.2 Hz, 1H, CH), 6.64 (dd, J = 6.7, 1.8 Hz, 1H, CH), 6.64 (d, J = 8.1 Hz, 1H, CH), 5.61 (s, 1H, CH), 4.22 (ddd, J = 16.2, 11.7, 5.3 Hz, 1H, CH_2), 3.91 (ddd, J = 15.9, 11.4, 5.0 Hz, 1H, CH_2), 3.74 (s, 3H, OCH_3), 3.68 (s, 3H, OCH_3), 3.29-3.22 (m, 2H, CH_2), 3.21-3.12 (m, 2H, CH_2), 2.09-2.00 (m, 1H, CH_2), 1.99-1.90 (m, 1H, CH_2), 1.49-1.40 (m, 4H, 2 x CH_2), 1.22-1.12 (m, 15H, 6 x CH_2, CH_3), 0.84-0.76 (m, 6H, 2 x CH_3) ppm. **^{13}C NMR (126 MHz, CD_2Cl_2):** δ = 171.27 (CO), 171.08 (CO), 159.92 (C), 158.47 (C), 150.84 (C), 146.41 (C), 143.15 (C), 138.70 (C), 134.85 (C), 132.47 (CH), 130.82 (CH), 129.14 (CH), 121.74 (C), 120.91 (C), 120.52 (CH), 114.90 (C), 112.61 (C), 106.28 (CH), 101.90 (CH), 100.89 (CH), 99.44 (CH), 55.42 (OCH_3), 55.36 (OCH_3), 51.33 (CH), 50.12 (CH_2), 39.66 (2 x CH_2), 31.87 (2 x CH_2), 29.82 (CH_2), 29.78 (CH_2), 27.03 (2 x CH_2), 22.90 (2 x CH_2), 18.96 (CH_2), 14.16 (CH_3), 14.13 (CH_3), 11.31 (CH_3) ppm. **IR (neat, cm^{-1}):** ν = 3301, 2956, 2928, 2855, 1673, 1599, 1573, 1525, 1485, 1463, 1389, 1251, 1161, 1112, 1071, 1011, 825, 744. **HRMS (ESI)** calculated for [M+]: 639.3906, Found 639.3932.

2-(1,13-dimethoxy-5-propyl-5*H*-quinolino[2,3,4-*kl*]acridin-8-yl)-*N*^{1},*N*^{3}-dioctylmalonamide (12b)

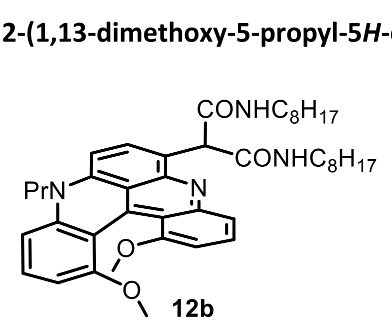

12b

Compound **12b** is prepared according to the general procedure, using 30 mg (0.06 mmol) of quinacridine **3a** and 1 mL of *n*-octylamine. The reaction mixture was heated for 4 h. The desired compound was isolated as a dark purple solid (31 mg, 74%).

^{1}H NMR (500 MHz, CD_2Cl_2): δ = 8.63 (t, J = 6.3 Hz, 2H, NH_2), 7.69 (d, J = 8.1 Hz, 1H, CH), 7.64-7.56 (m, 2H, 2 x CH), 7.51 (t, J = 8.3 Hz, 1H, CH), 6.94 (d, J = 8.6 Hz, 1H, CH), 6.68 (d, J = 8.1 Hz, 1H, CH), 6.64 (dd, J = 6.8, 1.8 Hz, 1H, CH), 6.62 (d, J = 8.0 Hz, 1H, CH), 5.61 (s, 1H, CH), 4.28-4.16 (m, 1H, CH_2), 3.96-3.86 (m, 1H, CH_2), 3.74 (s, 3H, OCH_3), 3.68 (s,

3H, OCH_3), 3.31-3.21 (m, 2H, CH_2), 3.20-3.13 (m, 2H, CH_2), 2.10-1.99 (m, 1H, CH_2), 1.98-1.89 (m, 1H, CH_2), 1.49-1.40 (m, 4H, 2 x CH_2), 1.23-1.11 (m, 23H, 10 x CH_2, CH_3), 0.88-0.82 (m, 6H, 2 x CH_3) ppm. **^{13}C NMR (126 MHz, CD_2Cl_2):** δ = 171.24 (CO), 171.08 (CO), 159.94 (C), 158.47 (C), 150.82 (C), 146.41 (C), 143.15 (C), 138.69 (C), 134.87 (C), 132.48 (CH), 130.83 (CH), 129.15 (CH), 121.74 (C), 120.90 (C), 120.50 (CH), 114.89 (C), 112.62 (C), 106.28 (CH), 101.92 (CH), 100.90 (CH), 99.44 (CH), 55.41 (OCH_3), 55.37 (OCH_3), 51.31 (CH), 50.14 (CH_2), 39.65 (2 x CH_2), 32.19 (CH_2), 30.09 (CH_2), 29.87 (CH_2), 29.81 (CH_2), 29.63 (3 x CH_2), 29.57 (CH_2), 27.37 (2 x CH_2), 23.04 (CH_2), 23.01 (CH_2), 18.97 (CH_2), 14.25 (2 x CH_3), 11.32 (CH_3) ppm. **IR (neat, cm^{-1}):** ν = 3291, 2956, 2923, 2853, 1675, 1599, 1573, 1526, 1483, 1462, 1346, 1256, 1161, 1113, 1071, 825, 743. **HRMS (ESI)** calculated for [M+]: 695.4531, Found 695.4526.

2-(1,13-dimethoxy-5-propyl-5*H*-quinolino[2,3,4-*kl*]acridin-8-yl)-*N*^{1},*N*^{3}-didodecylmalonamide (12c)

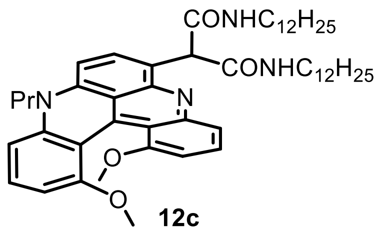

12c

Compound **12c** is prepared according to the general procedure, using 30 mg (0.06 mmol) of quinacridine **3a** and 1.1 g of *n*-dodecylamine. The reaction mixture was heated for 7 h. The desired compound was isolated as a dark purple solid (39 mg, 80%).

^{1}H NMR (500 MHz, CD_2Cl_2): δ = 8.63 (br s, 2H, NH_2), 7.69 (d, *J* = 8.1 Hz, 1H, CH), 7.64-7.55 (m, 2H, 2 x CH), 7.50 (t, *J* = 8.4 Hz, 1H, CH), 6.94 (d, *J* = 8.6 Hz, 1H, CH), 6.68 (d, *J* = 8.1 Hz, 1H, CH), 6.65-6.53 (m, 2H, 2 x CH), 5.61 (s, 1H, CH), 4.30-4.15 (m, 1H, CH_2), 3.97-3.86 (m, 1H, CH_2), 3.74 (s, 3H, OCH_3), 3.68 (s, 3H, OCH_3), 3.32-3.21 (m, 2H, CH_2), 3.20-3.11 (m, 2H, CH_2), 2.10-2.00 (m, 1H, CH_2), 1.99-1.89 (m, 1H, CH_2), 1.49-1.40 (m, 4H, 2 x CH_2), 1.25-1.12 (m, 39H, 18 x CH_2, CH_3), 0.88 (t, *J* = 7.0 Hz, 6H, 2 x CH_3) ppm. **^{13}C NMR (126 MHz, CD_2Cl_2):** δ = 171.24 (CO), 171.09 (CO), 159.94 (C), 158.47 (C), 150.82 (C), 146.40 (C), 143.15 (C), 138.69 (C), 134.88 (C), 132.49 (CH), 130.83 (CH), 129.15 (CH), 121.74 (C), 120.91 (C), 120.50 (CH), 114.89 (C), 112.63 (C), 106.29 (CH), 101.92 (CH), 100.89 (CH), 99.44 (CH), 55.41 (OCH_3), 55.38 (OCH_3), 51.32 (CH), 50.14 (CH_2), 39.66 (2 x CH_2), 32.34 (CH_2), 30.08 (CH_2), 30.05 (2 x CH_2), 30.01 (2 x CH_2), 29.93 (2 x CH_2), 29.88 (2 x CH_2), 29.82 (2 x CH_2), 29.78 (2 x CH_2), 29.69 (2 x CH_2), 27.38 (CH_2), 27.36 (CH_2), 23.11 (2 x CH_2), 18.99 (CH_2), 14.29 (2 x CH_3), 11.32 (CH_3) ppm. **IR (neat, cm^{-1}):** ν = 3289, 2956, 2922, 2852, 1676, 1599, 1574, 1537, 1526, 1463, 1347, 1257, 1162, 1113, 1072, 825, 743, 725. **HRMS (ESI)** calculated for [M+]: 807.5784, Found 807.5759.

2-(1,13-dimethoxy-5-propyl-5*H*-quinolino[2,3,4-*kl*]acridin-8-yl)-*N*^{1},*N*^{3}-dihexadecylmalonamide (12d)

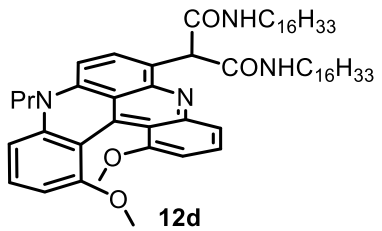

12d

Compound **12d** is prepared according to the general procedure, using 30 mg (0.06 mmol) of quinacridine **3a** and 1.45 g of *n*-hexadecylamine. The reaction mixture was heated for 7 h. The desired compound was isolated as a dark purple solid (44 mg, 80%).

^{1}H NMR (500 MHz, CD_2Cl_2): δ = 8.71-8.57 (m, 2H, NH_2), 7.69 (d, *J* = 8.1 Hz, 1H, CH), 7.64-7.55 (m, 2H, 2 x CH), 7.50 (t, *J* = 8.3 Hz, 1H, CH), 6.94 (d, *J* = 8.6 Hz, 1H, CH), 6.68 (d, *J* = 8.2 Hz, 1H, CH), 6.65-6.59 (m, 2H, 2 x CH), 5.60 (s, 1H, CH), 4.22 (ddd, *J* = 16.1, 11.5, 5.2 Hz, 1H, CH_2), 3.97-3.87 (m, 1H, CH_2), 3.74 (s, 3H, OCH_3), 3.68 (s, 3H, OCH_3), 3.26 (ddd, *J* = 18.3, 12.6, 6.3 Hz, 2H, CH_2), 3.20-3.13 (m, 2H, CH_2), 2.12-2.01 (m, 1H, CH_2), 1.99-1.88 (m, 1H, CH_2), 1.48-1.41 (m, 4H, 2 x CH_2), 1.27-1.12 (m, 55H, 26 x CH_2, CH_3), 0.88 (t, *J* = 6.8 Hz, 6H, 2 x CH_3) ppm. **^{13}C NMR (126 MHz, CD_2Cl_2):** δ = 171.24 (CO), 171.09 (CO), 159.94 (C), 158.46 (C), 150.83 (C), 146.42 (C), 143.15 (C), 138.70 (C), 134.86 (C), 132.48 (CH), 130.82 (CH), 129.15 (CH), 121.75 (C), 120.92 (C), 120.51 (CH), 114.90 (C), 112.63 (C), 106.28 (CH), 101.92 (CH), 100.88 (CH), 99.43 (CH), 55.40 (OCH_3), 55.37 (OCH_3), 51.32 (CH), 50.13 (CH_2), 39.65 (2 x CH_2), 32.35 (CH_2), 30.12 (8 x CH_2), 30.08 (2 x CH_2), 30.05 (CH_2), 30.02 (2 x CH_2), 29.94 (2 x CH_2), 29.88 (2 x CH_2), 29.82 (2 x CH_2), 29.78 (2 x CH_2), 29.70 (2 x CH_2), 27.38 (CH_2), 27.36 (CH_2), 23.11 (2 x CH_2), 18.98 (CH_2), 14.29 (2 x CH_3), 11.33 (CH_3) ppm. **IR (neat, cm^{-1}):** ν = 3289, 2956, 2921, 2852, 1677, 1599, 1575, 1538, 1464, 1377, 1257, 1162, 1113, 1073, 825, 743, 723. **HRMS (ESI)** calculated for [M+]: 919.7035, Found 919.7049.

2,2'-(1,13-dimethoxy-5-propyl-5*H*-quinolino[2,3,4-*kl*]acridine-6,8-diyl)bis(N^1,N^3-dihexylmalonamide) (13)

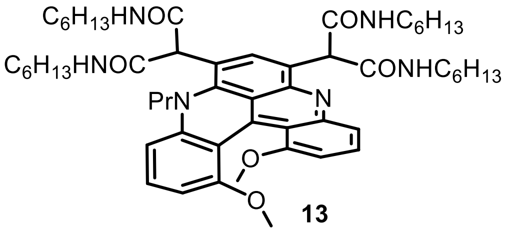

13

Compound **13** is prepared according to the general procedure, using 38 mg (0.06 mmol) of quinacridine **4aa** and 0.8 mL of *n*-hexylamine. The reaction mixture was heated for 4 h. The desired compound was isolated as a dark purple solid (43 mg, 79%).

^{1}H NMR (500 MHz, CD_2Cl_2): δ = 8.49-8.40 (m, 2H, NH_2), 7.67-7.63 (m, 2H, 2 x CH), 7.61-7.54 (m, 2H, CH, NH_2), 7.44 (t, *J* = 8.2 Hz, 1H, CH), 7.04 (d, *J* = 8.3 Hz, 1H, CH), 6.74-6.70 (m, 1H, CH), 6.62 (d, *J* = 8.1 Hz, 1H, CH), 6.41 (s, 1H, NH_2), 5.68 (s, 1H, CH), 4.81 (s, 1H, CH), 4.39 (ddd, *J* = 13.3, 8.4, 4.3 Hz, 1H, CH_2), 3.91-3.80 (m, 1H, CH_2), 3.75 (s, 3H, OCH_3), 3.65 (s, 3H, OCH_3), 3.34 (ddd, *J* = 13.3, 7.6, 6.0 Hz, 1H, CH_2), 3.28-3.20 (m, 4H, 2 x CH_2), 3.19-3.15 (m, 1H, CH_2), 3.11 (dd, *J* = 14.0, 6.9 Hz, 1H, CH_2), 3.06-2.98 (m, 1H, CH_2), 1.56-1.42 (m, 8H, 4 x CH_2), 1.34-1.30 (m, 6H, 3 x CH_2), 1.21-1.09 (m, 20H, 10 x CH_2), 0.94-0.85 (m, 6H, 2 x CH_3), 0.81-0.79 (m, 6H, 2 x CH_3), 0.51 (t, *J* = 7.3 Hz, 3H, CH_3) ppm. **^{13}C NMR (126 MHz, CD_2Cl_2):** δ = 170.63 (2 x CO), 169.34 (CO), 169.13 (CO), 159.03 (C), 158.35 (C), 150.52 (C), 145.41 (C), 145.08 (C), 140.99 (C), 133.99 (C), 131.66 (CH), 131.48 (CH), 130.86 (CH), 125.64 (C), 124.71 (C), 120.74 (CH), 118.23 (C), 115.75 (C), 114.04 (C), 111.49 (CH), 103.11 (CH), 102.16 (CH), 58.18 (CH_2), 55.71 (CH, OCH_3), 55.36 (OCH_3), 51.70 (CH), 40.47 (CH_2), 40.39 (CH_2), 39.88 (2 x CH_2), 31.92 (CH_2), 31.89 (CH_2), 31.75 (CH_2), 30.10 (CH_2), 29.86 (CH_2), 29.78 (CH_2), 29.73 (CH_2), 29.49 (CH_2), 29.07 (3 x CH_2), 26.84 (CH_2), 22.96 (CH_2), 22.90 (2 x CH_2), 22.84 (CH_2), 21.71 (CH_2), 14.21 (CH_3), 14.17 (2 x CH_3), 14.11 (CH_3), 10.98 (CH_3) ppm. **IR (neat, cm^{-1}):** ν = 3295, 2956, 2927, 2857, 1671, 1599, 1562, 1536, 1521, 1467, 1436, 1337, 1255, 1148, 1114, 1051, 827, 750, 727. **HRMS (ESI)** calculated for [M+]: 907.6056, Found 907.6032.

Chiral Stationary Phase (CSP) HPLC resolution

Resolution and specific rotation of compound **2**

Conditions:

Columns: CHIRALPAK IG analytic and semi-preparative.

Mobile phase: MeOH/EtOH (50:50) + 0.1% DEA (diethylamine)

Elution: 1 mL/min, 25 °C (for semi-preparative) or 1 mL/min, 25 °C (for analytical).

Due to the presence of DEA in the mobile phase, the separated enantiomer solutions were evaporated, dissolved in CH_2Cl_2 and washed three times with 1 M aq HBF_4 and once with 1.5 M aq NaOH. The organic layer was next dried over Na_2SO_4, filtered and evaporated.

Absolute configurations were previously determined for compounds of type **2** by VCD.[19]

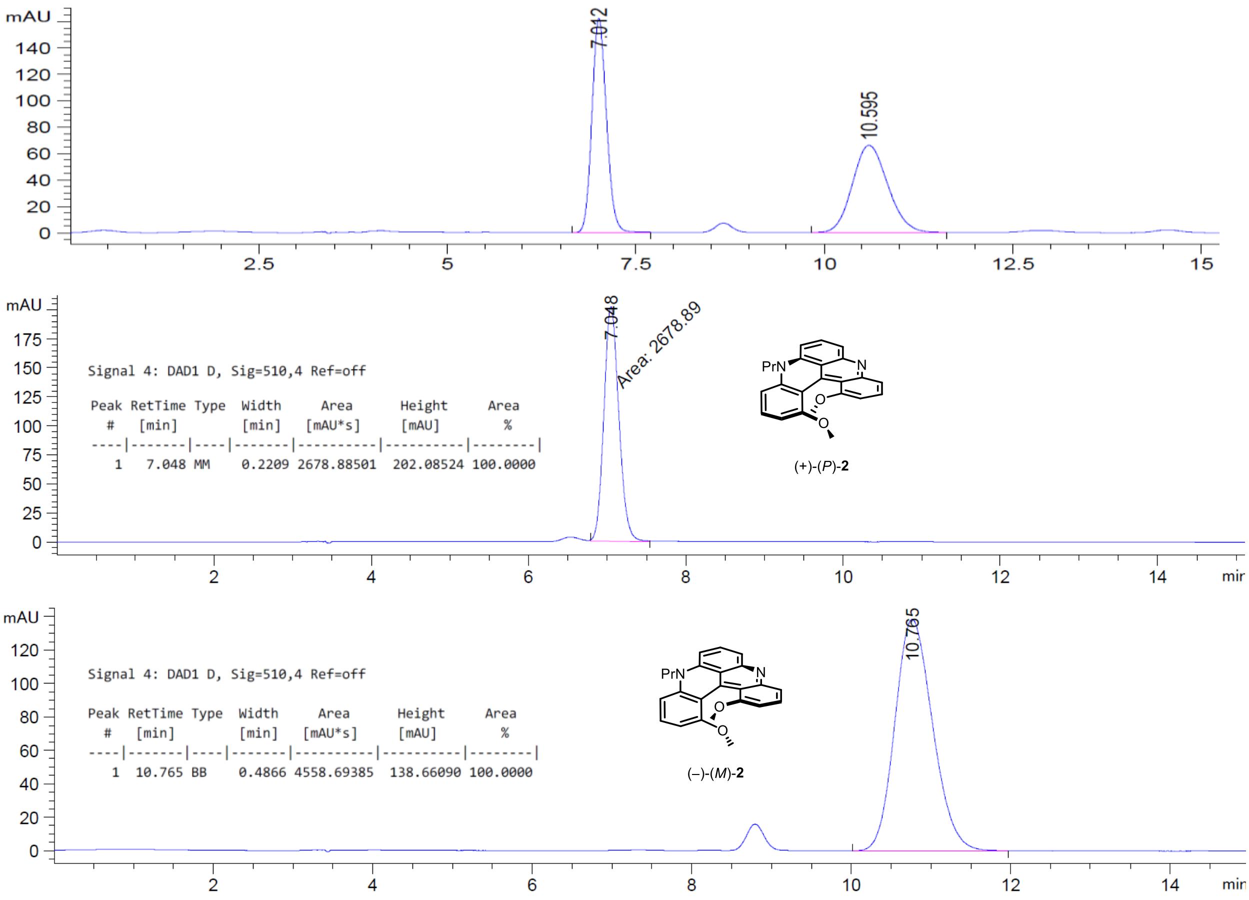

Figure S2. HPLC chromatograms of (+)-(*P*)-**2** and (–)-(*M*)-**2** recorded after HPLC resolution of *rac*-**2**.

Specific rotations of the enantiomers of **2**.

First eluted: $[\alpha]_{365}$ +11100 (in CH_3CN)

Second eluted: $[\alpha]_{365}$ −10800 (in CH_3CN)

Resolution and specific rotation of compound **3a**

Conditions:

Columns: CHIRALPAK IG analytic and semi-preparative.

Mobile phase: MeOH/EtOH (50:50) + 0.1% DEA

Elution: 2 mL/min, 25 °C (for semi-preparative) or 1 mL/min, 25 °C (for analytical).

Due to the presence of DEA in the mobile phase, the separated enantiomer solutions were evaporated, dissolved in CH_2Cl_2 and washed three times with 1 M aq HBF_4 and once with 1.5 M aq NaOH. The organic layer was next dried over Na_2SO_4, filtered and evaporated.

Absolute configurations are determined by comparison between the ECD spectra of **3a** with that of (+)-(*P*) and (−)-(*M*)-**2** (*vide infra*).

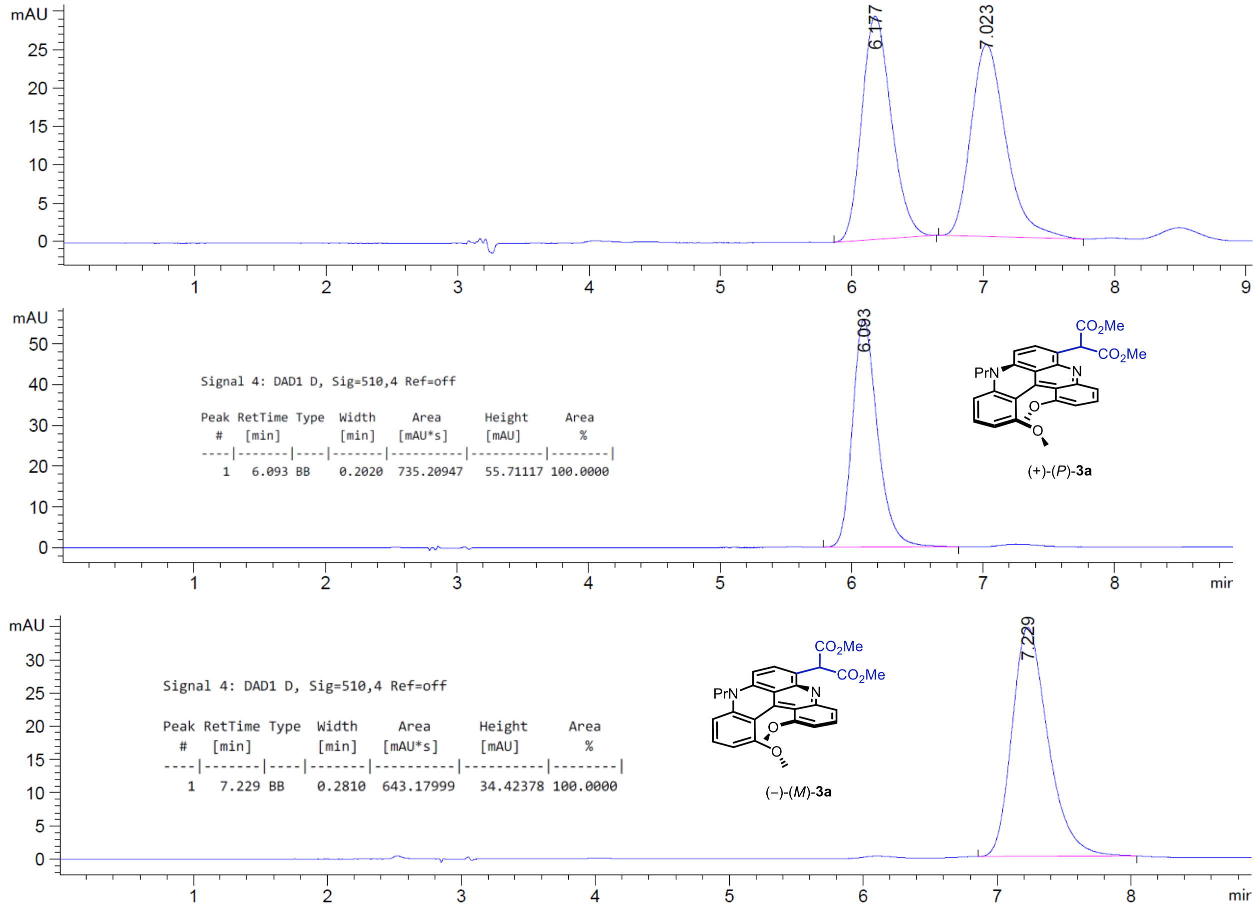

Figure S3. HPLC chromatograms of (+)-(*P*)-**3a** and (−)-(*M*)-**3a** recorded after HPLC resolution of *rac*-**3a**.

*Specific rotations of the enantiomers of **3a**.*

First eluted: $[\alpha]_{365}$ +9000 (in CH_3CN)

Second eluted: $[\alpha]_{365}$ −8000 (in CH_3CN)

Resolution and specific rotation of compound **4aa**

Conditions:

Columns: CHIRALPAK IH analytic and semi-preparative.

Mobile phase: MeOH/EtOH (25:75)

Elution: 1 mL/min, 25 °C (for semi-preparative) or 0.5 mL/min, 25 °C (for analytical).

Absolute configurations are determined by comparison between the ECD spectra of **4aa** with that of (+)-(*P*) and (−)-(*M*)-**2** (*vide infra*).

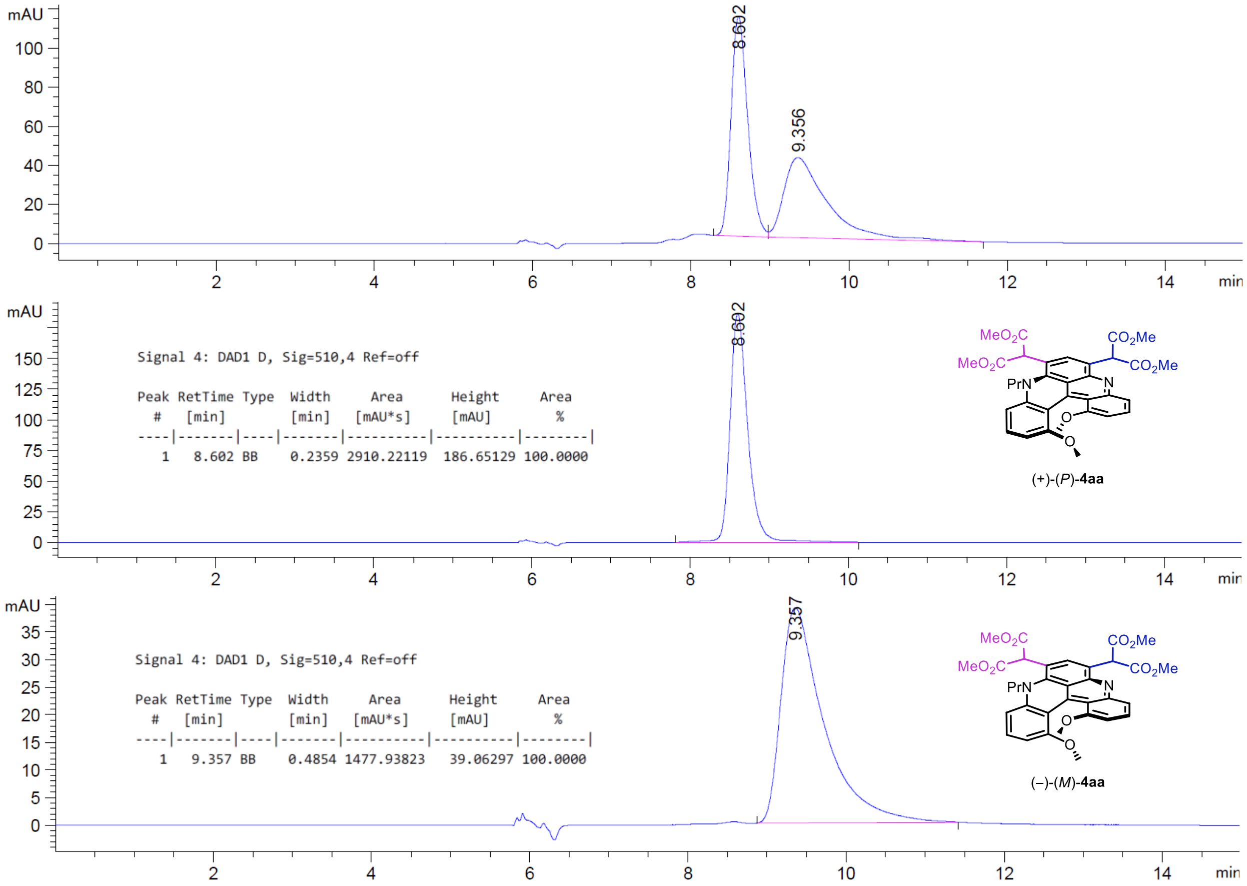

Figure S4. HPLC chromatograms of (+)-(*P*)-**4aa** and (−)-(*M*)-**4aa** recorded after HPLC resolution of *rac*-**4aa**.

*Specific rotations of the enantiomers of **4aa**.*

First eluted: $[\alpha]_{365}$ +5100 (in CH_3CN)

Second eluted: $[\alpha]_{365}$ −4900 (in CH_3CN)

Resolution and specific rotation of compound **5**

Conditions:

Columns: CHIRALPAK IG analytic and semi-preparative.

Mobile phase: MeOH/EtOH (50:50)

Elution: 2 mL/min, 25 °C (for semi-preparative) or 0.5 mL/min, 25 °C (for analytical).

Absolute configurations are determined by comparison between the ECD spectra of **5** with that of (+)-(*P*) and (−)-(*M*)-**2** (*vide infra*).

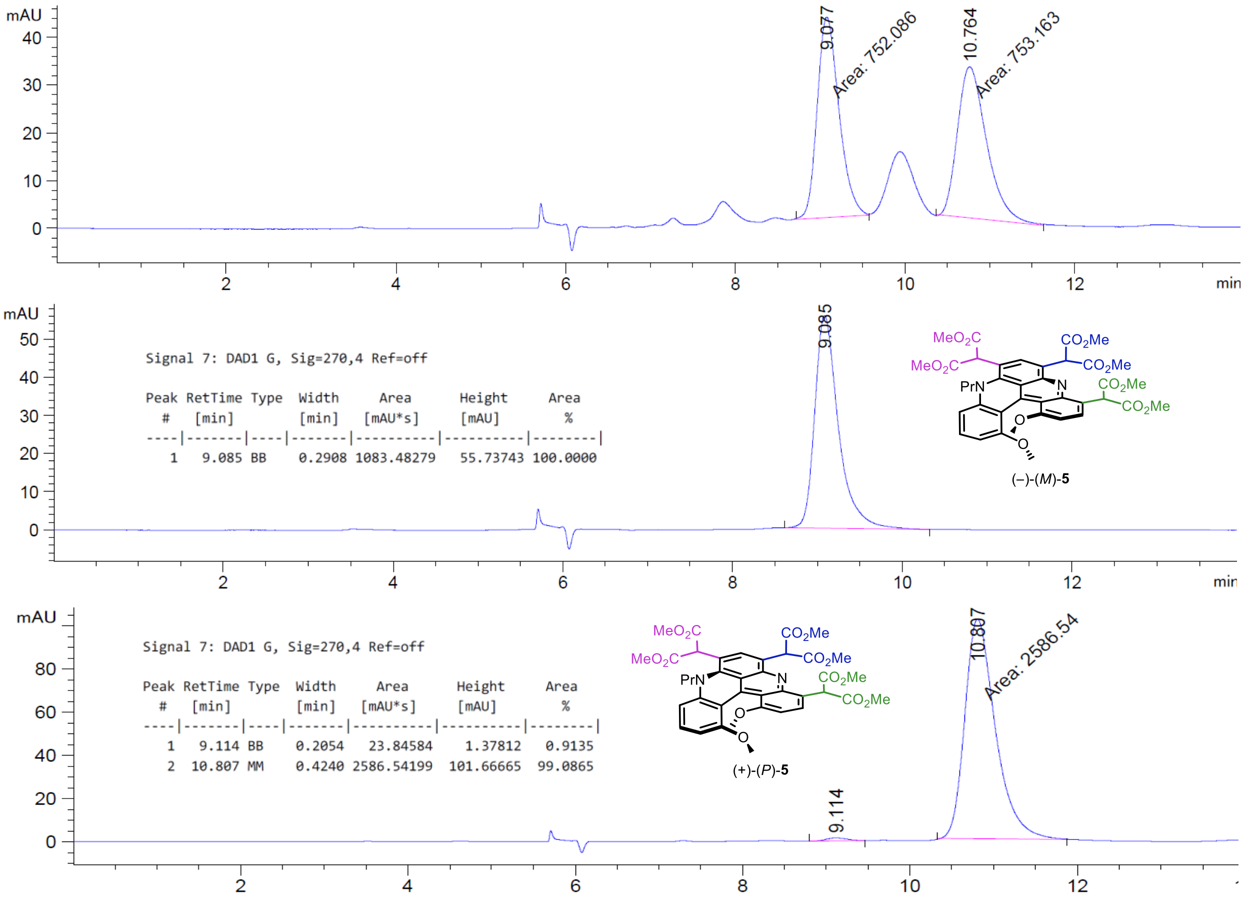

Figure S5. HPLC chromatograms of (+)-(*P*)-**5** and (–)-(*M*)-**5** recorded after HPLC resolution of *rac*-**5**.

*Specific rotations of the enantiomers of **5**.*

First eluted: $[\alpha]_{365}$ −4200 (in CH_3CN)

Second eluted: $[\alpha]_{365}$ +3900 (in CH_3CN)

Resolution and specific rotation of compound **6**

Conditions:

Columns: CHIRALPAK IG analytic and semi-preparative.

Mobile phase: MeOH/EtOH (50:50)

Elution: 2 mL/min, 25 °C (for semi-preparative) or 0.8 mL/min, 25 °C (for analytical).

Absolute configurations are determined by comparison between the ECD spectra of **6** with that of (+)-(*P*) and (−)-(*M*)-**2** (*vide infra*).

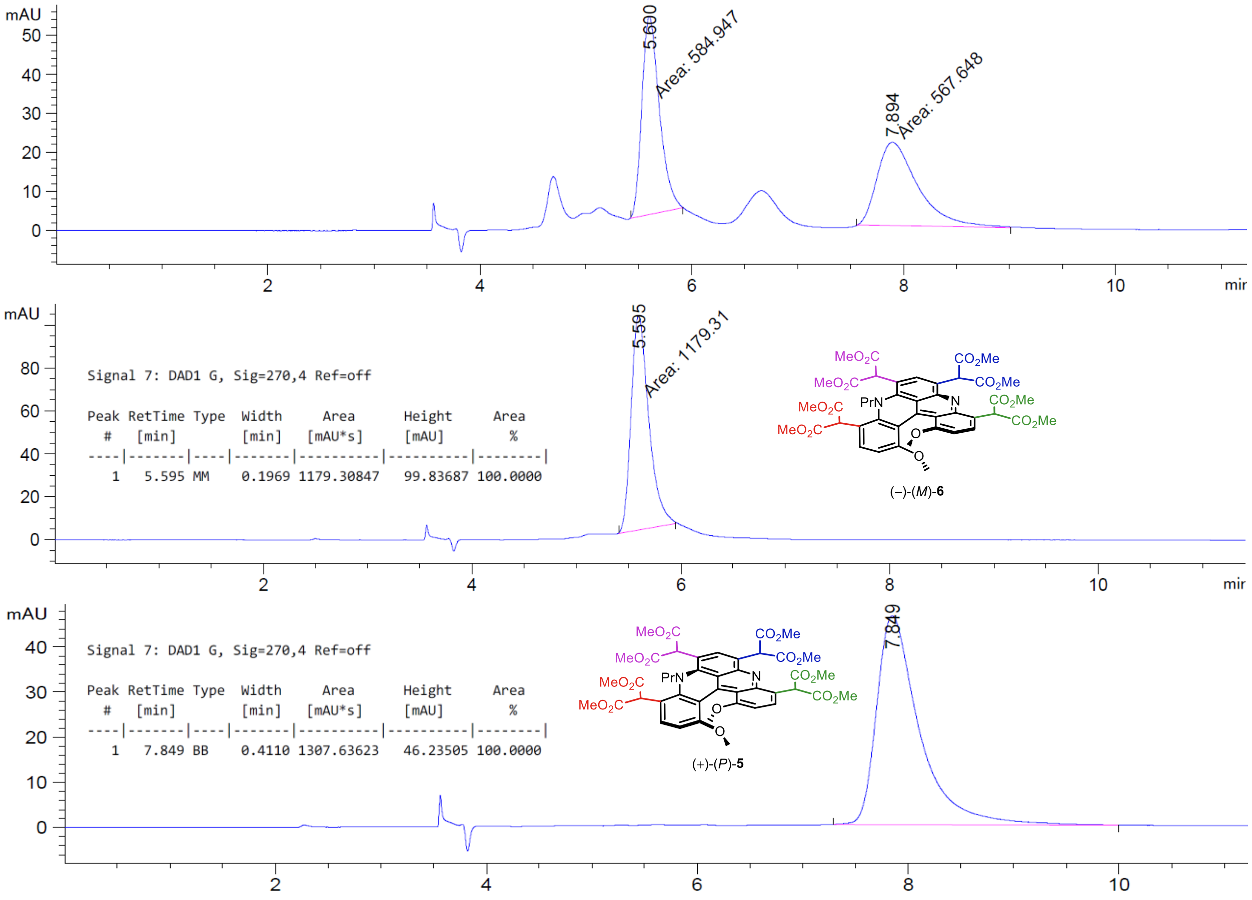

Figure S6. HPLC chromatograms of (+)-(*P*)-**6** and (–)-(*M*)-**6** recorded after HPLC resolution of *rac*-**6**.

Specific rotations of the enantiomers of ***6****.*

First eluted: $[\alpha]_{365}$ −1800 (in CH_3CN)

Second eluted: $[\alpha]_{365}$ +1700 (in CH_3CN)

Electrochemical properties

General Figure

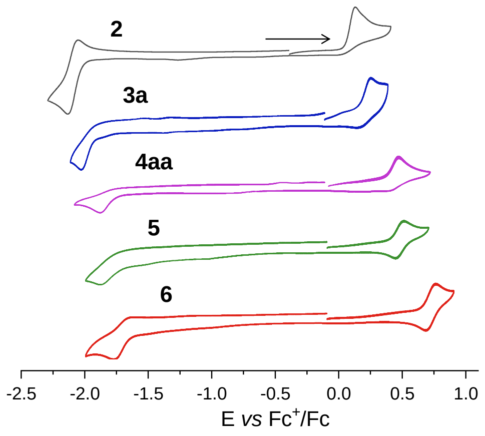

Figure S7. Cyclic voltammograms (potential values in V) for [4]helicenes **2** to **6** (5 x 10^{-4} M) in acetonitrile ([TBA][PF_6] 10^{-1} M) at a Pt electrode (Ø = 3 mm, v = 0.1 V/s).

Electrochemical data of compounds 2, 3a, 4aa, 5, 6

Table S1. Anodic and cathodic peak potentials (Ep_a, Ep_c, in V), half-wave potentials ($E_{1/2}$, in V) and electronic energy gap values (in eV) measured by CV for [4]helicenes (5x10^{-4} M) in acetonitrile ([TBA][PF_6] 10^{-1} M) at a Pt electrode (Ø = 3 mm, v = 0.1 V/s), E *vs* Fc^+/Fc. Only first oxidation and first reduction data are reported.

	Oxidation			Reduction			Electronic energy gap
Compound	Ep_a	Ep_c	$E_{1/2}$	Ep_c	Ep_a	$E_{1/2}$	
**2**	+0.13	-	-	-2.14	-2.05	-2.09	2.22
**3a**	+0.25	-	-	-2.03	-	-	2.28
**4aa**	+0.47	-	-	-1.88	-	-	2.35
**5**	+0.52	+0.45	+0.48	-1.87	-	-	2.36
**6**	+0.76	+0.68	+0.72	-1.77	-	-	2.50

Optical and chiroptical properties

Absorption, emission and ECD spectra of compounds 2 and 2•H⁺

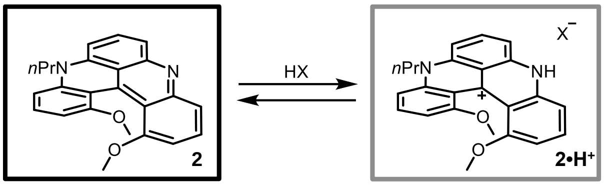

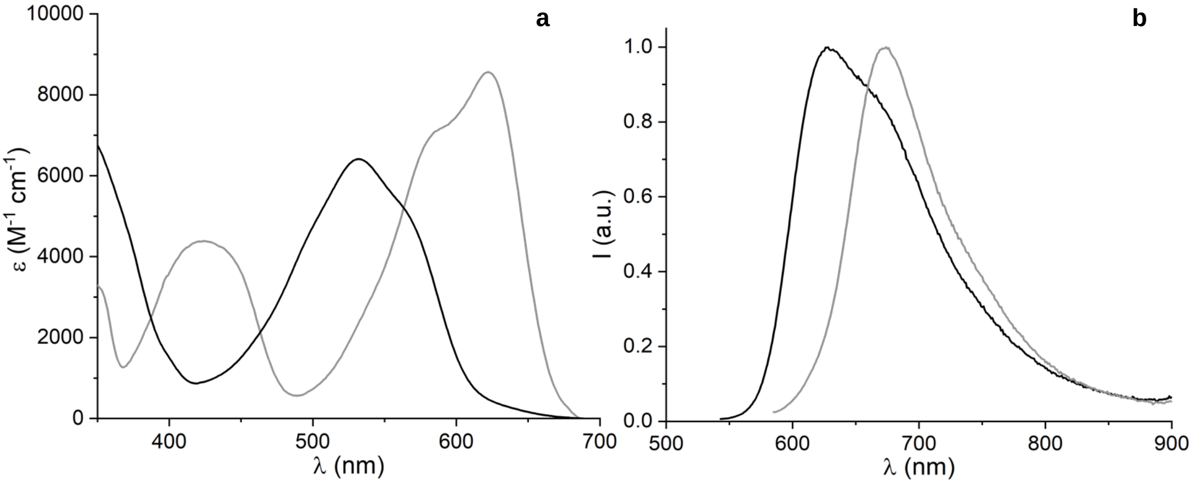

Figure S8. a) Absorption and b) normalized emission spectra of compound **2** (black) and **2•H⁺** (grey) in air-equilibrated acetonitrile solution (C *ca*. 10^{-5} M) at 293 K.

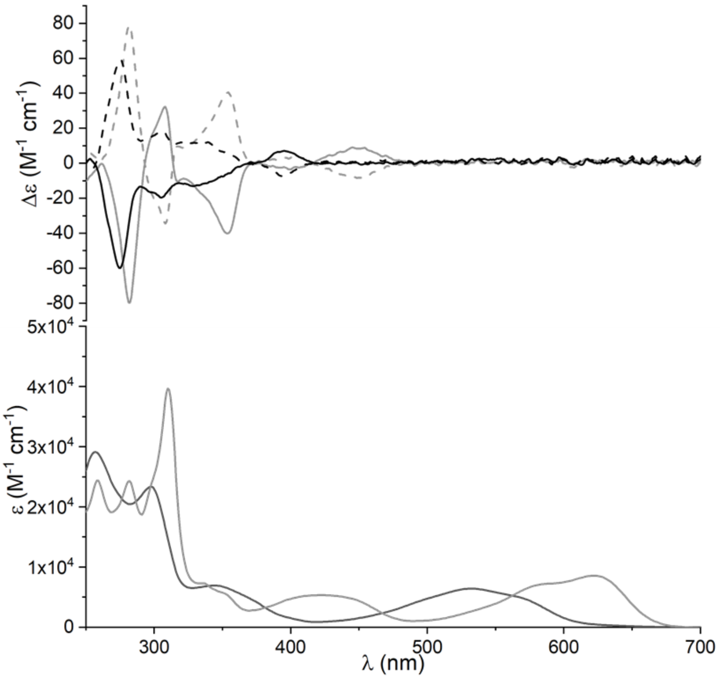

Figure S9. (Top) UV-vis ECD spectra and (bottom) the corresponding molar extinction coefficient (ε) of compound **2** (blank) and **2•H⁺** (grey) in air-equilibrated acetonitrile solution (C *ca.* 10^{-5} M) at 293 K. *M* full and *P* dashed lines.

Absorption, emission and ECD spectra of compounds **3a** and **3a•H+**

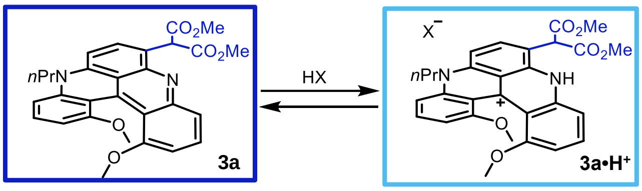

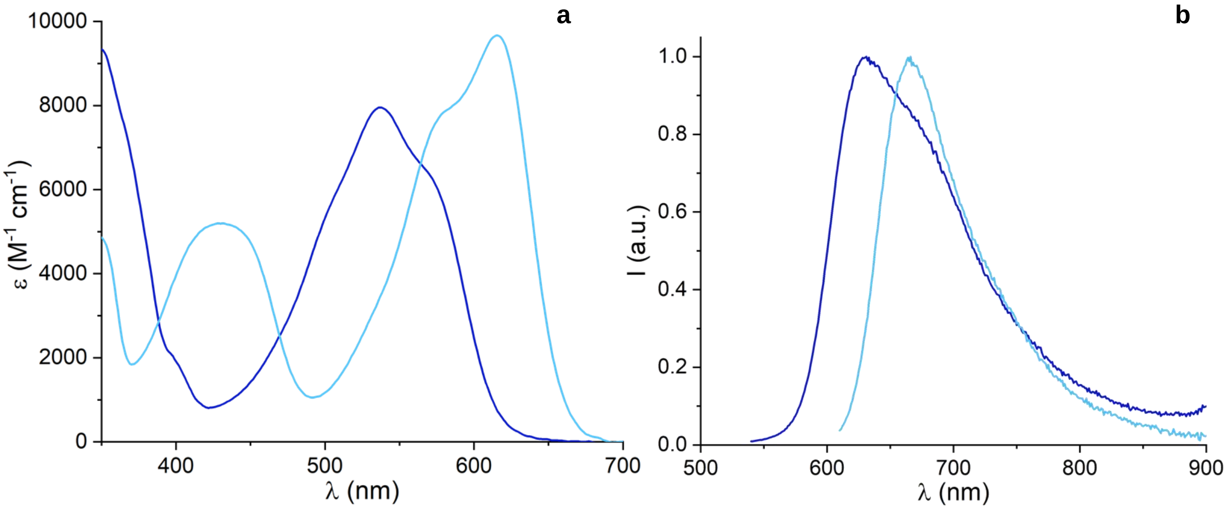

Figure S10. a) Absorption and b) normalized emission spectra of compound **3a** (blue) and **3a•H+** (light blue) in air-equilibrated acetonitrile solution (C *ca*. 10^{-5} M) at 293 K.

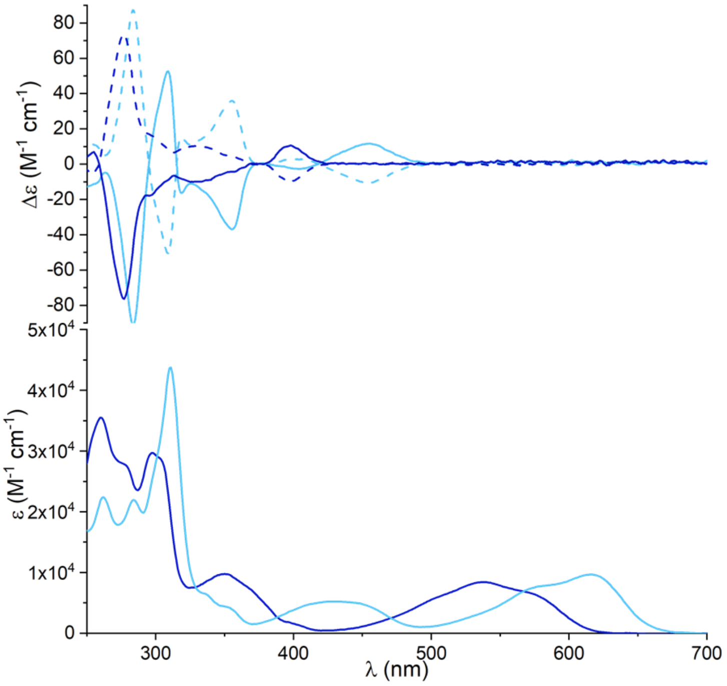

Figure S11. (Top) UV-vis ECD spectra and (bottom) the corresponding molar extinction coefficient (ε) of compound **3a** (blue) and **3a•H+** (light blue) in air-equilibrated acetonitrile solution (C *ca.* 10^{-5} M) at 293 K. *M* full and *P* dashed lines.

Absorption and emission spectra of compound **r-3a**

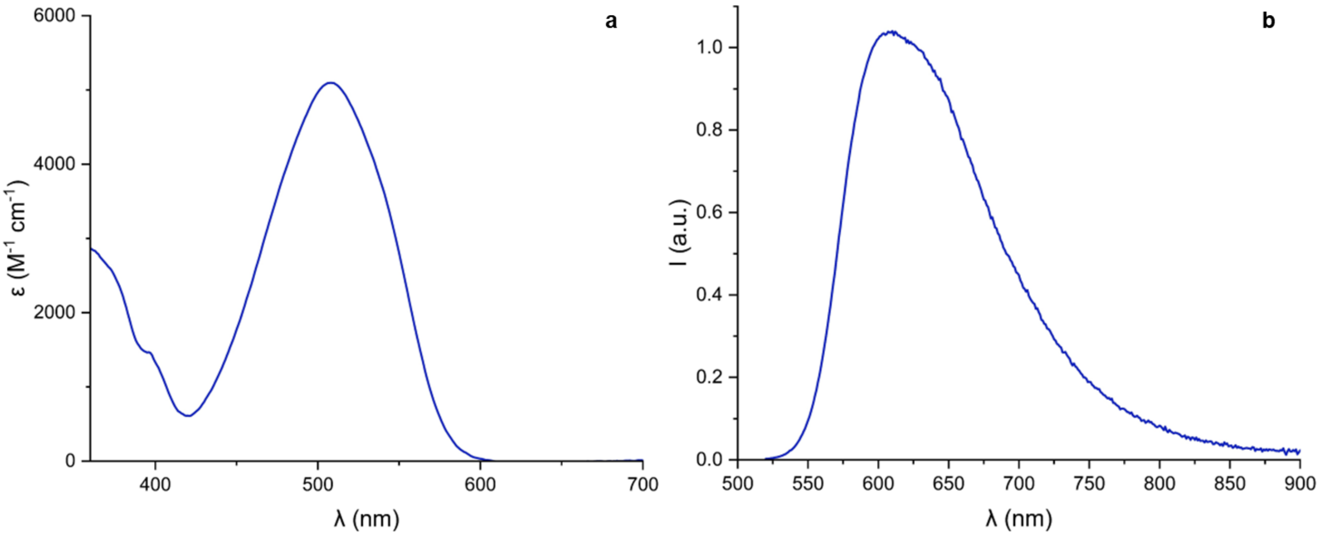

Figure S12. a) Absorption and b) normalized emission spectra of compound **r-3a** in air-equilibrated acetonitrile solution (C *ca*. 10^{-5} M) at 293 K.

Absorption, emission and ECD spectra of compounds **4aa** and **4aa•H$^+$**

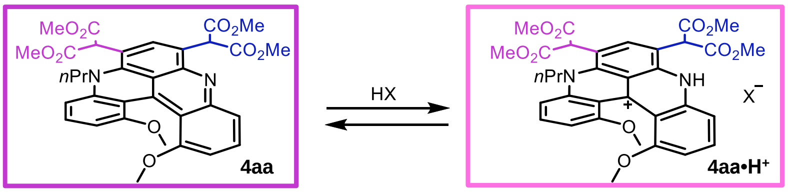

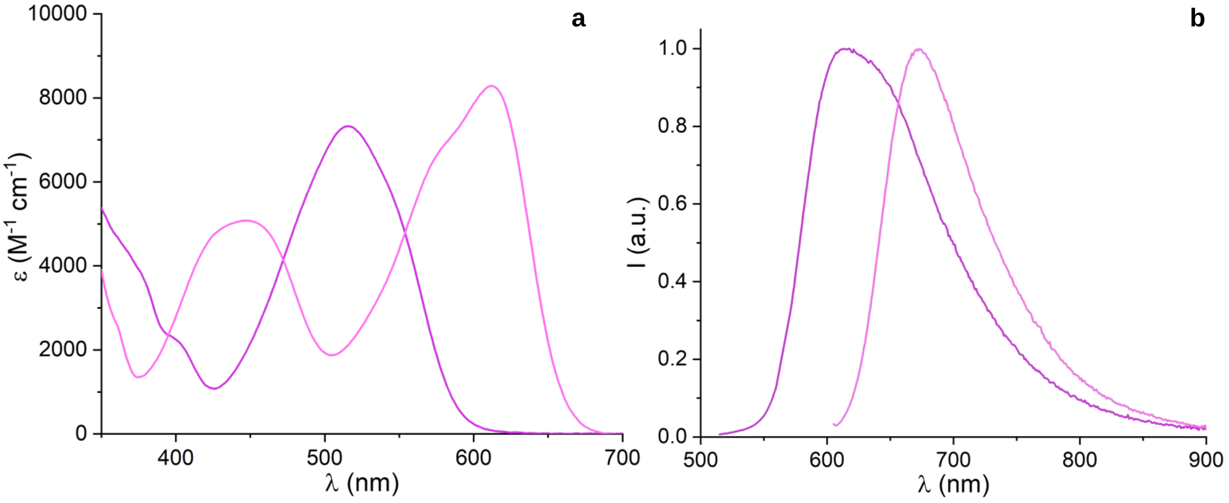

Figure S13. a) Absorption and b) normalized emission spectra of compound **4aa** (magenta) and **4aa•H$^+$** (pink) in air-equilibrated acetonitrile solution (C *ca*. 10^{-5} M) at 293 K.

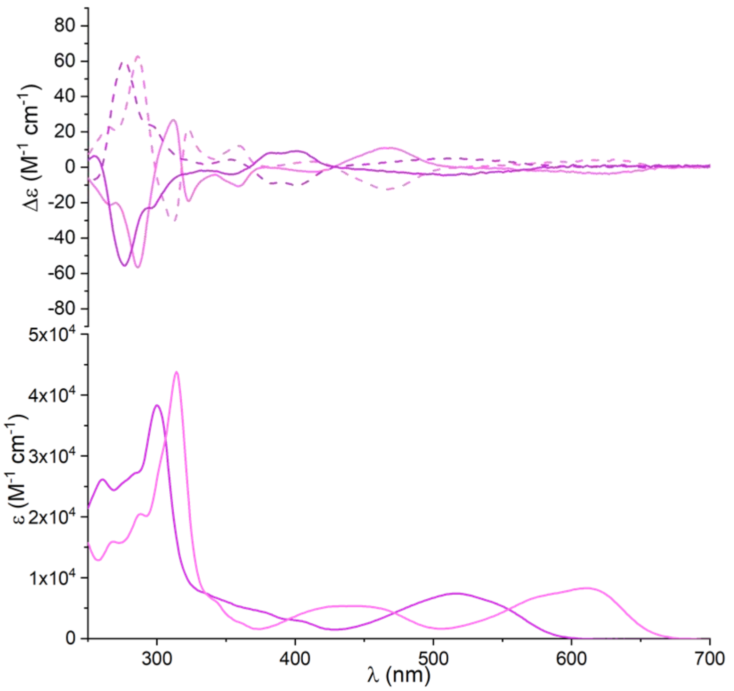

Figure S14. (Top) UV-vis ECD spectra and (bottom) the corresponding molar extinction coefficient (ε) of compound **4aa** (magenta) and **4aa•H⁺** (pink) in air-equilibrated acetonitrile solution (C *ca.* 10^{-5} M) at 293 K. *M* full and *P* dashed lines.

Absorption, emission and ECD spectra of compounds 5 and 5•H⁺

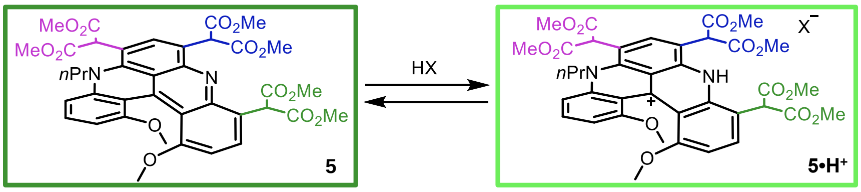

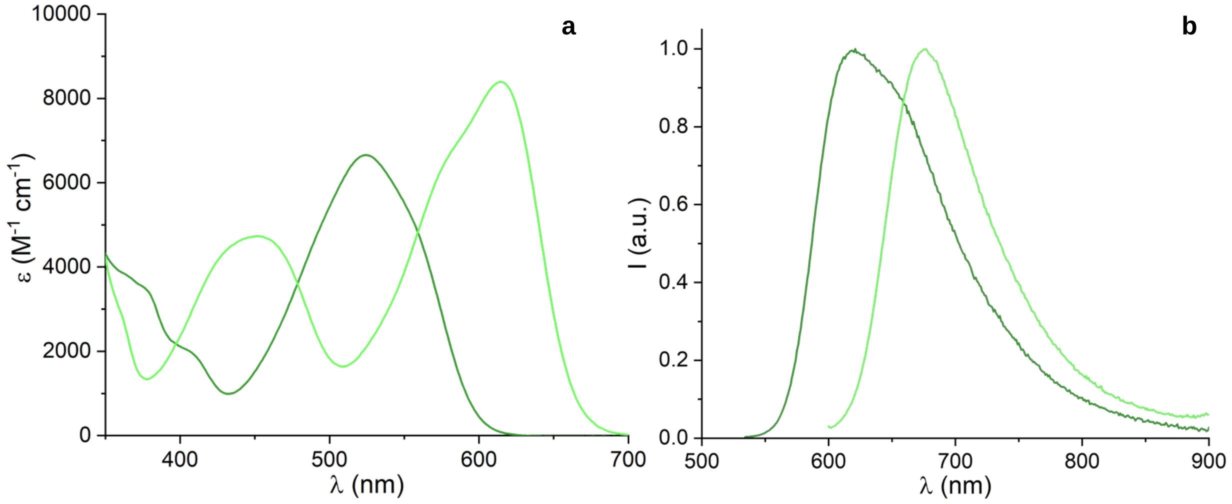

Figure S15. a) Absorption and b) normalized emission spectra of compound **5** (green) and **5•H⁺** (light green) in air-equilibrated acetonitrile solution (C *ca.* 10^{-5} M) at 293 K.

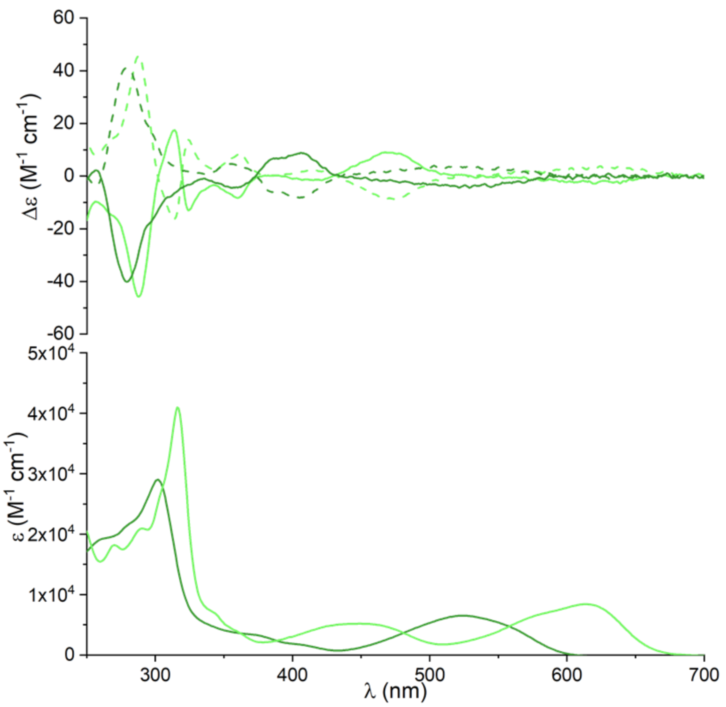

Figure S16. (Top) UV-vis ECD spectra and (bottom) the corresponding molar extinction coefficient (ε) of compound **5** (green) and **5•H**$^+$ (light green) in air-equilibrated acetonitrile solution (C *ca.* 10^{-5} M) at 293 K. *M* full and *P* dashed lines.

<u>Absorption, emission and ECD spectra of compounds 6 and 6•H$^+$</u>

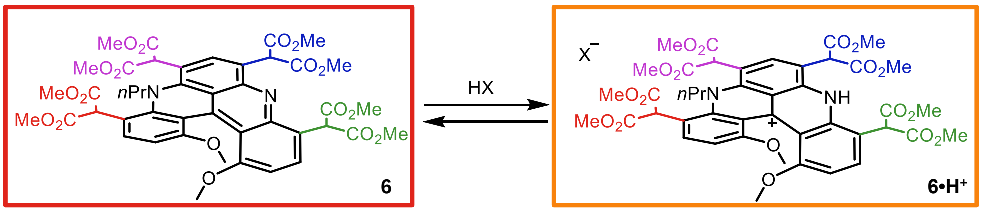

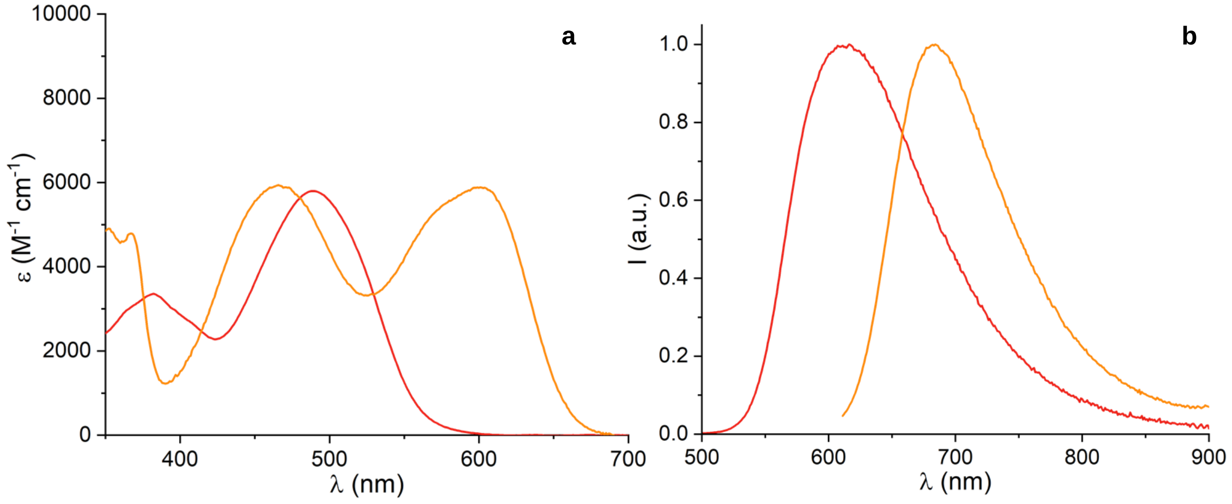

Figure S17. a) Absorption and b) normalized emission spectra of compound **6** (red) and **6•H**$^+$ (orange) in air-equilibrated acetonitrile solution (C *ca.* 10^{-5} M) at 293 K.

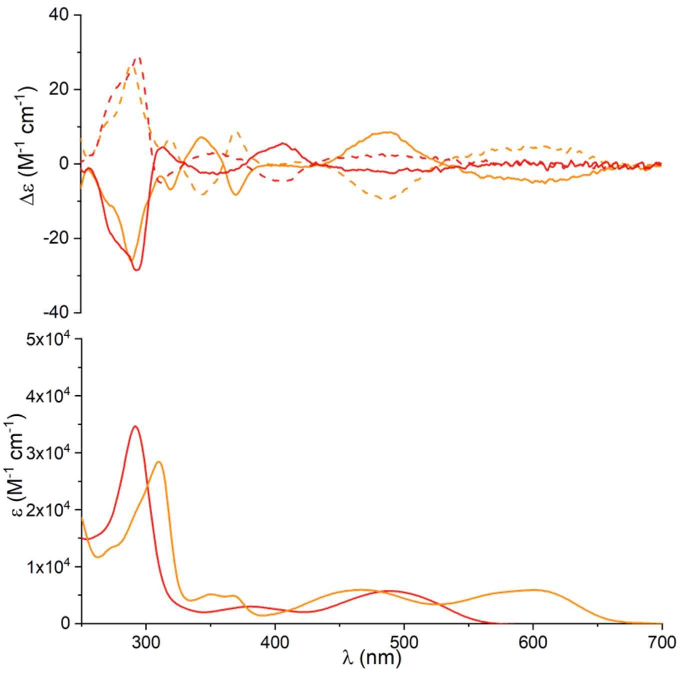

Figure S18. (Top) UV-vis ECD spectra and (bottom) the corresponding molar extinction coefficient (ε) of compound **6** (red) and **6•H**$^+$ (orange) in air-equilibrated acetonitrile solution (C *ca.* 10^{-5} M) at 293 K. *M* full and *P* dashed lines.

Lifetime measurements

For compounds **2** and **3aa** a component with a time constant around 10 ns and relative amplitude <2 was observed. Consequently, the corresponding fluorescence decays have been fitted with biexponential functions. In addition, the signals of fluorescence decays of compounds **2•H**$^+$, **3a•H**$^+$, **4aa•H**$^+$, **5•H**$^+$ and **6•H**$^+$ are noisy due to the low fluorescence quantum yield of these compounds.

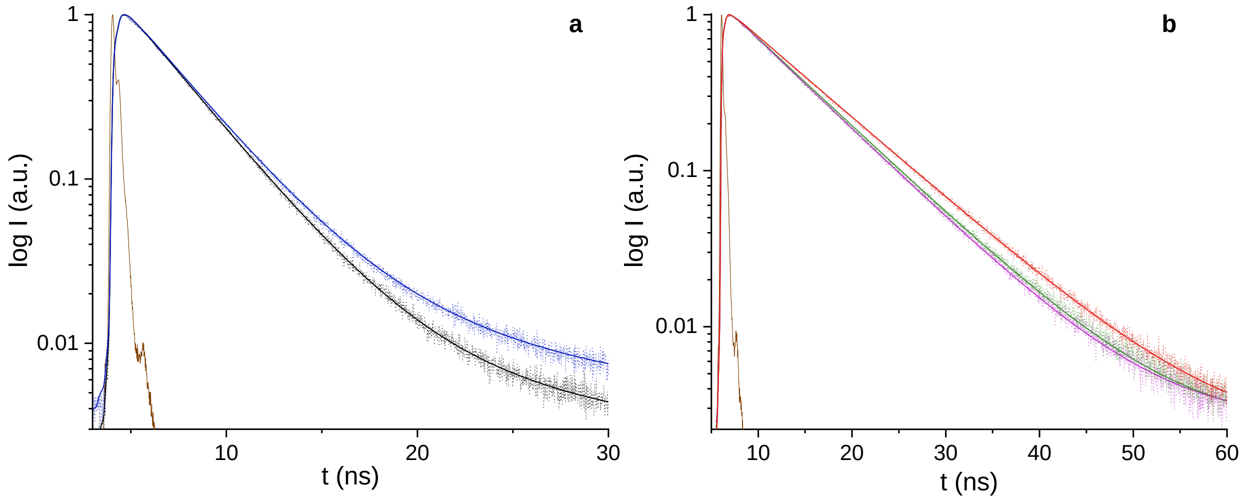

Figure S19. Fluorescence decays of air-equilibrated acetonitrile solutions (C *ca.* 10^{-5} M) at 293 K of compounds a) **2** (blank), **3a** (blue) and b) **4aa** (magenta), **5** (green) and **6** (red). The corresponding biexponential (graph a) and monoexponential (graph b) fitting functions are shown as solid lines. The instrument response function (IRF) is also reported (brown line).

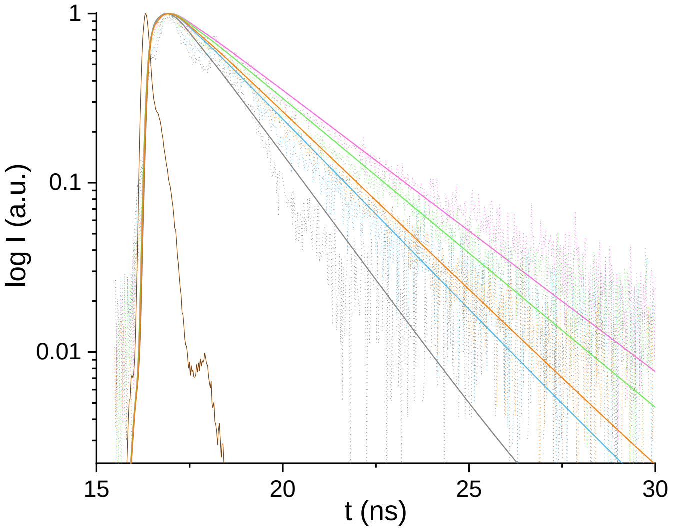

Figure S20. Fluorescence decays of air-equilibrated acetonitrile solutions (C *ca.* 10^{-5} M) at 293 K of compounds a) **2•H**$^+$ (grey), **3a•H**$^+$ (light blue), **4aa•H**$^+$ (pink), **5•H**$^+$ (light green) and **6•H**$^+$ (orange). The corresponding monoexponential fitting functions are shown as solid lines. The instrument response function (IRF) is also reported.

X-ray structure determination

Comparison

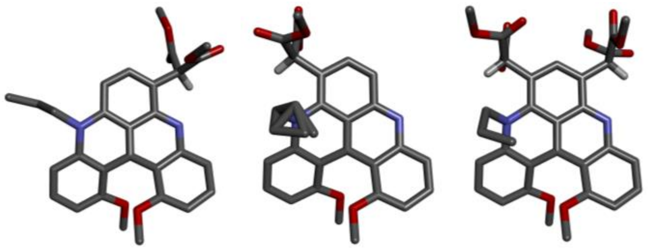

3a r-3a 4aa

Figure S21. X-ray structures of **3a**, **r-3a** and (*M*)-**4aa**. (*M*)-configurations for racemic **3a** and **r-3a** are arbitrarily selected. Only the malonate hydrogen atoms are drawn. Important structural disorder is noticed for the propyl chain of **r-3a**.

Compound **3a** (CCDC 2161999)

Table S2. Crystal data and structure refinement for compound **3a**.

Empirical formula	$C_{29}H_{28}N_2O_6$
Formula weight	500.53
Temperature/K	150.00(10)
Crystal system	Monoclinic
Space group	*P*21/*c*
a/Å	8.04610(10)
b/Å	14.5071(2)
c/Å	21.3030(3)
α/°	90
β/°	92.7440(10)
γ/°	90
Volume/Å^3	2483.75(6)
Z	4
ρ_{calc}g/cm^3	1.339
μ/mm^{-1}	0.772
F(000)	1056
Crystal size/mm^3	0.448 x 0.389 x 0.218
Theta range for data collection/°	3.688 to 73.901
Index ranges	-10<=h<=9, -18<=k<=18, -26<=l<=26
Reflections collected	95765
Independent reflections	4978 [R_{int} = 0.0225]
Completeness to theta = 67.684°	99.8 %
Absorption correction	Gaussian
Max. and min. transmission	1.000 and 0.232
Refinement method	Full-matrix least-squares on F2

Data/restraints/parameters	4978/0/340
Goodness-of-fit on F^2	1.057
Final R indexes [I>=2σ (I)]	R_1 = 0.0360, wR_2 = 0.0919
Final R indexes [all data]	R_1 = 0.0365, wR_2 = 0.0923
Largest diff. peak/hole / e Å^{-3}	0.253/-0.206

Comments on the model:

The crystal was a twin but only the major component was integrated.

Asymmetric unit with displacement ellipsoids at 50 percent probability.

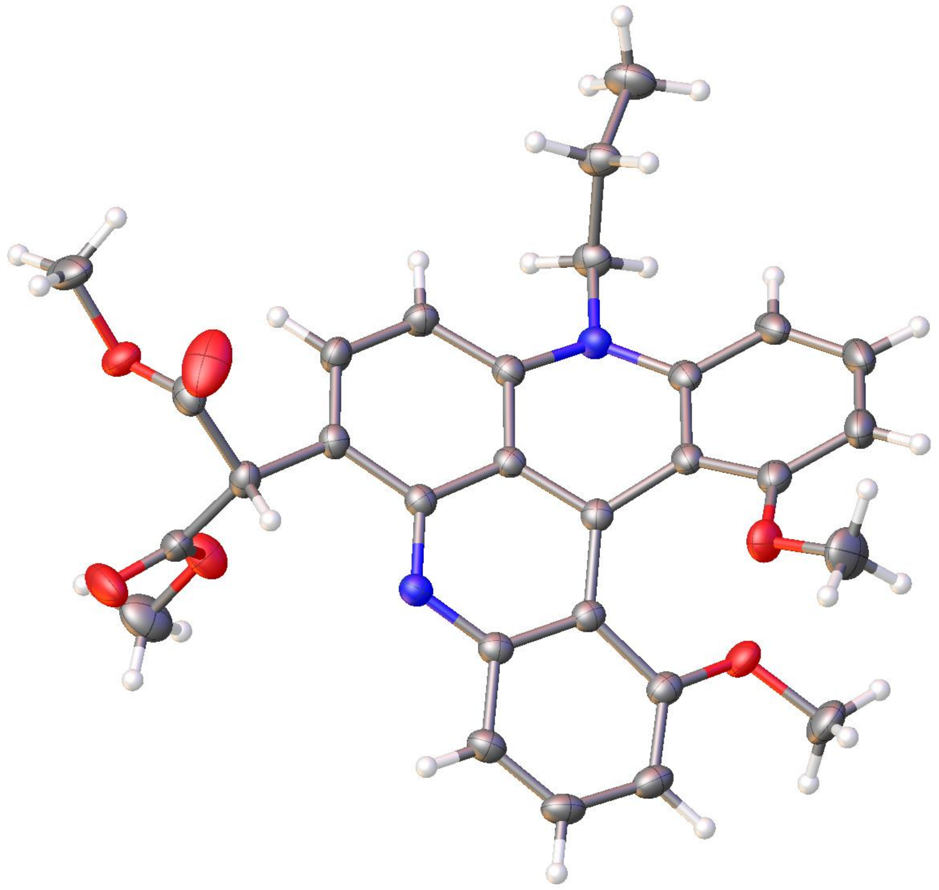

Compound **r-3a** (CCDC 2162000)

Table S3. Crystal data and structure refinement for compound **r-3a**.

Empirical formula	$C_{30}H_{30}Cl_2N_2O_6$
Formula weight	585.46
Temperature/K	180.01(10)
Crystal system	Monoclinic
Space group	*P*21/*c*
a/Å	11.0276(2)
b/Å	22.1679(3)
c/Å	11.5255(2)
α/°	90
β/°	96.5710(10)
γ/°	90
Volume/Å^3	2799.00(8)
Z	4
ρ_{calc}g/cm^3	1.389
μ/mm^{-1}	2.482
F(000)	1224
Crystal size/mm^3	0.293 x 0.1 x 0.025
Theta range for data collection	3.988 to 70.763
Index ranges	-13<=h<=13, -26<=k<=25, -14<=l<=12
Reflections collected	12050
Independent reflections	5263 [R_{int} = 0.0216]
Completeness to theta = 67.684°	99.9 %
Absorption correction	Gaussian
Max. and min. transmission	1.000 and 0.577
Refinement method	Full-matrix least-squares on F^2
Data/restraints/parameters	5263/33/386
Goodness-of-fit on F^2	1.041
Final R indexes [I>=2σ (I)]	R_1 = 0.0459, wR_2 = 0.1188
Final R indexes [all data]	R_1 = 0.0504, wR_2 = 0.1229
Extinction coefficient	n/a
Largest diff. peak/hole / e Å^{-3}	0.769/-0.877

Comments on the model:

A disordered *n*-Pr group was refined as two components, using same distances (SADI) restraints on C-C distances and SIMU restraints on displacement parameters.

Asymmetric unit with displacement ellipsoids at 50 percent probability.

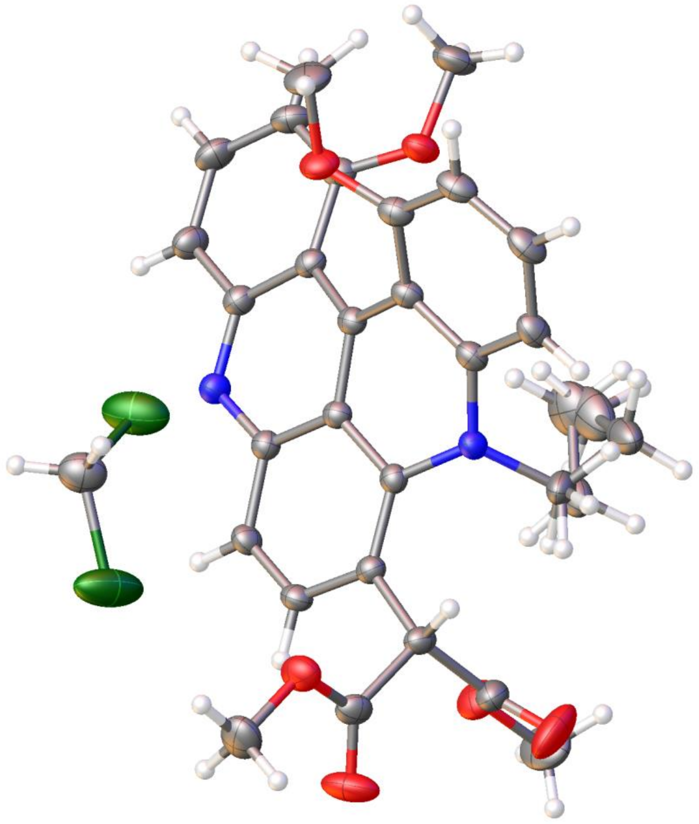

Compound **4aa** (CCDC 2162001)

Table S4. Crystal data and structure refinement for compound **4aa**.

Empirical formula	$C_{34}H_{34}N_2O_{10}$
Formula weight	630.63
Temperature/K	120.02(12)
Crystal system	Orthorhombic
Space group	*P*21 21 21
a/Å	12.44796(13)
b/Å	12.72252(11)
c/Å	c = 19.2214(2)
α/°	90
β/°	90
γ/°	90
Volume/Å^3	3044.09(5)
Z	4
ρ_{calc}g/cm^3	1.376
μ/mm^{-1}	0.849
F(000)	1328
Crystal size/mm^3	0.18 x 0.07 x 0.02
Theta range for data collection	4.167 to 74.224
Index ranges	-15<=h<=15, -15<=k<=13, -23<=l<=23
Reflections collected	33159
Independent reflections	6133 [R(int) = 0.0339]
Completeness to theta = 67.684°	100.0 %
Absorption correction	Analytical
Max. and min. transmission	0.981 and 0.877
Refinement method	Full-matrix least-squares on F^2
Data/restraints/parameters	6133/0/423
Goodness-of-fit on F^2	1.032
Final R indexes [I>=2σ (I)]	R_1 = 0.0320, wR_2 = 0.0832
Final R indexes [all data]	R_1 = 0.0350, wR_2 = 0.0852
Absolute structure parameter	-0.10(16)
Extinction coefficient	n/a
Largest diff. peak/hole / e Å^{-3}	0.152 and -0.194

Asymmetric unit with displacement ellipsoids at 50 percent probability.

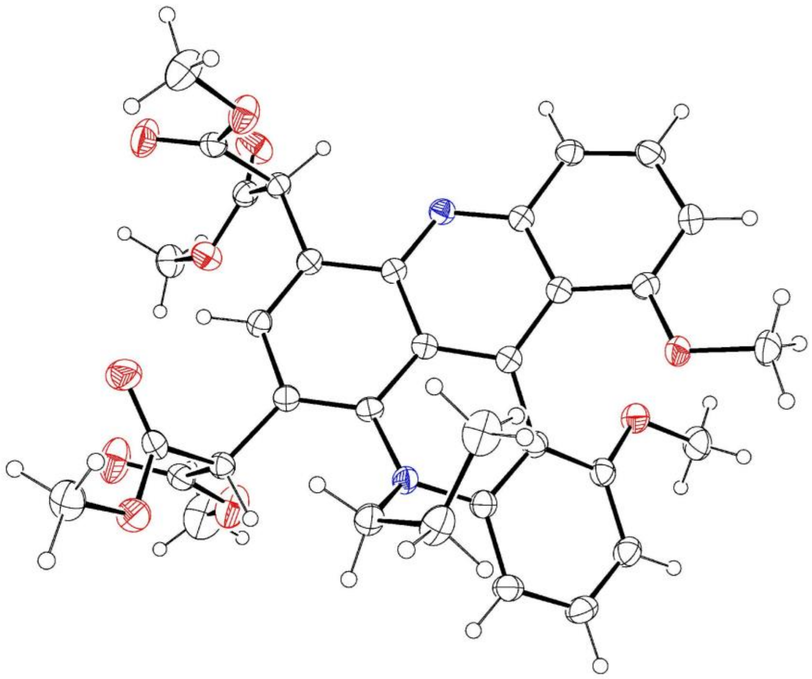

Computational results

Formation of betaine-type product 10

To gain insight on the possible formation of betaine product **10**, a possible pathway was computed starting from **3g** without the aid of the metal. Although the metal (Ru or Rh) could catalyze this pathway, a free-metal pathway that is kinetically available was found. The reaction first starts with a proton transfer CH➞NH. This step is analogous to that shown in Figure 4 of the main text (**M-TS-I3-O**). Intermediate **Ter-1** is found at only −0.7 kcal·mol^{-1} from **3g** species. Then, in a concerted manner, a molecule of trifluoroethanol is formed, with a relative barrier of 24.3 kcal·mol^{-1} (**Ter-TS2**), to form the ketene intermediate **Ter-2**. Finally, due to the high energy of **Ter-2** (lying at 12.7 kcal·mol^{-1}), the electrophilic ketene moiety is trapped by the N-atom, yielding the betaine-type product **10** found at −10.6 kcal·mol^{-1} from the initial reactant.

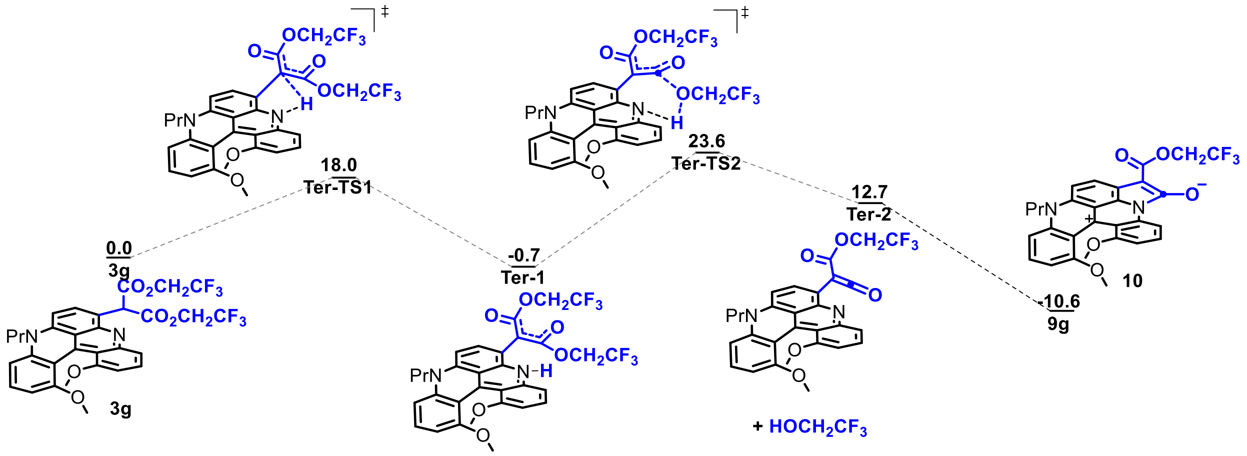

Figure S22. Computed Gibbs free energy profiles for the formation of the thermodynamic product **10** from **3g**. Energies in kcal·mol^{-1}.

Details concerning the formation of mono-malonate products 3a and r-3a

Precisely, the mechanism starts with the insertion of metal-carbene **I0** into the activated C–H bond of helicene **2** at position 6. Kinetically, different behaviors are found for Ru *vs* Rh systems (Figure 4, right panel). While the insertion constitutes the rate-determining step (RDS) for Ru (ΔG=19.1 kcal·mol^{-1}, **TS-I0**), this step occurs through a barrierless process for the Rh catalyst.[21] It yields intermediate **I1** which rapidly evolves to enol intermediate **I2** via an intramolecular CH➞OH proton transfer involving one ester group. The barriers for this step, via **TS-I1**, are 13.3 and 9.0 kcal·mol^{-1} with Ru and Rh complexes, respectively. At that stage, **I2** can formally react to form product **3a** directly following a keto-enol tautomerization but, unfortunately, such a step could not be located although it is known to happen experimentally.[22] Alternatively, a three-step process can be envisaged via a first proton transfer from the O to the neighboring N atom through a barrierless process (**I2➞I3**).[23] In **I3**, positioning the hydrogen on the N atom strongly stabilizes the intermediate structure by more than 10.0 kcal·mol^{-1}, in both Ru and Rh pathways. Then, after a migration of catalysts [M] to the O-atom of the ester carbonyl group (**I3➞I3-O**), a final proton transfer from the N to the carbenoid C atom takes place (**I3-O➞I4**) with barriers of ca. 15.0 kcal·mol^{-1}, **TS-I3-O**, for both systems. Finally, product **3a** is released to the media in a very exergonic step starting a new catalytic cycle.[24] These calculations transcribe effectively the experimental observations. For Ru, the first stages of the reaction encompass a slightly endergonic process (**I0➞I2**), while a very exergonic transformation is found for Rh. In addition, the C(sp^2)–H insertion step is rate-determining for Ru and barrierless for Rh.

Next, to account for the chemo and regioselectivity, the formation of **r-3a** was investigated (Figure 4, left panel). Initial steps are similar to those already described (*vide supra*). For Ru, carbene insertion takes place with almost the same barrier than for **3a**, 19.0 kcal·mol^{-1} (**r-TS-I0**). However, intermediate **r-I1** presents a higher relative energy in comparison to **I1** (6.0 vs 1.8 kcal·mol^{-1}, respectively). This difference can be attributed to the steric bulk of the neighboring *n*-Pr chain in **r-I1**. In the same vein, the subsequent proton transfer via **r-TS-I1** presents a higher energy of 23.5 kcal·mol^{-1}.

Finally, intermediate **r-I2** presents a relative energy of 8.1 kcal·mol^{-1} from **I0**, again higher than analogous intermediate **I2**. For Rh, the mechanism is identical with very different relative energies, nevertheless. The first insertion step (**r-TS-I0**) is a barrierless process (as for the formation of **3a**) yielding **r-I1**, found at −12.3 kcal·mol^{-1}. This intermediate rapidly evolves to **r-I2** through a barrier of only 10.6 kcal·mol^{-1} (**r-TS-I1)**. Finally, **r-I2** ought to form **r-3a** through a keto-enol tautomerization mechanism.

Formation of bis-malonate product **4aa**

Care was also taken to study the formation of **4aa**, the bis-functionalized adduct obtained only under Rh_2-catalysis. DFT calculations for the initial steps in both Rh and Ru series are shown in Figure S23, bottom and top panels. Addition of Rh-carbene **I0** to **3a** or **r-3a** are barrierless processes, yielding isoenergetic intermediates **r-Rh-I5** and **Rh-I5** lying at −15.0 and −14.6 kcal·mol^{-1}, respectively. These intermediates, in all likelihood, then converge to product **4aa** via mechanisms similar to that reported in Figure 4. In the case of Ru, transition states for the second metal carbene addition were also located. In the first step (**I0➞Ru-I5**), reaction of **3a** with the Ru-carbene is kinetically accessible with an activation barrier of 18.5 kcal·mol^{-1}, via **Ru-TS-I4**. However, formation of **Ru-I5** is strongly endergonic by 6.3 kcal·mol^{-1}. This suggests that an equilibrium occurs in favor of the initial components **3a** and **I0**, hence preventing the formation of **4aa** under CpRu catalysis. Finally, the theoretical case of a reactivity between **r-3a** and **I0** under Ru-catalysis was considered. In this case, the formation of the double addition product is not feasible due to a kinetic barrier of 28.1 kcal·mol^{-1} found for **r-Ru-TS-I4**.

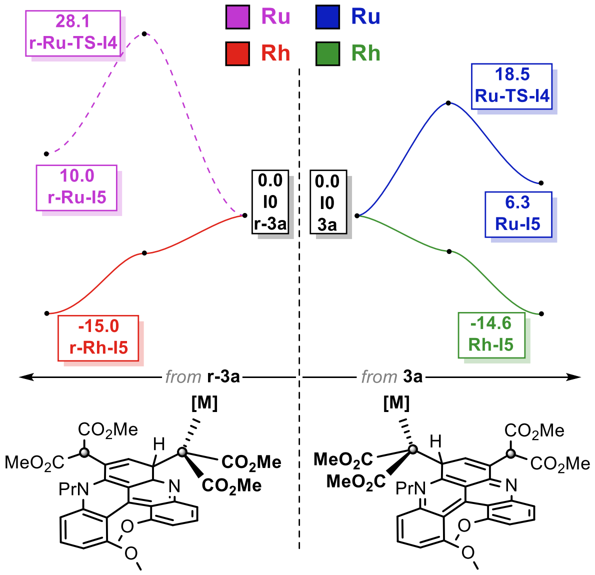

Figure S23. Computed Gibbs energy profiles for the initial steps of **4aa** formation from **3a** (right) and **r-3a** (left) under CpRu and dirhodium catalysis. All energies in kcal mol^{-1}.

TDDFT calculation of optical properties

The trend of S0-S1 transition energies of compounds **2**, **3a**, **r-3a**, **4aa**, **5** and **6**, i.e., upon subsequent substitution of the helicene core of **2** with malonate groups, was investigated by TDDFT calculations. Calculation details are provided in the dedicated section. Table S5 reports the experimental transition wavelengths, the calculated transition wavelengths at two different levels, and a series of dihedral angles (see diagrams) measured on DFT-optimized structures which quantify the progressive deformation of the partially saturated ring upon substitution.[25] The correlation between experimental and calculated transition wavelengths is displayed in Figure S24.

Table S5. Experimental and calculated data for compounds **2**, **3a**, **r-3a**, **4aa**, **5** and **6**.

Compound	Experimental		Dihedral angles [1]			CAM-B3LYP [2]		B3LYP [2]	
	λ_{max} (nm)	$\Delta\lambda_{max}$ from **2**	α (deg)	β (deg)	γ (deg)	Calc. λ_{max} (nm)	Calc. $\Delta\lambda_{max}$ from **2**	Calc. λ_{max} (nm)	Calc. $\Delta\lambda_{max}$ from **2**
**2**	533	0	18.6	15.7	163.5	463	0	526	0
**3a**	537	+4	18.6	16.1	163.5	463	0	526	0
**r-3a**	507	–26	28.8	15.8	152.3	454	–9	518	–8
**4aa**	516	–17	28.2	16.2	152.7	455	–8	520	–6
**5**	524	–9	28.0	16.1	152.1	457	–6	522	–4
**6**	489	–44	30.7	19.2	147.5	437	–36	504	–22

[1] Values of dihedral angles (defined in the diagram below) measured on the lowest energy B3LYP-D3/6-31+G(d,p)/SMD structure.
[2] Result of TDDFT calculations run with CAM-B3LYP or B3LYP functional, def2-TZVP basis sets and IEF-PCM solvent model for acetonitrile, using the lowest-energy B3LYP-D3/6-31+G(d,p)/SMD structure in each case.

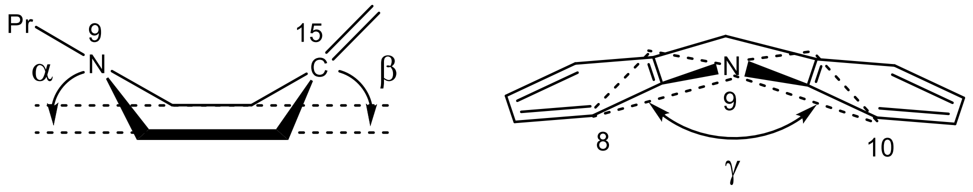

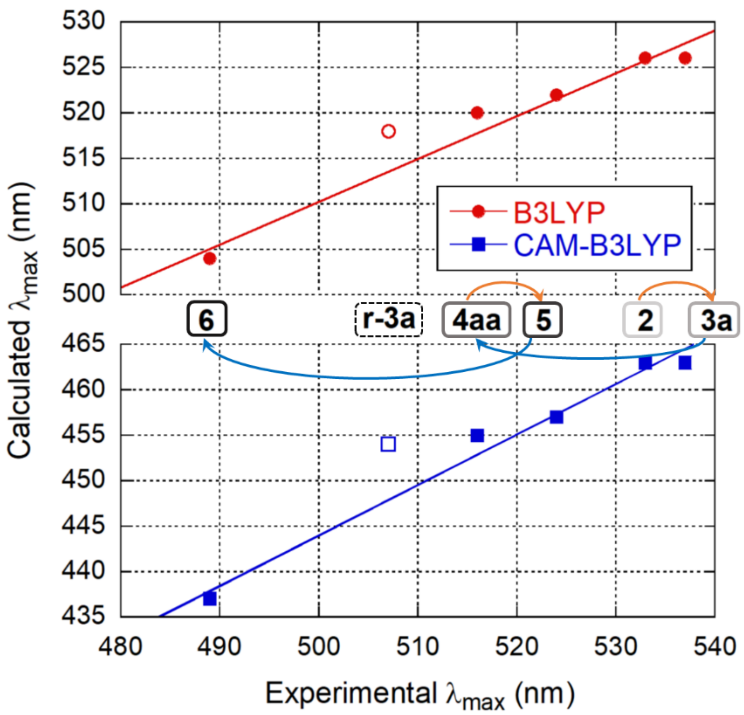

Figure S24. Correlation between experimental and calculated S0-S1 transition energies data for compounds **2**, **3a**, **r-3a**, **4aa**, **5** and **6**. See footnote [2] to Table S5 for details. The curved arrows help visualizing consecutive red/blue shift upon progressive substitution of the helicene core with malonate moieties.

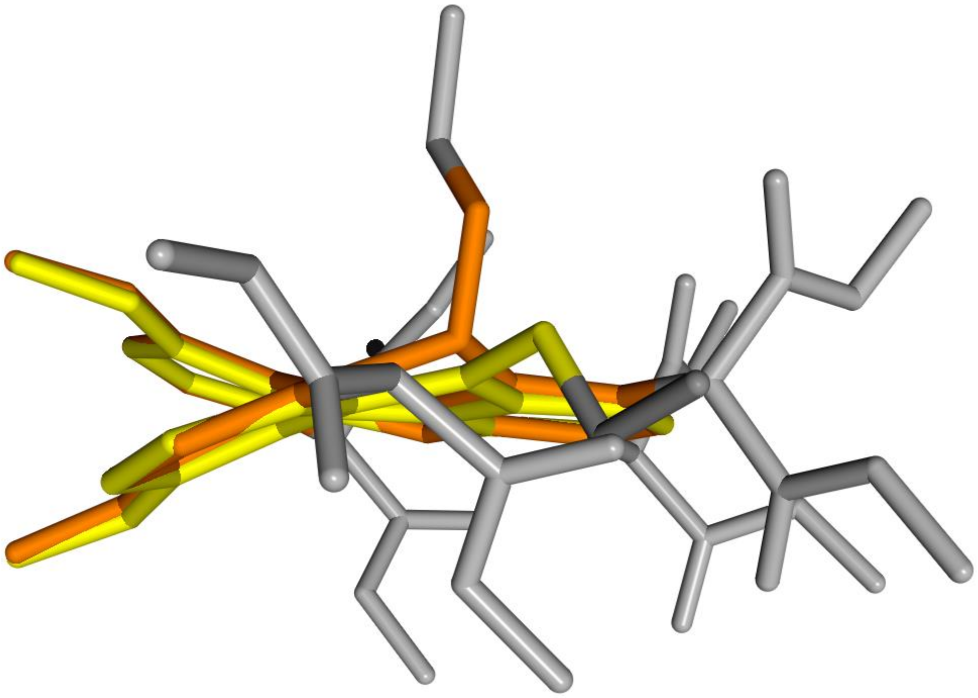

Figure S25. Overlay between DFT-optimized geometries of **2** and **6**.

pK_a Determination - nanoparticle-based method [26]

General Figure

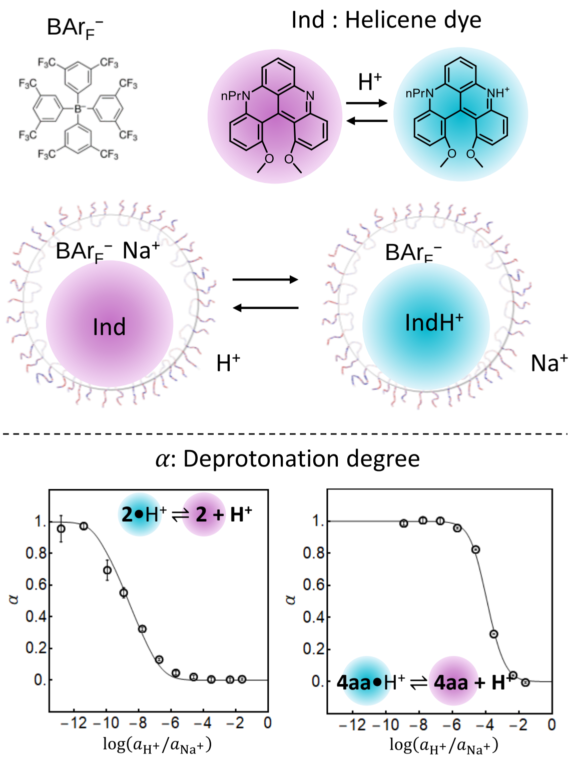

Figure S26. Proton transfer ability of **2**•H$^+$ to **5**•H$^+$ using a nanoparticle-based method for pK_a value measurements (Top). Examples of pK_a determination for **2**•H$^+$ and **5**•H$^+$ (bottom).

Particle preparation

8.0 mg of DOS, 5.0 mg of F127, 1.74 mg of NaTFPB, 0.44, 0.59, 0.75, 0.90 or 1.06 mg of compounds **2**, **3a**, **4aa**, **5** and **6**, respectively, were dissolved in 2 mL of THF. To form nanoparticles, 2 mL of each mixture was injected into a 100 mL beaker containing 50 mL of deionized water with stirring at 600 rpm. THF was then removed by blowing compressed air into the beaker for more than 3 hours. As this process removes not only THF but also a part of water, deionized water was added to adjust the final volume of the nanoparticle solution to 50 mL.

Buffer preparation

A stock solution containing 40 mM boronic acid, 40 mM citric acid and 40 mM monosodium phosphate was prepared. The pH of this solution was tracked by a commercial 827 pH lab pH meter and adjusted to 7.4 by adding 1 M NaOH. The volumes of NaOH solution added to prepare each pH buffer were recorded since sodium ion concentration should be known for equilibrium constant calculation. Then, the pH-adjusted buffer solution was diluted to reach a boronic acid, citric acid and monosodium phosphate of 20 mM.

Absorbance measurements

100 µL of the particle solution and 100 µL of each buffer solution were dispensed into a well of a 96 microplate. Absorption spectra of each well was recorded by TECAN plate reader. As malonate dyes present ratiometric

spectral shift, wavelengths of the two peaks were chosen for deprotonation degree calculation. Equation below was used to obtain deprotonation degrees;[27]

$$\alpha = \frac{\log\left(\frac{I_{P2}I_1}{I_{P1}I_2}\right)}{\log\left(\frac{I_{P2}I_{D1}}{I_{P1}I_{D2}}\right)}$$

where the two chosen wavelengths are denoted as 1 and 2, P and D represents fully protonated and deprotonated form and *I* is the light intensity calculated based on absorbance.

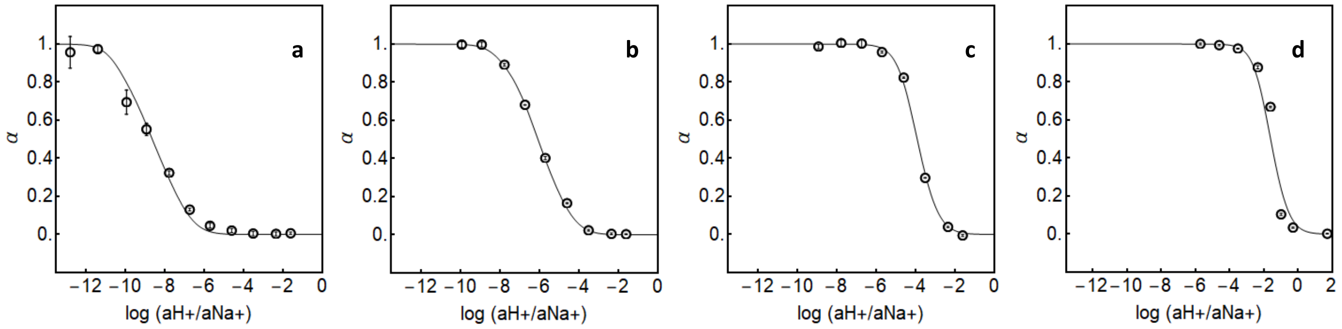

Figure S27. pH response curves of compounds a) **2**, b) **3a**, c) **4aa** and d) **5**.

To obtain the pK_a value, the ion exchange constant for the following reaction (K_{ex}) was obtained in the same manner as reported earlier:[26]

$$H^+(aq) + Na^+(org) + Ind(org) \rightleftarrows Na^+(aq) + IndH^+(org)$$

where Ind and IndH$^+$ are the deprotonated and protonated lipophilic helicene dye. The K_a and the above-mentioned exchange constant K_{ex} are related as follows:[26]

$$K_a = \frac{[H^+]_{org}[Ind]_{org}}{[IndH^+]_{org}} = \frac{K_{Na^+,H^+}}{K_{ex}}$$

where square brackets denote concentrations while K_{Na^+,H^+} is the exchange constant for the uncomplexed sodium and hydrogen ions between the organic phase and the aqueous solution. The $\log K_{Na^+,H^+}$ value was determined earlier as 0.9 with the help of an ionic solvatochromic dye.[26] This approach allowed us to obtain the desired pK_a values of the various helicene structures in the organic phase.

Cell imaging

General conditions for the cell imaging experiments are specified on page S6.

Live and fixed cell (PFA) imaging procedures were performed by treatment of Hela-MZ cells with DMSO solutions of **2**, esters **3a**, **4aa**, **5** and amides **12a**, **12b**, **12c** and **13**. Compound **6** is precipitating in water based medium, therefore the image analysis in this case cannot be considered. Compound **12d** is not soluble in DMSO and was not used for the analysis.

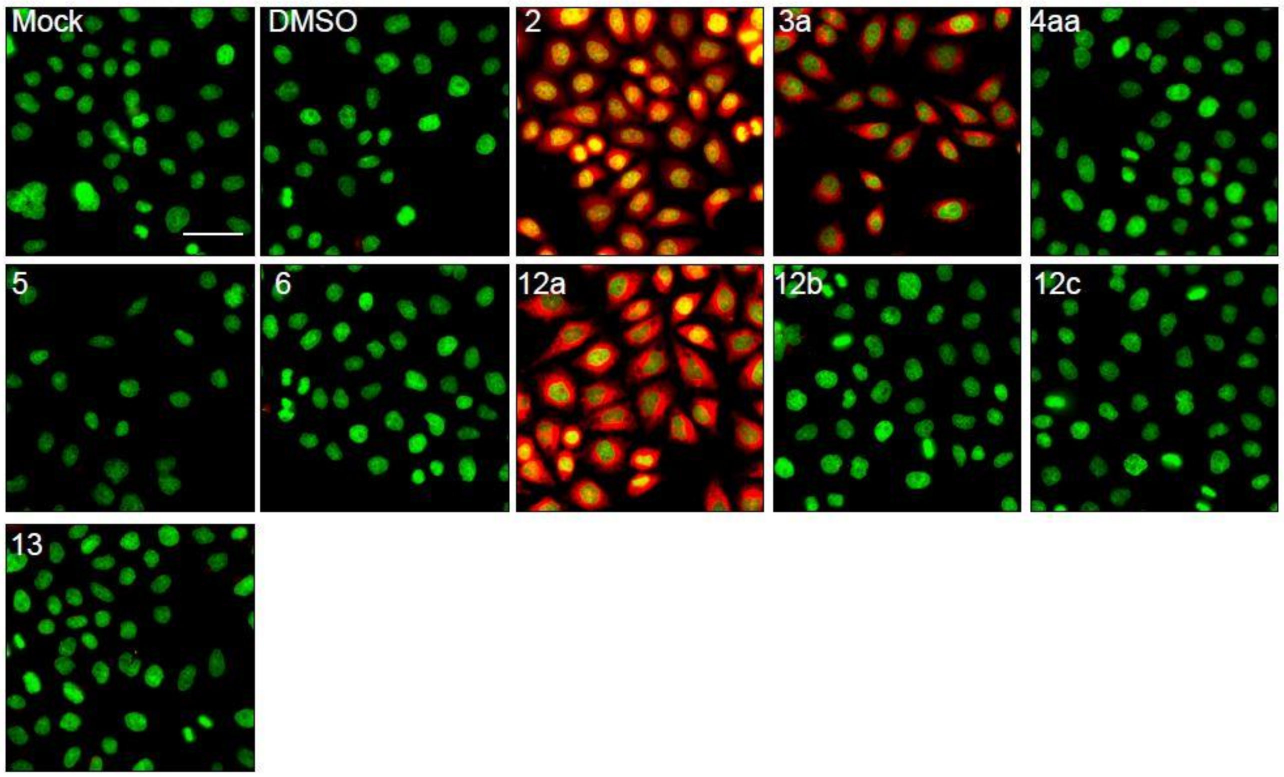

Figure S28. Microscopy images of Hela-MZ cells treated for 60 min with DMSO solutions of **2**, esters **3a**, **4aa**, **5** and amides **12a**, **12b**, **12c** and **13** (green = DAPI channel, Red = Txred channel).

^{1}H, ^{13}C, ^{19}F NMR spectra, IR spectra and HRMS reports of new compounds

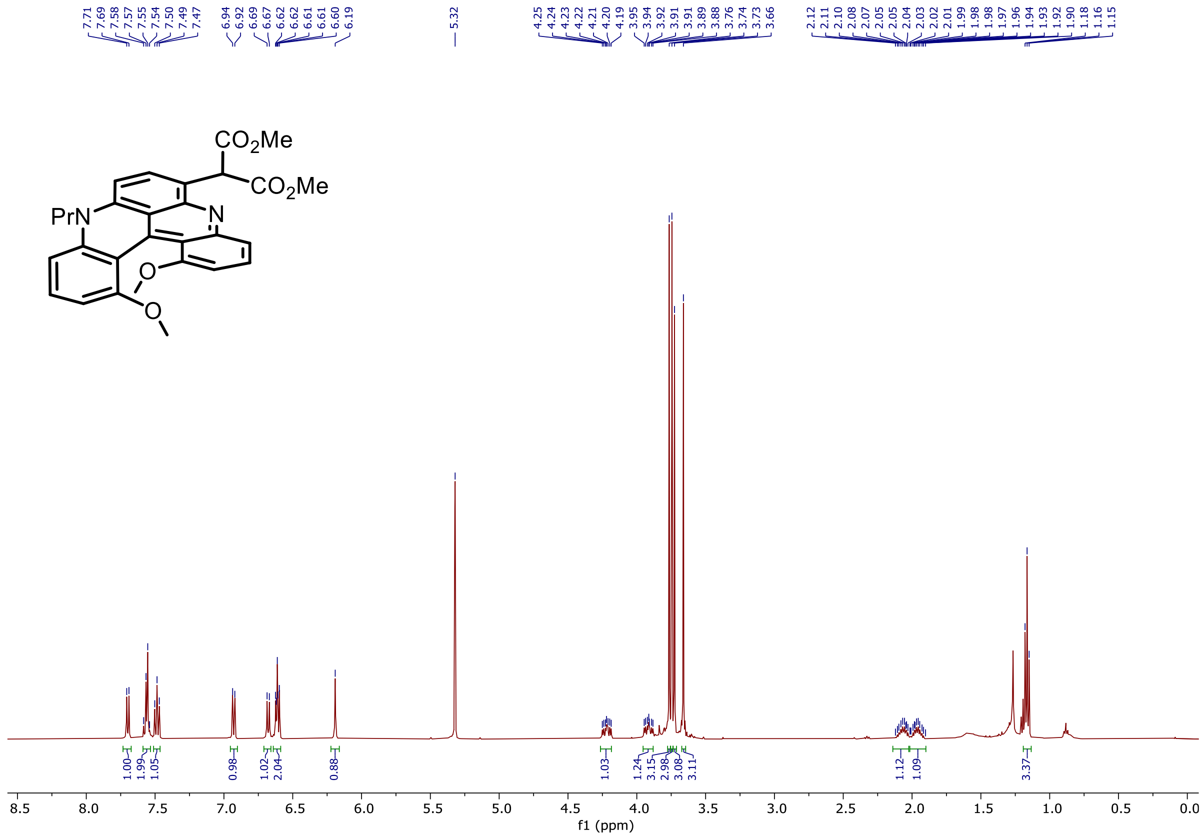

Figure S29. ^{1}H NMR (500 MHz, CD_2Cl_2) spectrum of **3a**.

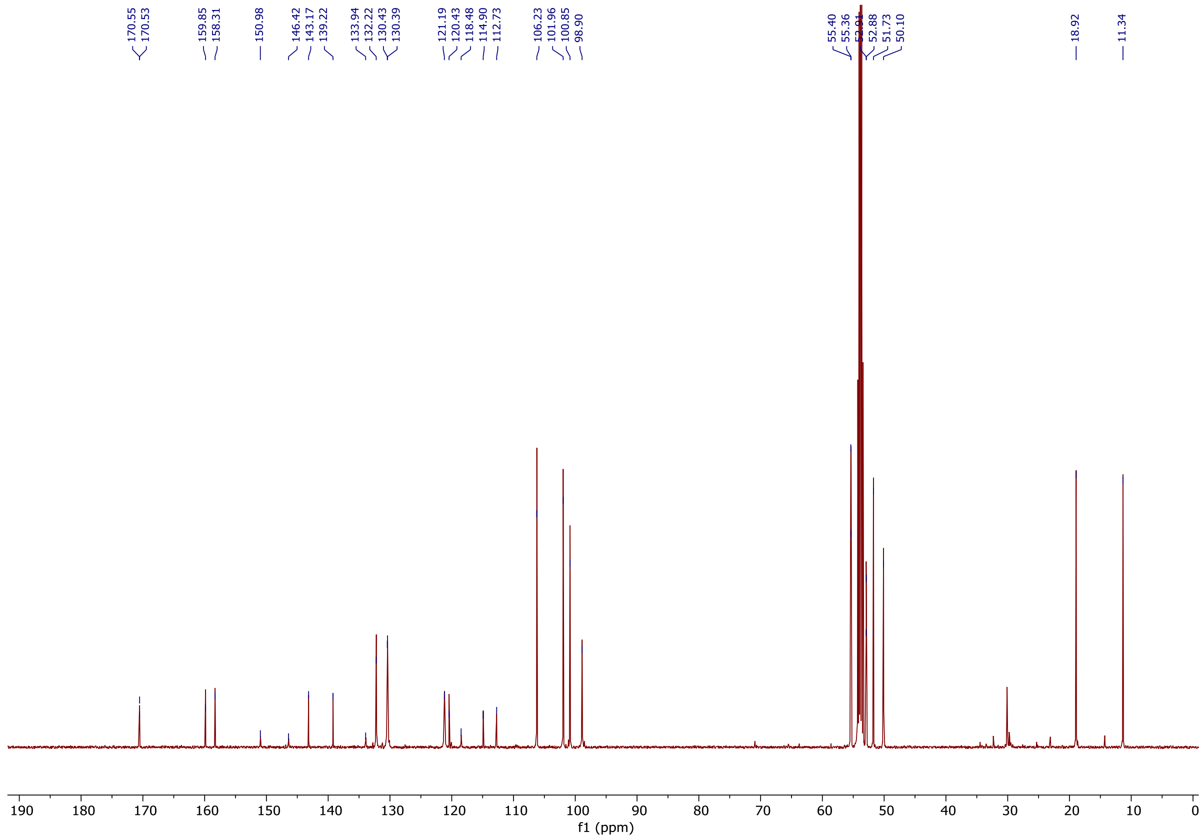

Figure S30. ^{13}C NMR (126 MHz, CD_2Cl_2) spectrum of **3a**.

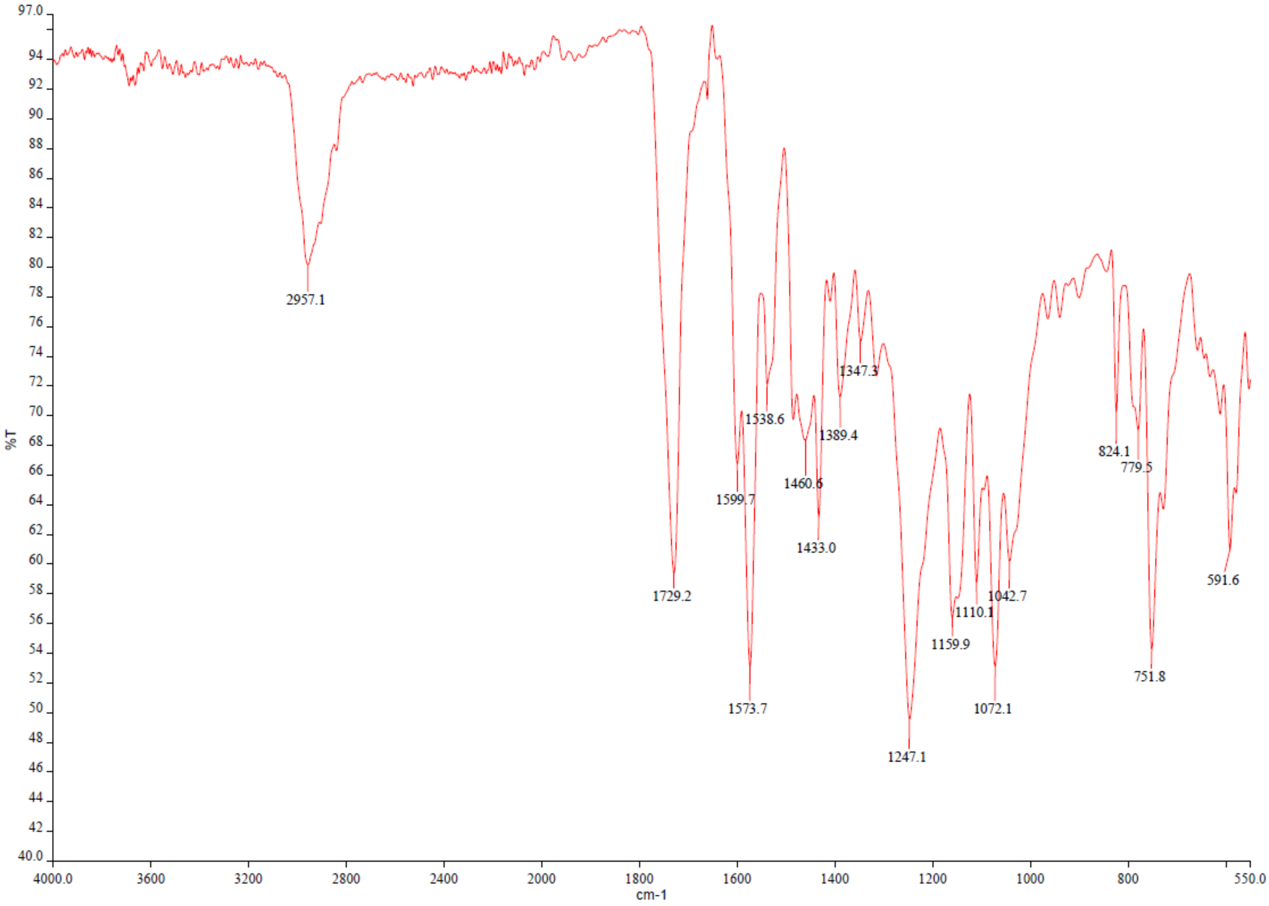

Figure S31. IR (neat) spectrum of **3a**.

Mass Spectrometry Core Facility

Lacour Group – University of Geneva

UNIVERSITÉ DE GENÈVE
FACULTÉ DES SCIENCES

ESI-HRMS – Certificate of Analysis

Applicant:	Yana Nikolova	Date of certificate:	16/04/21
Sample name:	YN-Me-mal	Instrument:	Xevo G2 Tof (TOF)
Folder:	300321.PRO	Mobile phase:	MeOH (100 µl/min)
Analyst:	Stéphane Grass	Ionisation mode:	ESI (positive polarity)

**Elemental Formula**	**Ion type**	***Masslynx values ****** *calc. m/z*	*meas. m/z*	**Calc. *m/z***	**Meas. *m/z***	**Accuracy** [a] *(ppm)*
$C_{31}H_{32}N_2O_6$	$[M+H]^+$	*501.2026*	*501.2021*	501.2021	501.2016	-1.0

[a] *Mass spectrum is calibrated by the use of the MS lockspray system (LeuEnk calibration solution).*

******* *MassLynx software does not take into account the mass of the electron for ionic species, therefore the shift of m/z 0.000459.*

Zoomed mass spectrum – Isotopic distribution.

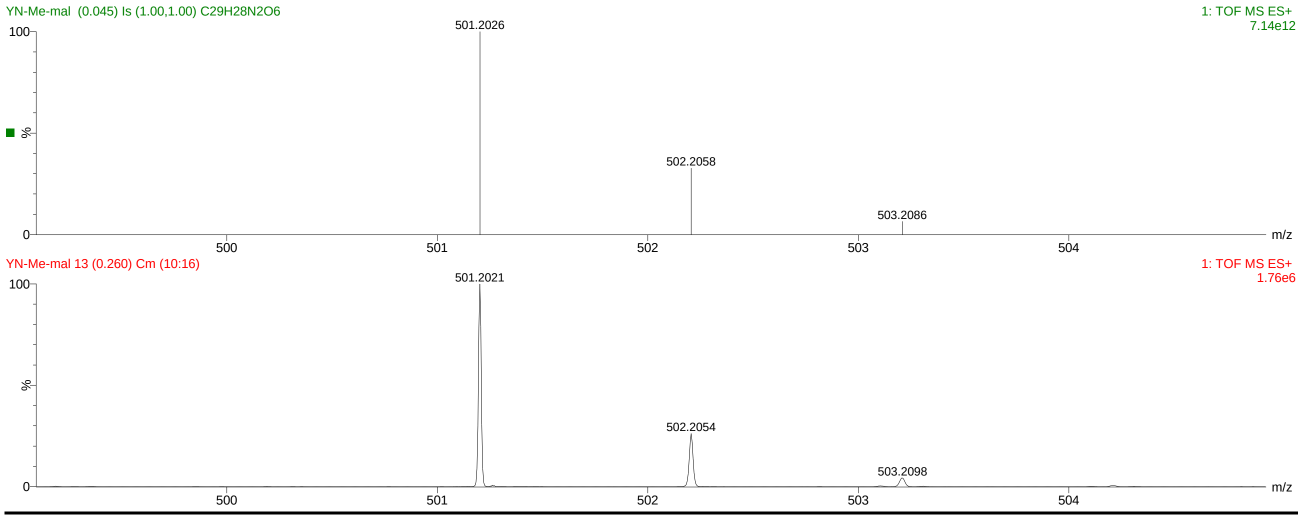

Figure S32. HRMS analysis (ESI, CH_3OH) report of **3a**.

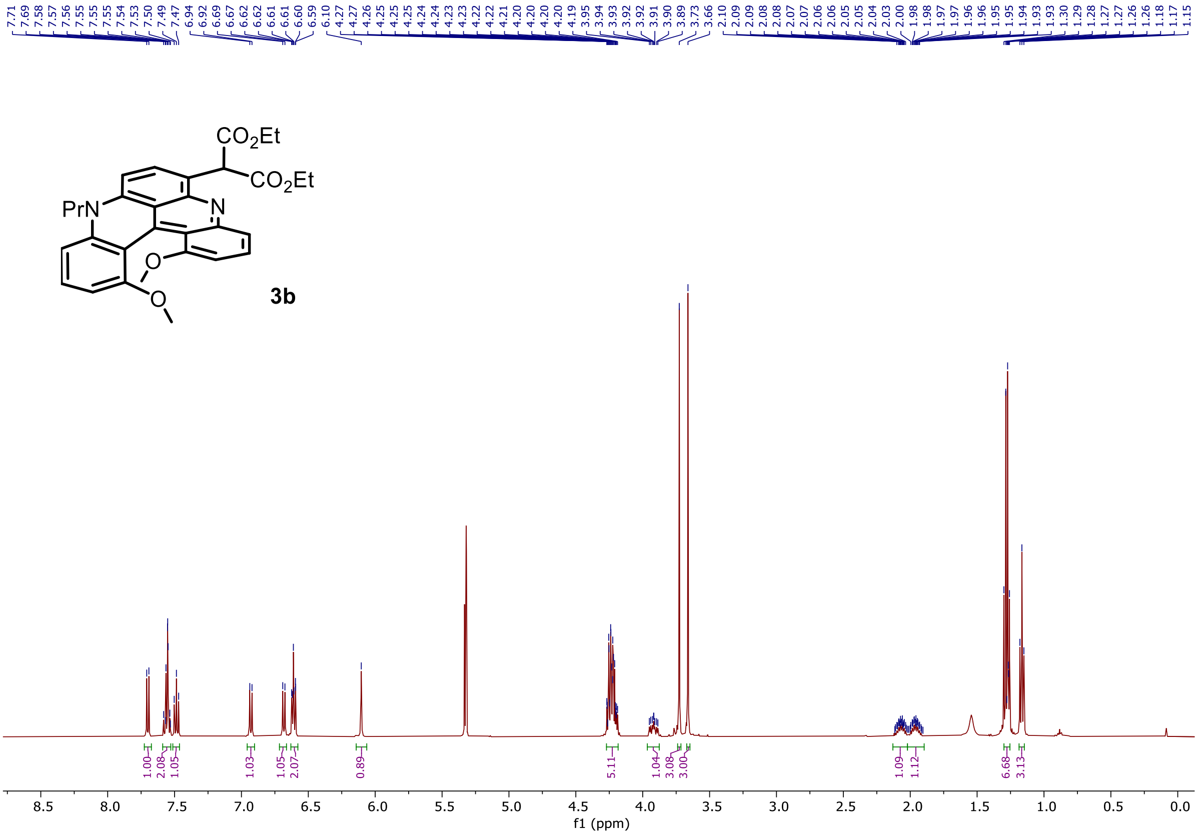

Figure S33. ^{1}H NMR (500 MHz, CD_2Cl_2) spectrum of **3b**.

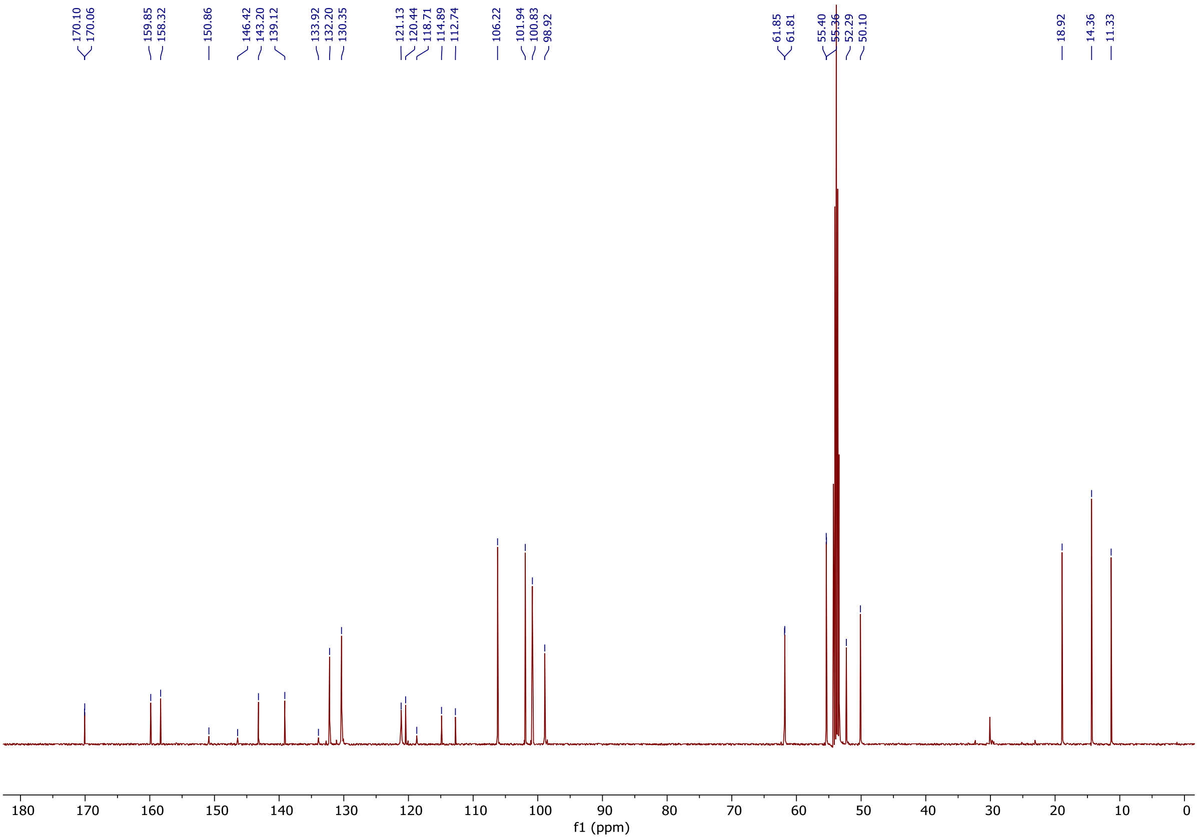

Figure S34. ^{13}C NMR (126 MHz, CD_2Cl_2) spectrum of **3b**.

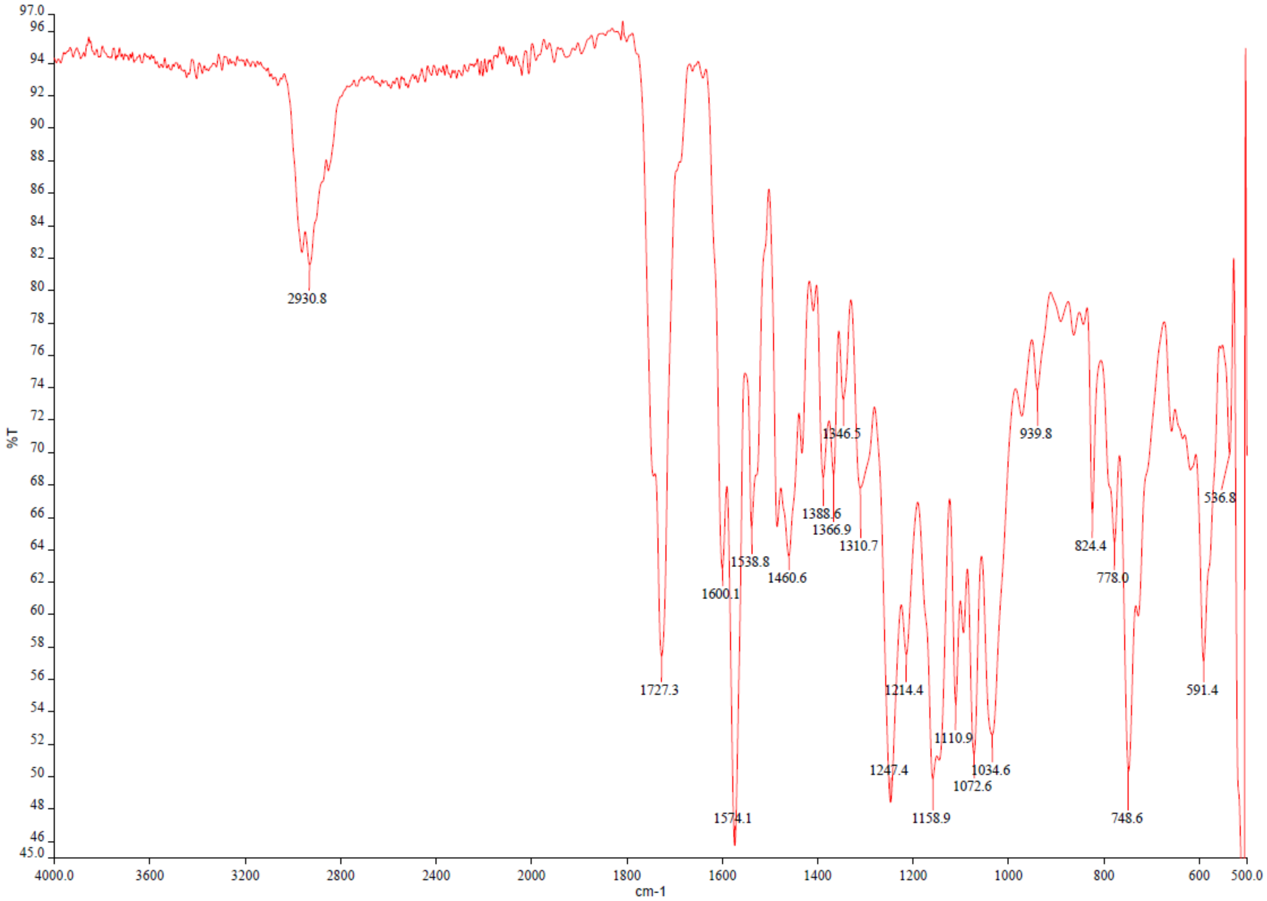

Figure S35. IR (neat) spectrum of **3b**.

Mass Spectrometry Core Facility

Lacour Group – University of Geneva

UNIVERSITÉ DE GENÈVE
FACULTÉ DES SCIENCES

ESI-HRMS – Certificate of Analysis

Applicant:	Yana Nikolova	Date of certificate:	17/05/21
Sample name:	YN-monoEt	Instrument:	Xevo G2 Tof (TOF)
Folder:	170521.PRO	Mobile phase:	MeOH (100 µl/min)
Analyst:	Stéphane Grass	Ionisation mode:	ESI (positive polarity)

Elemental Formula	Ion type	*Masslynx values* *** *calc. m/z*	*meas. m/z*	Calc. *m/z*	Meas. *m/z*	Accuracy [a] *(ppm)*
$C_{31}H_{32}N_2O_6$	$[M+H]^+$	*529.2339*	*529.2337*	529.2334	529.2332	-0.4

[a] *Mass spectrum is calibrated by the use of the MS lockspray system (LeuEnk calibration solution).*

**** MassLynx software does not take into account the mass of the electron for ionic species, therefore the shift of m/z 0.000459.*

Zoomed mass spectrum – Isotopic distribution.

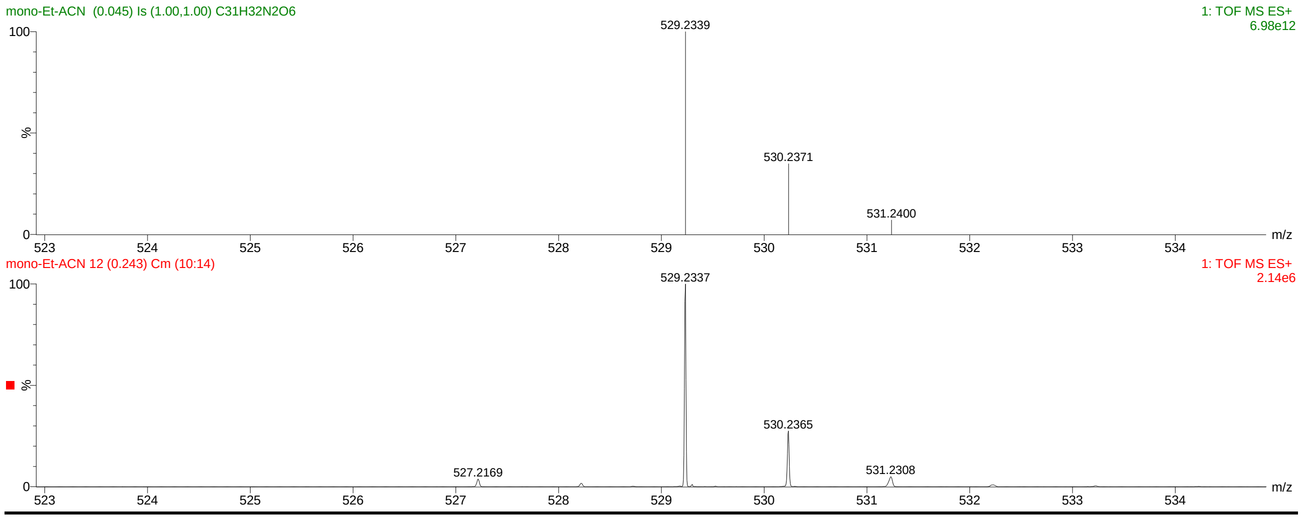

Figure S36. HRMS analysis (ESI, CH_3OH) report of **3b**.

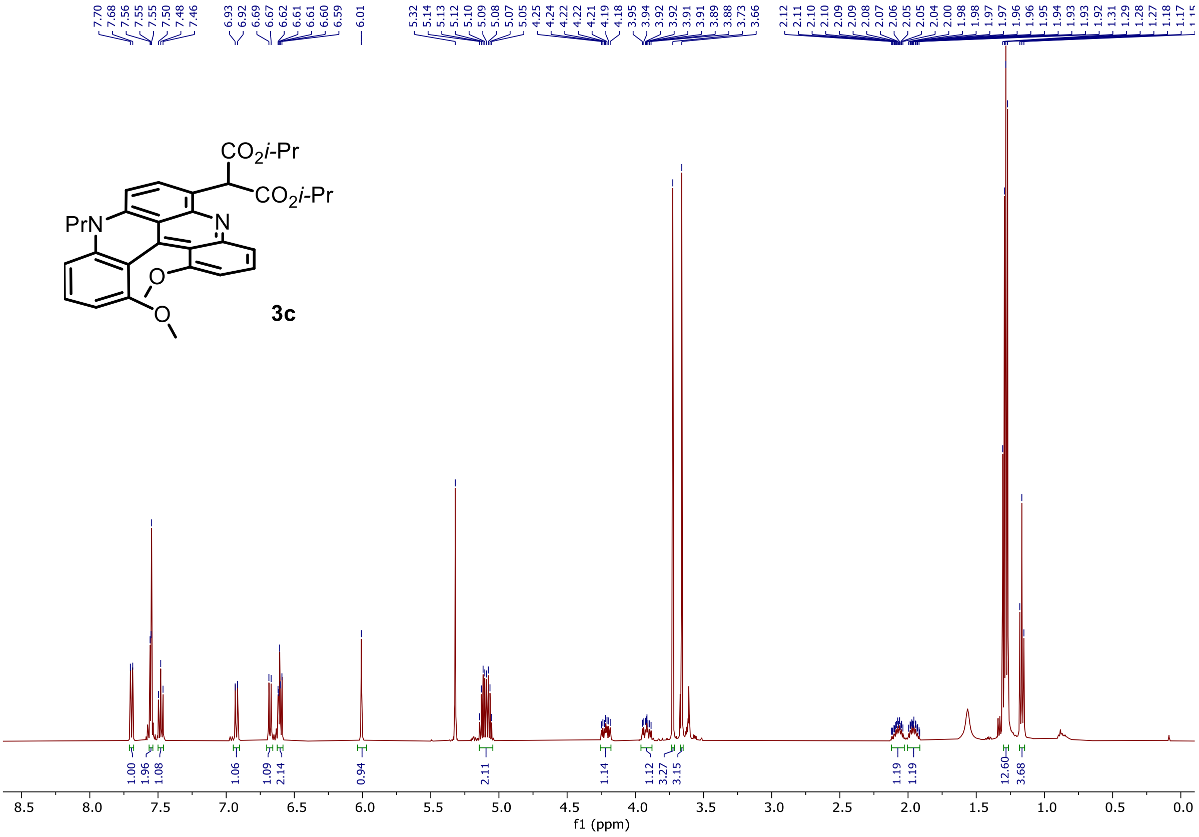

Figure S37. ^{1}H NMR (500 MHz, CD_2Cl_2) spectrum of **3c**.

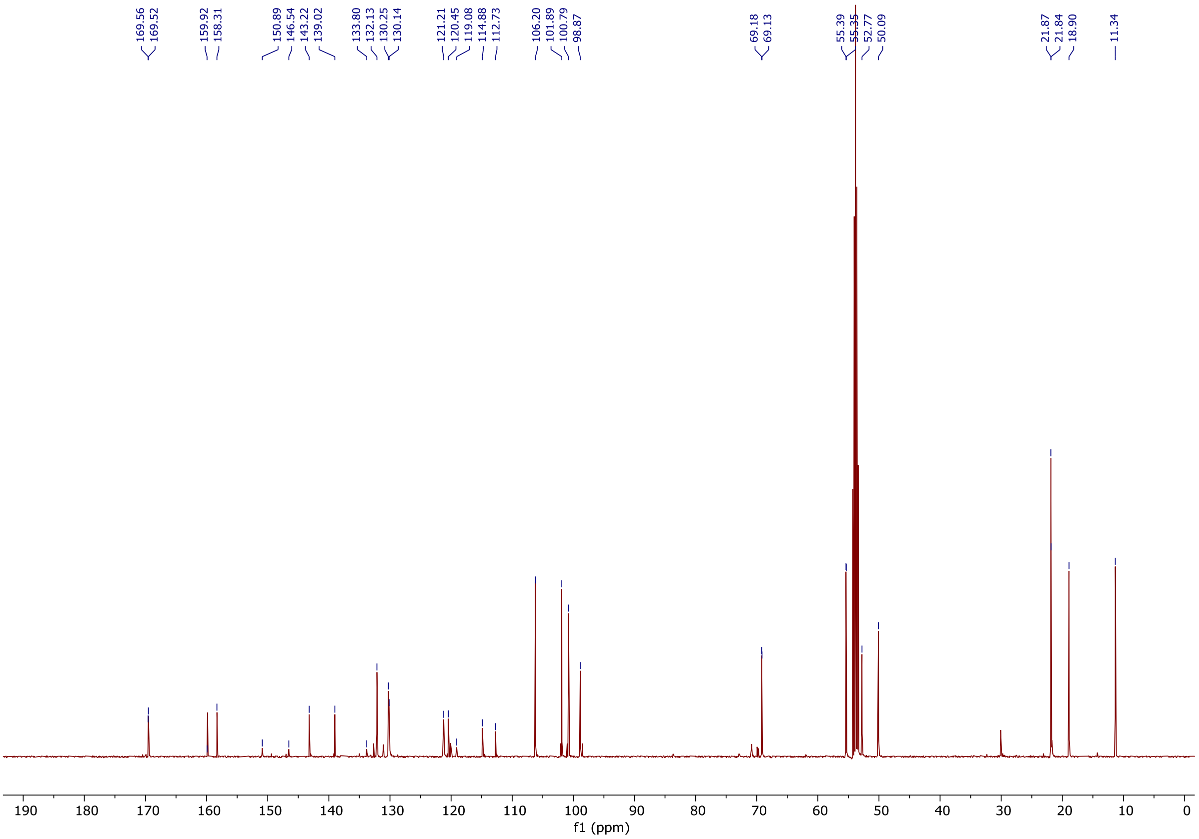

Figure S38. ^{13}C NMR (126 MHz, CD_2Cl_2) spectrum of **3c**.

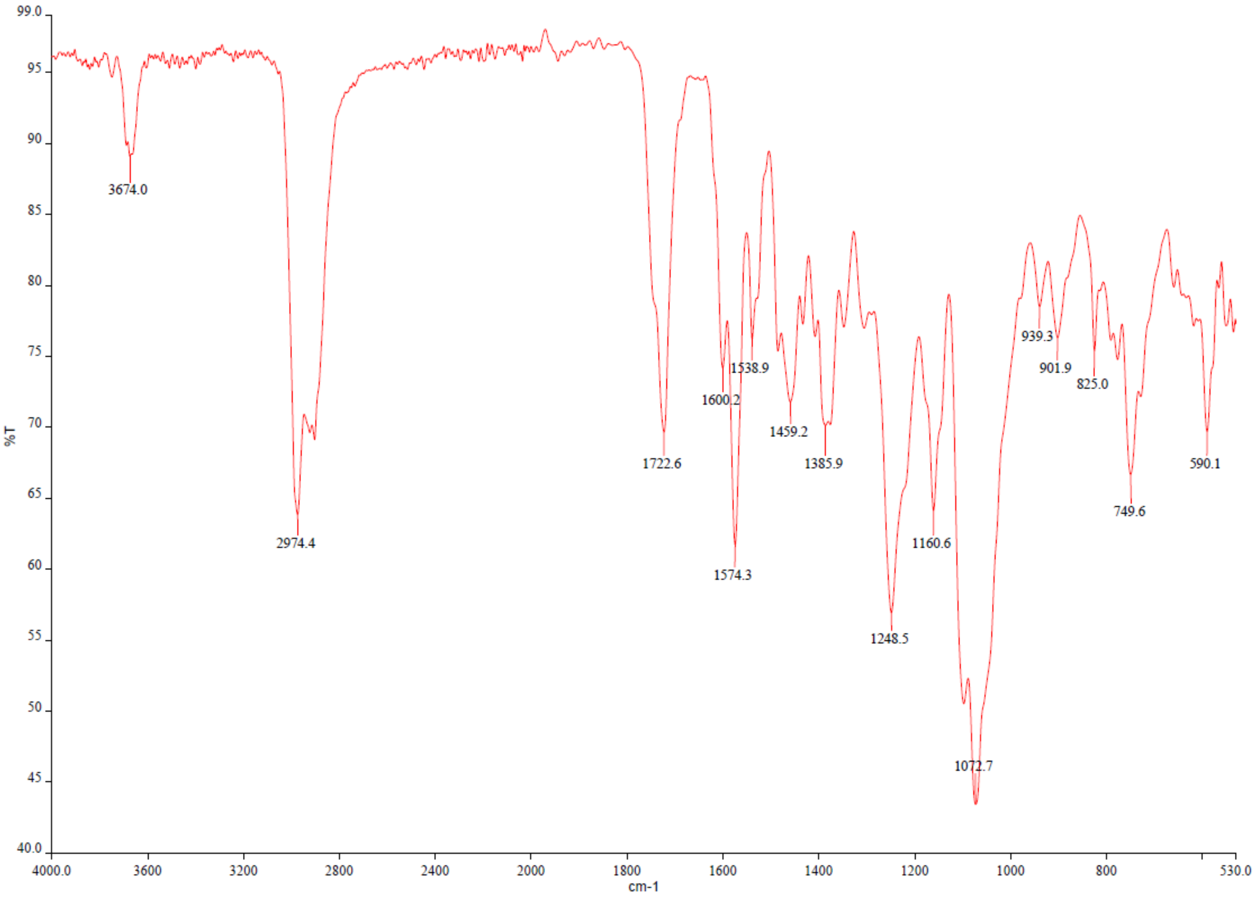

Figure S39. IR (neat) spectrum of **3c**.

Mass Spectrometry Core Facility

Lacour Group – University of Geneva

UNIVERSITÉ DE GENÈVE
FACULTÉ DES SCIENCES

ESI-HRMS – Certificate of Analysis

Applicant:	Yana Nikolova	Date of certificate:	02/06/21
Sample name:	YN-iPr-mal	Instrument:	Xevo G2 Tof (TOF)
Folder:	020621.PRO	Mobile phase:	MeOH (100 µl/min)
Analyst:	Stéphane Grass	Ionisation mode:	ESI (positive polarity)

Elemental Formula	Ion type	*Masslynx values **** *calc. m/z*	*meas. m/z*	Calc. *m/z*	Meas. *m/z*	Accuracy [a] *(ppm)*
$C_{33}H_{36}N_2O_6$	$[M+H]^+$	*557.2665*	*557.2649*	557.2660	557.2644	-2.9

[a] *Mass spectrum is calibrated by the use of the MS lockspray system (LeuEnk calibration solution).*

**** MassLynx software does not take into account the mass of the electron for ionic species, therefore the shift of m/z 0.000459.*

Zoomed mass spectrum – Isotopic distribution.

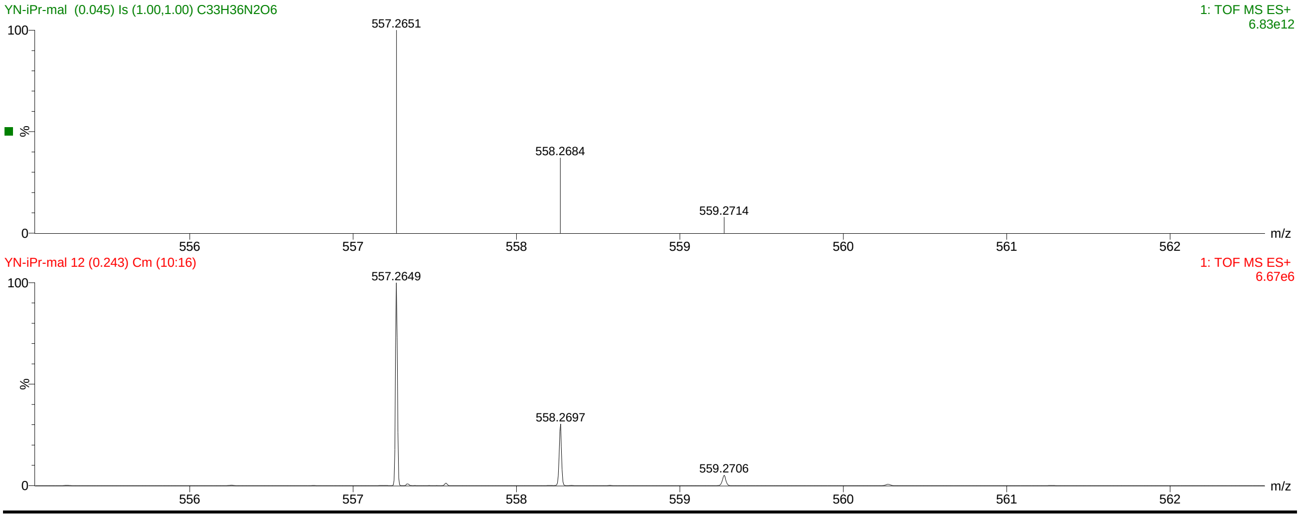

Figure S40. HRMS analysis (ESI, CH_3OH) report of **3c**.

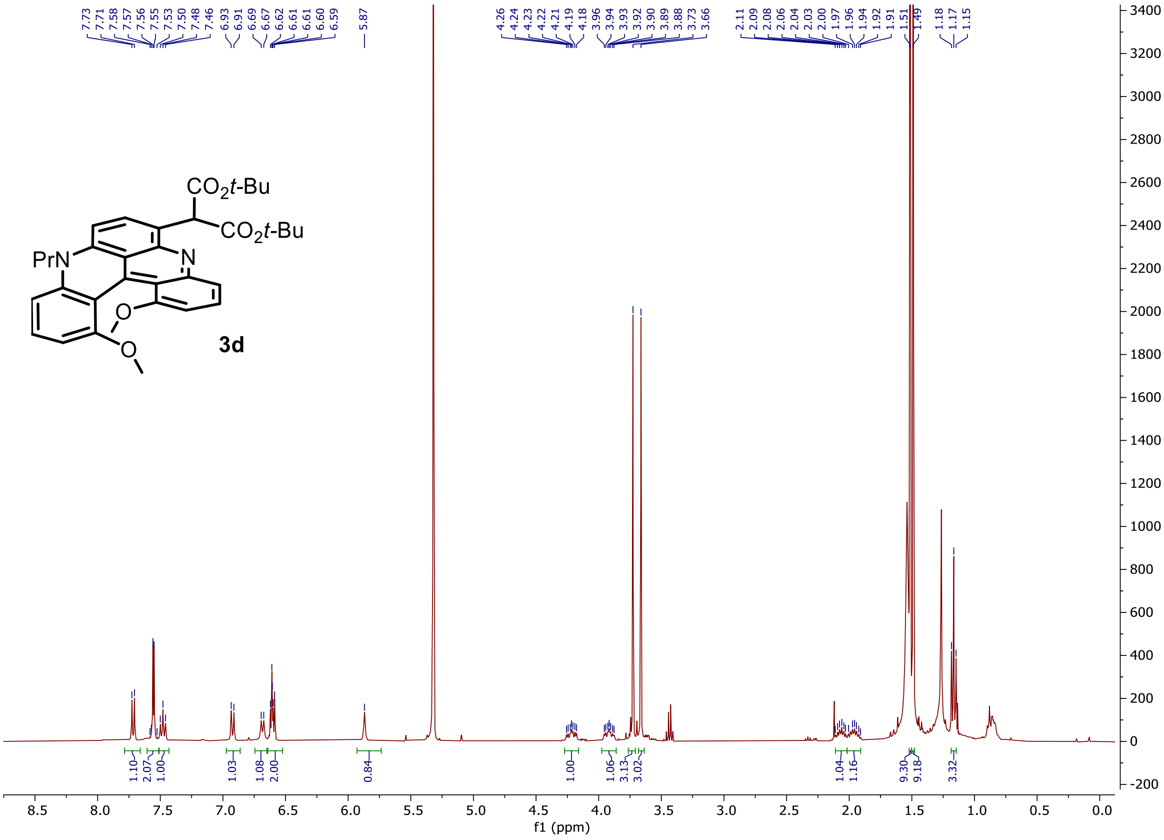

Figure S41. ^{1}H NMR (500 MHz, CD_2Cl_2) spectrum of **3d**.

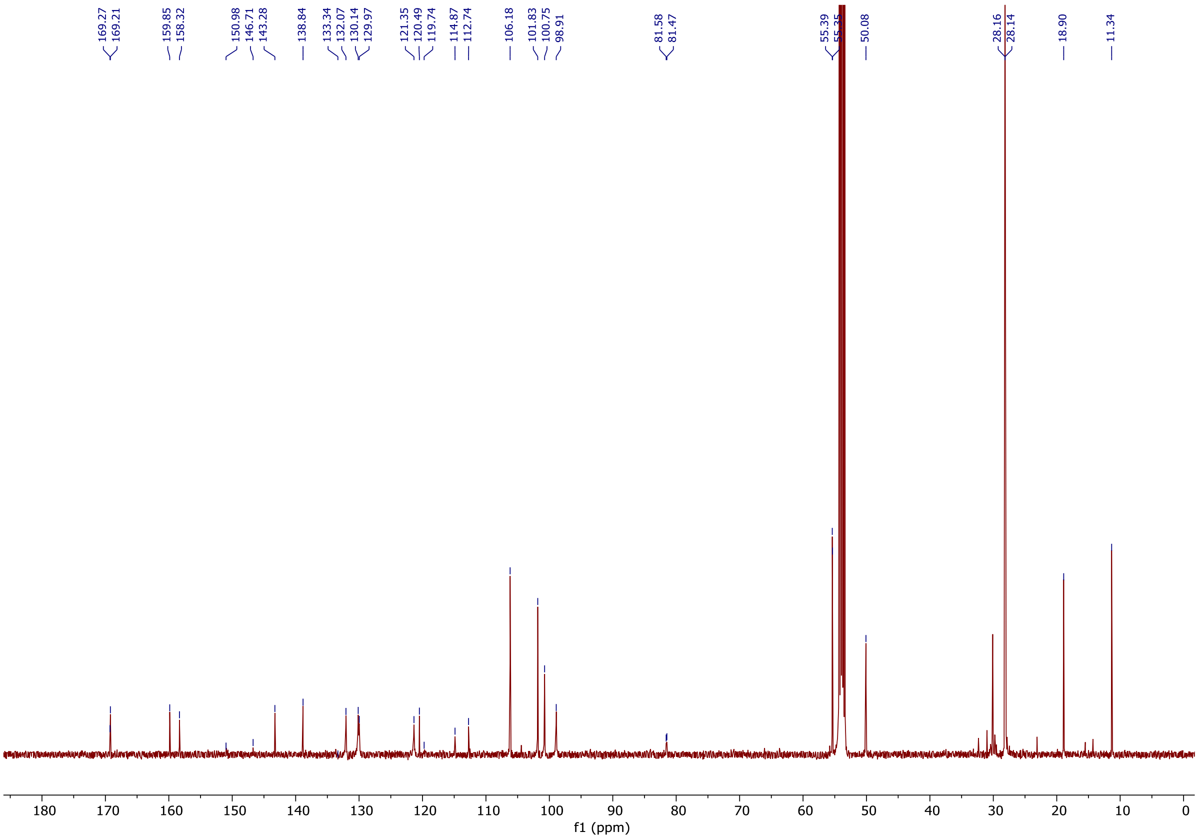

Figure S42. ^{13}C NMR (126 MHz, CD_2Cl_2) spectrum of **3d**.

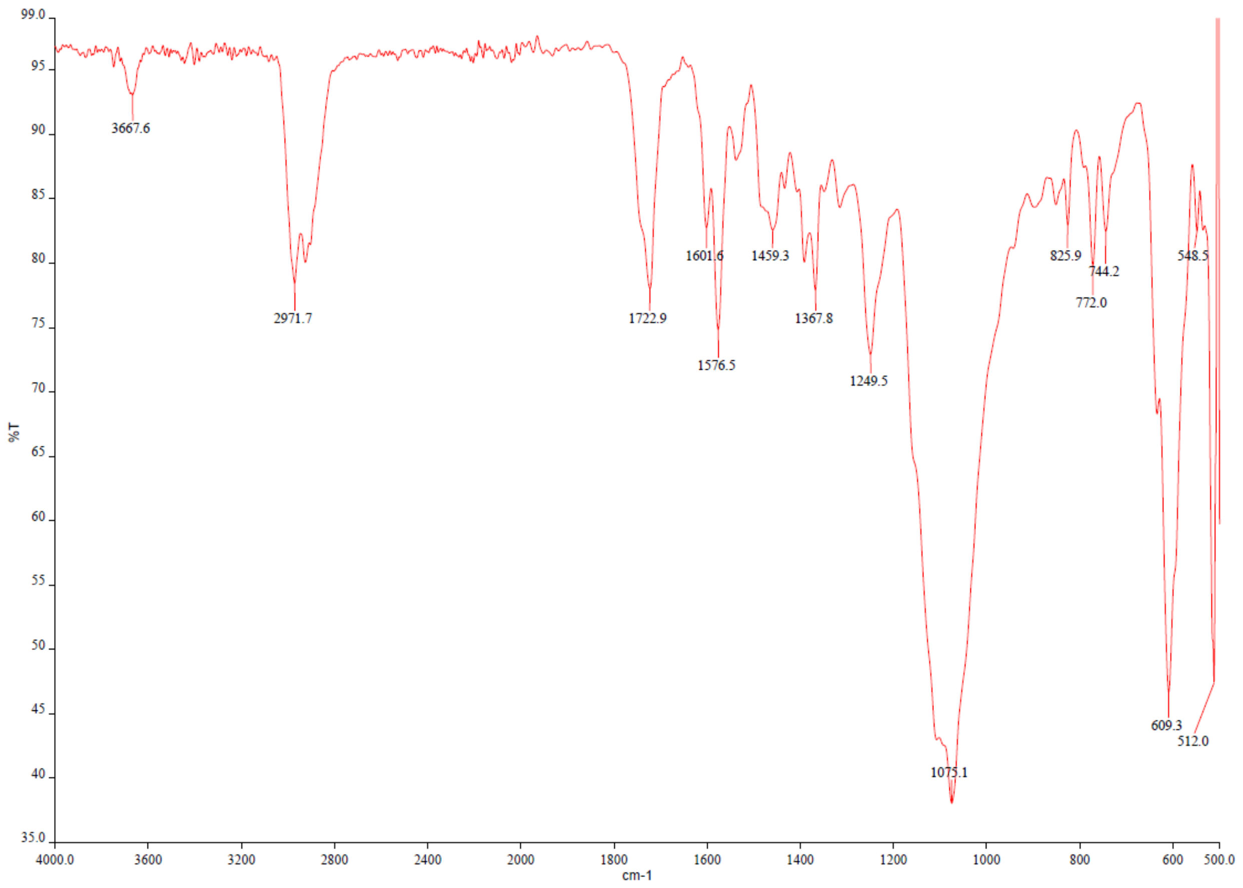

Figure S43. IR (neat) spectrum of **3d**.

Mass Spectrometry Core Facility

Lacour Group – University of Geneva

UNIVERSITÉ DE GENÈVE
FACULTÉ DES SCIENCES

ESI-HRMS – Certificate of Analysis

Applicant:	Yana Nikolova	Date of certificate:	16/04/21
Sample name:	YN-tBu-mal	Instrument:	Xevo G2 Tof (TOF)
Folder:	300321.PRO	Mobile phase:	MeOH (100 µl/min)
Analyst:	Stéphane Grass	Ionisation mode:	ESI (positive polarity)

Elemental Formula	Ion type	*Masslynx values **** *calc. m/z*	*meas. m/z*	Calc. *m/z*	Meas. *m/z*	Accuracy [a] *(ppm)*
$C_{35}H_{40}N_2O_6$	$[M+H]^+$	*585.2964*	*585.2967*	585.2959	585.2962	0.5

[a] *Mass spectrum is calibrated by the use of the MS lockspray system (LeuEnk calibration solution).*

**** MassLynx software does not take into account the mass of the electron for ionic species, therefore the shift of m/z 0.000459.*

Zoomed mass spectrum – Isotopic distribution.

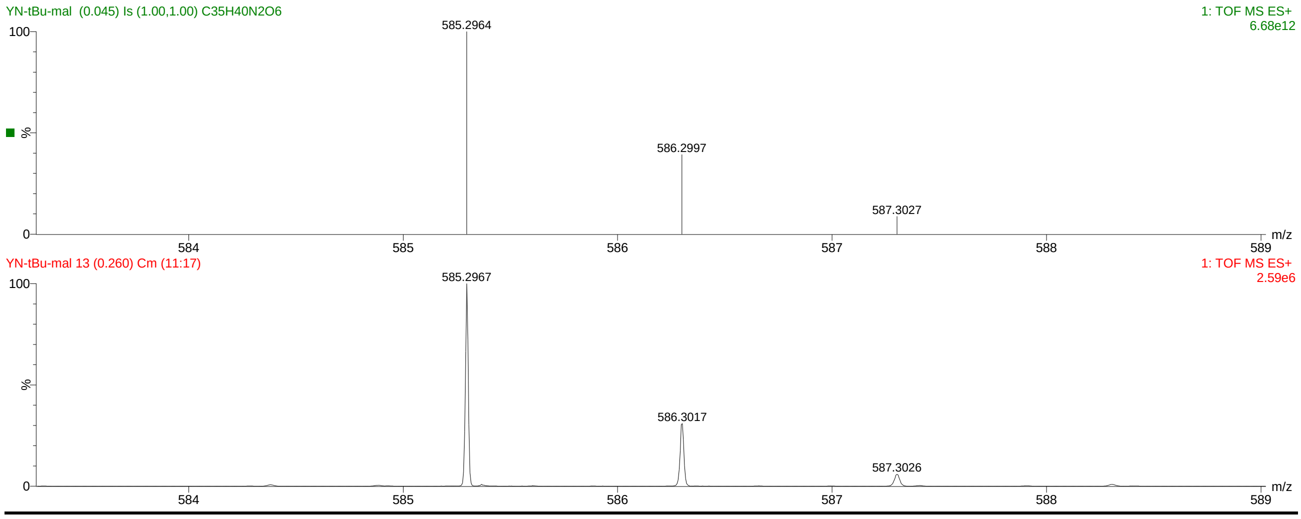

Figure S44. HRMS analysis (ESI, CH_3OH) report of **3d**.

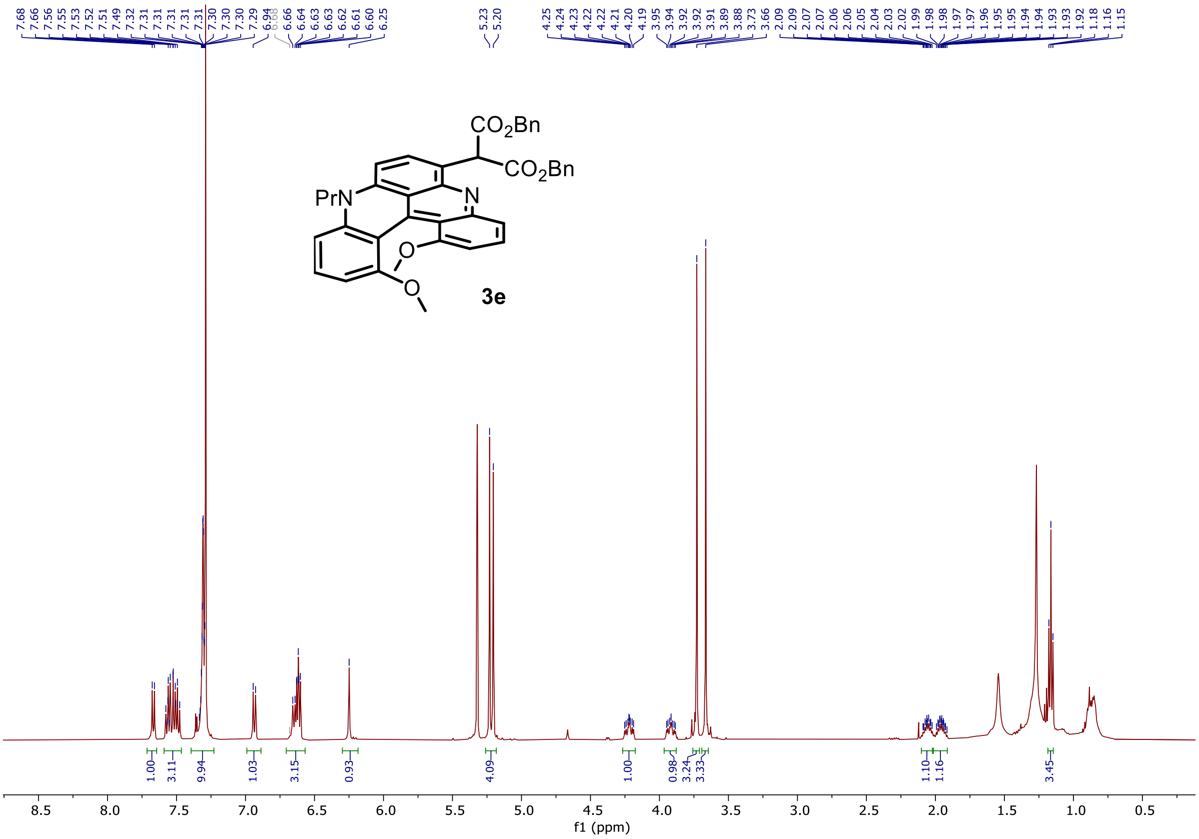

Figure S45. ^{1}H NMR (500 MHz, CD_2Cl_2) spectrum of **3e**.

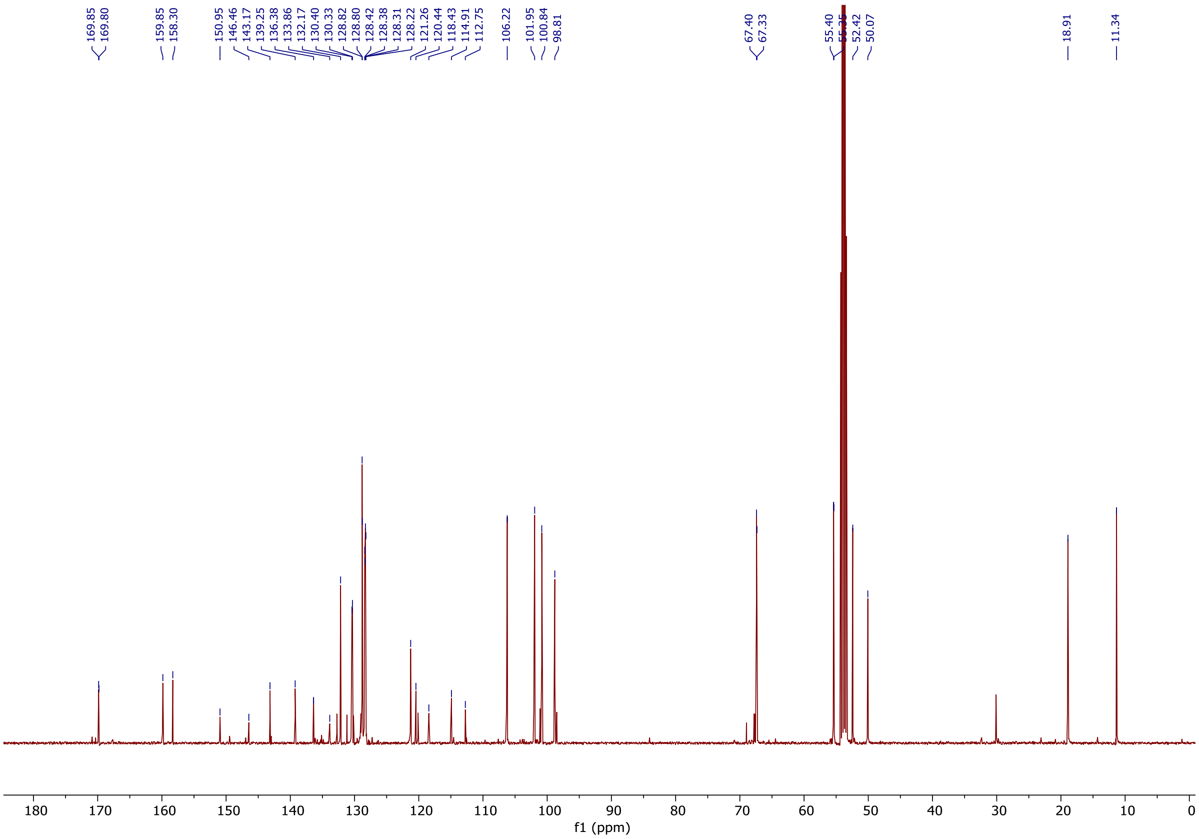

Figure S46. ^{13}C NMR (126 MHz, CD_2Cl_2) spectrum of **3e**.

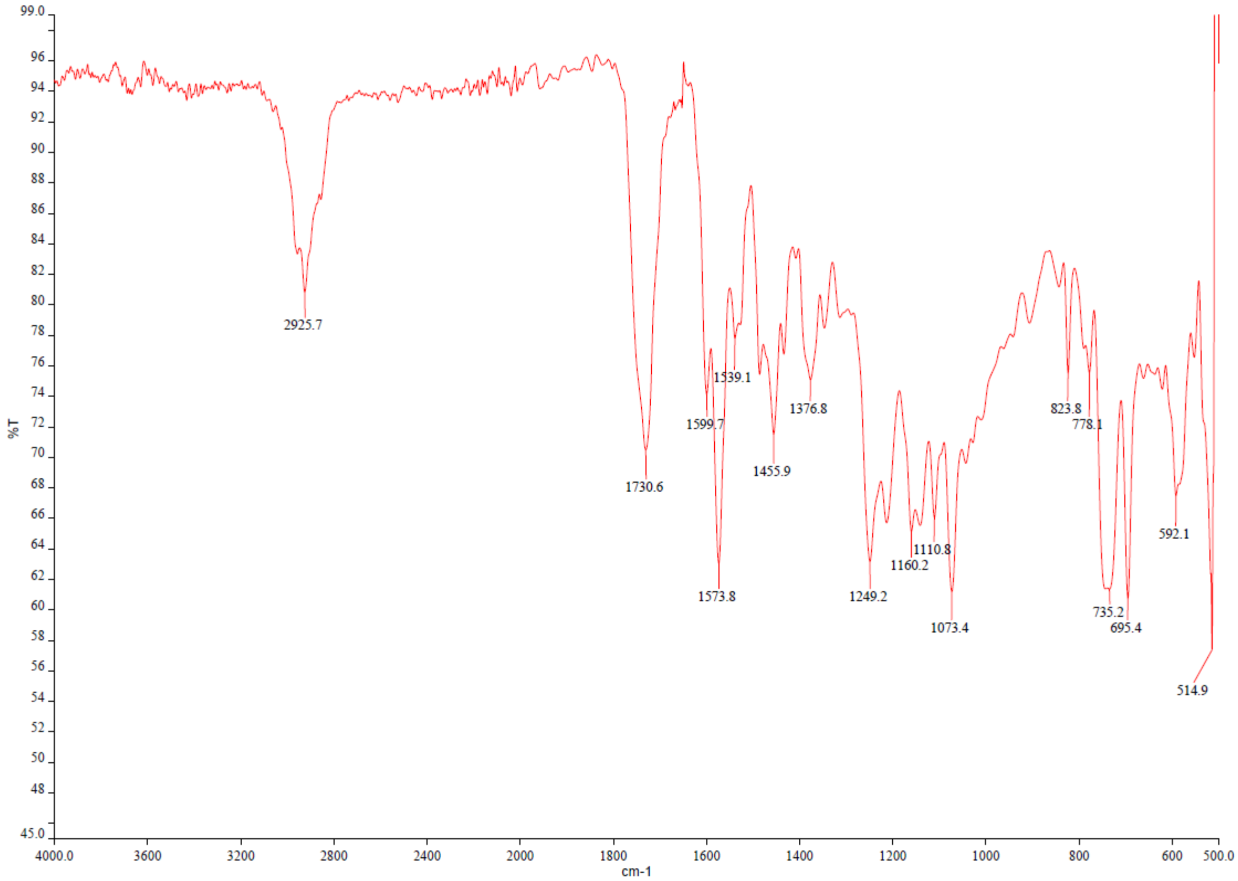

Figure S47. IR (neat) spectrum of **3e**.

Mass Spectrometry Core Facility

Lacour Group – University of Geneva

UNIVERSITÉ DE GENÈVE
FACULTÉ DES SCIENCES

ESI-HRMS – Certificate of Analysis

Applicant:	Yana Nikolova	Date of certificate:	16/04/21
Sample name:	YN-Bn-mal	Instrument:	Xevo G2 Tof (TOF)
Folder:	300321.PRO	Mobile phase:	MeOH (100 µl/min)
Analyst:	Stéphane Grass	Ionisation mode:	ESI (positive polarity)

Elemental Formula	Ion type	*Masslynx values **** *calc. m/z*	*meas. m/z*	Calc. *m/z*	Meas. *m/z*	Accuracy [a] *(ppm)*
$C_{41}H_{36}N_2O_6$	$[M+H]^+$	*653.2651*	*653.2675*	653.2646	653.2670	3.7

[a] *Mass spectrum is calibrated by the use of the MS lockspray system (LeuEnk calibration solution).*

**** MassLynx software does not take into account the mass of the electron for ionic species, therefore the shift of m/z 0.000459.*

Zoomed mass spectrum – Isotopic distribution.

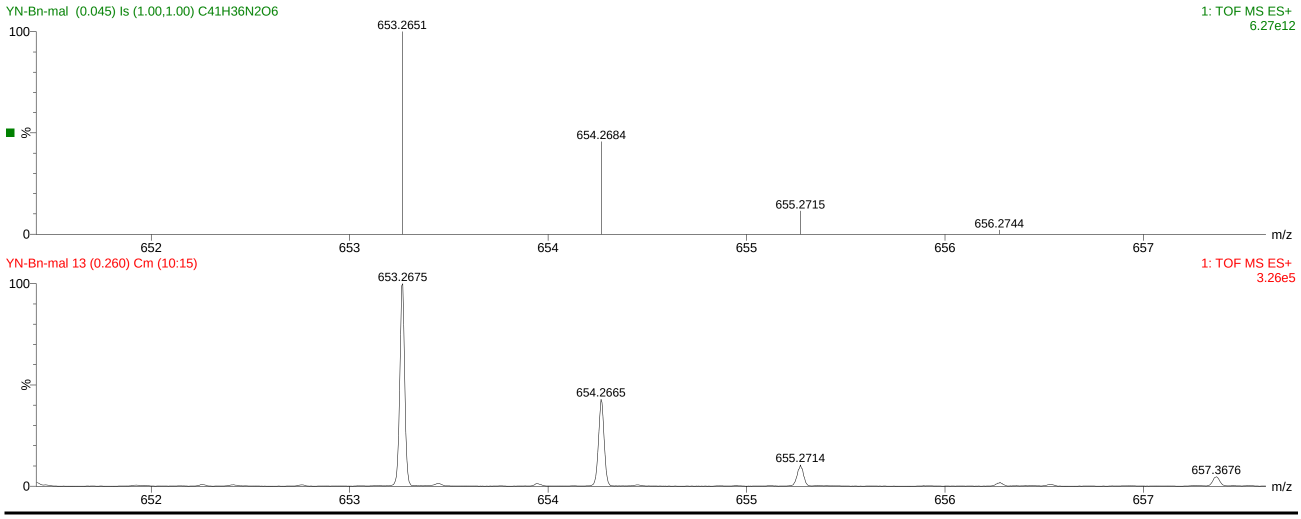

Figure S48. HRMS analysis (ESI, CH_3OH) report of **3e**.

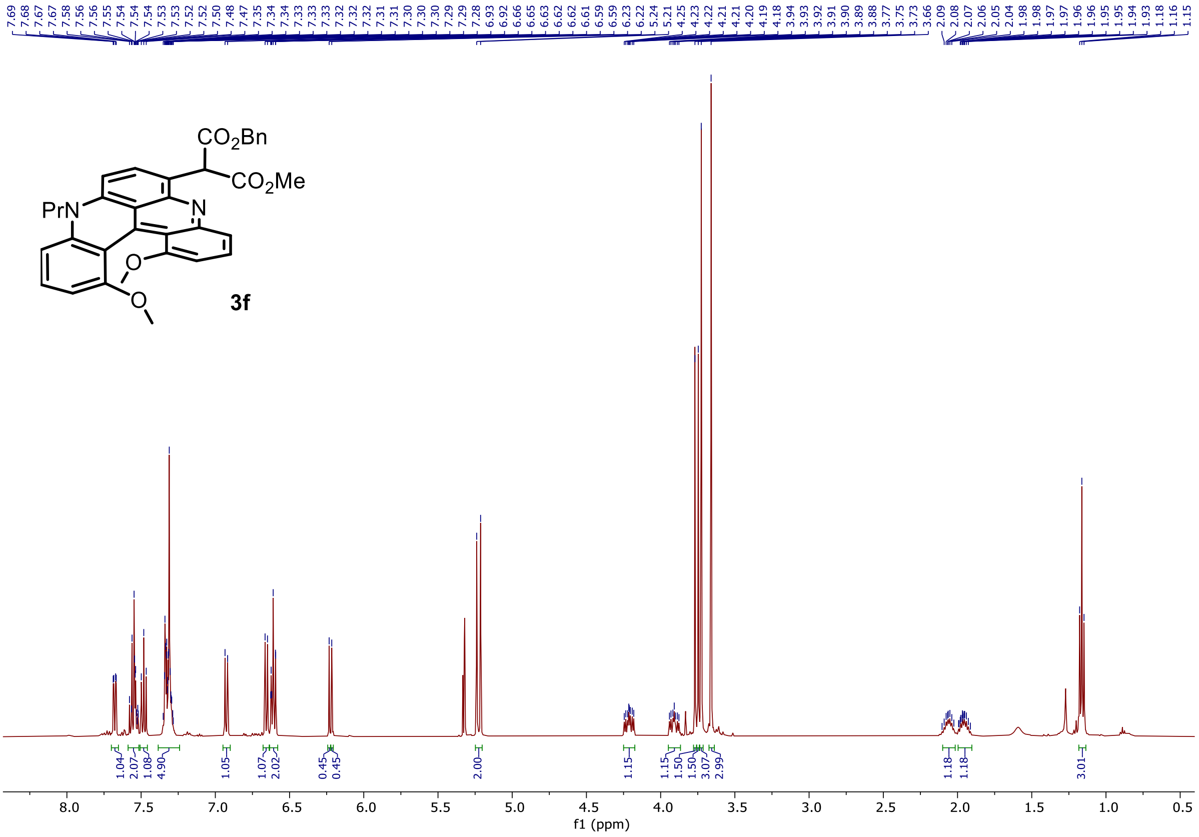

Figure S49. ^{1}H NMR (500 MHz, CD_2Cl_2) spectrum of **3f**.

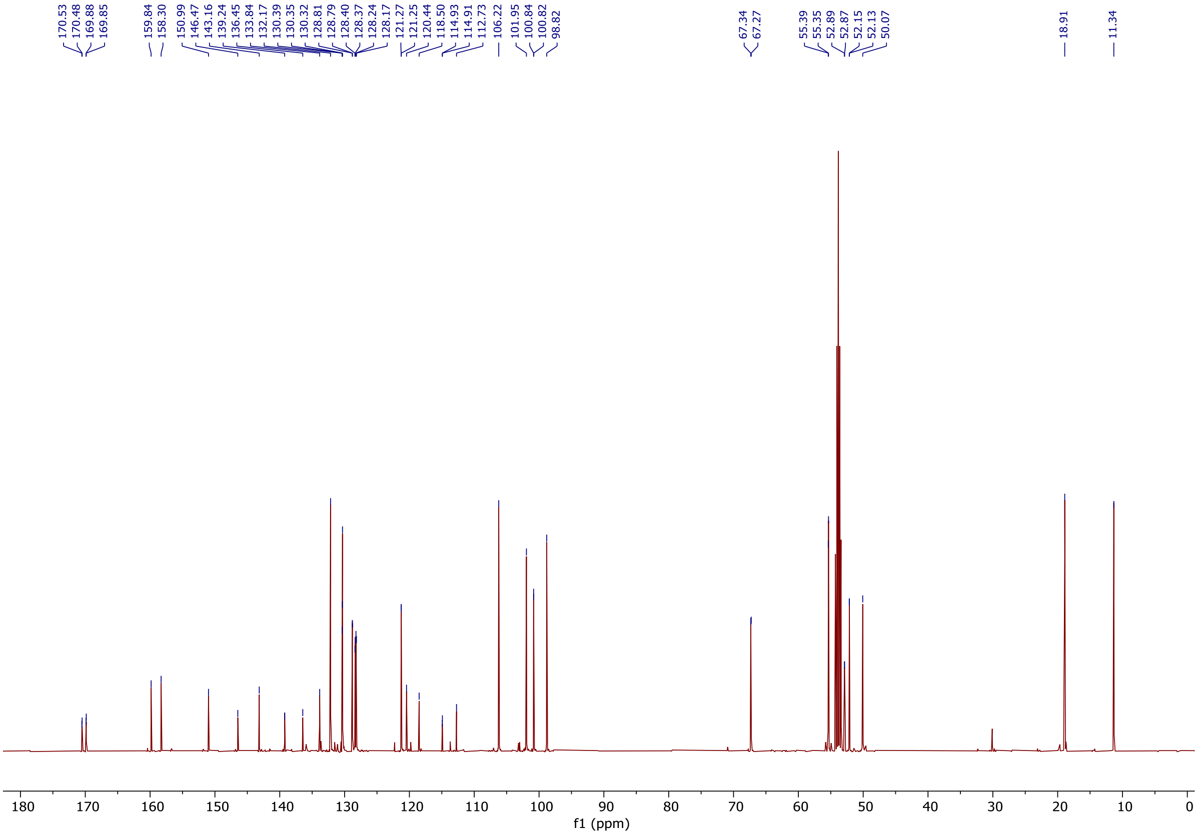

Figure S50. ^{13}C NMR (126 MHz, CD_2Cl_2) spectrum of **3f**.

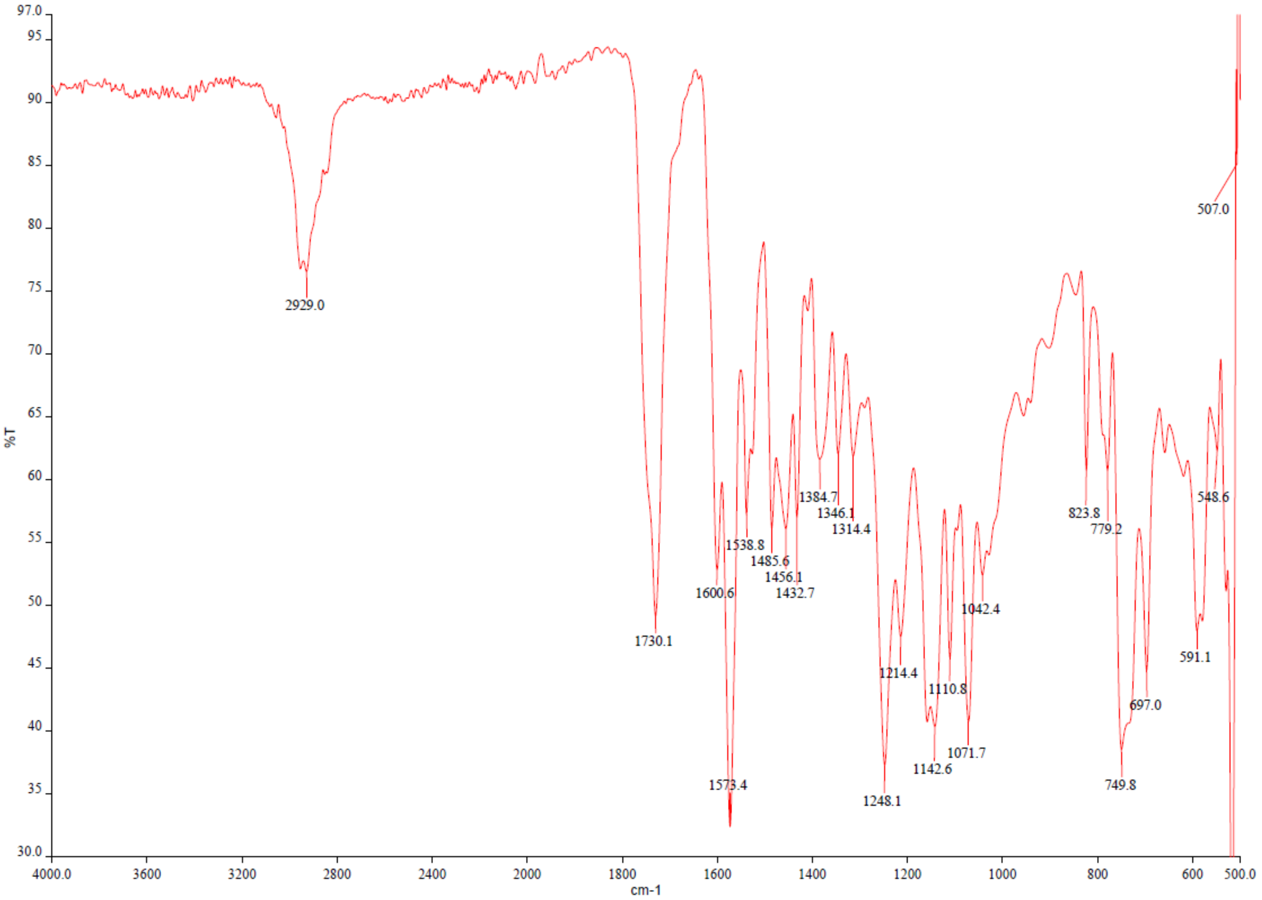

Figure S51. IR (neat) spectrum of **3f**.

Mass Spectrometry Core Facility

Lacour Group – University of Geneva

UNIVERSITÉ DE GENÈVE
FACULTÉ DES SCIENCES

ESI-HRMS – Certificate of Analysis

Applicant:	Yana Nikolova	Date of certificate:	16/04/21
Sample name:	YN-BnMe-mal	Instrument:	Xevo G2 Tof (TOF)
Folder:	300321.PRO	Mobile phase:	MeOH (100 µl/min)
Analyst:	Stéphane Grass	Ionisation mode:	ESI (positive polarity)

Elemental Formula	Ion type	*Masslynx values **** *calc. m/z*	*meas. m/z*	Calc. *m/z*	Meas. *m/z*	Accuracy [a] *(ppm)*
$C_{35}H_{32}N_2O_6$	$[M+H]^+$	*577.2339*	*577.2335*	577.2334	577.2330	-0.7

[a] *Mass spectrum is calibrated by the use of the MS lockspray system (LeuEnk calibration solution).*

**** MassLynx software does not take into account the mass of the electron for ionic species, therefore the shift of m/z 0.000459.*

Zoomed mass spectrum – Isotopic distribution.

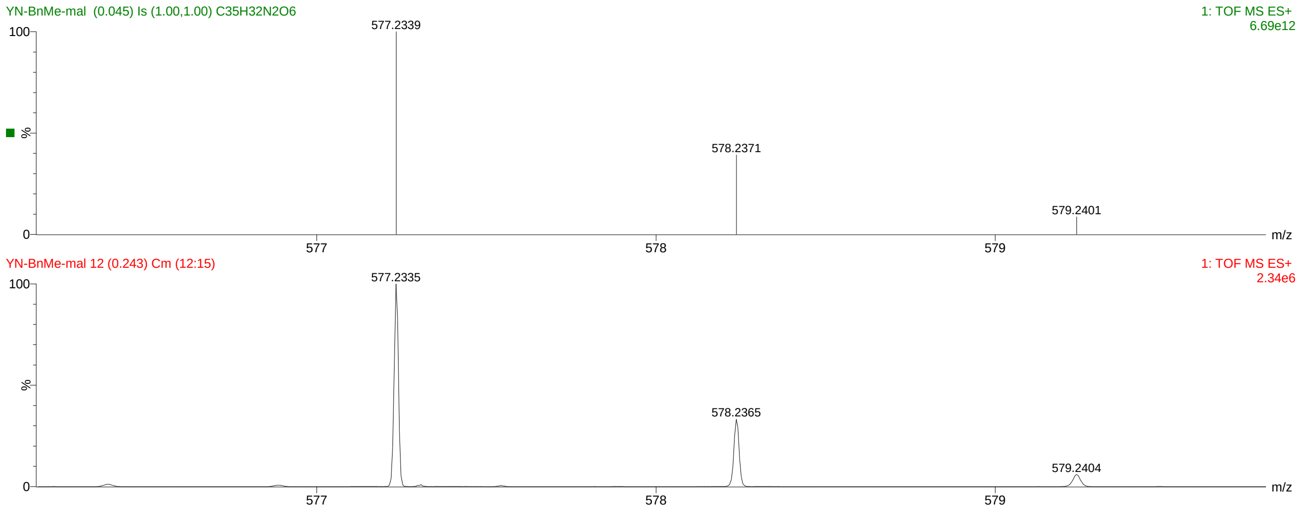

Figure S52. HRMS analysis (ESI, CH_3OH) report of **3f**.

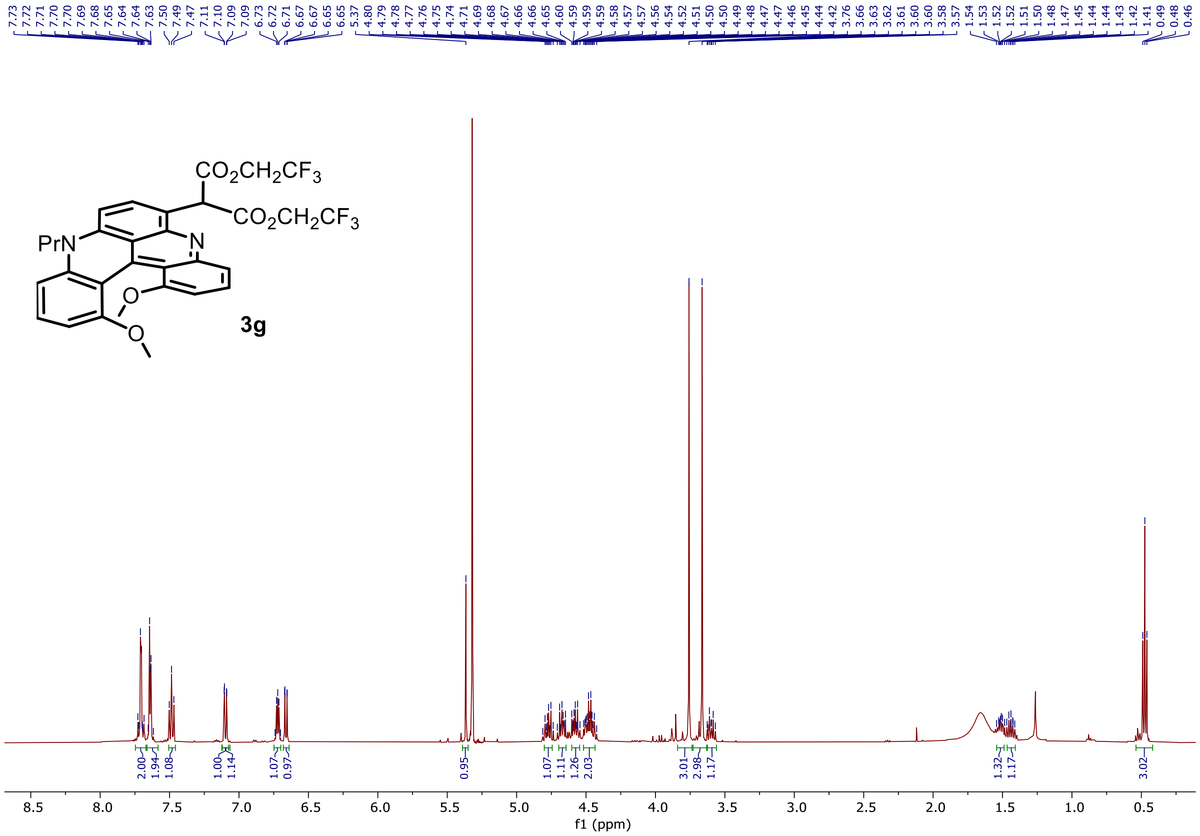

Figure S53. ^{1}H NMR (500 MHz, CD_2Cl_2) spectrum of **3g**.

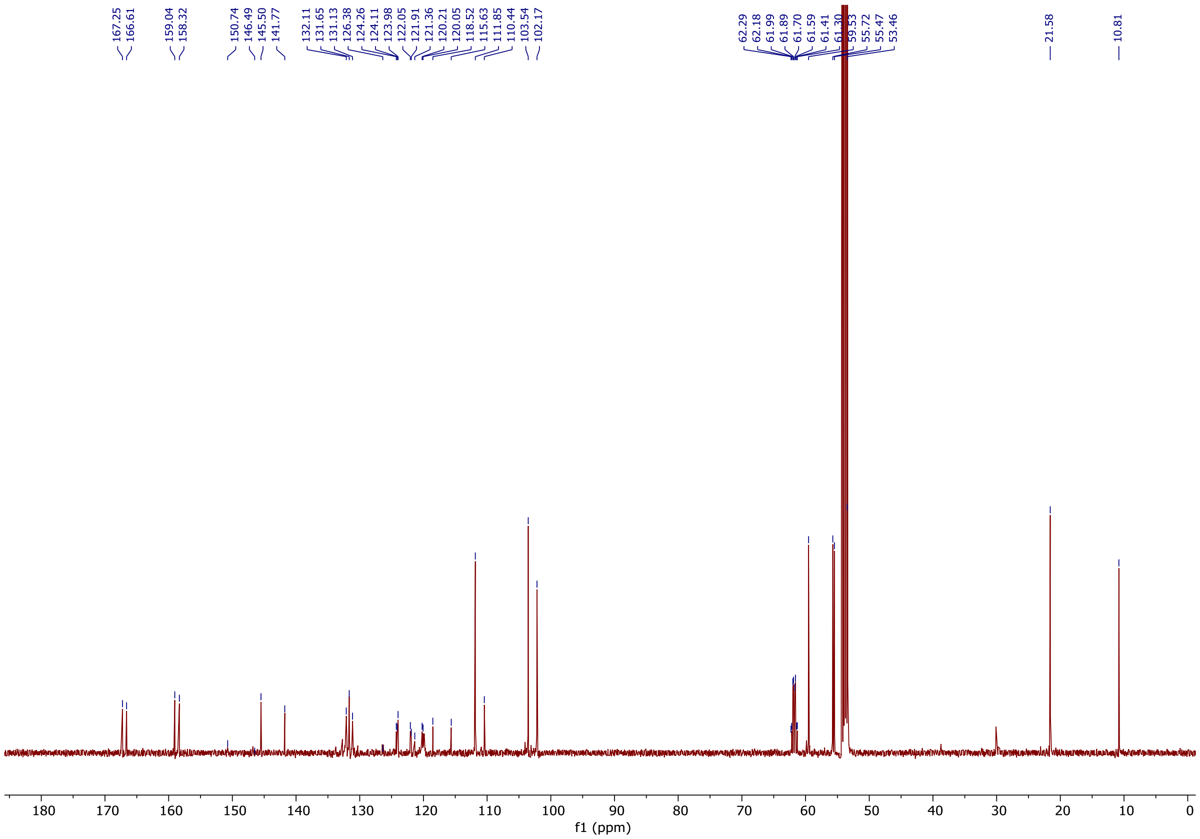

Figure S54. ^{13}C NMR (126 MHz, CD_2Cl_2) spectrum of **3g**.

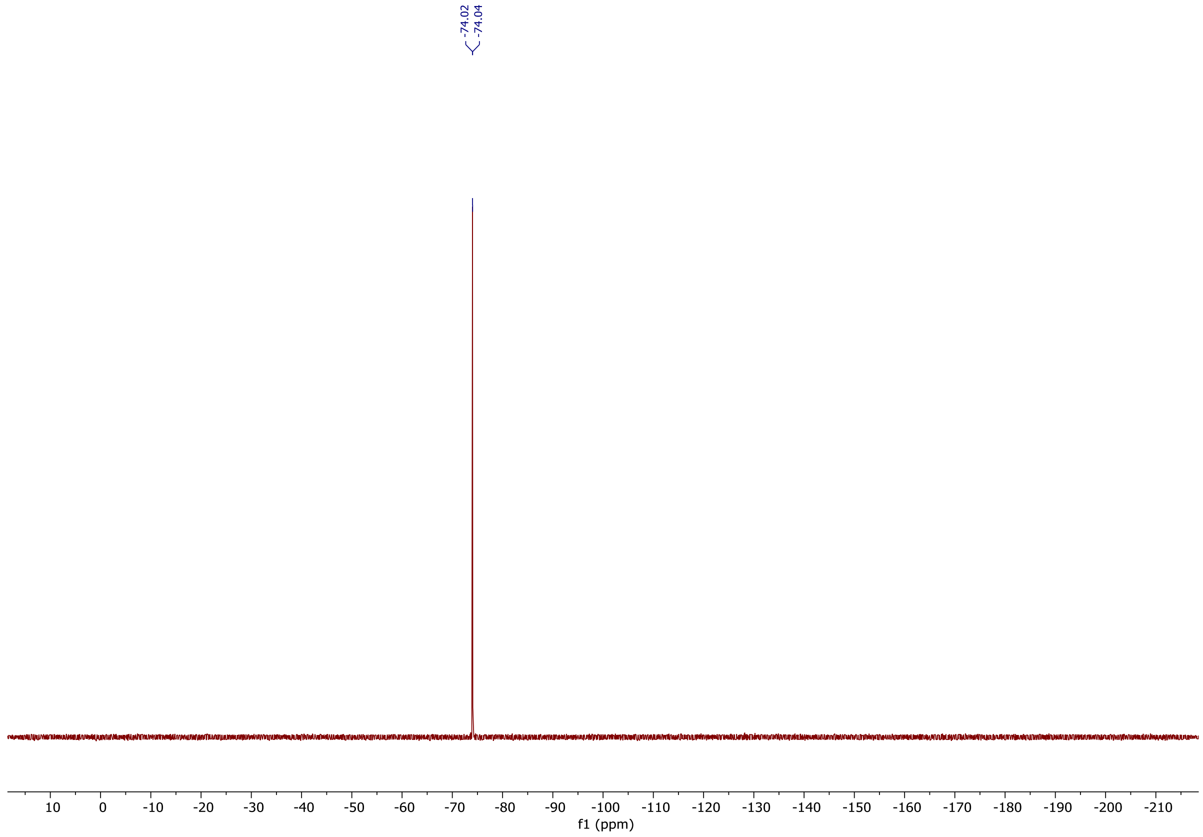

Figure S55. ^{19}F NMR (282 MHz, CD_2Cl_2) spectrum of **3g**.

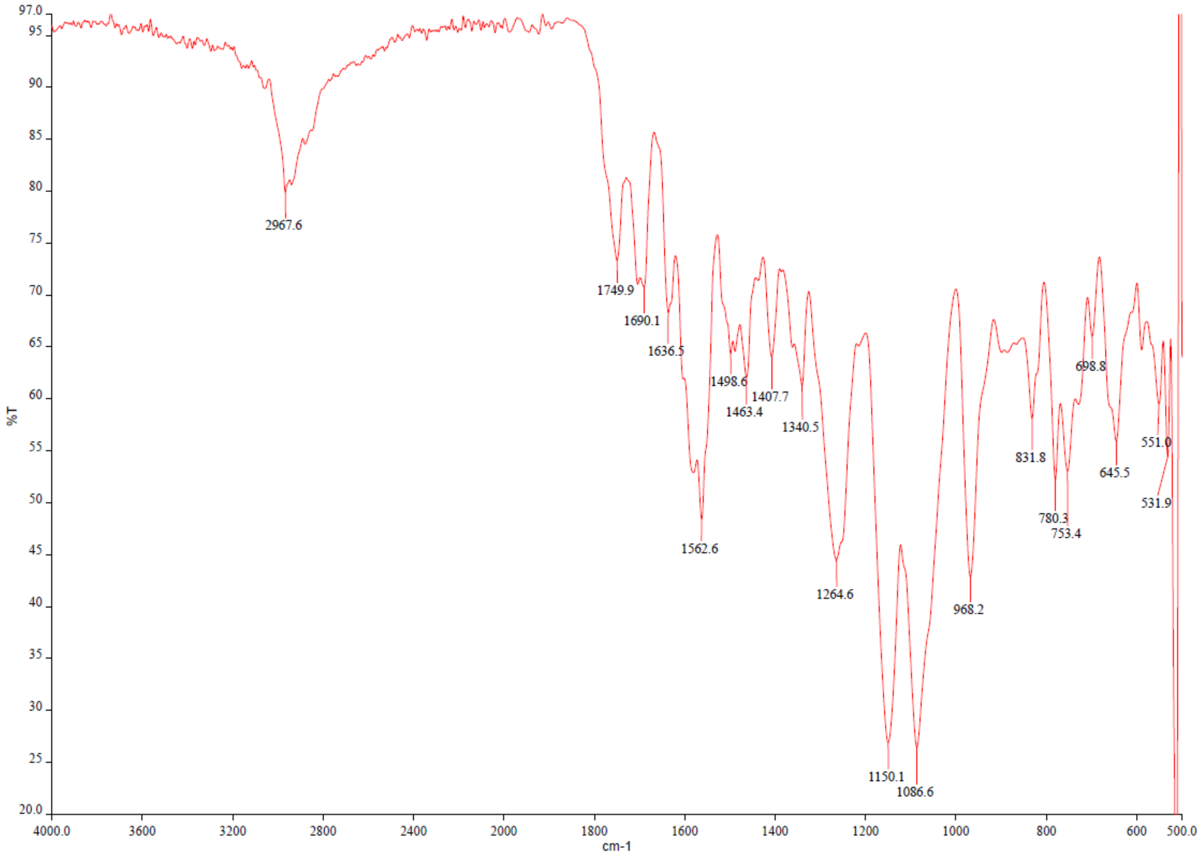

Figure S56. IR (neat) spectrum of **3g**.

Mass Spectrometry Core Facility

Lacour Group – University of Geneva

UNIVERSITÉ DE GENÈVE
FACULTÉ DES SCIENCES

ESI-HRMS – Certificate of Analysis

Applicant:	Yana Nikolova	Date of certificate:	16/04/21
Sample name:	YN-CF3-mal	Instrument:	Xevo G2 Tof (TOF)
Folder:	300321.PRO	Mobile phase:	MeOH (100 µl/min)
Analyst:	Stéphane Grass	Ionisation mode:	ESI (positive polarity)

Elemental Formula	Ion type	*Masslynx values **** *calc. m/z*	*meas. m/z*	Calc. *m/z*	Meas. *m/z*	Accuracy [a] *(ppm)*
$C_{31}H_{26}F_6N_2O_6$	$[M+H]^+$	*637.1773*	*637.1763*	637.1768	637.1758	-1.6

[a] *Mass spectrum is calibrated by the use of the MS lockspray system (LeuEnk calibration solution).*

**** MassLynx software does not take into account the mass of the electron for ionic species, therefore the shift of m/z 0.000459.*

Zoomed mass spectrum – Isotopic distribution.

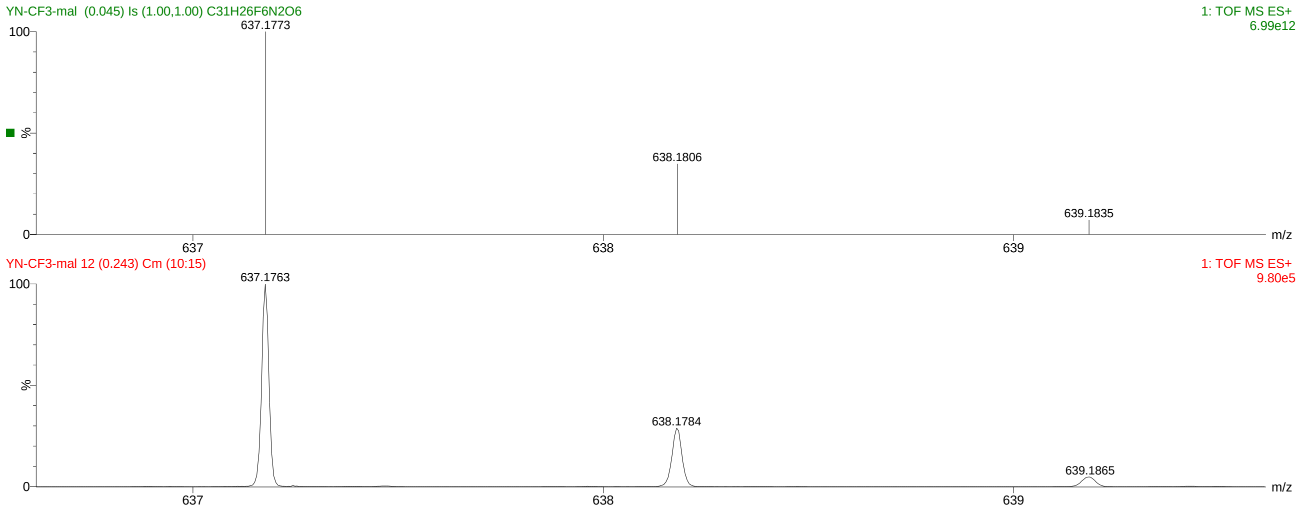

Figure S57. HRMS analysis (ESI, CH_3OH) report of **3g**.

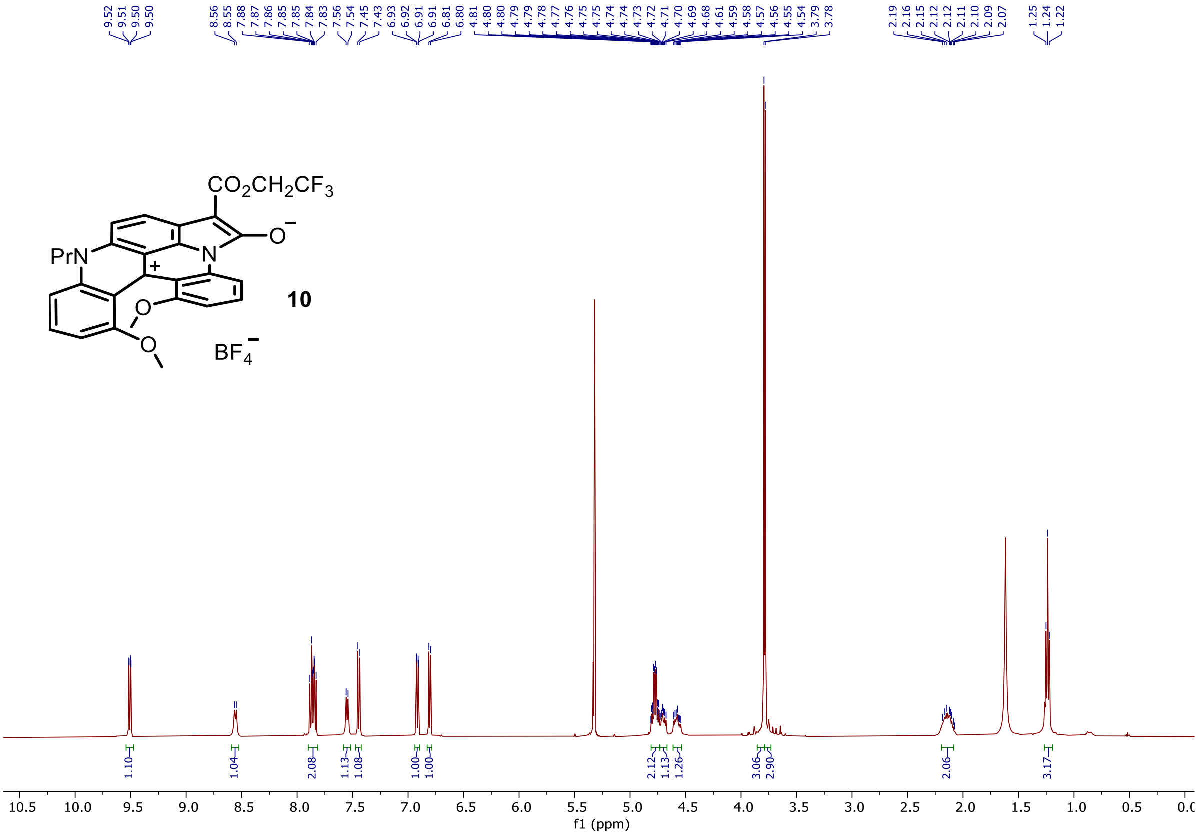

Figure S58. ^{1}H NMR (500 MHz, CD_2Cl_2) spectrum of **10**.

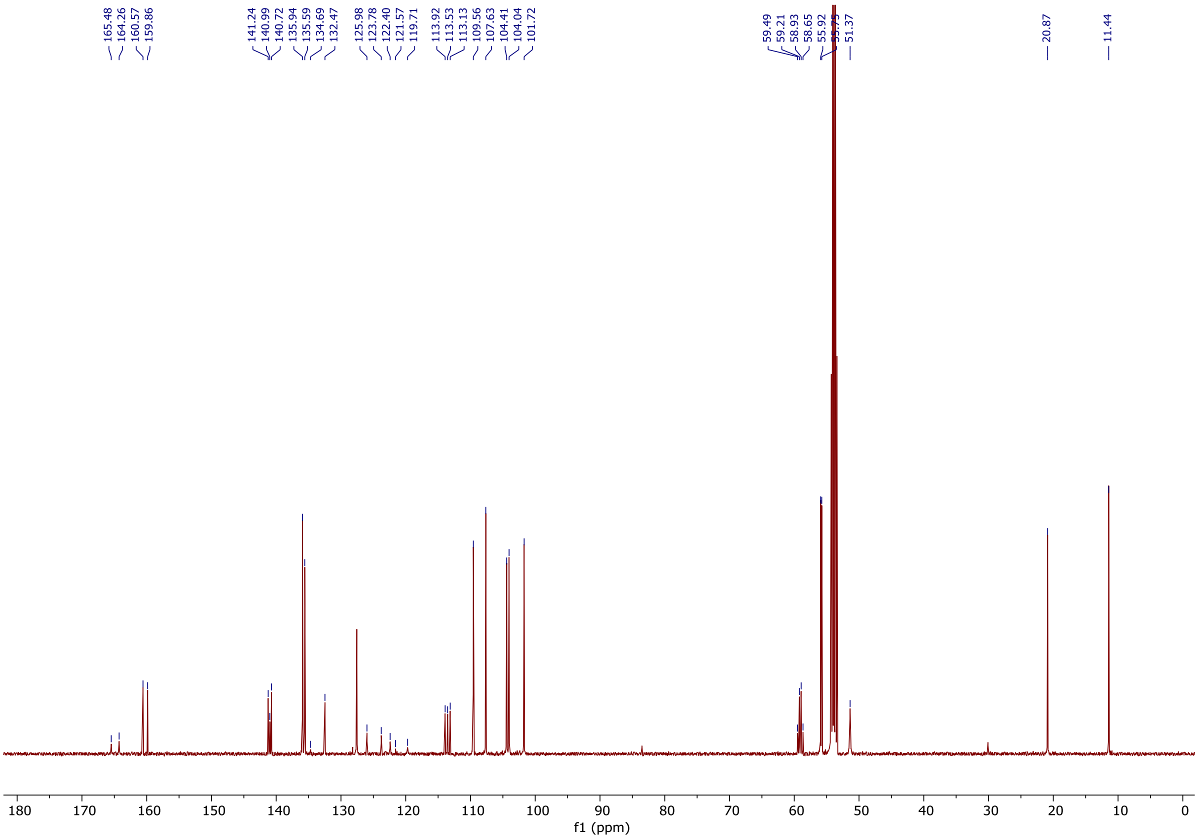

Figure S59. ^{13}C NMR (126 MHz, CD_2Cl_2) spectrum of **10**.

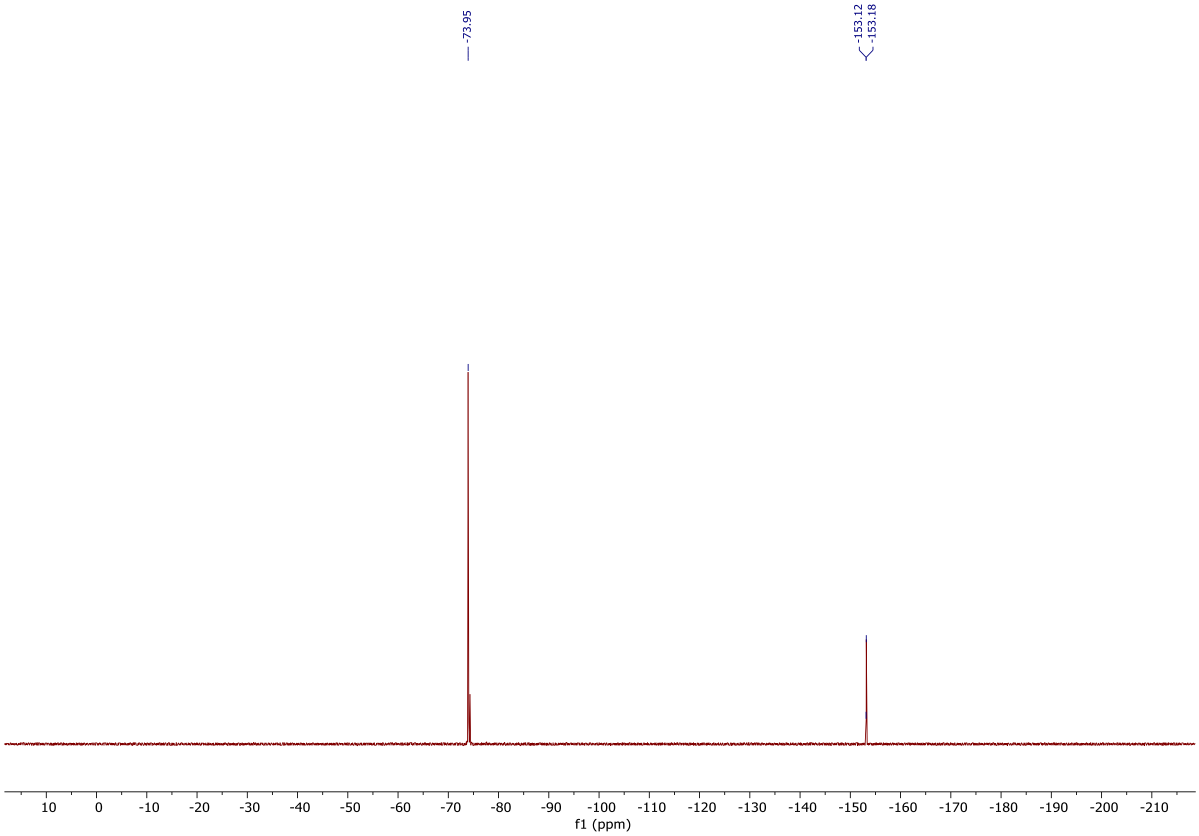

Figure S60. ^{19}F NMR (282 MHz, CD_2Cl_2) spectrum of **10**.

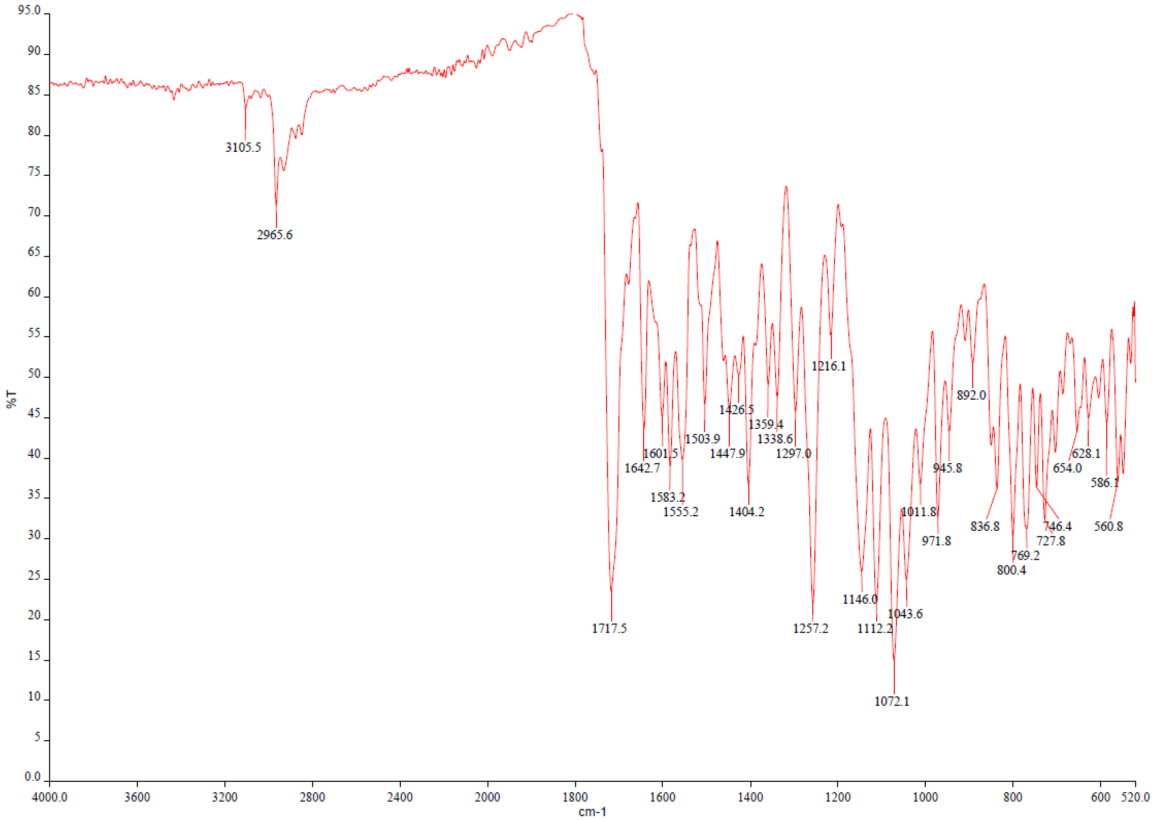

Figure S61. IR (neat) spectrum of **10**.

Mass Spectrometry Core Facility

Lacour Group – University of Geneva

UNIVERSITÉ DE GENÈVE
FACULTÉ DES SCIENCES

ESI-HRMS – Certificate of Analysis

Applicant:	Yana Nikolova	Date of certificate:	02/06/21
Sample name:	YN2-072-F4	Instrument:	Xevo G2 Tof (TOF)
Folder:	020621.PRO	Mobile phase:	MeOH (100 µl/min)
Analyst:	Stéphane Grass	Ionisation mode:	ESI (positive polarity)

Elemental Formula	Ion type	*Masslynx values **** *calc. m/z*	*meas. m/z*	Calc. *m/z*	Meas. *m/z*	Accuracy [a] *(ppm)*
$C_{29}H_{23}F_3N_2O_5$	$[M]^+$	*536.1559*	*536.1548*	536.1554	536.1543	-2.1

[a] *Mass spectrum is calibrated by the use of the MS lockspray system (LeuEnk calibration solution).*

**** MassLynx software does not take into account the mass of the electron for ionic species, therefore the shift of m/z 0.000459.*

<u>Zoomed mass spectrum – Isotopic distribution</u>.

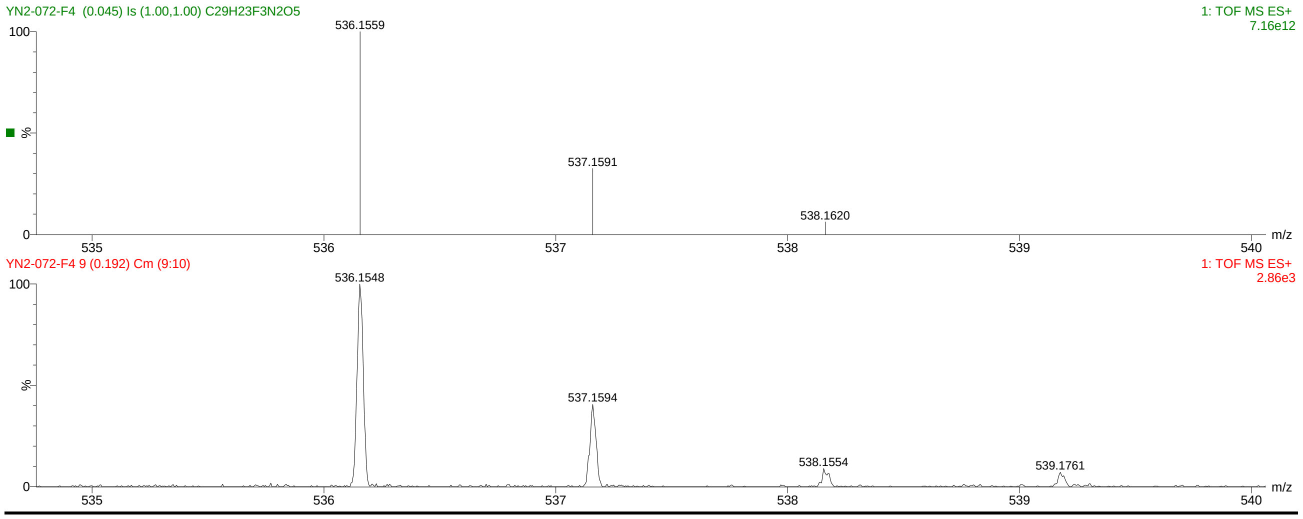

Figure S62. HRMS analysis (ESI, CH_3OH) report of **10**.

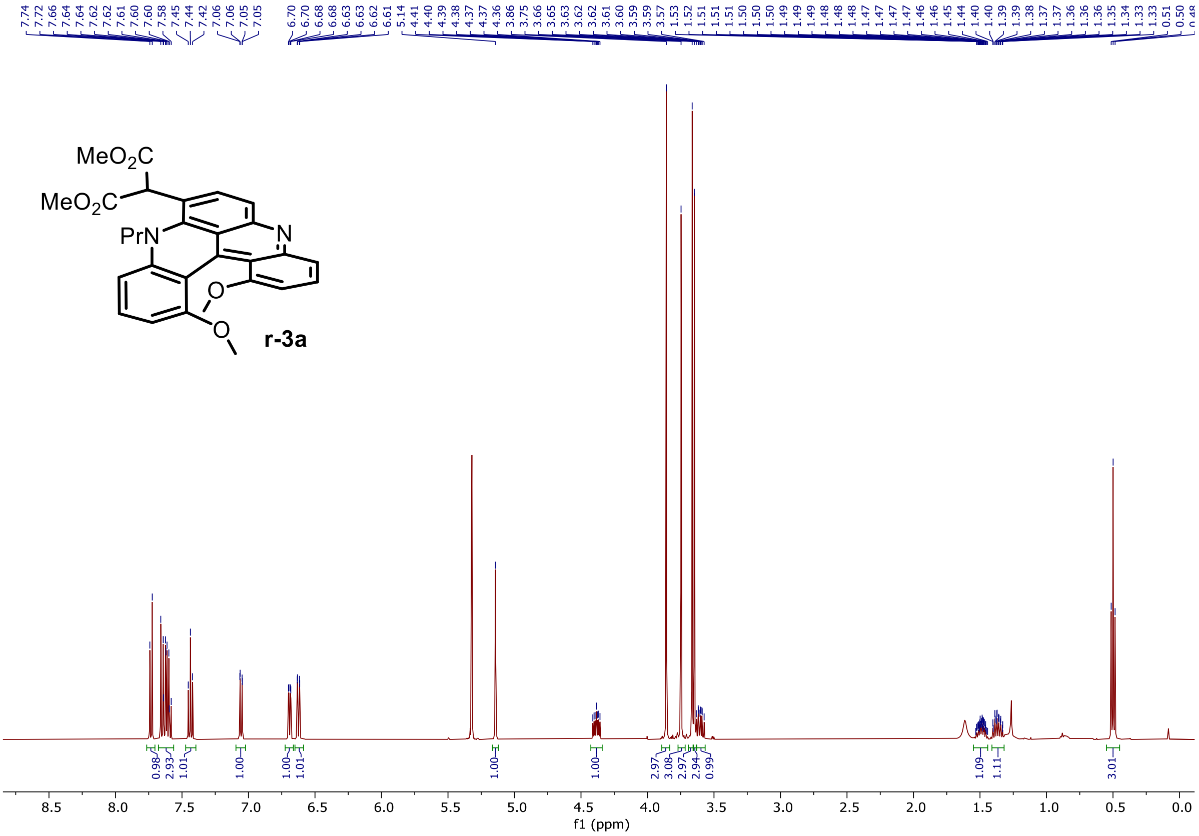

Figure S63. ^{1}H NMR (500 MHz, CD_2Cl_2) spectrum of **r-3a**.

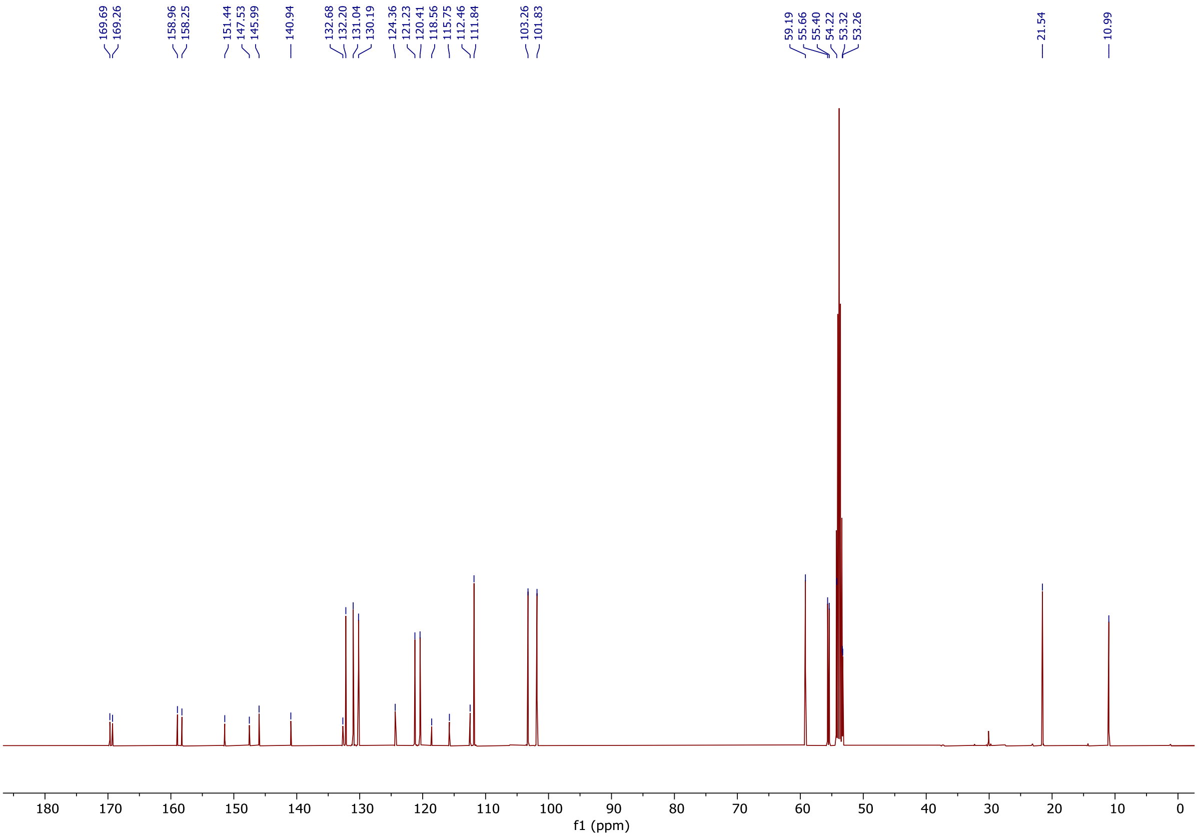

Figure S64. ^{13}C NMR (126 MHz, CD_2Cl_2) spectrum of **r-3a**.

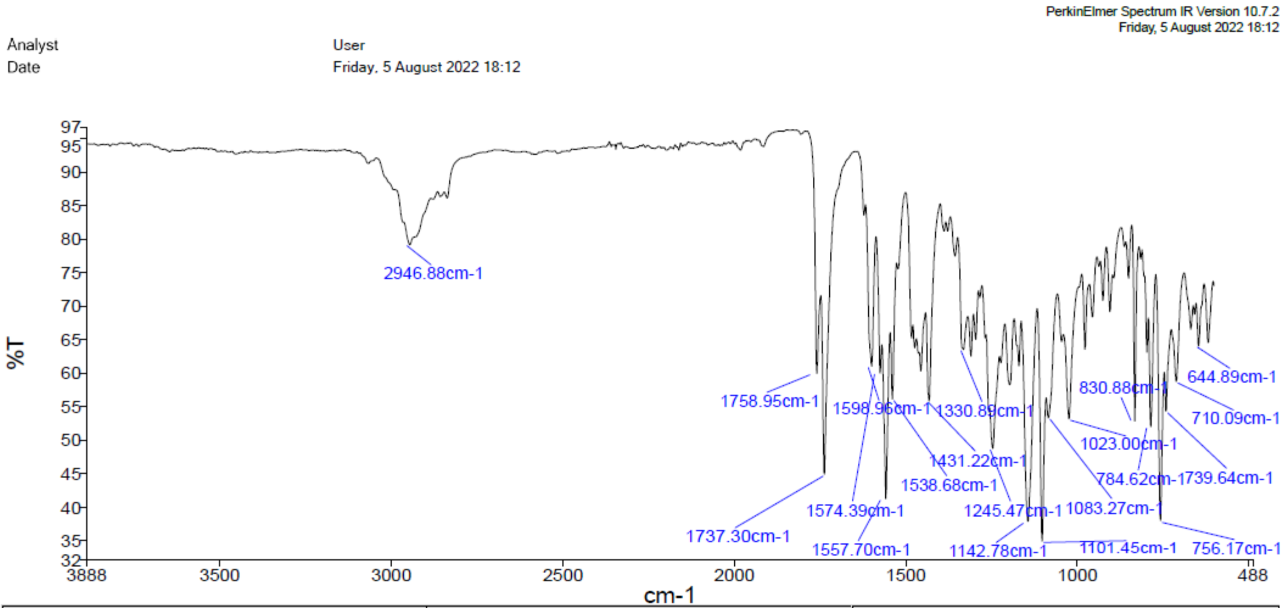

Sample Name	Description	Quality Checks
YN3-085-F1	Sample 012 By Guest Date Friday, August 05 2022	The Quality Checks do not report any warnings for the sample.

Figure S65. IR (neat) spectrum of **r-3a**.

Mass Spectrometry Core Facility

Lacour Group – University of Geneva

UNIVERSITÉ DE GENÈVE
FACULTÉ DES SCIENCES

ESI-HRMS – Certificate of Analysis

Applicant:	Yana Nikolova	Date of certificate:	05/08/22
Sample name:	YN3-085-F1	Instrument:	Xevo G2 Tof (TOF)
Folder:	050822.PRO	Mobile phase:	MeOH (100 µl/min)
Analyst:	Stéphane Grass	Ionisation mode:	ESI (positive polarity)

Elemental Formula	Ion type	Masslynx values *** calc. *m/z*	Masslynx values *** meas. *m/z*	Calc. *m/z*	Meas. *m/z*	Accuracy [a] (*ppm*)
$C_{29}H_{28}N_2O_6$	$[M+H]^+$	*501.2026*	*501.2048*	501.2021	501.2043	4.4

[a)] *Mass spectrum is calibrated by the use of the MS lockspray system (LeuEnk calibration solution).*

**** MassLynx software does not take into account the mass of the electron for ionic species, therefore the shift of m/z 0.000459.*

Zoomed mass spectrum – Isotopic distribution.

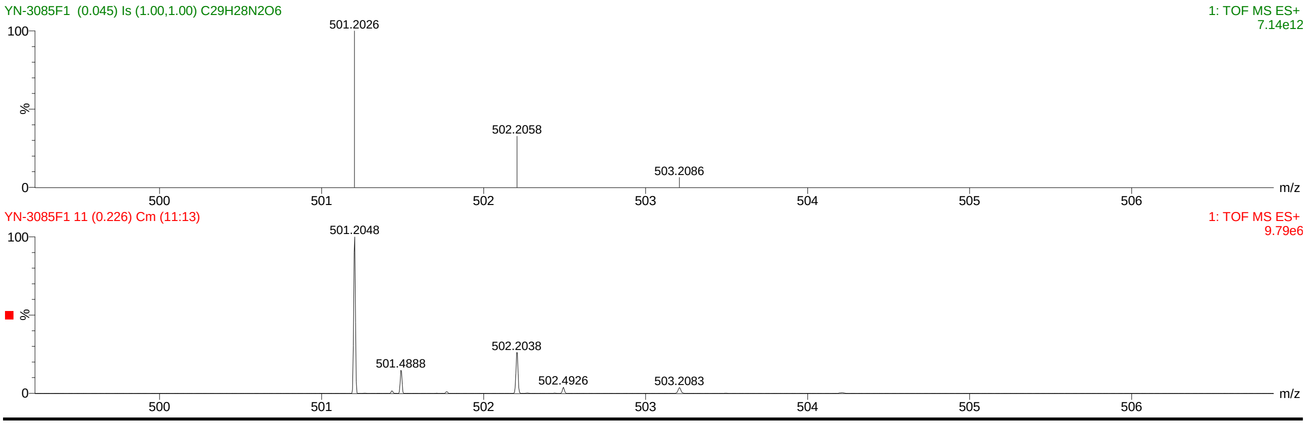

Figure S66. HRMS analysis (ESI, CH_3OH) report of **r-3a**.

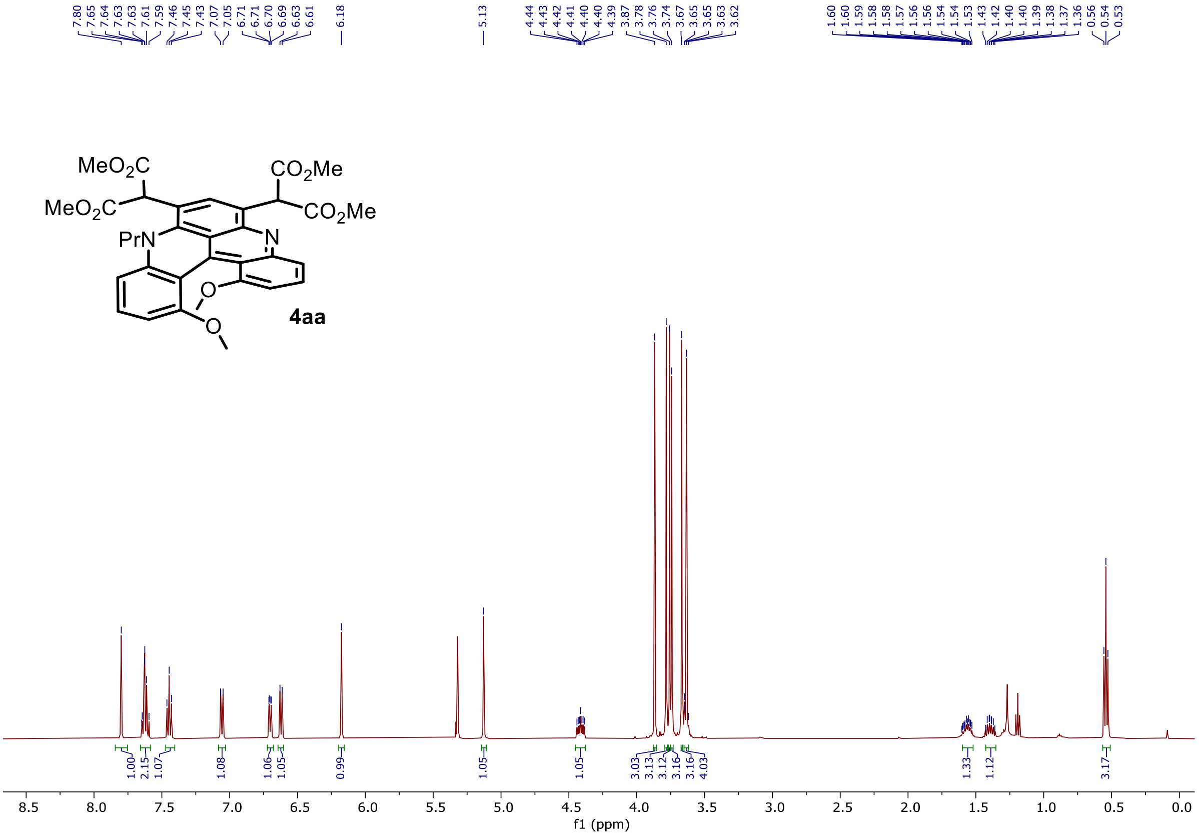

Figure S67. ^{1}H NMR (500 MHz, CD_2Cl_2) spectrum of **4aa**.

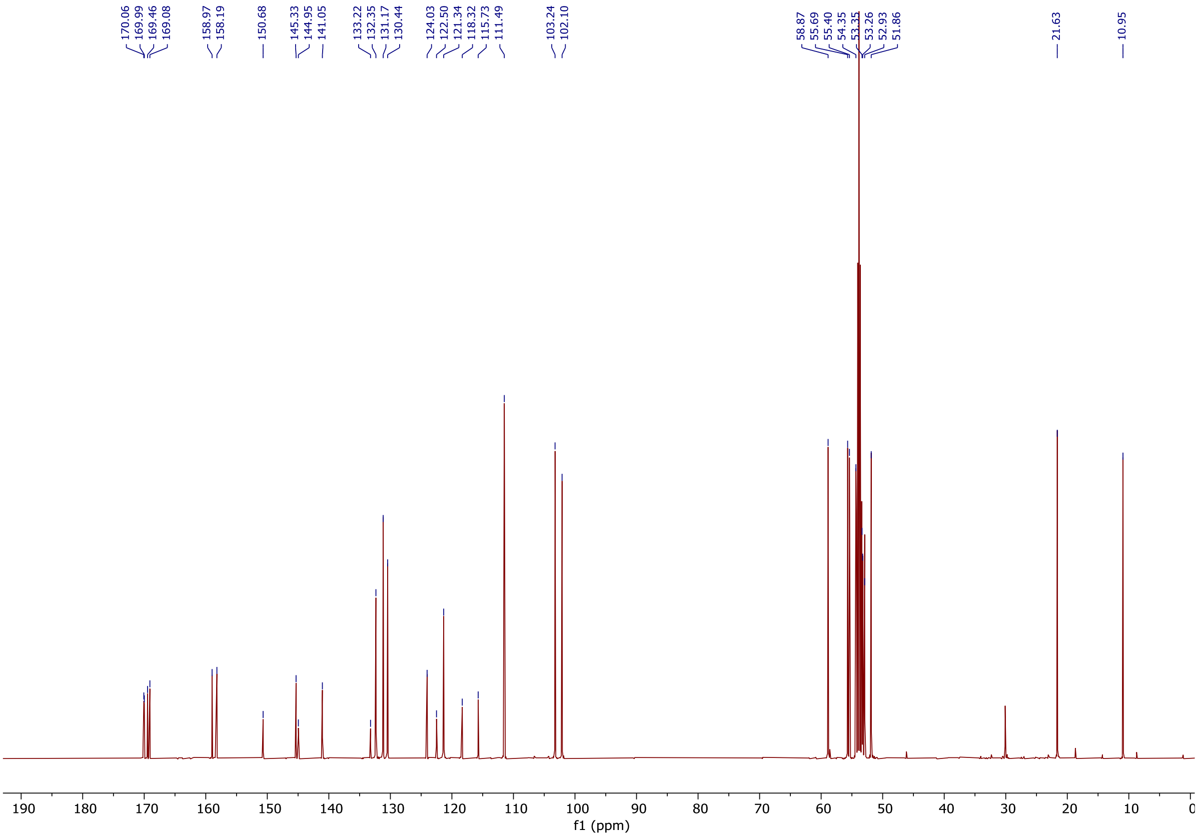

Figure S68. ^{13}C NMR (126 MHz, CD_2Cl_2) spectrum of **4aa**.

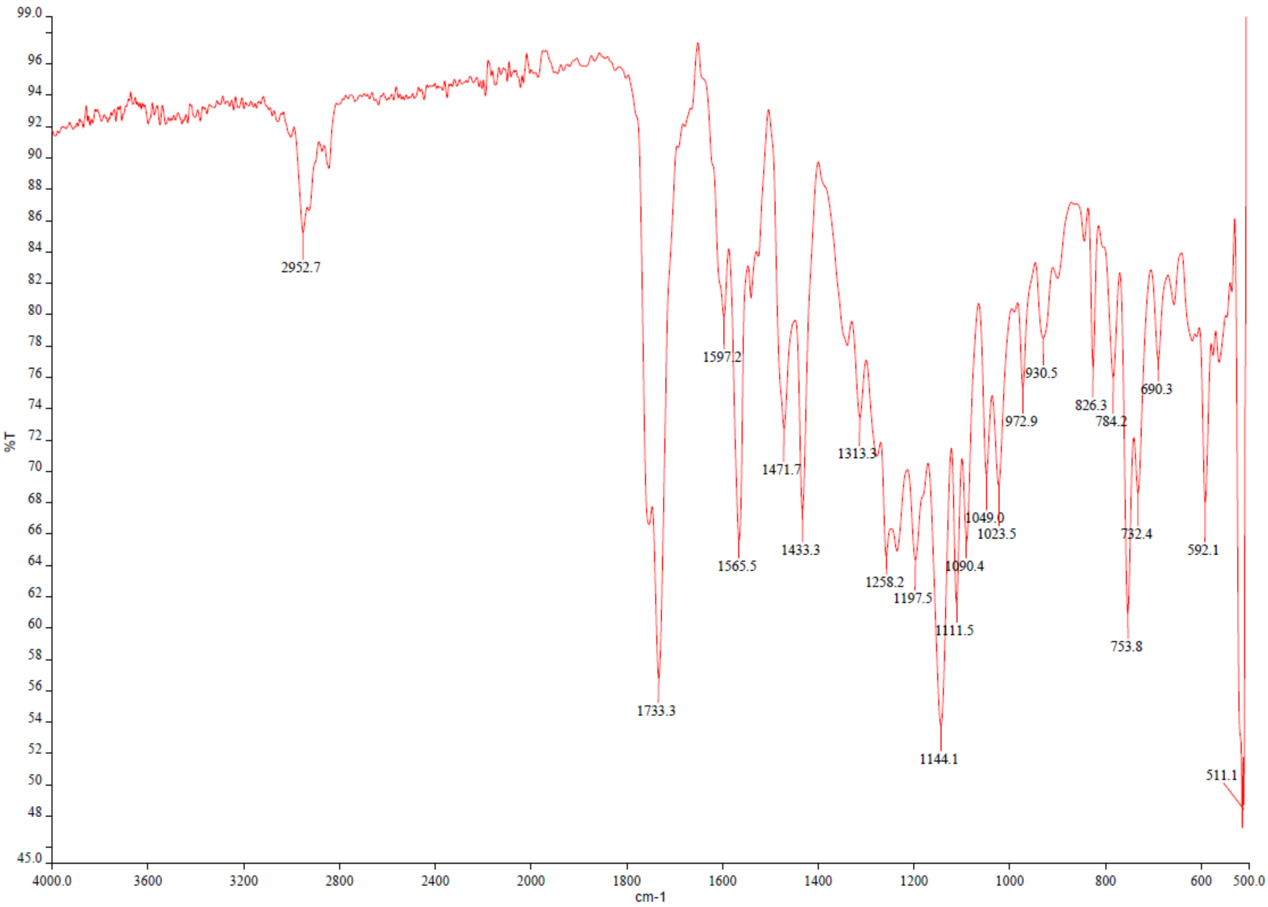

Figure S69. IR (neat) spectrum of **4aa**.

Mass Spectrometry Core Facility

Lacour Group – University of Geneva

UNIVERSITÉ DE GENÈVE
FACULTÉ DES SCIENCES

ESI-HRMS – Certificate of Analysis

Applicant:	Yana Nikolova	Date of certificate:	17/05/21
Sample name:	YN-bisMe	Instrument:	Xevo G2 Tof (TOF)
Folder:	170521.PRO	Mobile phase:	MeOH (100 µl/min)
Analyst:	Stéphane Grass	Ionisation mode:	ESI (positive polarity)

Elemental Formula	Ion type	*Masslynx values **** *calc. m/z*	*meas. m/z*	Calc. *m/z*	Meas. *m/z*	Accuracy [a] (ppm)
$C_{34}H_{34}N_2O_{10}$	$[M+H]^+$	*631.2292*	*631.2266*	631.2287	631.2261	-4.1

[a] *Mass spectrum is calibrated by the use of the MS lockspray system (LeuEnk calibration solution).*

**** MassLynx software does not take into account the mass of the electron for ionic species, therefore the shift of m/z 0.000459.*

Zoomed mass spectrum – Isotopic distribution.

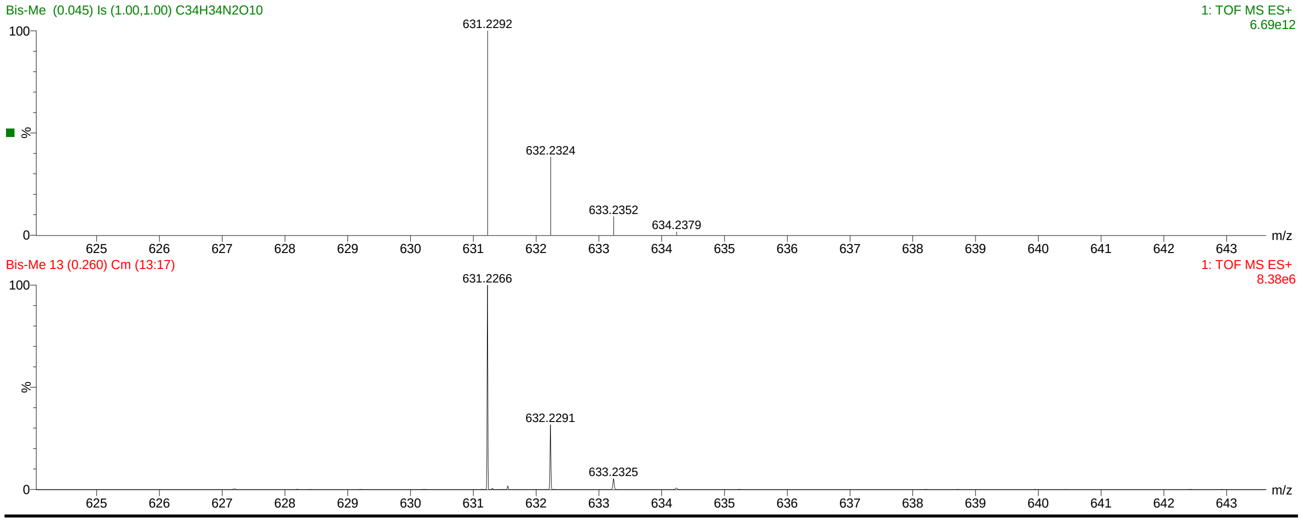

Figure S70. HRMS analysis (ESI, CH_3OH) report of **4aa**.

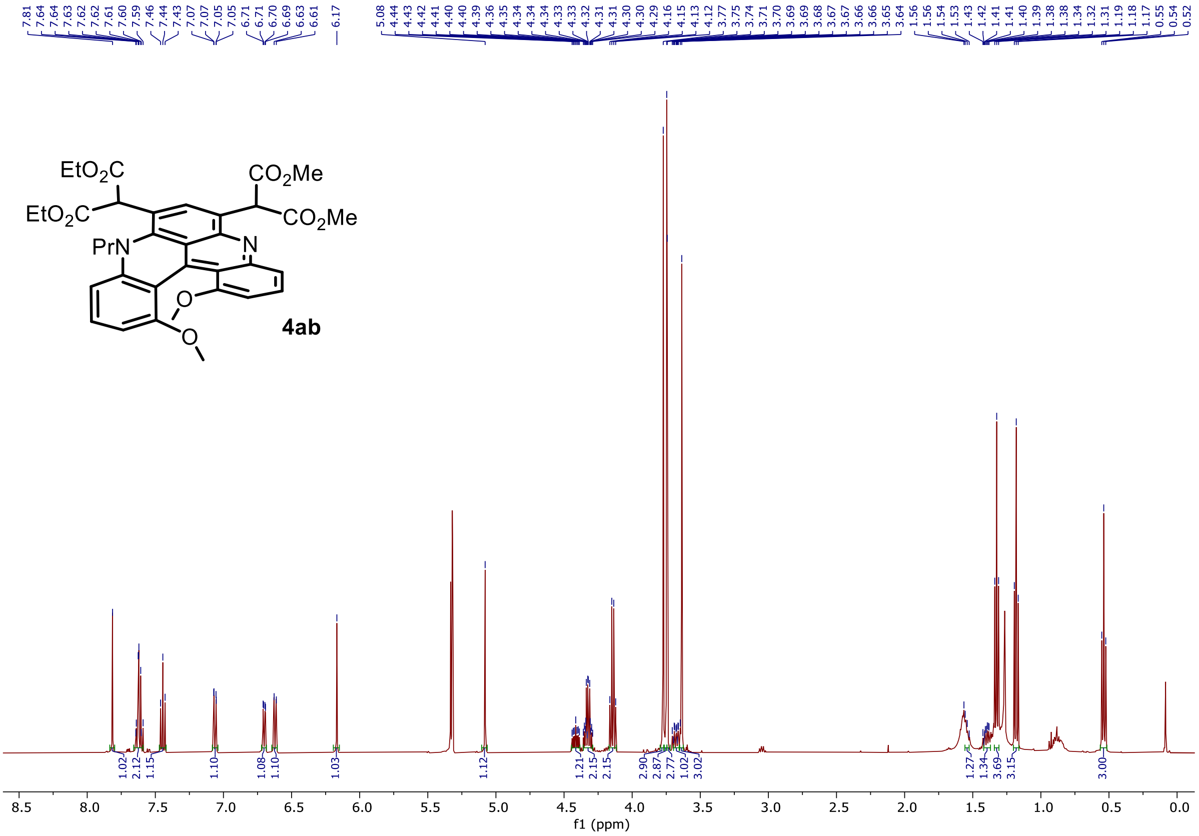

Figure S71. ^{1}H NMR (500 MHz, CD_2Cl_2) spectrum of **4ab**.

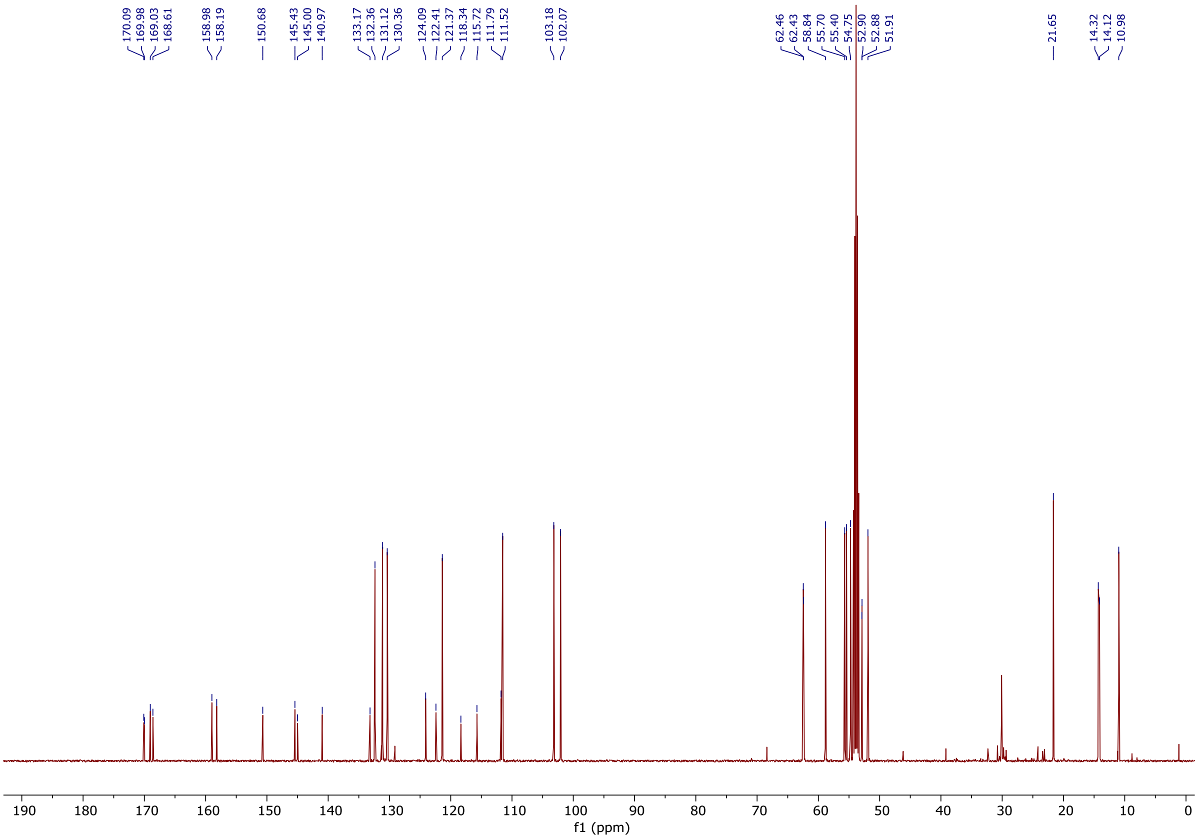

Figure S72. ^{13}C NMR (126 MHz, CD_2Cl_2) spectrum of **4ab**.

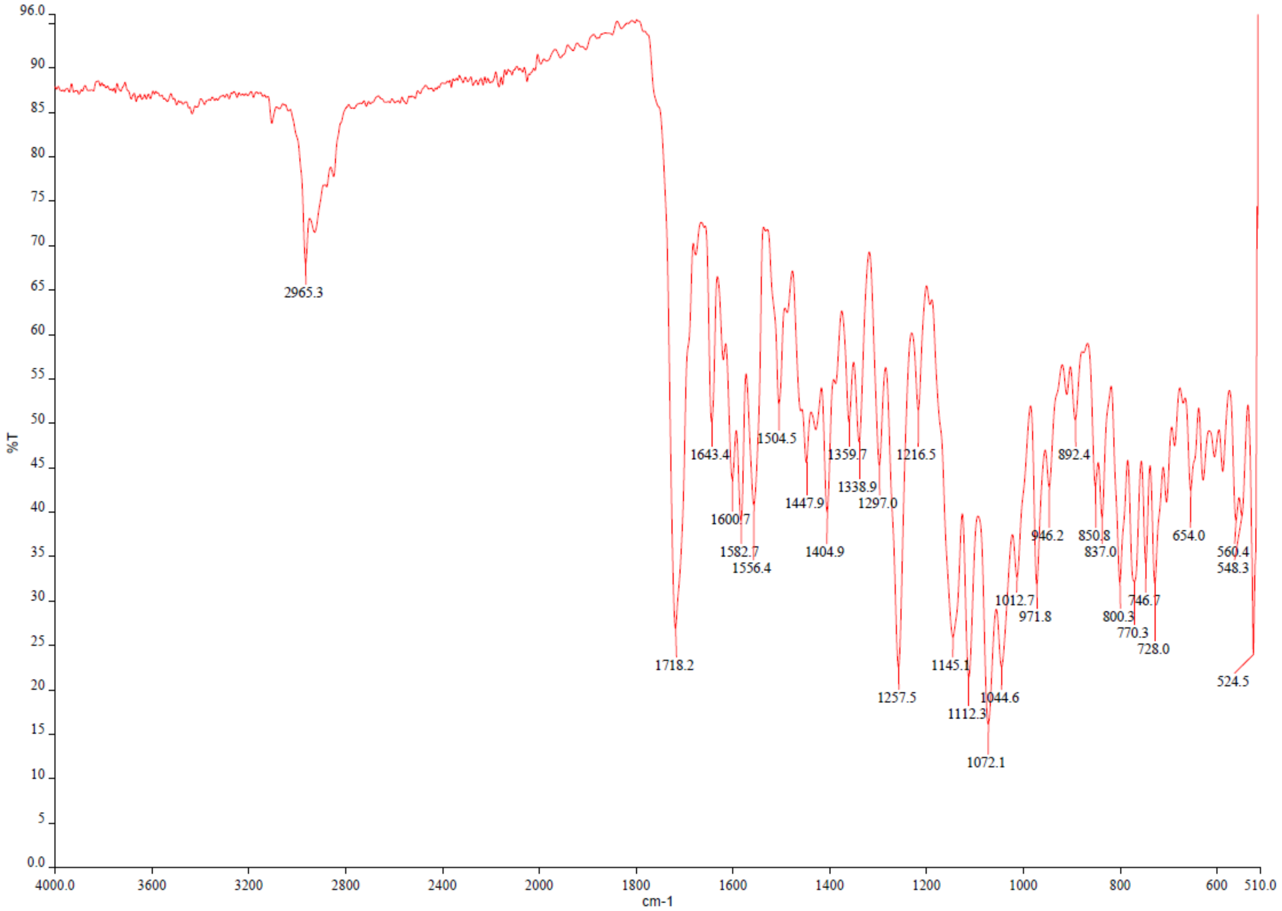

Figure S73. IR (neat) spectrum of **4ab**.

Mass Spectrometry Core Facility

Lacour Group – University of Geneva

UNIVERSITÉ DE GENÈVE
FACULTÉ DES SCIENCES

ESI-HRMS – Certificate of Analysis

Applicant:	Yana Nikolova	Date of certificate:	17/05/21
Sample name:	YN-PML428	Instrument:	Xevo G2 Tof (TOF)
Folder:	170521.PRO	Mobile phase:	MeOH (100 µl/min)
Analyst:	Stéphane Grass	Ionisation mode:	ESI (positive polarity)

Elemental Formula	Ion type	*Masslynx values **** *calc. m/z*	*meas. m/z*	Calc. *m/z*	Meas. *m/z*	Accuracy [a] *(ppm)*
$C_{36}H_{38}N_2O_{10}$	$[M+H]^+$	*659.2605*	*659.2569*	659.2600	659.2564	-5.5

[a] *Mass spectrum is calibrated by the use of the MS lockspray system (LeuEnk calibration solution).*

**** MassLynx software does not take into account the mass of the electron for ionic species, therefore the shift of m/z 0.000459.*

Zoomed mass spectrum – Isotopic distribution.

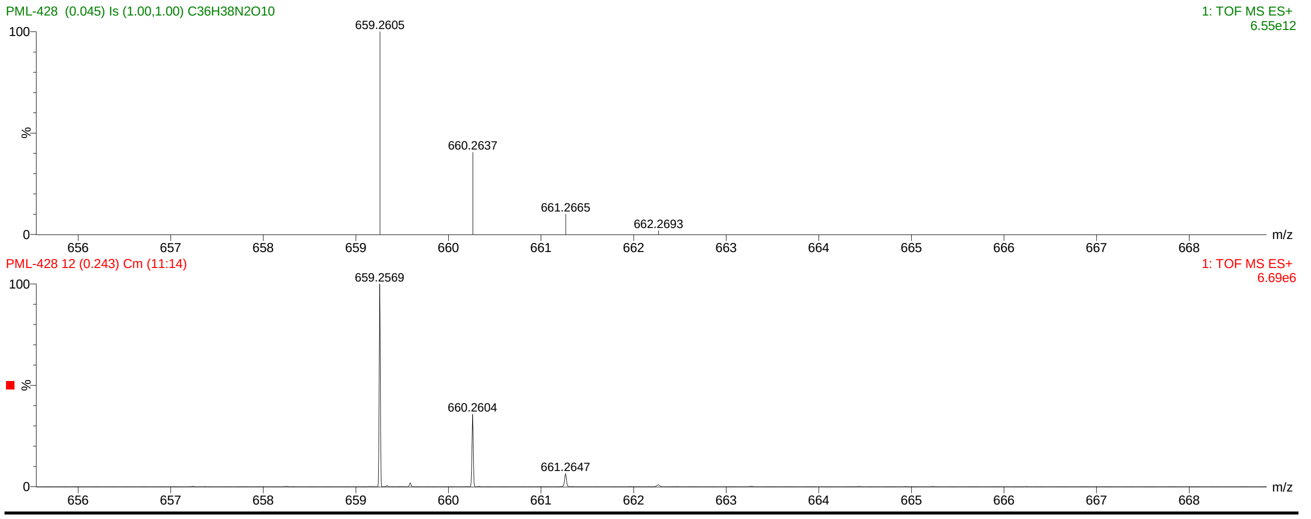

Figure S74. HRMS analysis (ESI, CH_3OH) report of **4ab**.

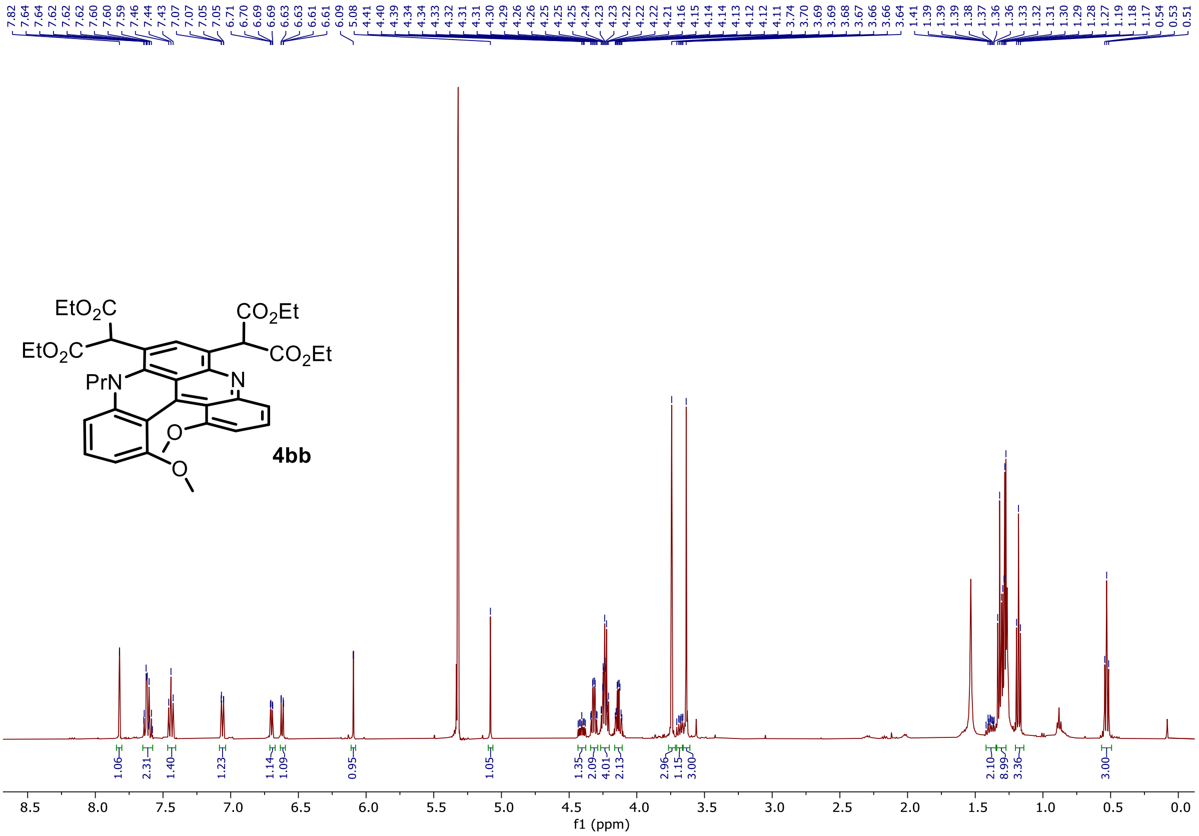

Figure S75. ^{1}H NMR (500 MHz, CD_2Cl_2) spectrum of **4bb**.

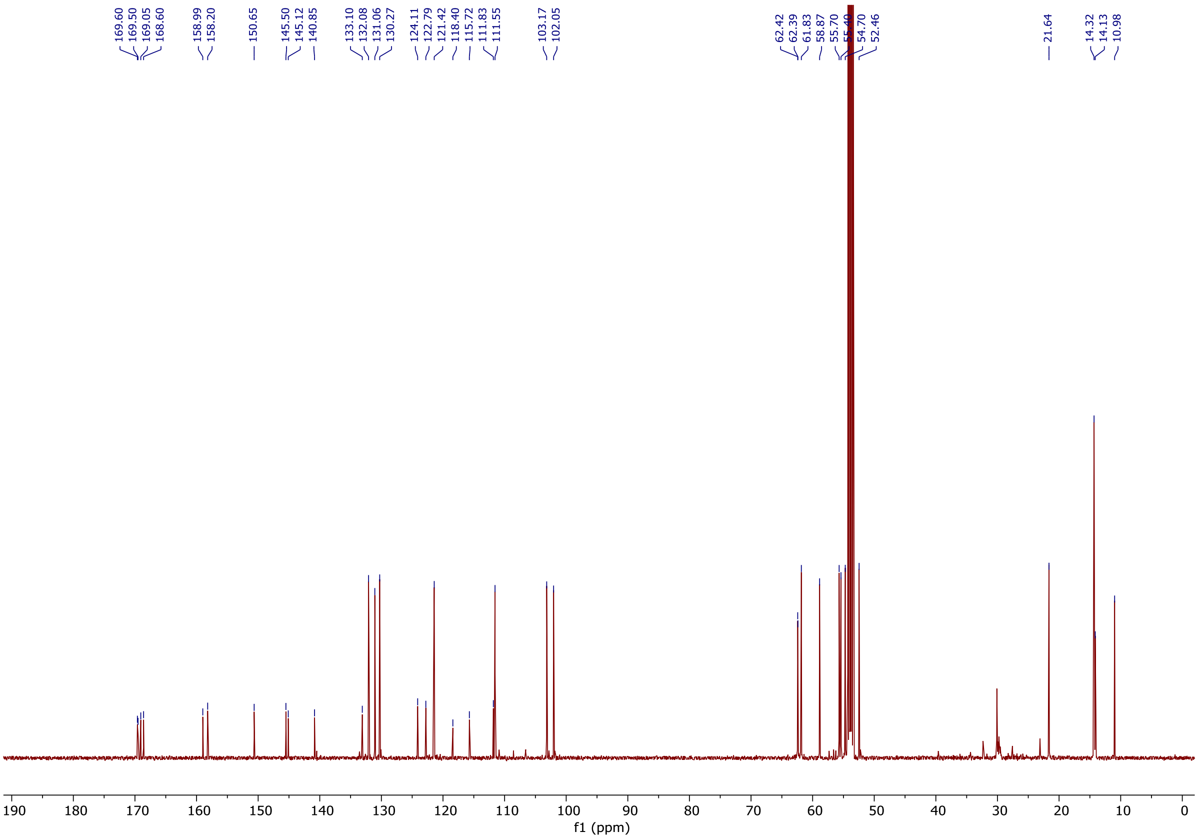

Figure S76. ^{13}C NMR (126 MHz, CD_2Cl_2) spectrum of **4bb**.

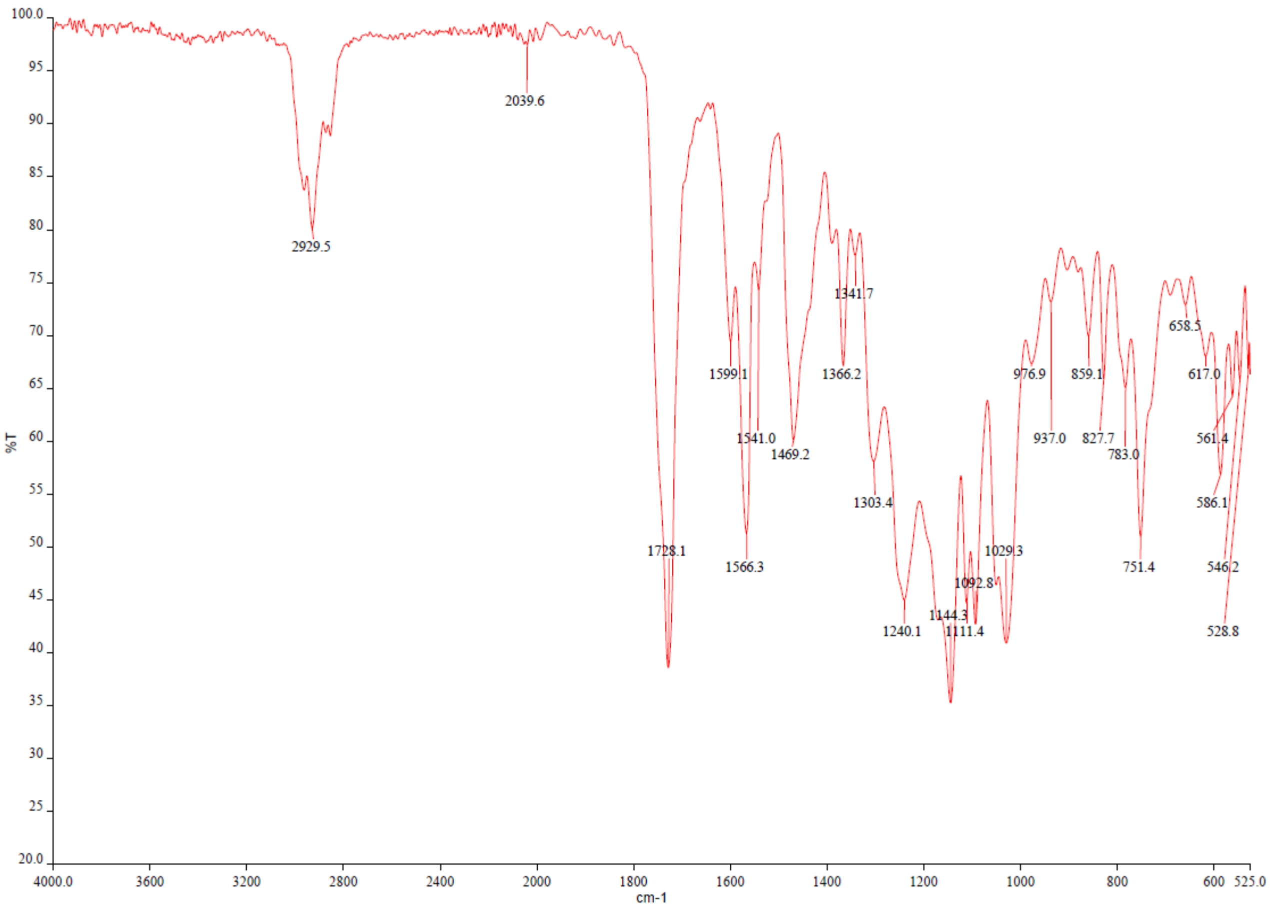

Figure S77. IR (neat) spectrum of **4bb**.

Mass Spectrometry Core Facility

Lacour Group – University of Geneva

UNIVERSITÉ DE GENÈVE
FACULTÉ DES SCIENCES

ESI-HRMS – Certificate of Analysis

Applicant:	Yana Nikolova	Date of certificate:	17/05/21
Sample name:	YN-bisEt	Instrument:	Xevo G2 Tof (TOF)
Folder:	170521.PRO	Mobile phase:	MeOH (100 µl/min)
Analyst:	Stéphane Grass	Ionisation mode:	ESI (positive polarity)

Elemental Formula	Ion type	*Masslynx values **** *calc. m/z*	*meas. m/z*	Calc. *m/z*	Meas. *m/z*	Accuracy [a] *(ppm)*
$C_{38}H_{42}N_2O_{10}$	$[M+H]^+$	*687.2917*	*687.2900*	687.2912	687.2895	-2.5

[a] *Mass spectrum is calibrated by the use of the MS lockspray system (LeuEnk calibration solution).*

**** MassLynx software does not take into account the mass of the electron for ionic species, therefore the shift of m/z 0.000459.*

Zoomed mass spectrum – Isotopic distribution.

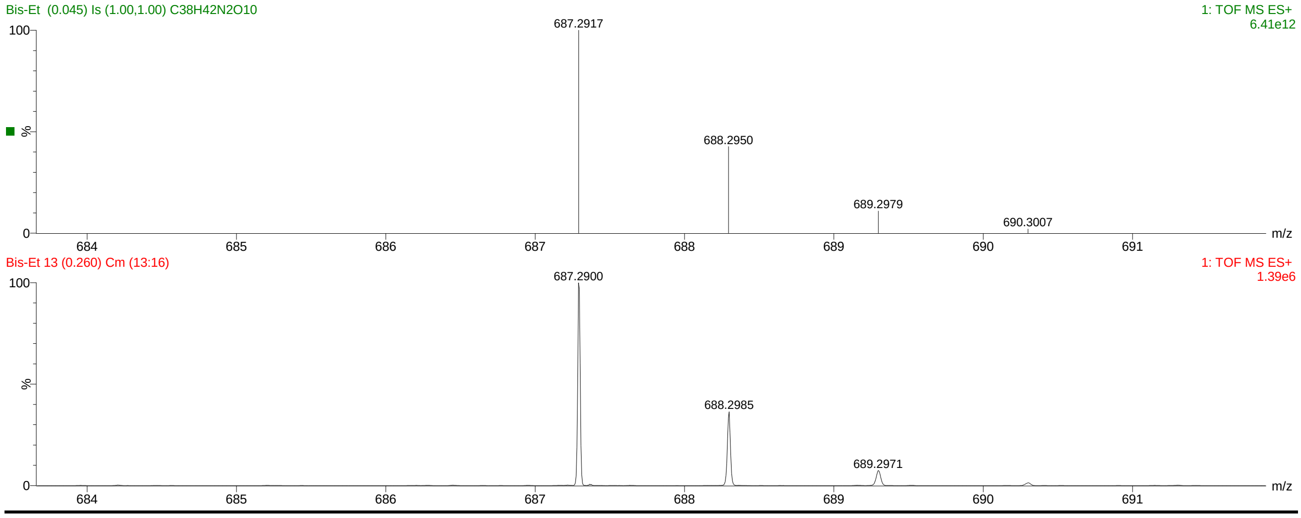

Figure S78. HRMS analysis (ESI, CH_3OH) report of **4bb**.

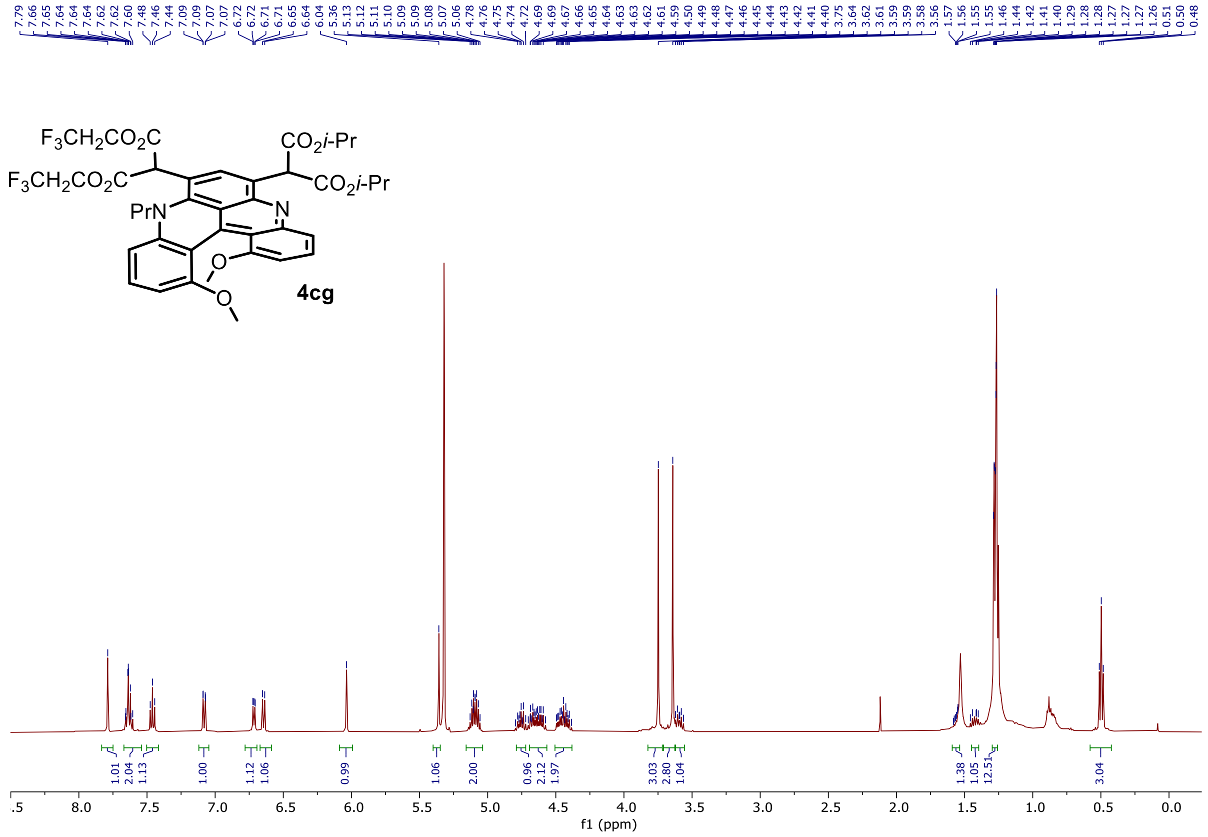

Figure S79. ^{1}H NMR (500 MHz, CD_2Cl_2) spectrum of **4cg**.

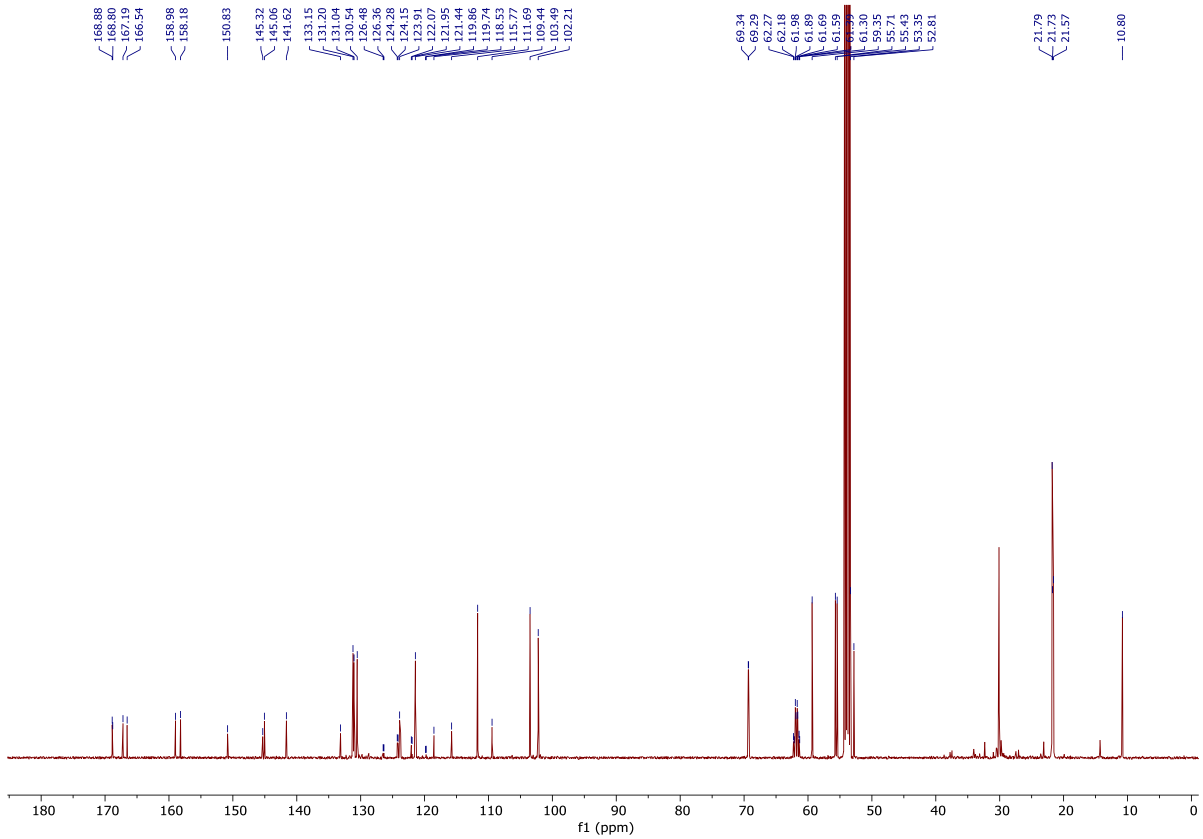

Figure S80. ^{13}C NMR (126 MHz, CD_2Cl_2) spectrum of **4cg**.

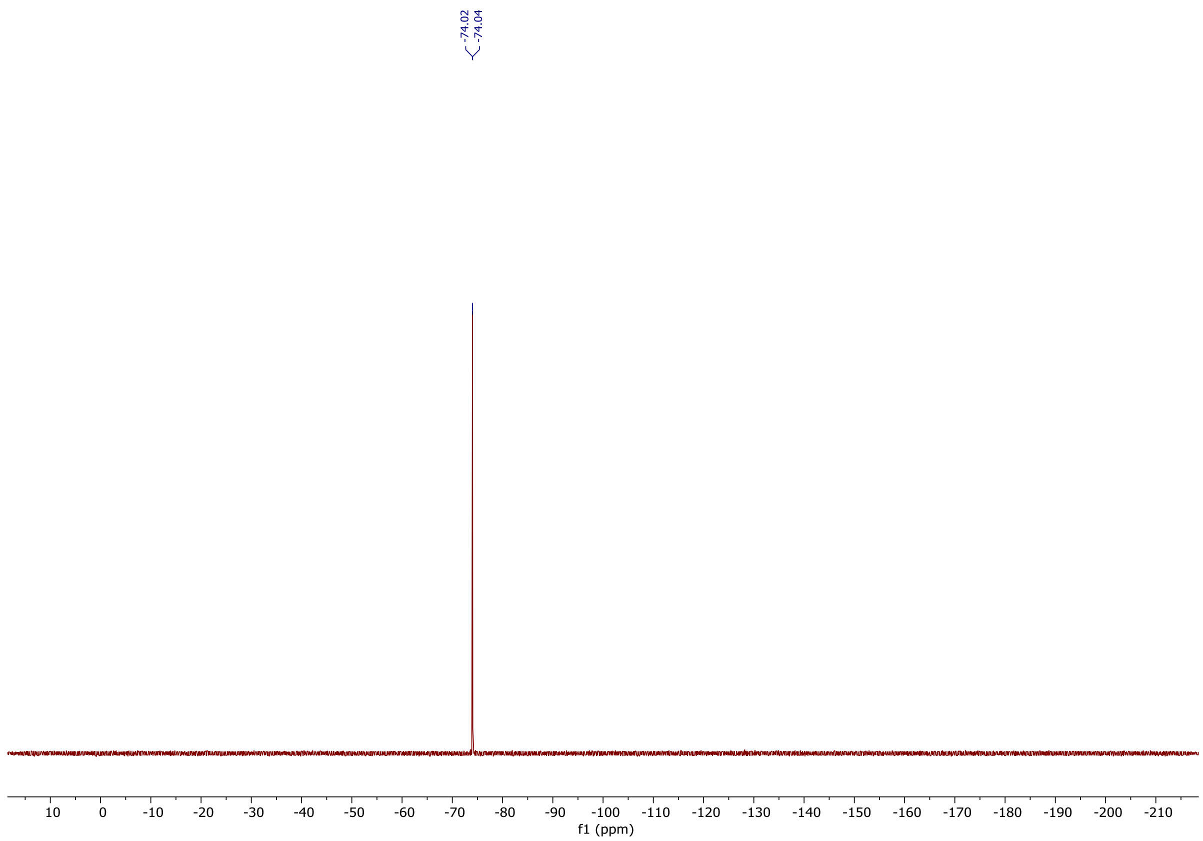

Figure S81. ^{19}F NMR (282 MHz, CD_2Cl_2) spectrum of **4cg**.

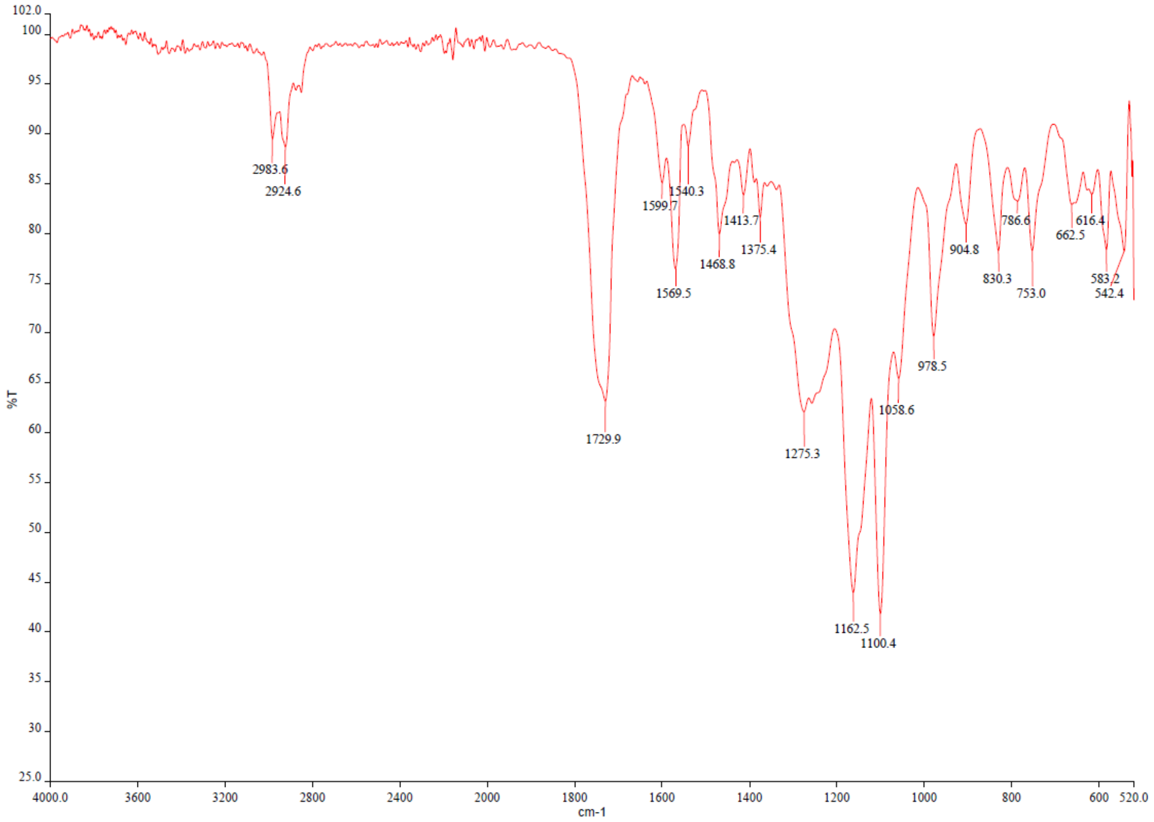

Figure S82. IR (neat) spectrum of **4cg**.

Mass Spectrometry Core Facility

Lacour Group – University of Geneva

UNIVERSITÉ DE GENÈVE
FACULTÉ DES SCIENCES

ESI-HRMS – Certificate of Analysis

Applicant:	Yana Nikolova	Date of certificate:	02/07/21
Sample name:	YN2-86-F3	Instrument:	Xevo G2 Tof (TOF)
Folder:	020721.PRO	Mobile phase:	MeOH (100 µl/min)
Analyst:	Stéphane Grass	Ionisation mode:	ESI (positive polarity)

Elemental Formula	Ion type	*Masslynx values *** calc. m/z*	*Masslynx values *** meas. m/z*	Calc. *m/z*	Meas. *m/z*	Accuracy [a] *(ppm)*
$C_{40}H_{40}F_6N_2O_{10}$	$[M+H]^+$	*823.2665*	*823.2657*	823.2660	823.2652	-1.0

[a] *Mass spectrum is calibrated by the use of the MS lockspray system (LeuEnk calibration solution).*

**** MassLynx software does not take into account the mass of the electron for ionic species, therefore the shift of m/z 0.000459.*

Zoomed mass spectrum – Isotopic distribution.

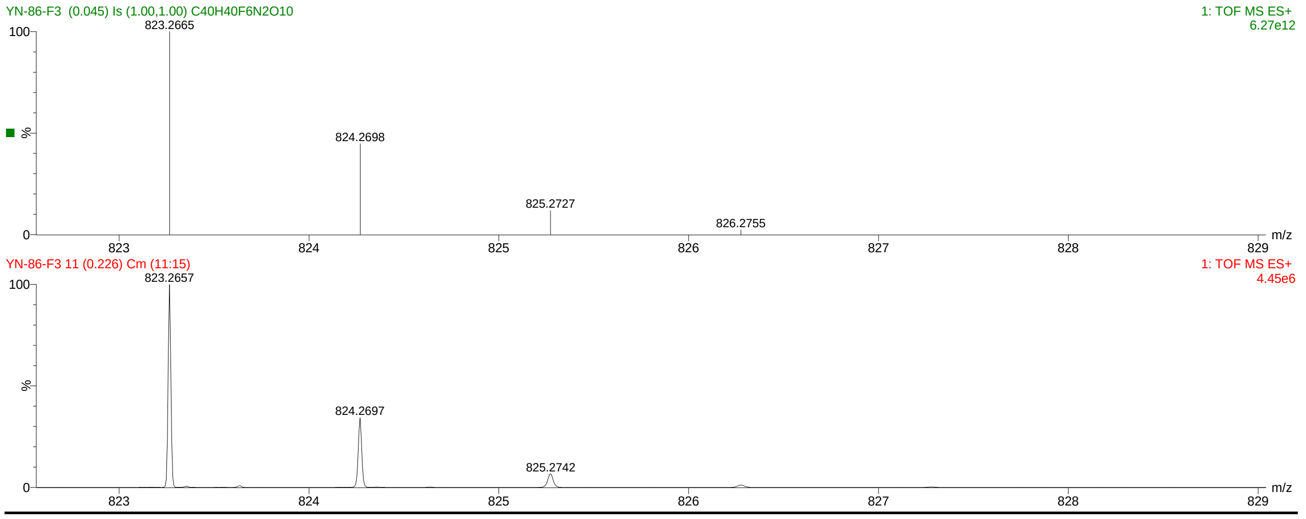

Figure S83. HRMS analysis (ESI, CH_3OH) report of **4cg**.

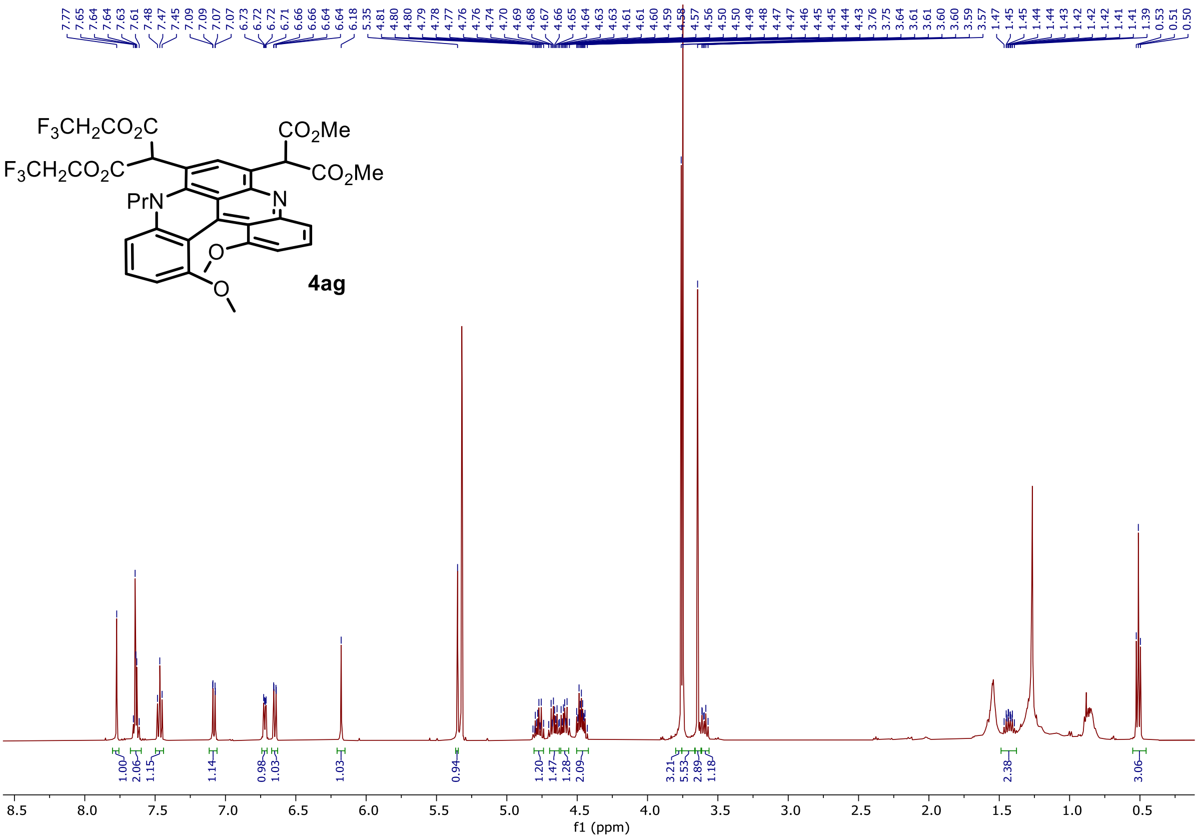

Figure S84. ^{1}H NMR (500 MHz, CD_2Cl_2) spectrum of **4ag**.

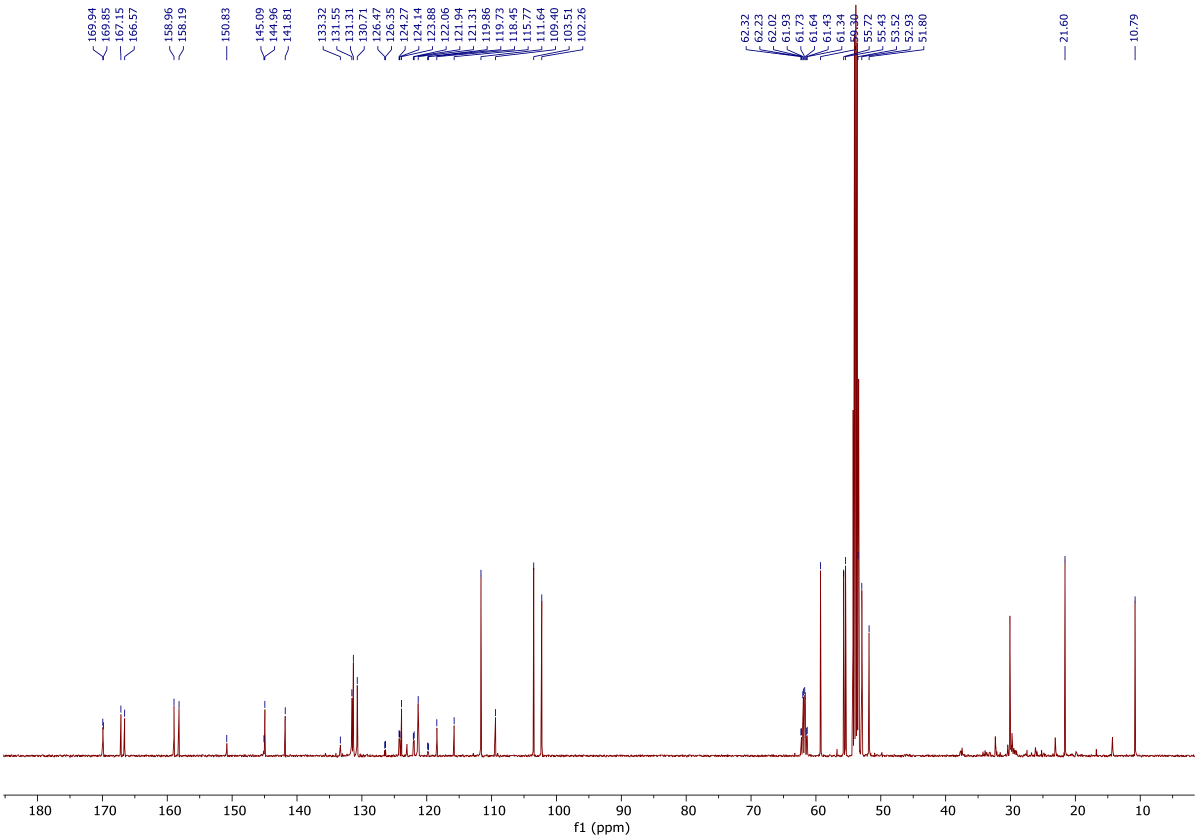

Figure S85. ^{13}C NMR (126 MHz, CD_2Cl_2) spectrum of **4ag**.

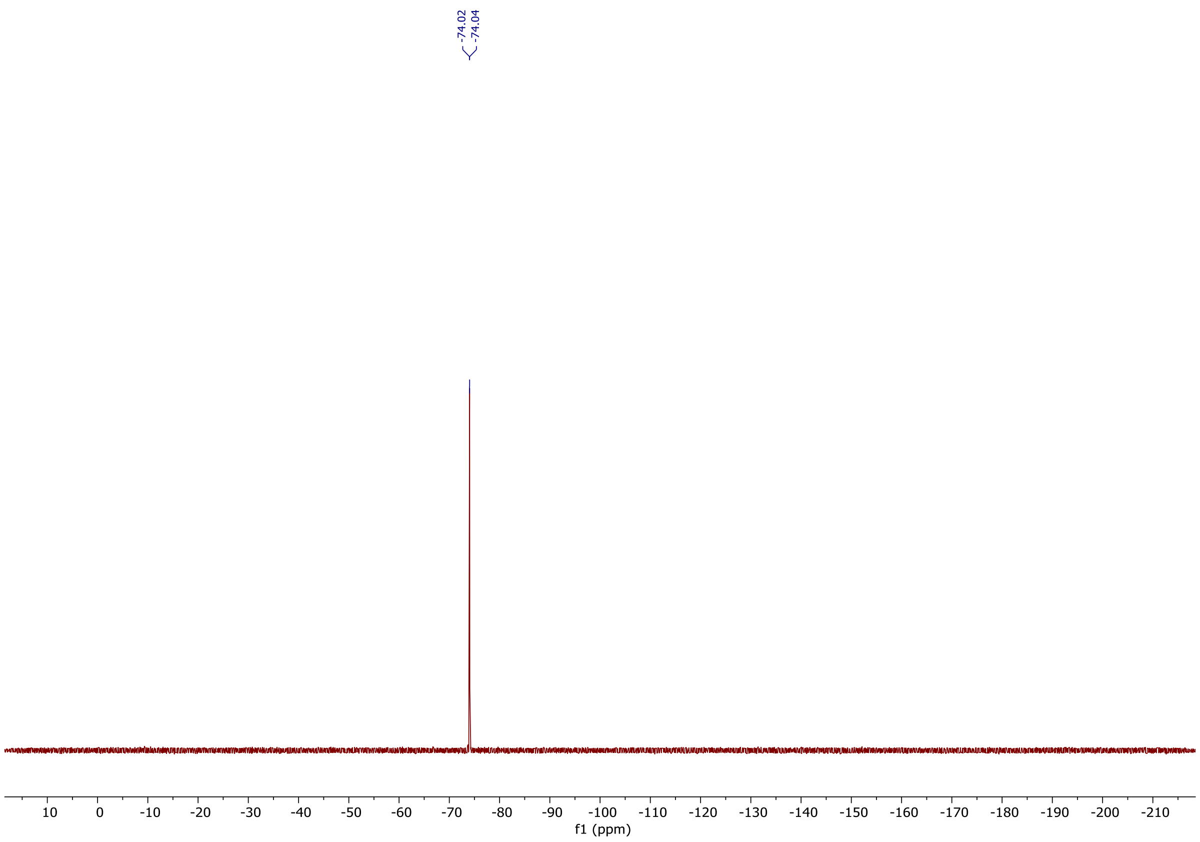

Figure S86. ^{19}F NMR (282 MHz, CD_2Cl_2) spectrum of **4ag**.

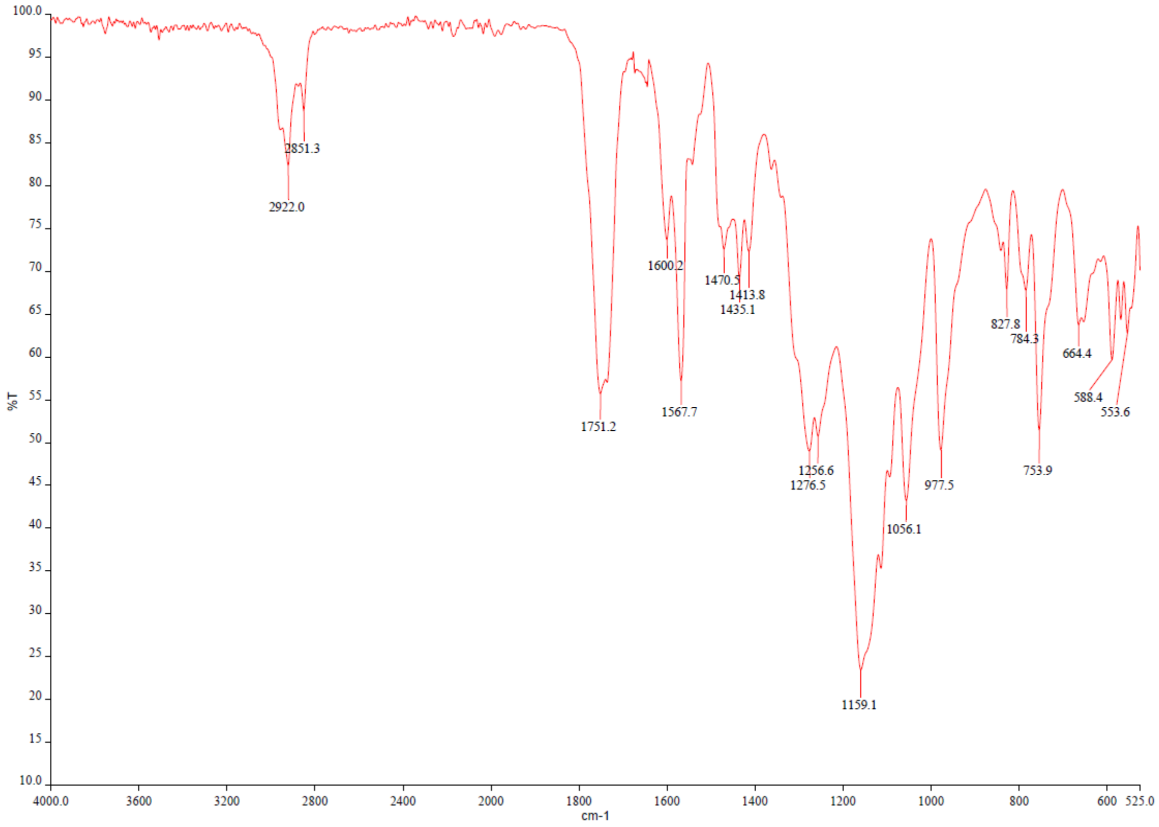

Figure S87. IR (neat) spectrum of **4ag**.

Mass Spectrometry Core Facility

Lacour Group – University of Geneva

UNIVERSITÉ DE GENÈVE
FACULTÉ DES SCIENCES

ESI-HRMS – Certificate of Analysis

Applicant:	Yana Nikolova	Date of certificate:	02/07/21
Sample name:	YN2-85-F1	Instrument:	Xevo G2 Tof (TOF)
Folder:	020721.PRO	Mobile phase:	MeOH (100 µl/min)
Analyst:	Stéphane Grass	Ionisation mode:	ESI (positive polarity)

Elemental Formula	Ion type	*Masslynx values **** *calc. m/z*	*meas. m/z*	Calc. *m/z*	Meas. *m/z*	Accuracy [a] *(ppm)*
$C_{36}H_{32}F_6N_2O_{10}$	$[M+H]^+$	*767.2039*	*767.2065*	767.2034	767.2060	3.4

[a] *Mass spectrum is calibrated by the use of the MS lockspray system (LeuEnk calibration solution).*

**** MassLynx software does not take into account the mass of the electron for ionic species, therefore the shift of m/z 0.000459.*

Zoomed mass spectrum – Isotopic distribution.

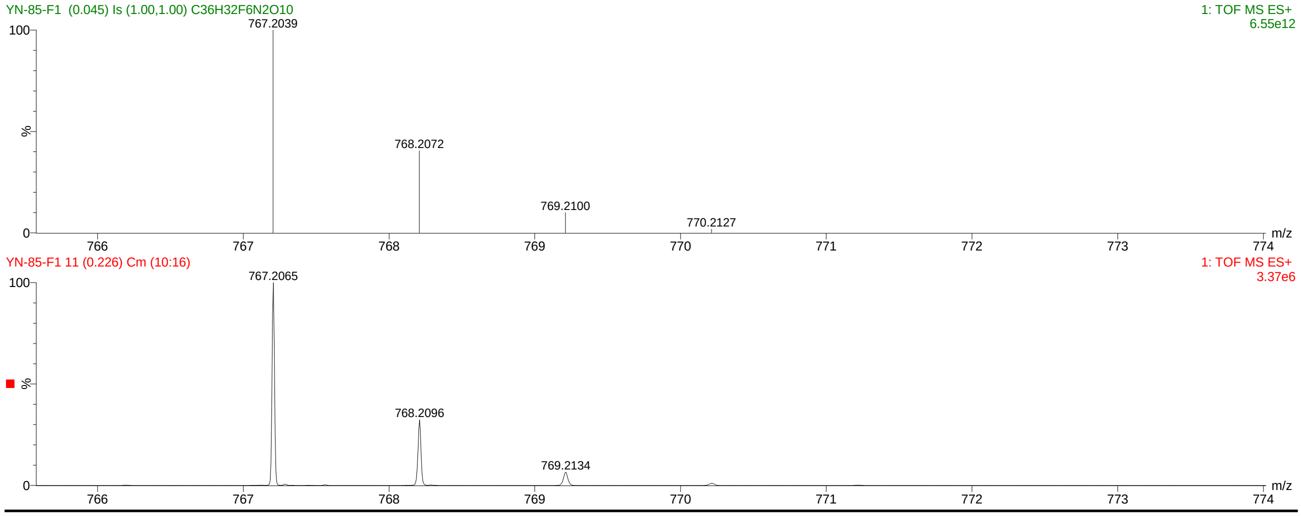

Figure S88. HRMS analysis (ESI, CH_3OH) report of **4ag**.

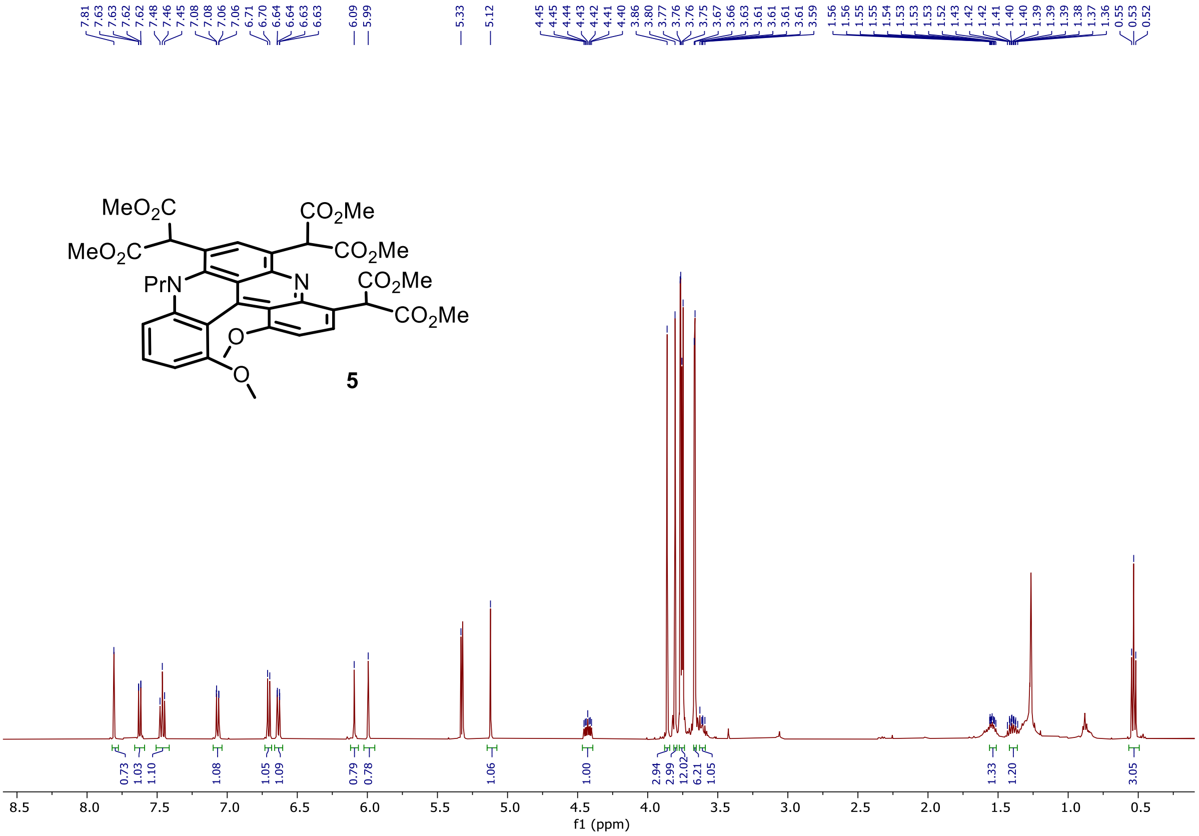

Figure S89. ^{1}H NMR (500 MHz, CD_2Cl_2) spectrum of **5**.

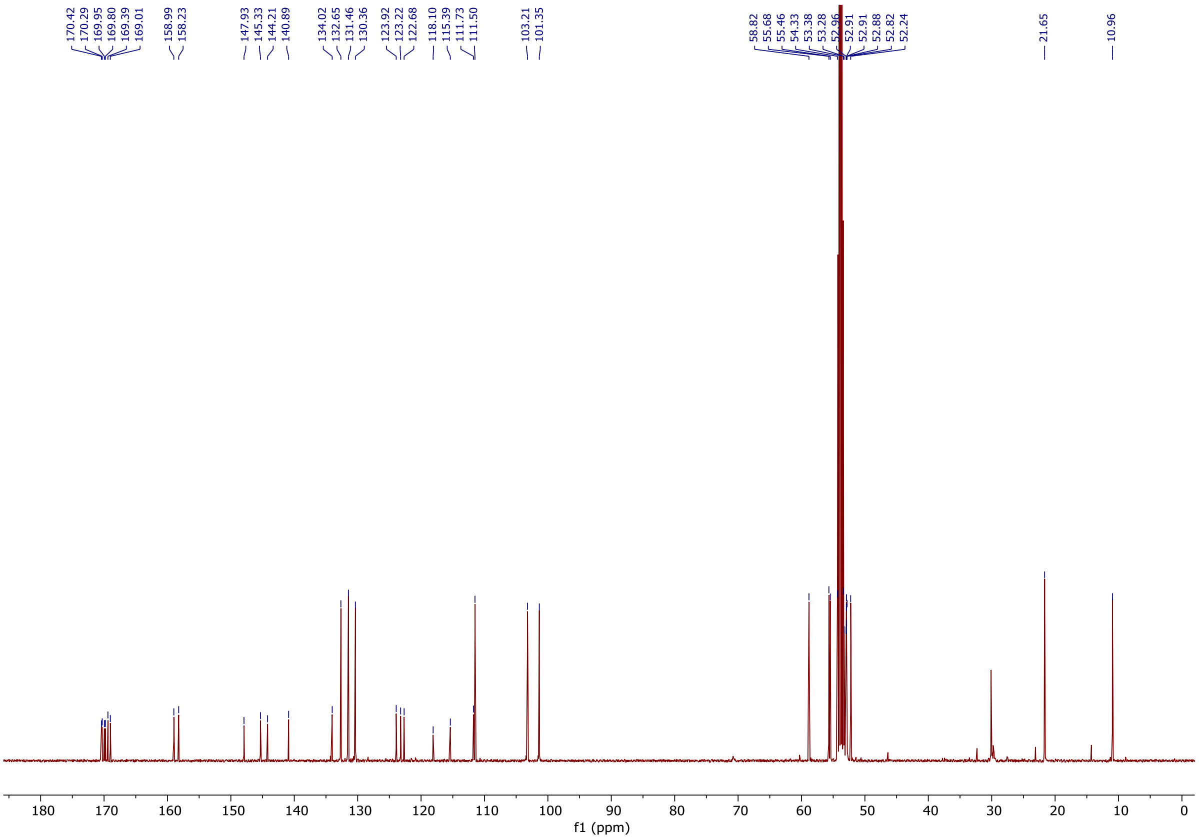

Figure S90. ^{13}C NMR (126 MHz, CD_2Cl_2) spectrum of **5**.

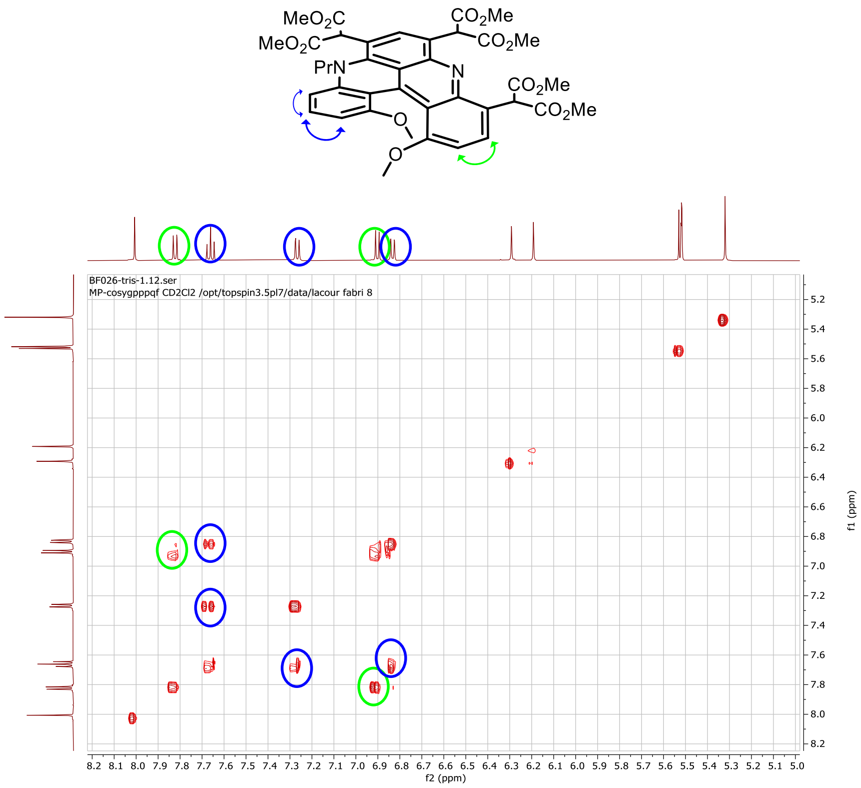

Figure S91. COSY (500 MHz, CD_2Cl_2) analysis (solution state conformation) of **5**.

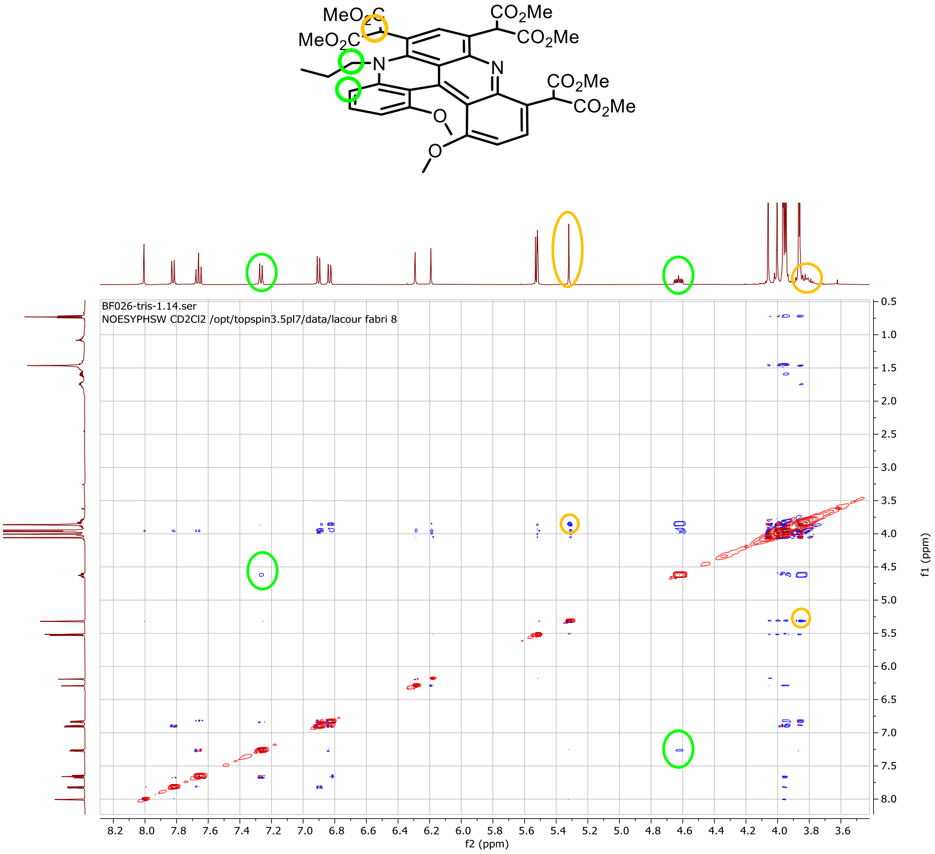

Figure S92. NOESY (500 MHz, CD_2Cl_2) analysis (solution state conformation) of **5**.

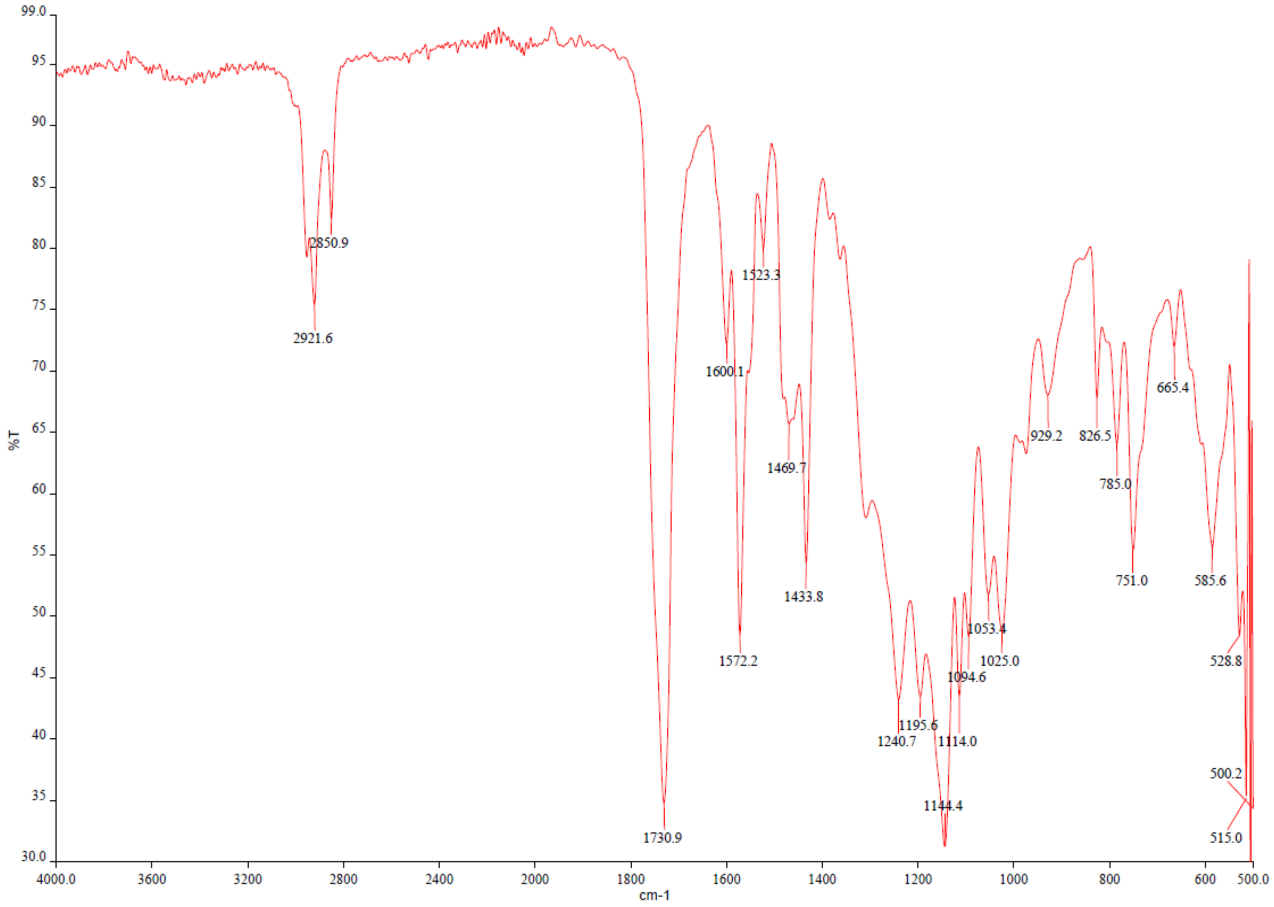

Figure S93. IR (neat) spectrum of **5**.

Mass Spectrometry Core Facility

Lacour Group – University of Geneva

UNIVERSITÉ DE GENÈVE
FACULTÉ DES SCIENCES

ESI-HRMS – Certificate of Analysis

Applicant:	Bibiana Fabri	Date of certificate:	17/05/21
Sample name:	BF-tris(-)-S	Instrument:	Xevo G2 Tof (TOF)
Folder:	170521.PRO	Mobile phase:	MeOH (100 µl/min)
Analyst:	Stéphane Grass	Ionisation mode:	ESI (positive polarity)

Elemental Formula	Ion type	*Masslynx values **** calc. m/z	*Masslynx values **** meas. m/z	Calc. *m/z*	Meas. *m/z*	Accuracy [a] (ppm)
$C_{39}H_{40}N_2O_{14}$	$[M+H]^+$	*761.2558*	*761.2577*	761.2553	761.2572	2.5

[a] *Mass spectrum is calibrated by the use of the MS lockspray system (LeuEnk calibration solution).*

**** MassLynx software does not take into account the mass of the electron for ionic species, therefore the shift of m/z 0.000459.*

Zoomed mass spectrum – Isotopic distribution.

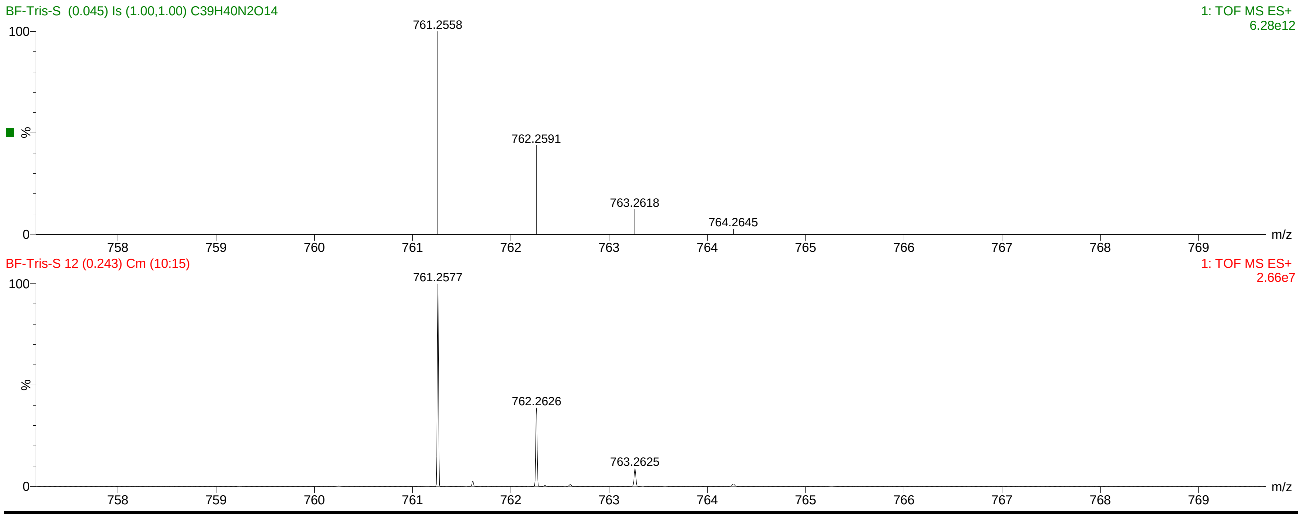

Figure S94. HRMS analysis (ESI, CH_3OH) report of **5**.

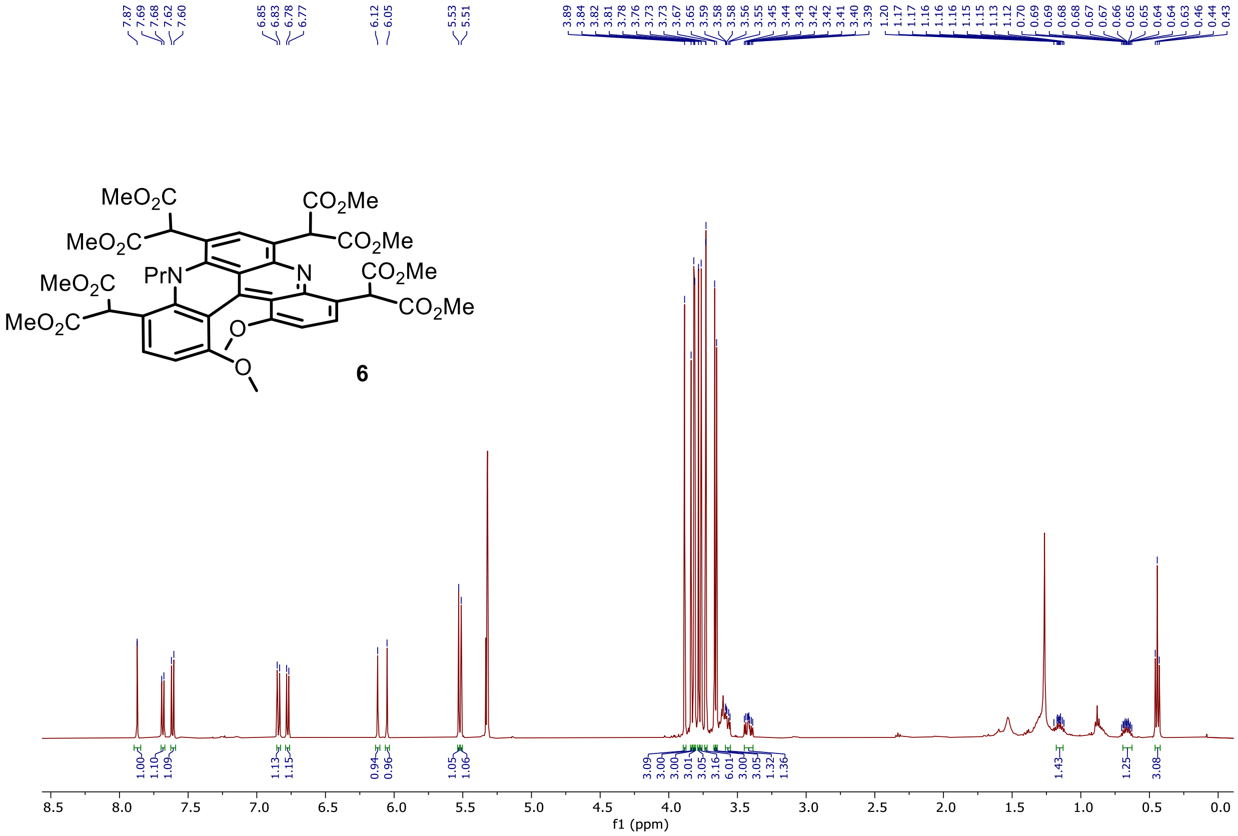

Figure S95. ^{1}H NMR (500 MHz, CD_2Cl_2) spectrum of **6**.

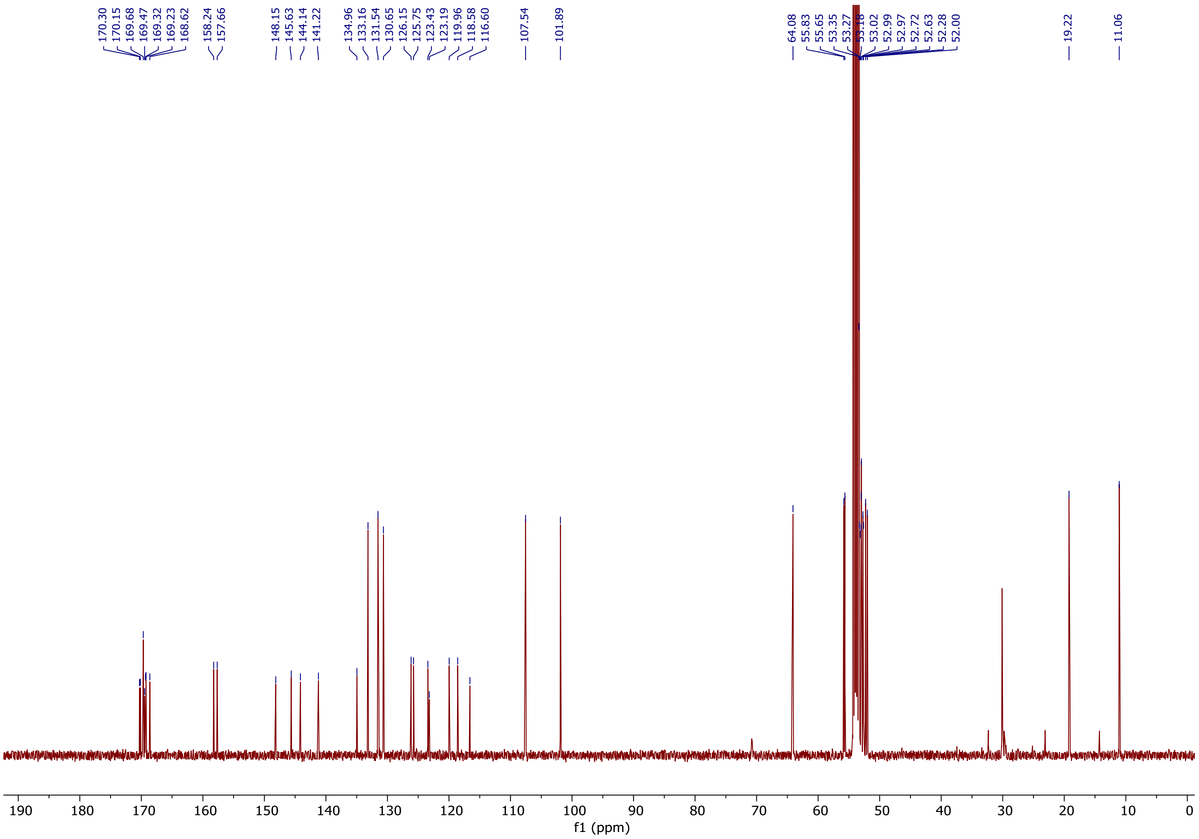

Figure S96. ^{13}C NMR (126 MHz, CD_2Cl_2) spectrum of **6**.

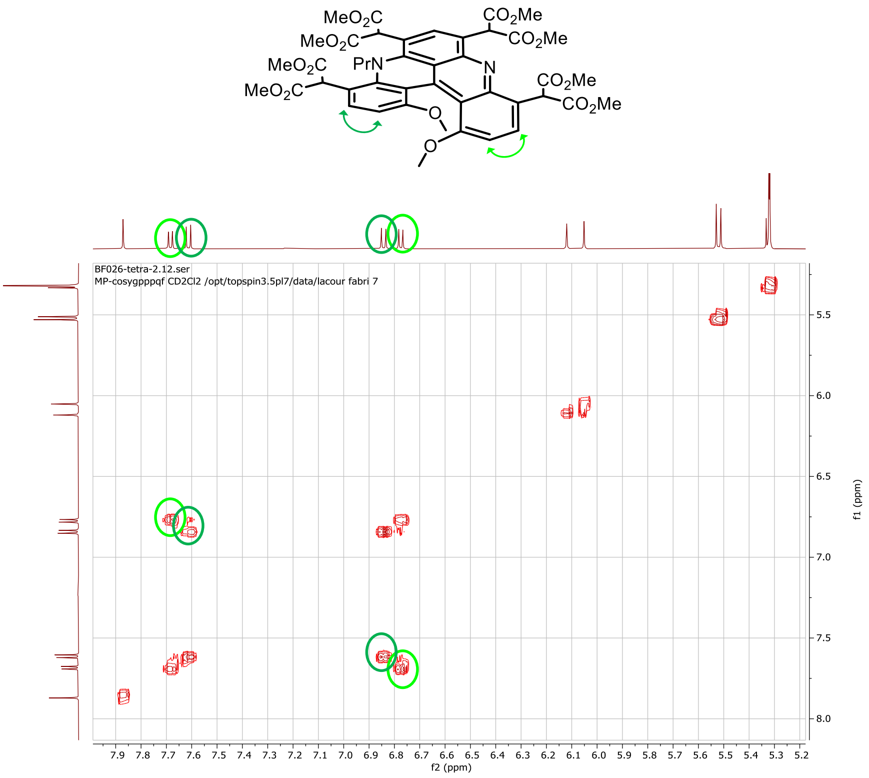

Figure S97. COSY (500 MHz, CD_2Cl_2) analysis (solution state conformation) of **6**.

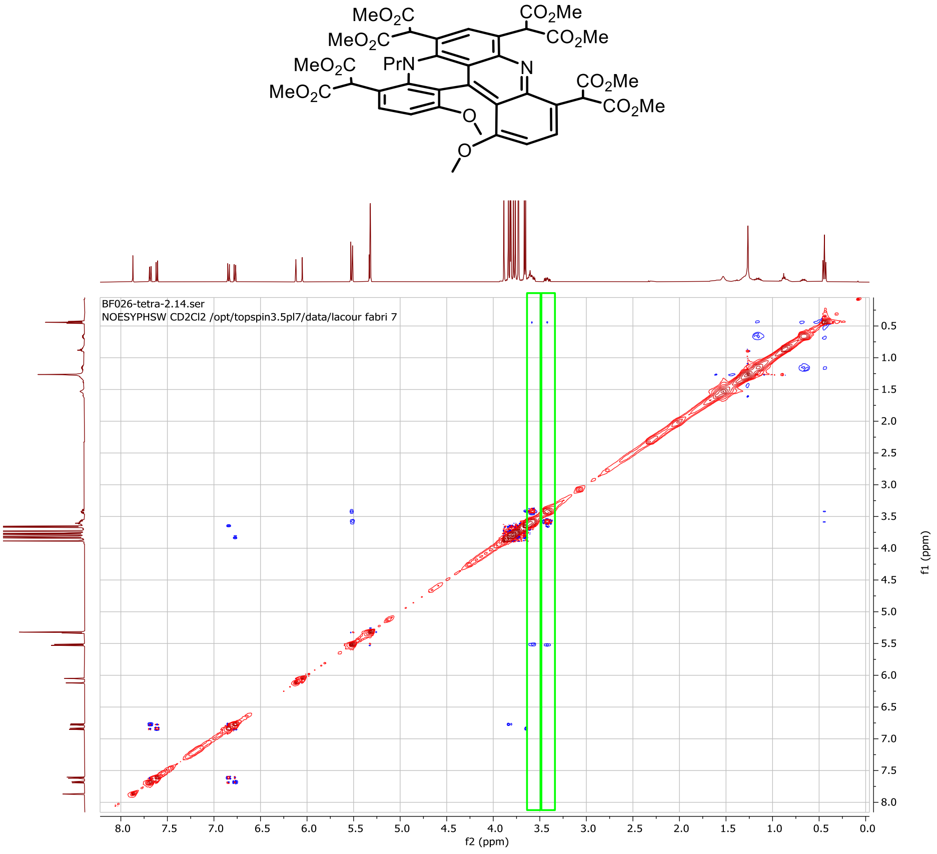

Figure S98. NOESY (500 MHz, CD_2Cl_2) analysis (solution state conformation) of **6**. No coupling through space between the propyl chain protons and aromatic protons is observed.

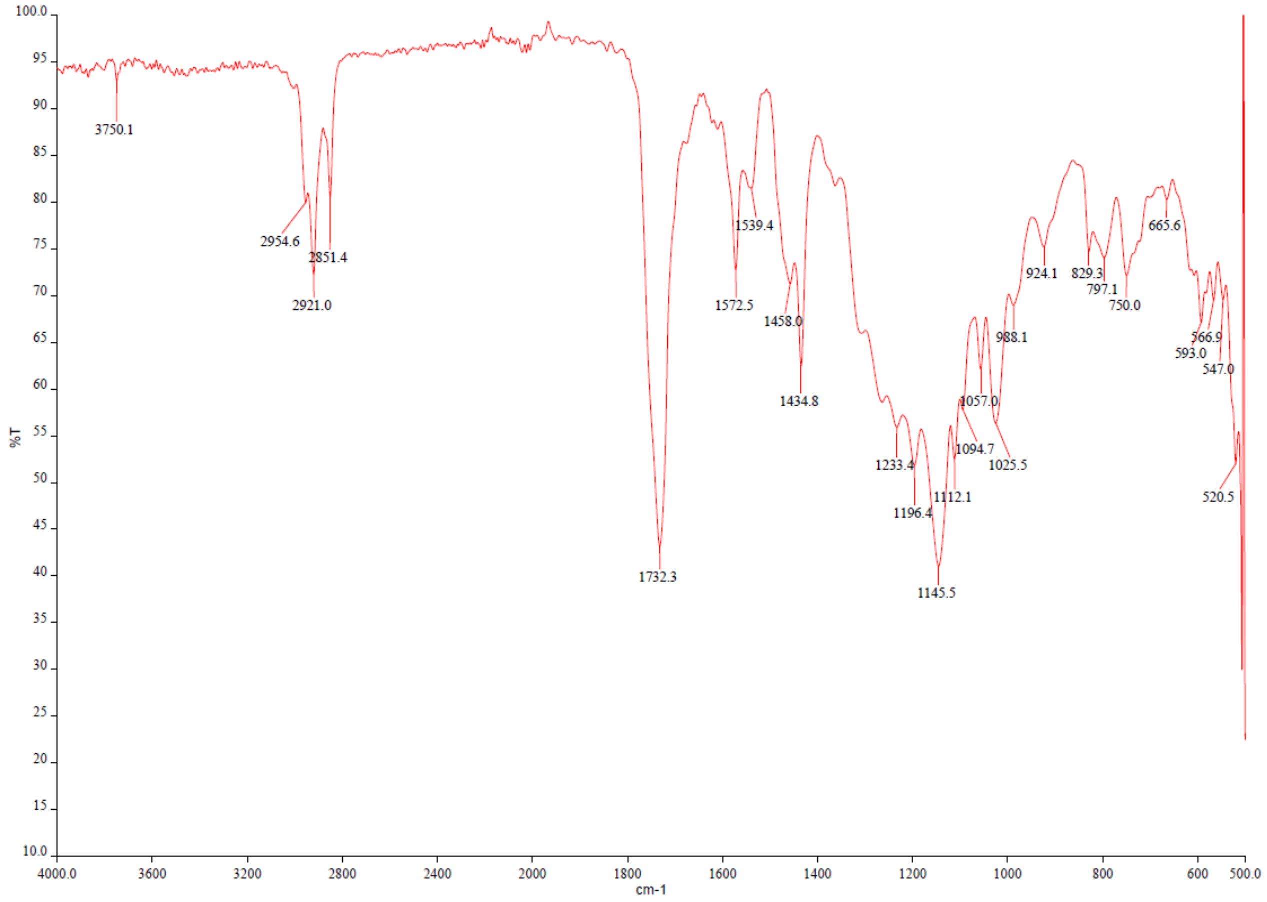

Figure S99. IR (neat) spectrum of **6**.

Mass Spectrometry Core Facility

Lacour Group – University of Geneva

UNIVERSITÉ DE GENÈVE
FACULTÉ DES SCIENCES

ESI-HRMS – Certificate of Analysis

Applicant:	Bibiana Fabri	Date of certificate:	17/05/21
Sample name:	BF-tetra-2	Instrument:	Xevo G2 Tof (TOF)
Folder:	170521.PRO	Mobile phase:	MeOH (100 µl/min)
Analyst:	Stéphane Grass	Ionisation mode:	ESI (positive polarity)

Elemental Formula	Ion type	*Masslynx values **** *calc. m/z*	*meas. m/z*	Calc. *m/z*	Meas. *m/z*	Accuracy [a] *(ppm)*
$C_{44}H_{46}N_2O_{18}$	$[M+H]^+$	*891.2824*	*891.2762*	891.2819	891.2757	-7.0

[a] *Mass spectrum is calibrated by the use of the MS lockspray system (LeuEnk calibration solution).*

**** MassLynx software does not take into account the mass of the electron for ionic species, therefore the shift of m/z 0.000459.*

Zoomed mass spectrum – Isotopic distribution.

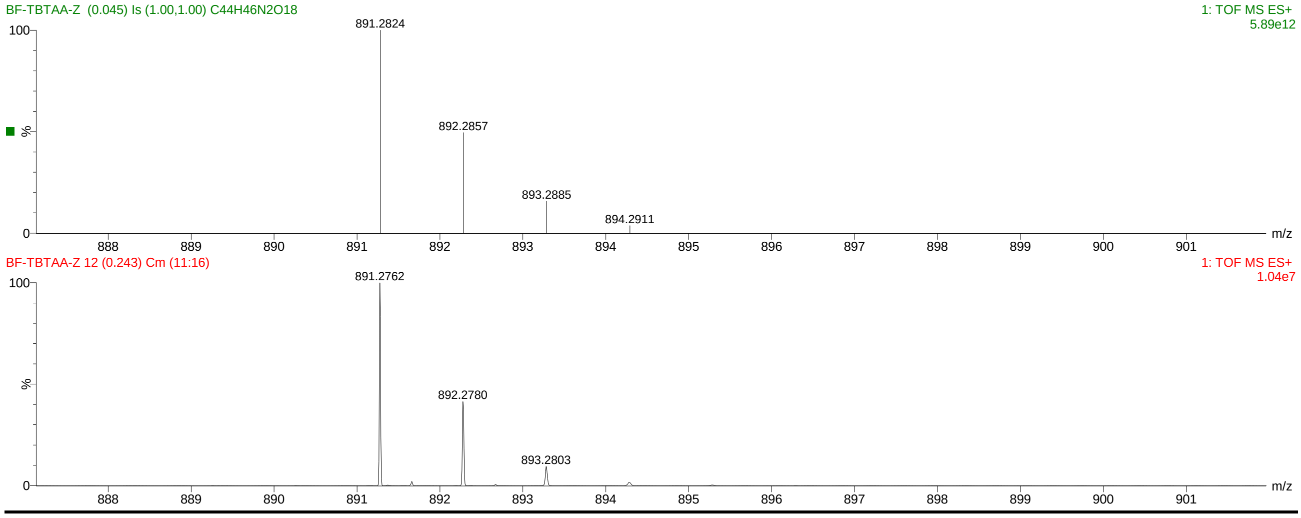

Figure S100. HRMS analysis (ESI, CH_3OH) report of **6**.

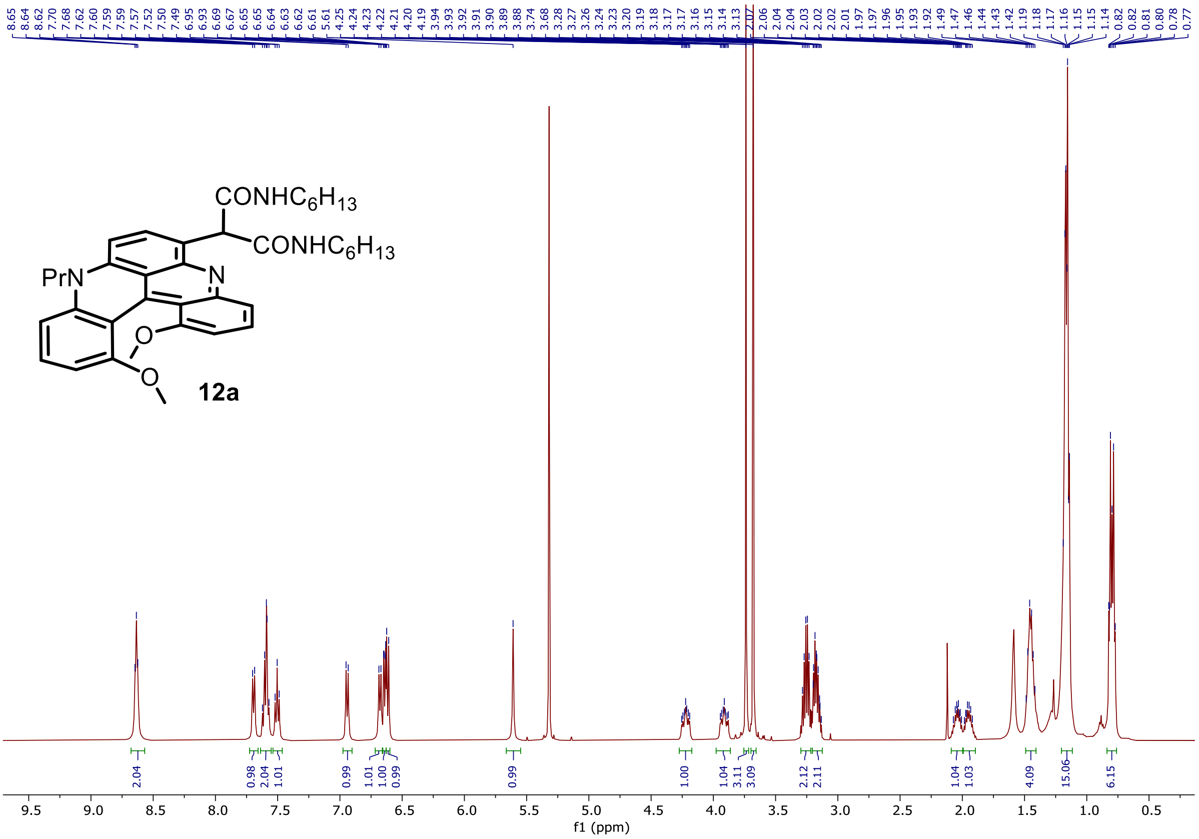

Figure S101. ^{1}H NMR (500 MHz, CD_2Cl_2) spectrum of **12a**.

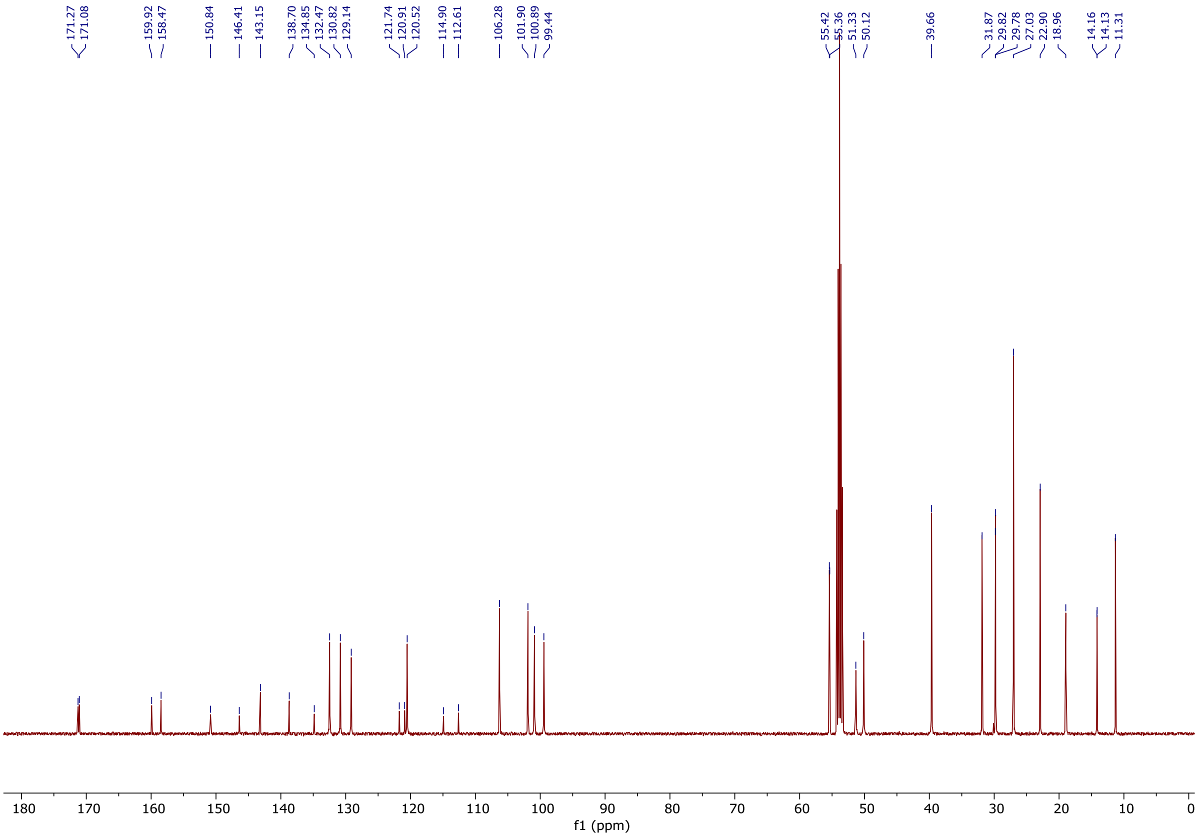

Figure S102. ^{13}C NMR (126 MHz, CD_2Cl_2) spectrum of **12a**.

PerkinElmer Spectrum IR Version 10.7.2
Friday, 1 July 2022 18:10

Analyst User
Date Friday, 1 July 2022 18:10

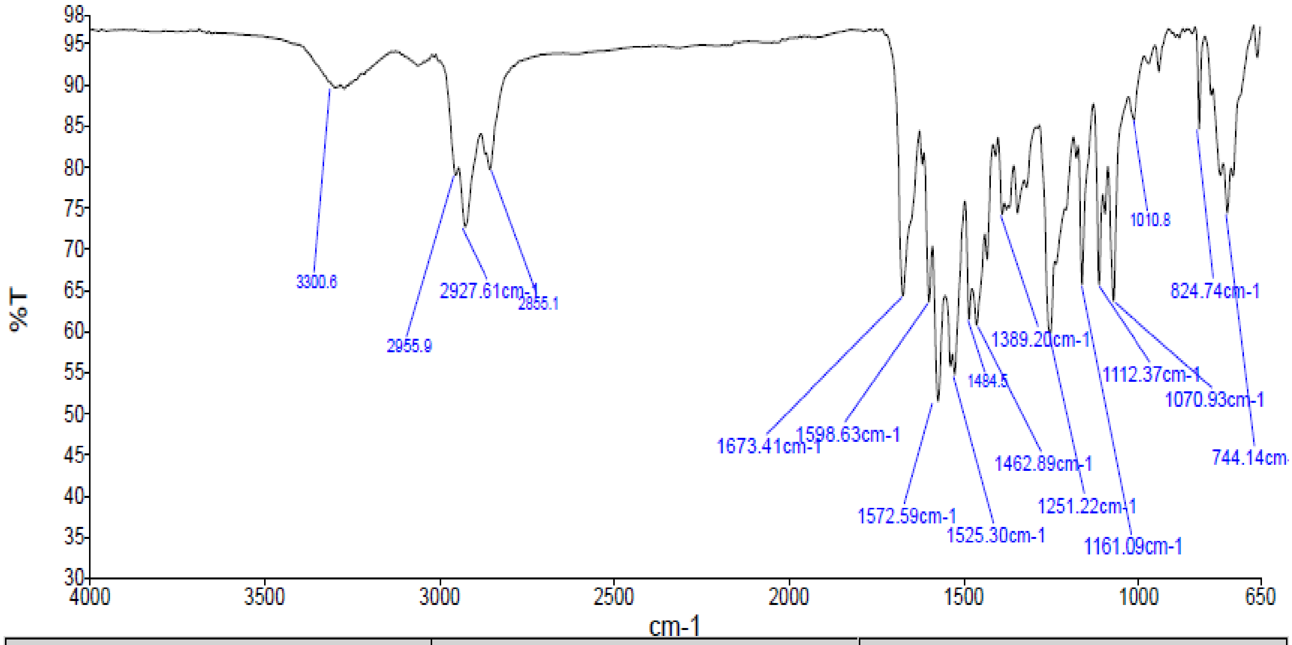

Sample Name	Description	Quality Checks
YN3070F1_1_1	Friday, 1 July 2022	The Quality Checks do not report any warnings for the sample.

Figure S103. IR (neat) spectrum of **12a**.

Mass Spectrometry Core Facility

Lacour Group – University of Geneva

UNIVERSITÉ DE GENÈVE
FACULTÉ DES SCIENCES

ESI-HRMS – Certificate of Analysis

Applicant:	Yana Nikolova	Date of certificate:	01/07/22
Sample name:	YN3-70-F1	Instrument:	Xevo G2 Tof (TOF)
Folder:	010722.PRO	Mobile phase:	MeOH (100 µl/min)
Analyst:	Stéphane Grass	Ionisation mode:	ESI (positive polarity)

Ion type	*Masslynx values **** *calc. m/z*	*meas. m/z*	Calc. *m/z*	Meas. *m/z*	Accuracy [a] *(ppm)*
$[M+H]^+$	*639.3911*	*639.3937*	639.3906	639.3932	4.1

[a] *Mass spectrum is calibrated by the use of the MS lockspray system (LeuEnk calibration solution).*

**** MassLynx software does not take into account the mass of the electron for ionic species, therefore the shift of m/z 0.000459.*

Zoomed mass spectrum – Isotopic distribution.

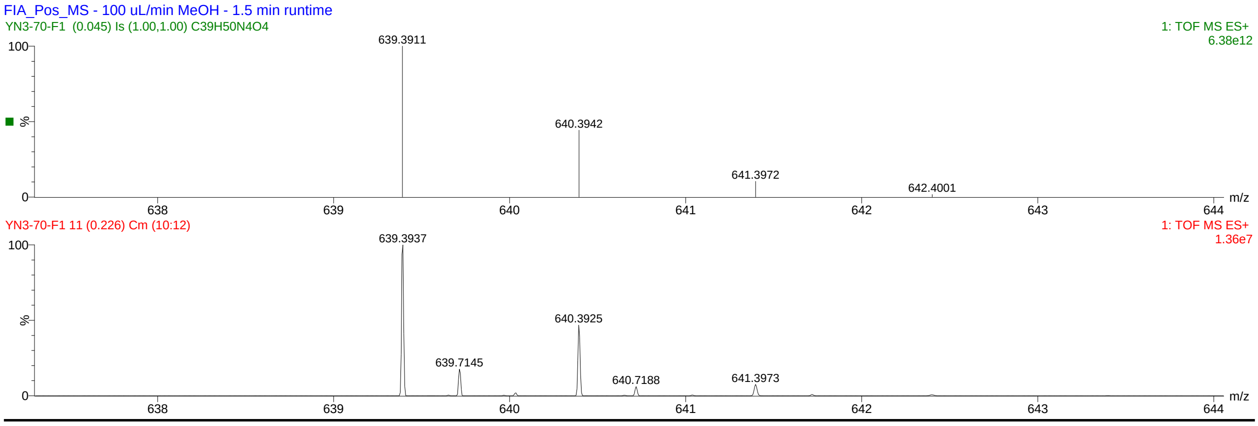

Figure S104. HRMS analysis (ESI, CH_3OH) report of **12a**.

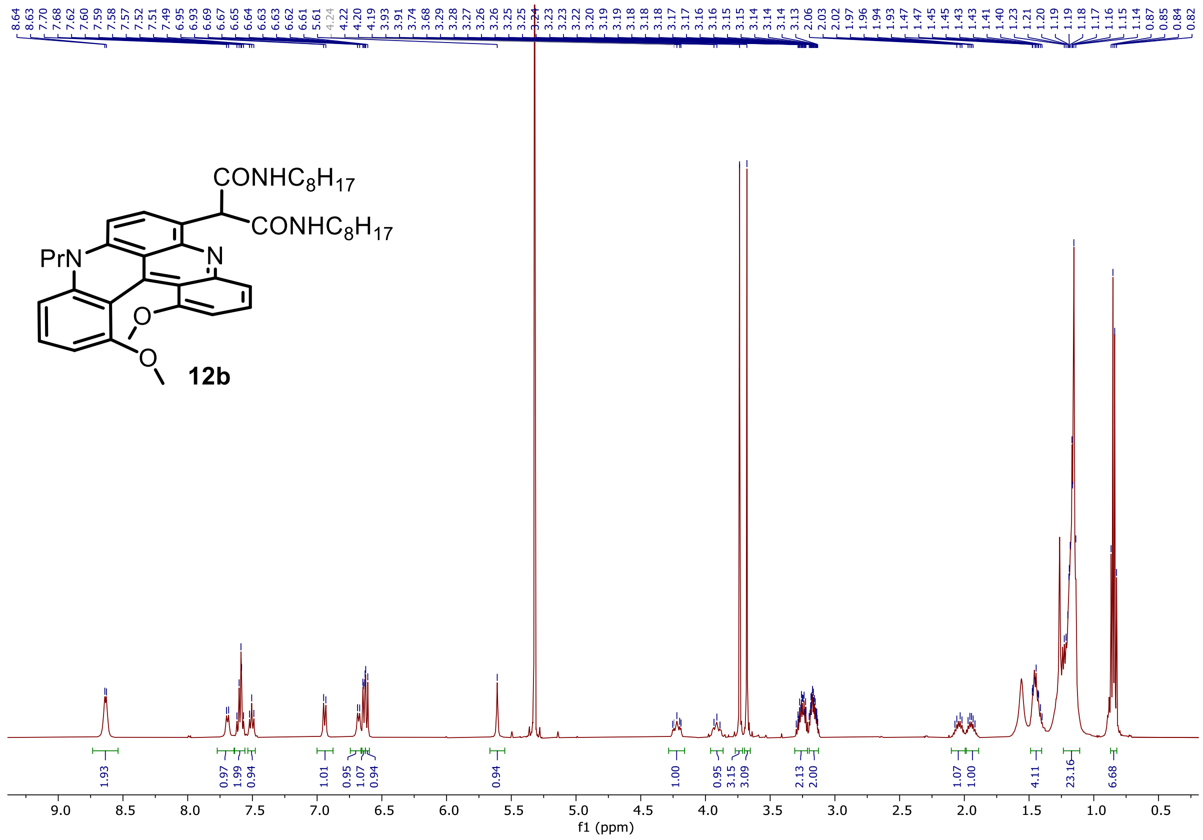

Figure S105. ^{1}H NMR (500 MHz, CD_2Cl_2) spectrum of **12b**.

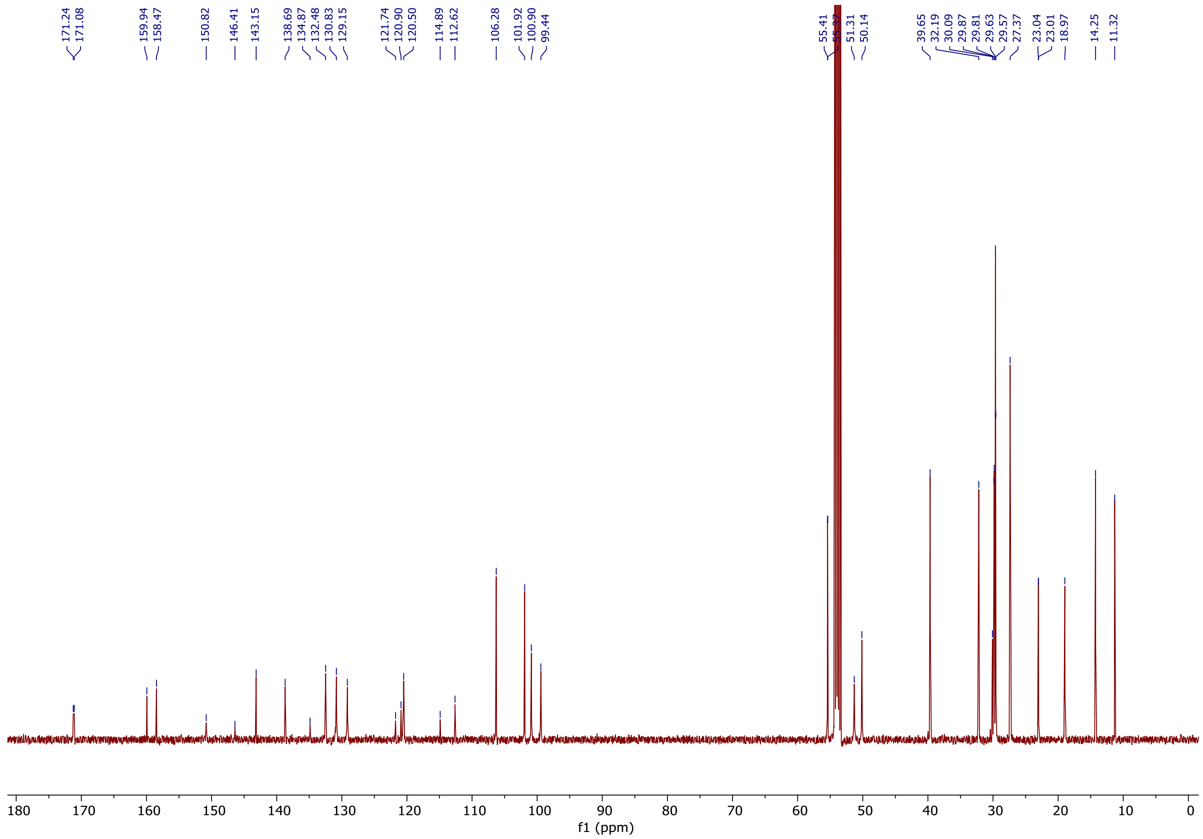

Figure S106. ^{13}C NMR (126 MHz, CD_2Cl_2) spectrum of **12b**.

PerkinElmer Spectrum IR Version 10.7.2
Friday, 1 July 2022 18:07

Analyst	User
Date	Friday, 1 July 2022 18:07

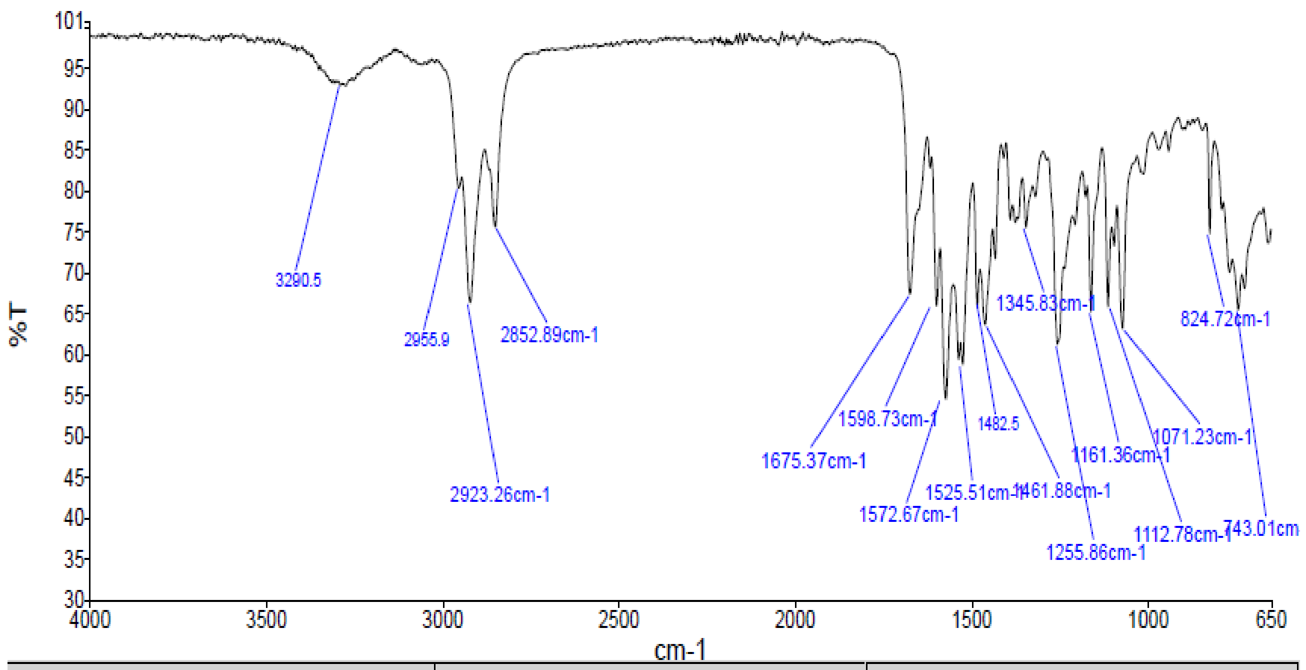

Sample Name	Description	Quality Checks
YN3073F1	Friday, 1 July 2022	The Quality Checks do not report any warnings for the sample.

Figure S107. IR (neat) spectrum of **12b**.

Mass Spectrometry Core Facility

Lacour Group – University of Geneva

UNIVERSITÉ DE GENÈVE
FACULTÉ DES SCIENCES

ESI-HRMS – Certificate of Analysis

Applicant:	Yana Nikolova	Date of certificate:	01/07/22
Sample name:	YN3-73-F1	Instrument:	Xevo G2 Tof (TOF)
Folder:	010722.PRO	Mobile phase:	MeOH (100 μl/min)
Analyst:	Stéphane Grass	Ionisation mode:	ESI (positive polarity)

Elemental Formula	Ion type	*Masslynx values **** *calc. m/z*	*meas. m/z*	Calc. *m/z*	Meas. *m/z*	Accuracy [a] *(ppm)*
$C_{43}H_{58}N_4O_4$	$[M+H]^+$	*695.4536*	*695.4531*	695.4531	695.4526	-0.7

[a] *Mass spectrum is calibrated by the use of the MS lockspray system (LeuEnk calibration solution).*

**** MassLynx software does not take into account the mass of the electron for ionic species, therefore the shift of m/z 0.000459.*

Zoomed mass spectrum – Isotopic distribution.

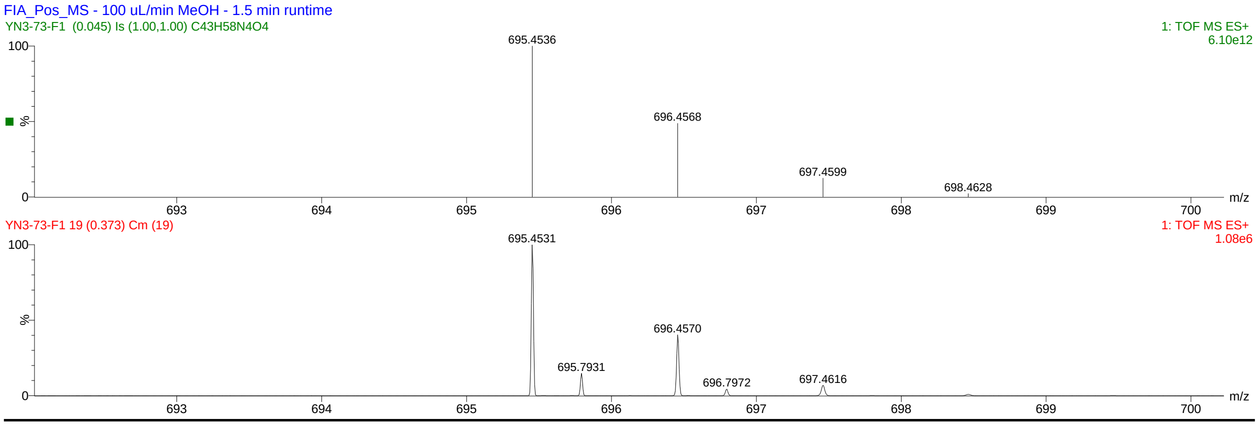

Figure S108. HRMS analysis (ESI, CH_3OH) report of **12b**.

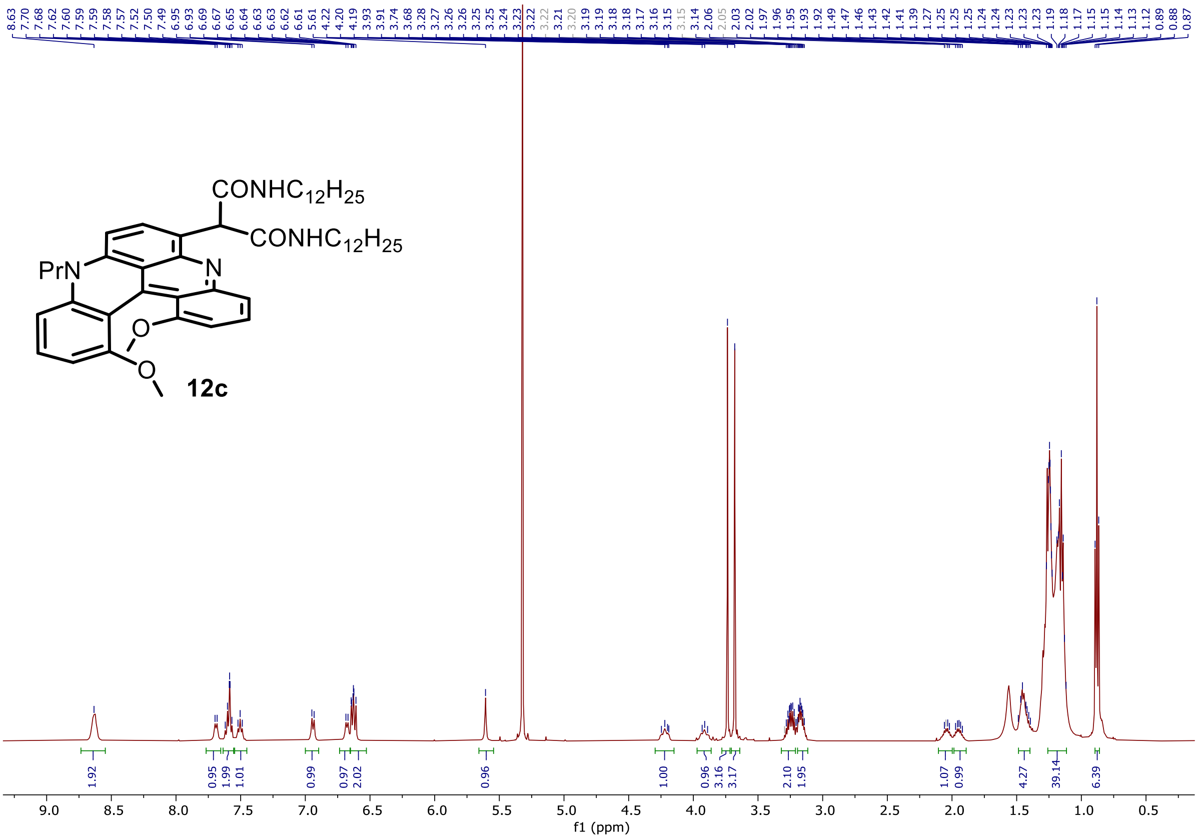

Figure S109. ^{1}H NMR (500 MHz, CD_2Cl_2) spectrum of **12c**.

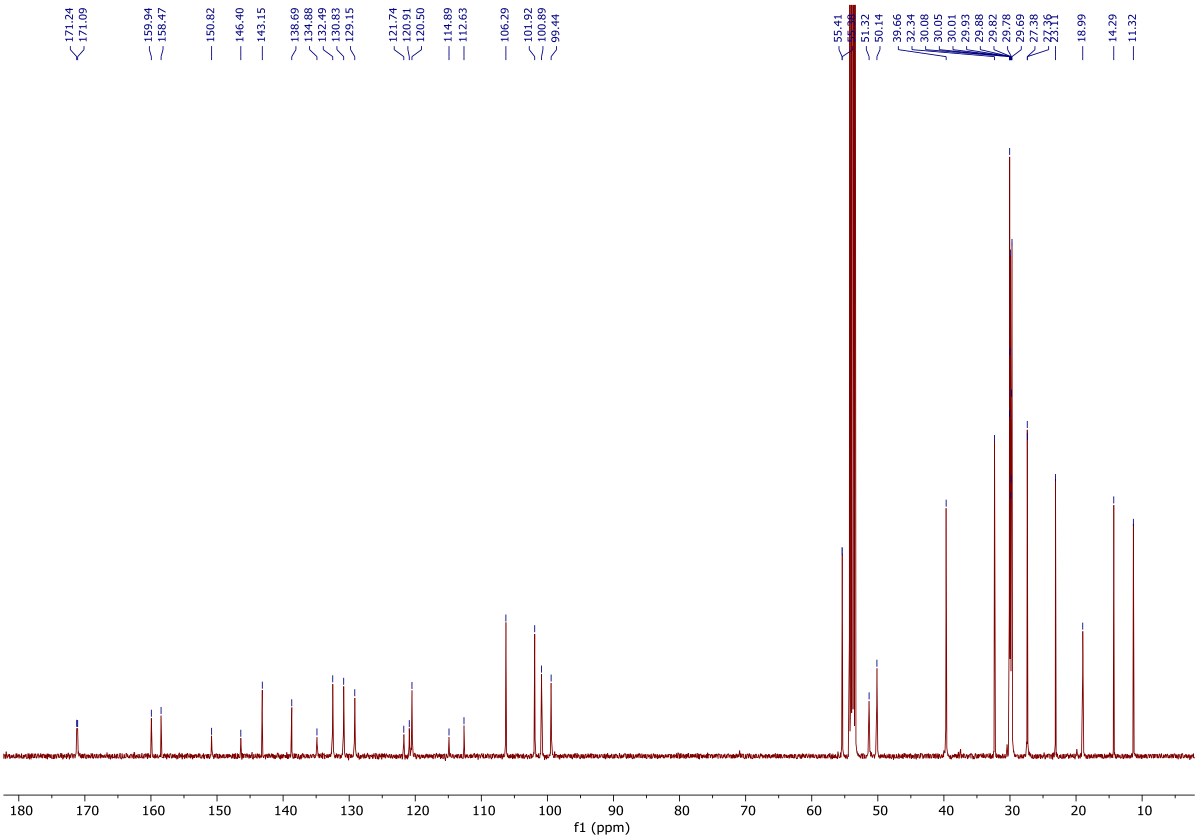

Figure S110. ^{13}C NMR (126 MHz, CD_2Cl_2) spectrum of **12c**.

PerkinElmer Spectrum IR Version 10.7.2
Friday, 1 July 2022 18:05

Analyst	User
Date	Friday, 1 July 2022 18:05

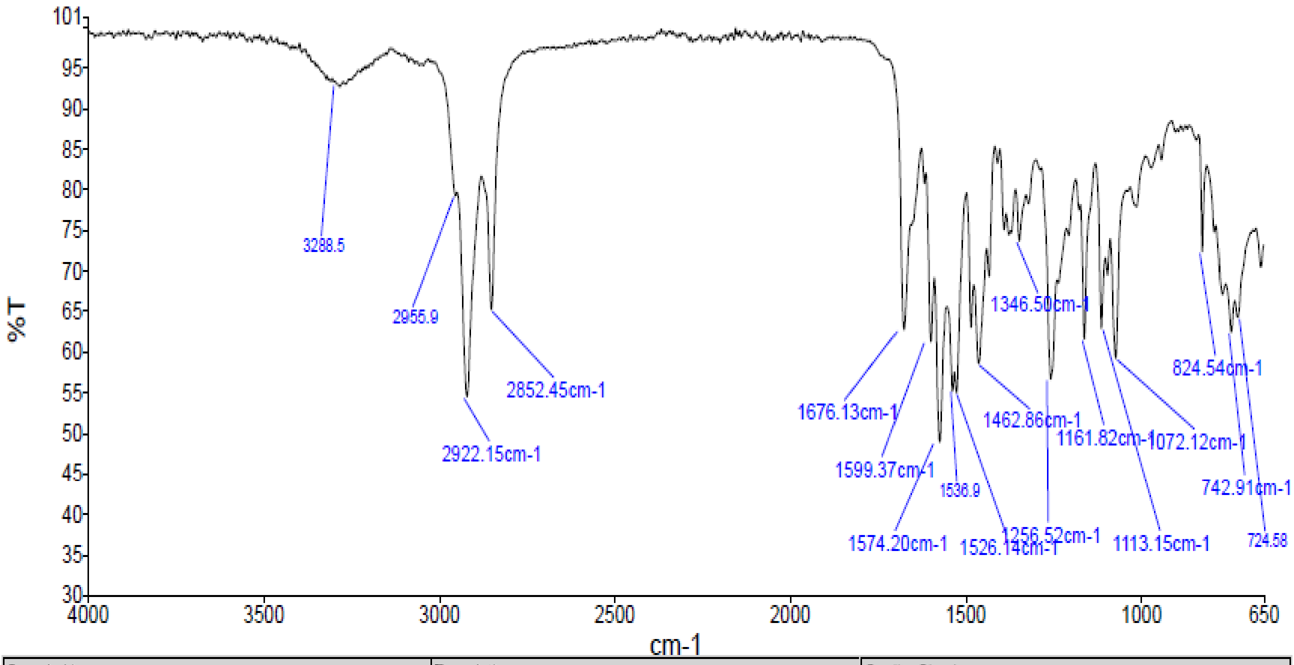

Sample Name	Description	Quality Checks
YN3074F1	Friday, 1 July 2022	The Quality Checks do not report any warnings for the sample.

Figure S111. IR (neat) spectrum of **12c**.

Mass Spectrometry Core Facility

Lacour Group – University of Geneva

UNIVERSITÉ DE GENÈVE
FACULTÉ DES SCIENCES

ESI-HRMS – Certificate of Analysis

Applicant:	Yana Nikolova	Date of certificate:	01/07/22
Sample name:	YN3-74-F1	Instrument:	Xevo G2 Tof (TOF)
Folder:	010722.PRO	Mobile phase:	MeOH (100 µl/min)
Analyst:	Stéphane Grass	Ionisation mode:	ESI (positive polarity)

Elemental Formula	Ion type	*Masslynx values **** *calc. m/z*	*meas. m/z*	Calc. *m/z*	Meas. *m/z*	Accuracy [a] *(ppm)*
$C_{51}H_{74}N_4O_4$	$[M+H]^+$	*807.5789*	*807.5764*	807.5784	807.5759	-3.1

[a] *Mass spectrum is calibrated by the use of the MS lockspray system (LeuEnk calibration solution).*

**** MassLynx software does not take into account the mass of the electron for ionic species, therefore the shift of m/z 0.000459.*

Zoomed mass spectrum – Isotopic distribution.

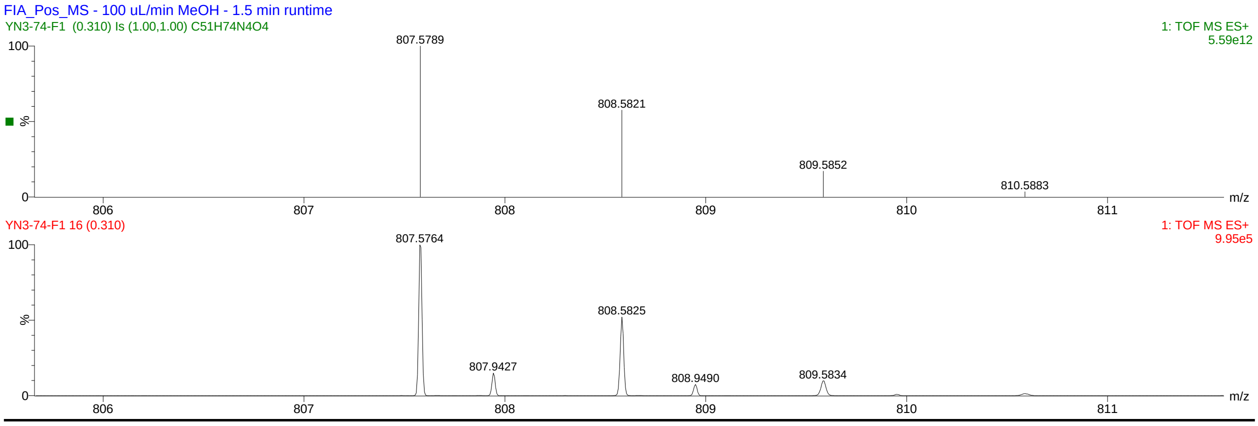

Figure S112. HRMS analysis (ESI, CH_3OH) report of **12c**.

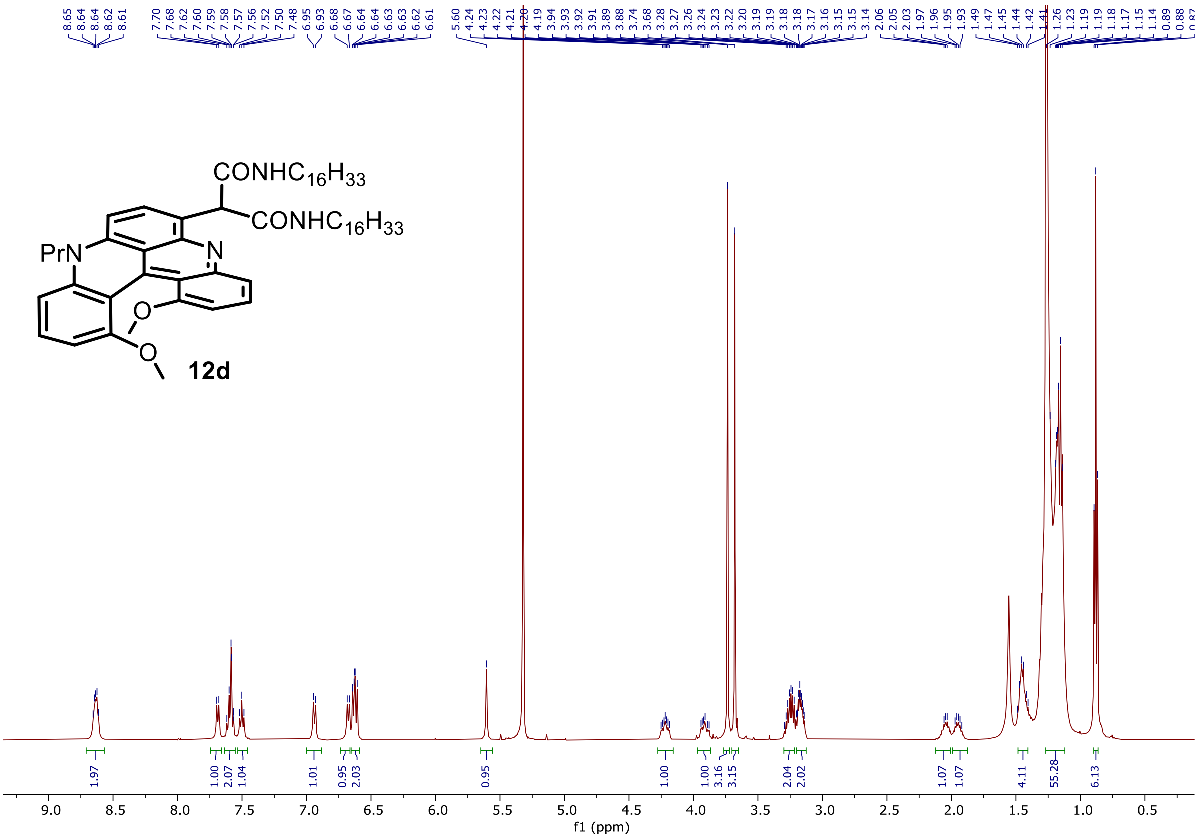

Figure S113. ^{1}H NMR (500 MHz, CD_2Cl_2) spectrum of **12d**.

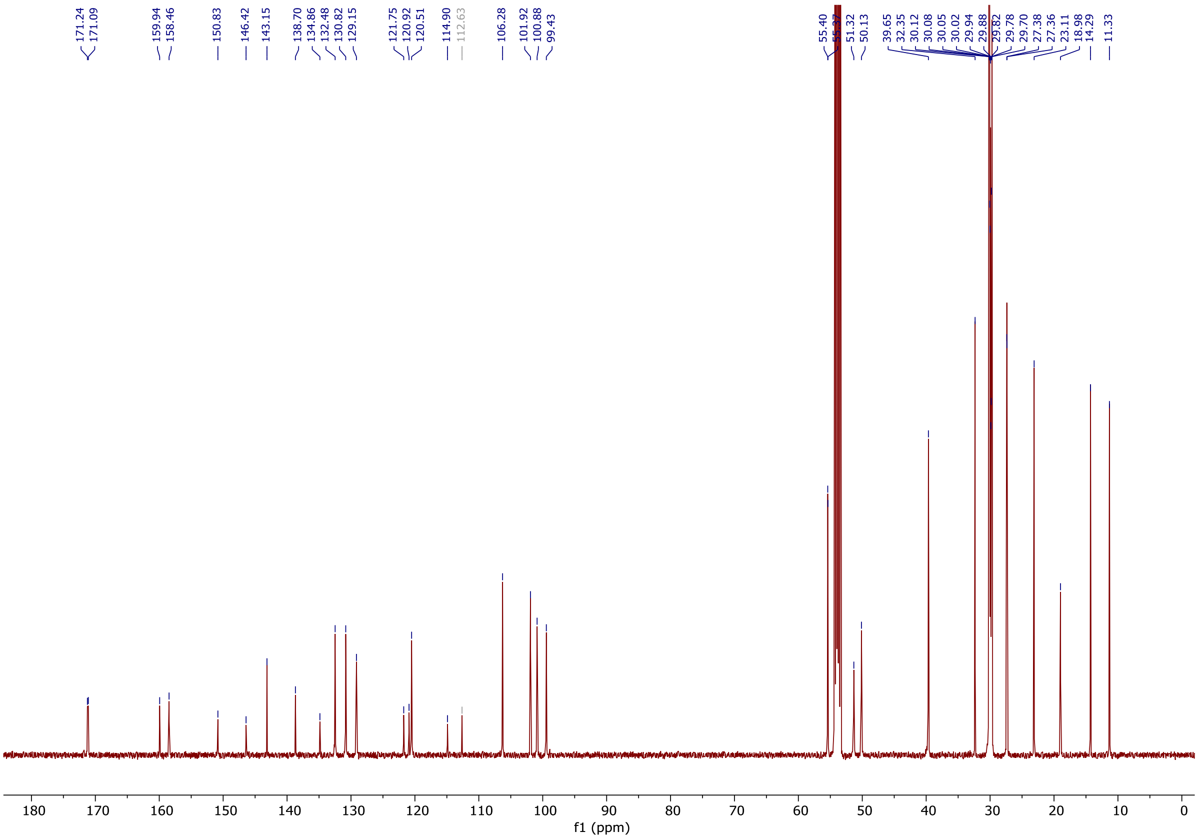

Figure S114. ^{13}C NMR (126 MHz, CD_2Cl_2) spectrum of **12d**.

PerkinElmer Spectrum IR Version 10.7.2
Friday, 1 July 2022 18:02

Analyst	User
Date	Friday, 1 July 2022 18:02

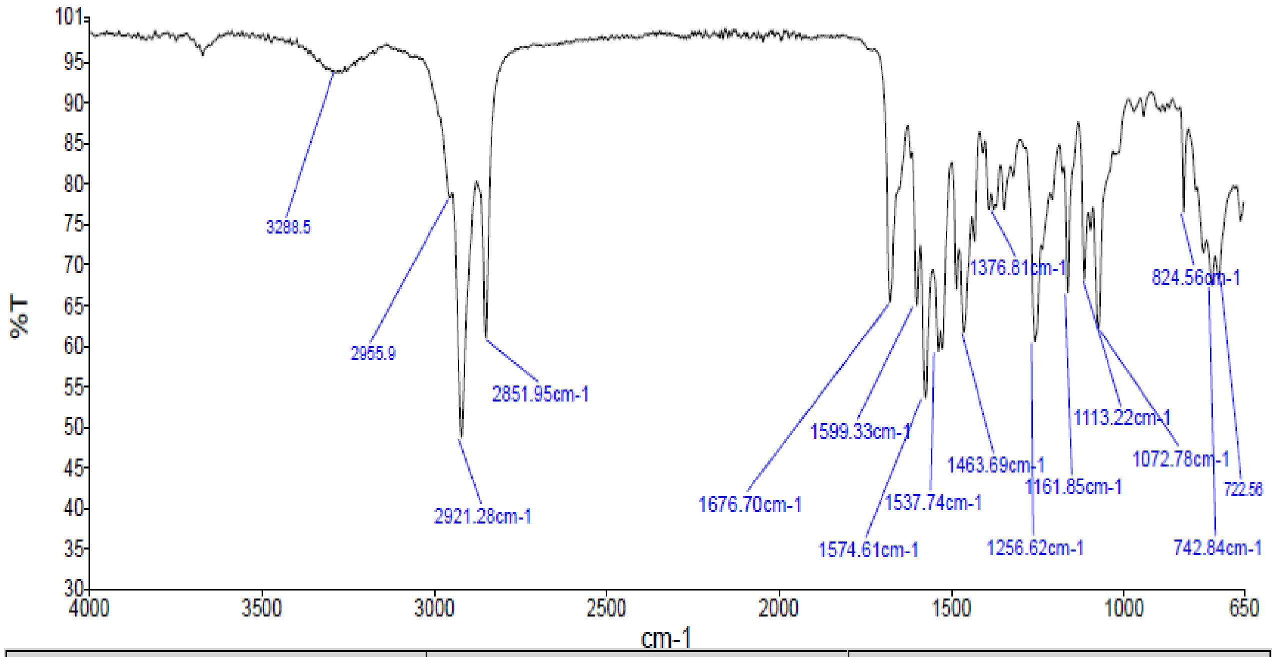

Sample Name	Description	Quality Checks
YN3075F1	Friday, 1 July 2022	The Quality Checks do not report any warnings for the sample.

Figure S115. IR (neat) spectrum of **12d**.

Mass Spectrometry Core Facility

Lacour Group – University of Geneva

UNIVERSITÉ DE GENÈVE
FACULTÉ DES SCIENCES

ESI-HRMS – Certificate of Analysis

Applicant:	Yana Nikolova	Date of certificate:	01/07/22
Sample name:	YN3-75-F1	Instrument:	Xevo G2 Tof (TOF)
Folder:	010722.PRO	Mobile phase:	MeOH (100 µl/min)
Analyst:	Stéphane Grass	Ionisation mode:	ESI (positive polarity)

Elemental Formula	Ion type	*Masslynx values **** *calc. m/z*	*meas. m/z*	Calc. *m/z*	Meas. *m/z*	Accuracy [a] *(ppm)*
$C_{59}H_{90}N_4O_4$	$[M+H]^+$	*919.704*	*919.7054*	919.7035	919.7049	1.5

[a] *Mass spectrum is calibrated by the use of the MS lockspray system (LeuEnk calibration solution).*

**** MassLynx software does not take into account the mass of the electron for ionic species, therefore the shift of m/z 0.000459.*

Zoomed mass spectrum – Isotopic distribution.

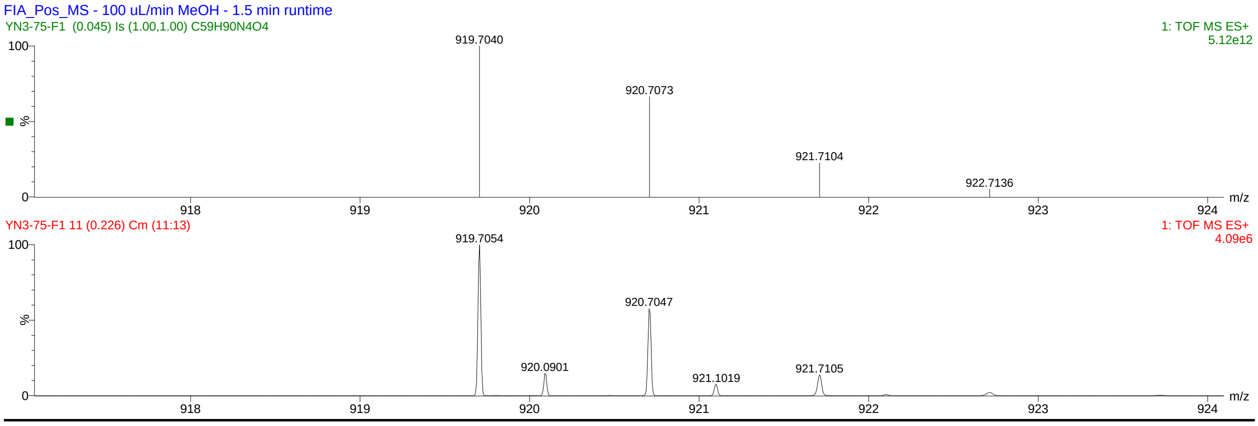

Figure S116. HRMS analysis (ESI, CH_3OH) report of **12d**.

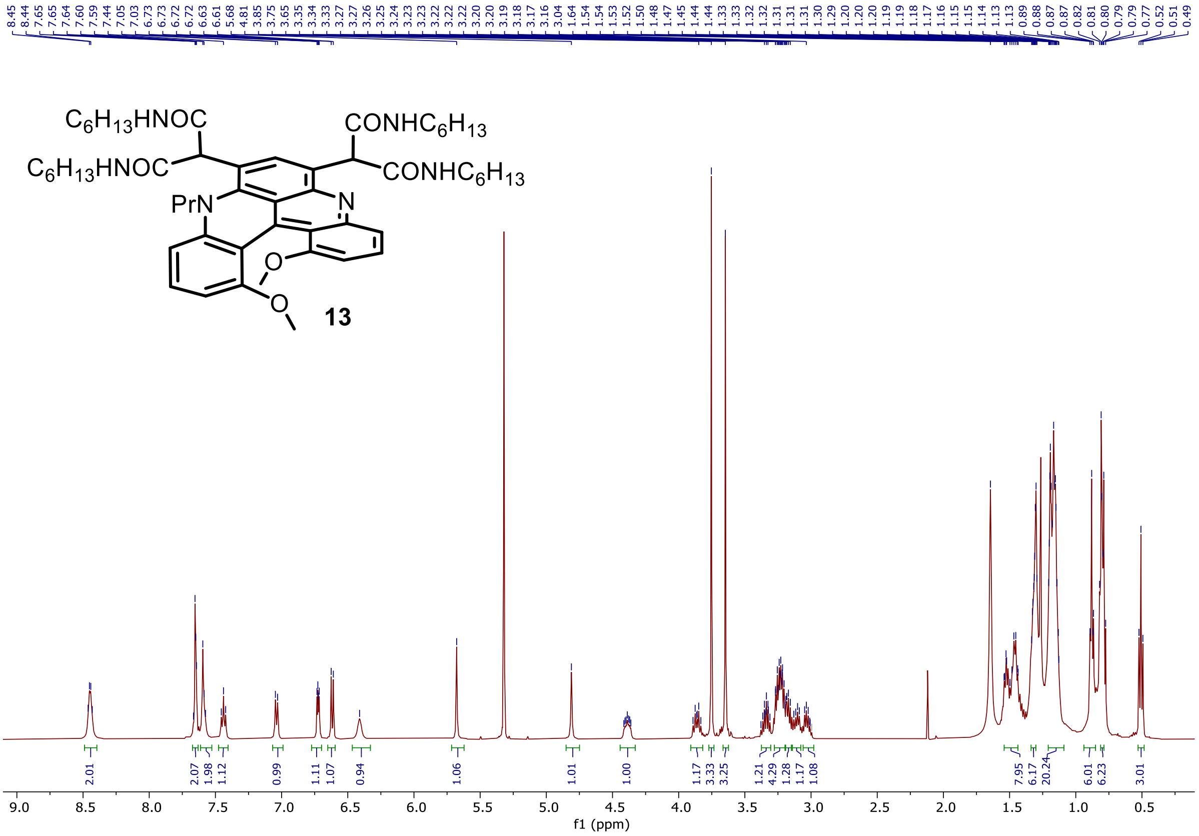

Figure S117. ^{1}H NMR (500 MHz, CD_2Cl_2) spectrum of **13**.

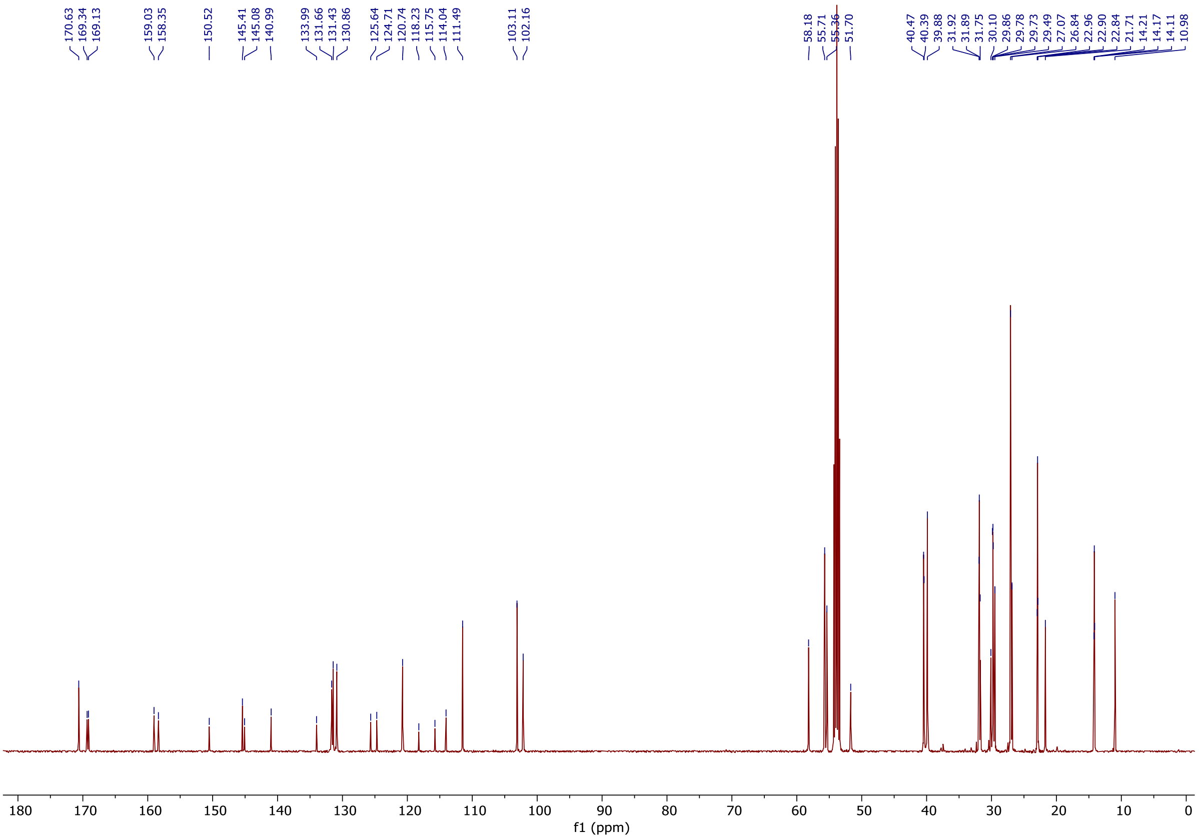

Figure S118. ^{13}C NMR (126 MHz, CD_2Cl_2) spectrum of **13**.

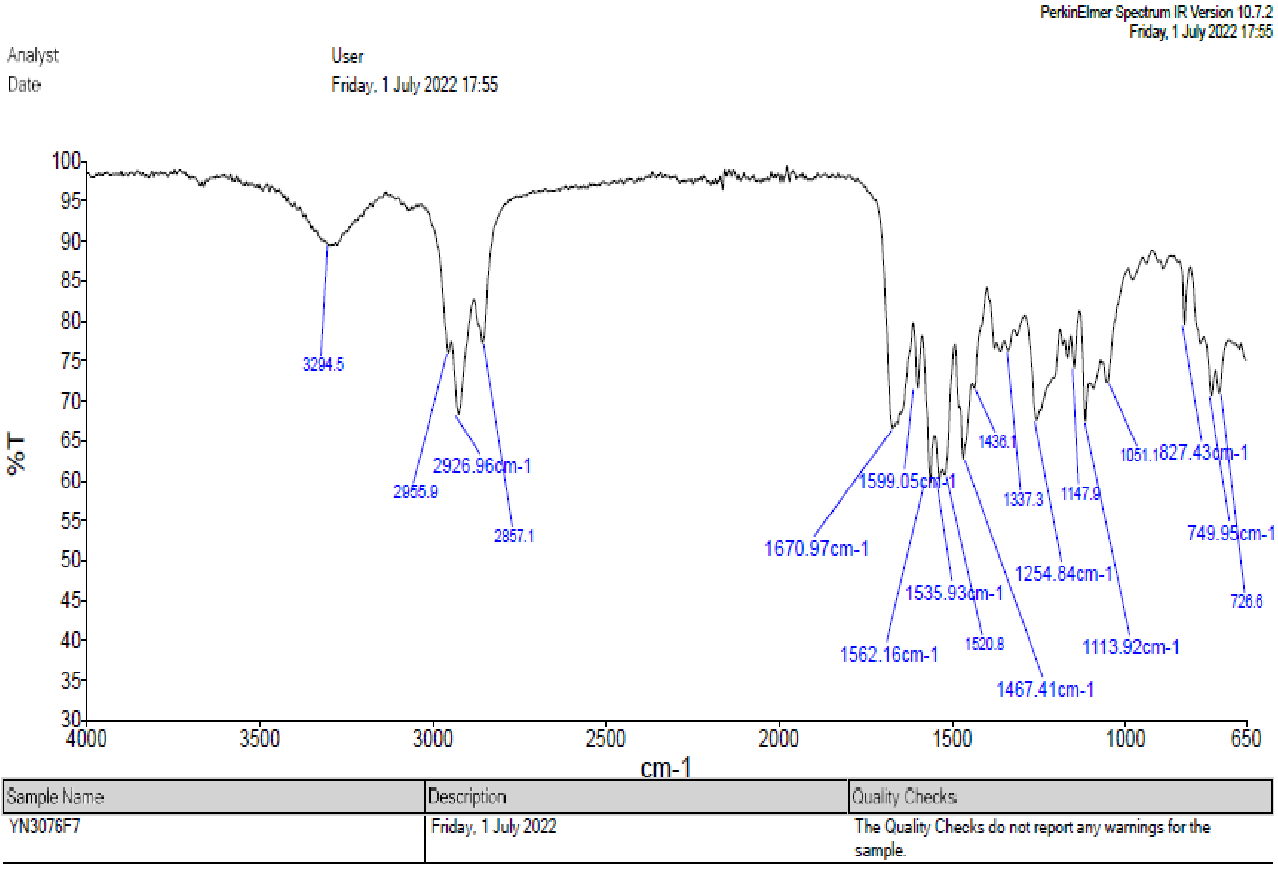

Figure S119. IR (neat) spectrum of **13**.

Mass Spectrometry Core Facility

Lacour Group – University of Geneva

UNIVERSITÉ DE GENÈVE
FACULTÉ DES SCIENCES

ESI-HRMS – Certificate of Analysis

Applicant:	Yana Nikolova	Date of certificate:	01/07/22
Sample name:	YN3-76-F7	Instrument:	Xevo G2 Tof (TOF)
Folder:	010722.PRO	Mobile phase:	MeOH (100 µl/min)
Analyst:	Stéphane Grass	Ionisation mode:	ESI (positive polarity)

Elemental Formula	Ion type	*Masslynx values **** *calc. m/z*	*meas. m/z*	Calc. *m/z*	Meas. *m/z*	Accuracy [a] *(ppm)*
$C_{54}H_{78}N_6O_6$	$[M+H]^+$	*907.6061*	*907.6037*	907.6056	907.6032	-2.6

[a] *Mass spectrum is calibrated by the use of the MS lockspray system (LeuEnk calibration solution).*

**** MassLynx software does not take into account the mass of the electron for ionic species, therefore the shift of m/z 0.000459.*

Zoomed mass spectrum – Isotopic distribution.

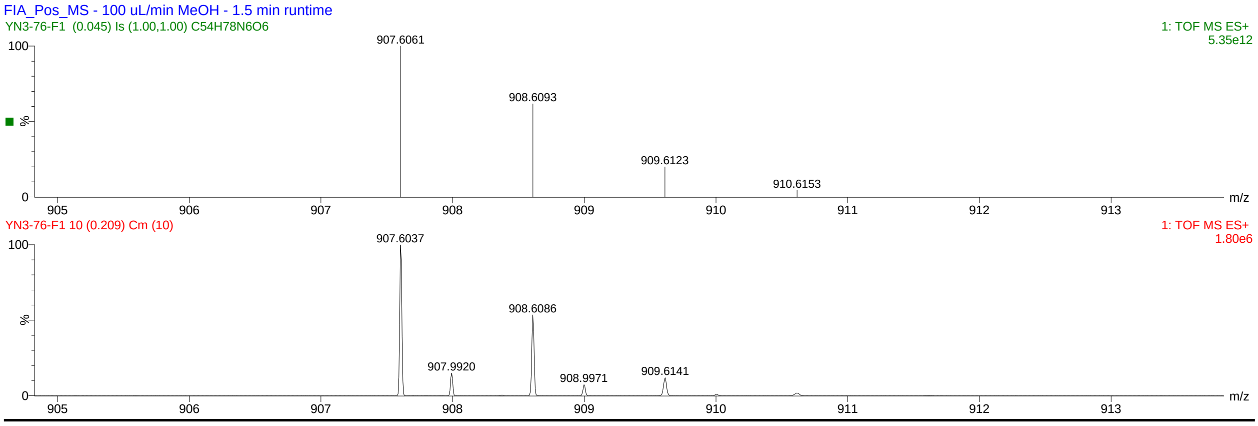

Figure S120. HRMS analysis (ESI, CH_3OH) report of **13**.

XYZ Coordinates

Rh-I4

Energy (POTENTIAL) = -2816.8890314 Eh

Atom	X	Y	Z
O	-2.2428	-1.0279	0.3611
C	-2.9331	-0.2724	-0.3140
C	-2.6237	0.2104	-1.7263
C	-2.5914	-0.9352	-2.7365
O	-2.0159	-0.8978	-3.8026
O	-4.0400	0.2834	0.1698
C	-4.3918	-0.0612	1.5306
H	-5.3079	0.4914	1.7390
H	-4.5622	-1.1376	1.6163
H	-3.5952	0.2431	2.2147
O	-3.3806	-1.9390	-2.3273
C	-3.4526	-3.0958	-3.1801
H	-4.2034	-3.7444	-2.7298
H	-3.7518	-2.8093	-4.1922
H	-2.4838	-3.5977	-3.2057
O	-1.0039	7.2507	-0.2295
O	-0.5255	7.1985	-2.8899
N	-2.7734	3.0257	-1.6243
N	2.0357	3.5539	-1.2431
C	-0.5044	4.7312	-1.4973
C	-1.8033	5.2401	-1.1947
C	-2.0815	6.5514	-0.6657
C	-3.3829	6.9993	-0.5413
H	-3.5936	7.9852	-0.1453
C	-4.4644	6.1443	-0.8779
H	-5.4784	6.5237	-0.7799
C	-4.2498	4.8454	-1.2590
H	-5.0677	4.1575	-1.4496
C	-2.9174	4.3394	-1.3668
C	-1.5386	2.4956	-1.6156
C	-0.3615	3.3264	-1.5428
C	0.9384	2.7240	-1.4697
C	3.2956	2.9563	-0.7737
H	3.0312	2.1210	-0.1189
H	3.8013	3.6897	-0.1403
C	4.2324	2.4784	-1.8919
H	3.7010	1.7711	-2.5384
H	4.5084	3.3284	-2.5264
C	5.4877	1.8215	-1.3127
H	6.1574	1.4803	-2.1100
H	5.2311	0.9511	-0.6951
C	1.9661	4.9095	-1.5532
C	3.1370	5.6936	-1.6523
H	4.1086	5.2772	-1.4248
C	3.0510	7.0009	-2.1025
H	3.9587	7.5922	-2.1870
C	1.8344	7.5537	-2.5180
H	1.8125	8.5447	-2.9537
C	0.6706	6.7973	-2.3990
C	-0.6821	8.5464	-3.3239
H	-0.3805	9.2564	-2.5451
H	-1.7472	8.6665	-3.5319
H	-0.1089	8.7421	-4.2390
C	0.6964	5.5068	-1.7921
C	1.0437	1.3371	-1.5354
H	2.0043	0.8418	-1.4922
C	-0.1085	0.5413	-1.6632
H	0.0176	-0.5318	-1.7243
C	-1.3802	1.0762	-1.7027
C	-1.1899	8.5769	0.2534
H	-0.1871	8.9613	0.4508
H	-1.6832	9.2127	-0.4916
H	-1.7738	8.5859	1.1830
H	6.0482	2.5232	-0.6818
Rh	-0.8327	-2.8627	0.1843
Rh	0.5659	-4.8281	0.1164
O	-1.1428	-5.9326	-0.2786
C	-2.2652	-5.3337	-0.3527
O	-2.4574	-4.0853	-0.1938
C	-3.4675	-6.1801	-0.6924
H	-3.4147	-7.1367	-0.1653
H	-3.4576	-6.3885	-1.7692
H	-4.3935	-5.6596	-0.4401
O	0.2409	-5.0345	2.1488
C	-0.4906	-4.1828	2.7516
O	-1.0731	-3.1848	2.2150
C	-0.6621	-4.3482	4.2415
H	-1.6874	-4.1040	4.5322
H	0.0099	-3.6470	4.7514
H	-0.4117	-5.3651	4.5506
O	0.8759	-1.7533	0.5559
C	2.0029	-2.3444	0.6037
O	2.1949	-3.5987	0.4880
C	3.2258	-1.4776	0.7821
H	3.7331	-1.3846	-0.1859
H	2.9466	-0.4830	1.1354
H	3.9233	-1.9471	1.4815
O	-0.4940	-2.6735	-1.8396
C	0.2581	-3.5041	-2.4491

O	0.8242	-4.5120	-1.9190
C	0.5091	-3.2477	-3.9130
H	0.4933	-4.1897	-4.4684
H	-0.2314	-2.5504	-4.3079
H	1.5086	-2.8092	-4.0237
H	-3.4624	0.8458	-2.0318

Rh-I1

Energy (POTENTIAL) = -2816.8469098 Eh

Atom	X	Y	Z
O	-2.6033	-0.3439	2.5643
C	-1.7407	-0.2280	1.7124
C	-1.8281	-0.4310	0.2476
C	-3.1605	-0.3792	-0.4074
O	-3.3217	-0.1348	-1.6006
O	-0.4602	0.1324	2.0565
C	-0.2191	0.2957	3.4539
H	0.8314	0.5829	3.5415
H	-0.8561	1.0786	3.8797
H	-0.3962	-0.6404	3.9942
O	-4.2032	-0.6774	0.3935
C	-5.4618	-0.8104	-0.2746
H	-6.1673	-1.1403	0.4913
H	-5.7898	0.1469	-0.6948
H	-5.3981	-1.5521	-1.0741
O	-0.3935	6.0143	2.7040
O	0.1341	6.7833	0.1881
N	-2.1302	2.4145	0.0759
N	2.6280	2.7638	0.5635
C	0.1172	4.0028	0.7154
C	-1.1861	4.4103	1.1529
C	-1.4620	5.4841	2.0660
C	-2.7719	5.8682	2.3122
H	-2.9860	6.6895	2.9851
C	-3.8431	5.1603	1.7271
H	-4.8594	5.4833	1.9350
C	-3.6188	4.0366	0.9618
H	-4.4303	3.4289	0.5754
C	-2.2917	3.6150	0.7096
C	-0.9287	1.9143	-0.0704
C	0.2496	2.6803	0.2392
C	1.5392	2.0734	0.1464
C	3.9173	2.0544	0.7735
H	3.6818	1.0420	1.1038
H	4.4149	2.5369	1.6156
C	4.8206	2.0304	-0.4646
H	4.2906	1.5490	-1.2944
H	5.0352	3.0557	-0.7846
C	6.1231	1.2845	-0.1651
H	6.7705	1.2632	-1.0484
H	5.9266	0.2471	0.1323
C	2.5774	4.1580	0.7381
C	3.7603	4.9054	0.9080
H	4.7259	4.4280	0.9880
C	3.6916	6.2883	0.9224
H	4.6055	6.8613	1.0485
C	2.4852	6.9650	0.7067
H	2.4805	8.0453	0.6318
C	1.3116	6.2357	0.5488
C	0.0022	8.2048	0.1695
H	0.2718	8.6416	1.1375
H	-1.0518	8.3970	-0.0382
H	0.6190	8.6498	-0.6208
C	1.3179	4.8133	0.6981
C	1.6571	0.7261	-0.3425
H	2.6346	0.2891	-0.4904
C	0.5630	-0.0020	-0.6572
H	0.6957	-0.9938	-1.0627
C	-0.8352	0.4769	-0.5492
C	-0.5906	7.1036	3.6040
H	0.4084	7.3942	3.9347
H	-1.0715	7.9539	3.1061
H	-1.1899	6.7987	4.4710
H	6.6791	1.7677	0.6479
H	-1.2424	0.4443	-1.5732
Rh	-1.3989	-2.7048	0.0578
Rh	-0.8611	-5.1001	-0.2104
O	-2.8413	-5.3998	-0.7577
C	-3.6081	-4.3918	-0.8713
O	-3.2999	-3.1776	-0.6502
C	-5.0277	-4.6422	-1.3291
H	-5.2480	-5.7114	-1.3525
H	-5.1591	-4.2238	-2.3337
H	-5.7271	-4.1293	-0.6616
O	-1.3783	-5.3572	1.7851
C	-1.8415	-4.3606	2.4271
O	-1.9938	-3.1769	1.9865
C	-2.2935	-4.6092	3.8486
H	-2.2251	-3.6916	4.4377
H	-1.6996	-5.4040	4.3070
H	-3.3425	-4.9305	3.8314
O	0.5388	-2.4796	0.7778
C	1.3527	-3.4583	0.7350
O	1.0906	-4.6433	0.3606

C	2.7789	-3.1517	1.1345
H	3.2870	-4.0524	1.4866
H	3.3137	-2.7729	0.2541
H	2.7995	-2.3763	1.9047
O	-0.7590	-2.4485	-1.9088
C	-0.4137	-3.4669	-2.5956
O	-0.3742	-4.6694	-2.1923
C	0.0016	-3.1885	-4.0224
H	-0.7664	-2.5878	-4.5200
H	0.9288	-2.6035	-4.0185
H	0.1635	-4.1179	-4.5720

Ru-TS-I3-O

Energy (POTENTIAL) = -2542.5600348 Eh

Atom	X	Y	Z
N	-1.7630	-2.8736	2.3271
C	-3.0271	-2.3565	2.3243
C	-3.8167	-2.2422	3.4919
C	-3.2592	-2.7149	4.7012
C	-1.9834	-3.2478	4.6910
C	-1.2620	-3.3056	3.4861
C	-3.5472	-1.9282	1.0630
H	-1.5175	-3.6118	5.6000
H	-0.2550	-3.7017	3.4577
C	-4.8375	-1.3529	0.9839
C	-5.2914	-0.9529	-0.2924
C	-4.4719	-1.1439	-1.3907
C	-3.2020	-1.7219	-1.2192
N	-2.7456	-2.1056	-0.0259
H	-4.7883	-0.8513	-2.3862
H	-2.5345	-1.8701	-2.0603
Ru	-0.7906	-2.8310	0.4224
C	-0.3174	-4.9336	0.0797
C	-0.6027	-4.3081	-1.1779
C	0.3756	-3.2874	-1.4155
C	1.2700	-3.2956	-0.2863
C	0.8688	-4.3101	0.6208
H	-1.4331	-4.5552	-1.8289
H	0.4505	-2.6547	-2.2900
H	2.0881	-2.6036	-0.1306
H	1.3535	-4.5607	1.5539
H	-0.8627	-5.7599	0.5186
O	-0.5185	-0.6258	0.8033
C	-0.6101	0.2190	1.7061
C	0.0259	0.2420	3.0187
C	0.8534	-0.8836	3.4998
O	0.9419	-1.2281	4.6701
H	-6.2754	-0.5053	-0.3985
H	-3.8321	-2.6479	5.6215
C	-5.1218	-1.6500	3.3888
C	-5.6083	-1.2178	2.1890
H	-6.5959	-0.7707	2.1215
H	-5.7149	-1.5540	4.2937
O	-1.3190	1.3480	1.4887
C	-1.9749	1.4814	0.2164
H	-2.3628	2.5012	0.1999
H	-1.2722	1.3315	-0.6075
H	-2.7990	0.7690	0.1284
O	1.6205	-1.4403	2.5306
C	2.5563	-2.4282	2.9883
H	3.0674	-2.7894	2.0954
H	3.2795	-1.9827	3.6791
H	2.0501	-3.2539	3.4962
O	0.9604	7.1177	4.6710
O	2.2207	5.9717	6.7338
N	1.0266	2.5192	3.4846
N	-2.0590	3.8933	6.9227
C	0.2629	4.4425	5.3924
C	1.1645	4.7885	4.3234
C	1.6115	6.1232	4.0264
C	2.5917	6.3442	3.0724
H	2.9364	7.3482	2.8595
C	3.1097	5.2637	2.3231
H	3.8806	5.4631	1.5837
C	2.6160	3.9886	2.4694
H	2.9552	3.1656	1.8486
C	1.6001	3.7428	3.4277
C	-0.0287	2.2940	4.2860
C	-0.4528	3.2336	5.2545
C	-1.6015	2.9146	6.0452
C	-3.3887	3.7315	7.5366
H	-4.0294	3.2440	6.7976
H	-3.8154	4.7243	7.6946
C	-3.3733	2.9297	8.8451
H	-2.9230	1.9470	8.6626
H	-2.7299	3.4358	9.5740
C	-4.7861	2.7676	9.4101
H	-4.7715	2.1987	10.3464
H	-5.4376	2.2364	8.7047
C	-1.2217	4.9356	7.3127
C	-1.5651	5.7612	8.4073
H	-2.5071	5.6423	8.9234
C	-0.6614	6.7008	8.8728
H	-0.9356	7.3191	9.7230

C	0.6218	6.8245	8.3265
H	1.3384	7.4990	8.7777
C	0.9685	6.0357	7.2351
C	3.2144	6.8728	7.2197
H	2.8944	7.9157	7.1163
H	4.0946	6.6982	6.5984
H	3.4597	6.6649	8.2685
C	0.0074	5.1726	6.6188
C	-2.2342	1.6832	5.8446
H	-3.0985	1.4016	6.4311
C	-1.7533	0.7610	4.8877
H	-2.2684	-0.1880	4.7835
C	-0.6577	1.0414	4.0965
C	1.4238	8.4589	4.5269
H	0.8239	9.0523	5.2195
H	2.4840	8.5472	4.7910
H	1.2701	8.8258	3.5043
H	-5.2447	3.7426	9.6179
H	0.9553	1.3411	2.8718

Ru-TS-I0

Energy (POTENTIAL) = -2542.5375621 Eh

Atom	X	Y	Z
N	0.2870	-2.5801	1.1383
C	0.1167	-2.6189	2.4928
C	1.1850	-2.5141	3.4125
C	2.4884	-2.3976	2.8824
C	2.6541	-2.3973	1.5087
C	1.5316	-2.4837	0.6675
C	-1.2219	-2.7533	2.9636
H	3.6399	-2.3212	1.0621
H	1.6424	-2.4695	-0.4087
C	-1.4973	-2.7610	4.3504
C	-2.8474	-2.8857	4.7422
C	-3.8259	-2.9883	3.7688
C	-3.4672	-2.9630	2.4112
N	-2.1966	-2.8571	2.0138
H	-4.8740	-3.0776	4.0335
H	-4.2130	-3.0067	1.6275
Ru	-1.4963	-2.7727	0.0220
C	-0.9925	-4.8100	-1.0210
C	-2.3870	-4.7885	-0.7887
C	-2.9594	-3.6803	-1.4971
C	-1.8913	-3.0242	-2.1924
C	-0.6744	-3.6899	-1.8746
H	-2.9311	-5.4732	-0.1485
H	-4.0102	-3.4291	-1.5556
H	-1.9962	-2.1496	-2.8185
H	0.3062	-3.4433	-2.2614
H	-0.2849	-5.5190	-0.6099
O	-2.7224	0.6292	1.8277
C	-1.8318	0.0346	1.2458
C	-1.9968	-0.8085	0.0160
C	-3.3267	-0.6412	-0.6815
O	-3.5199	-0.4455	-1.8707
H	-3.1031	-2.8955	5.7977
H	3.3395	-2.3151	3.5518
C	0.8858	-2.5233	4.8185
C	-0.3982	-2.6374	5.2680
H	-0.6142	-2.6412	6.3324
H	1.7104	-2.4365	5.5203
O	-0.5552	0.0450	1.6911
C	-0.3244	0.7950	2.8945
H	0.7484	0.7304	3.0794
H	-0.6231	1.8393	2.7681
H	-0.8775	0.3591	3.7312
O	-4.3461	-0.8479	0.1806
C	-5.6669	-0.7646	-0.3849
H	-6.3489	-0.9379	0.4489
H	-5.8388	0.2255	-0.8169
H	-5.8112	-1.5264	-1.1572
O	-0.9941	5.3824	2.7532
O	0.1844	6.5381	0.6234
N	-2.1870	2.4719	-0.8474
N	2.3235	2.3023	0.8735
C	-0.0878	3.7180	0.6135
C	-1.4379	4.1572	0.7802
C	-1.8875	5.0901	1.7772
C	-3.1863	5.5668	1.7542
H	-3.5278	6.2778	2.4963
C	-4.1035	5.0813	0.7908
H	-5.1168	5.4739	0.7923
C	-3.7523	4.0847	-0.0871
H	-4.4674	3.6420	-0.7729
C	-2.4318	3.5550	-0.0697
C	-0.9995	1.8786	-0.7500
C	0.0986	2.4805	-0.0380
C	1.3460	1.7862	0.0593
C	3.4568	1.4421	1.2772
H	3.0732	0.4231	1.3597
H	3.7496	1.7352	2.2877
C	4.6571	1.4907	0.3233
H	4.3277	1.2430	-0.6922
H	5.0518	2.5115	0.2789

C	5.7513	0.5199	0.7737
H	6.6106	0.5586	0.0955
H	5.3828	-0.5138	0.7894
C	2.2967	3.6499	1.2563
C	3.4327	4.2578	1.8300
H	4.3240	3.6894	2.0538
C	3.4297	5.6231	2.0640
H	4.3102	6.0873	2.4990
C	2.3478	6.4277	1.6904
H	2.4115	7.5025	1.8046
C	1.2157	5.8350	1.1380
C	0.1459	7.9535	0.8016
H	0.2131	8.2264	1.8609
H	-0.8203	8.2705	0.4050
H	0.9506	8.4467	0.2424
C	1.1193	4.4139	1.0291
C	1.5152	0.5555	-0.6210
H	2.4647	0.0405	-0.5995
C	0.4654	0.0017	-1.3216
H	0.6191	-0.9373	-1.8434
C	-0.8232	0.5724	-1.3420
C	-1.3181	6.3877	3.7107
H	-0.4164	6.5225	4.3115
H	-1.5783	7.3347	3.2239
H	-2.1440	6.0707	4.3599
H	6.1080	0.7631	1.7823
H	-1.5379	0.2740	-2.0973

r-Rh-I1

Energy (POTENTIAL) = -2816.835632 Eh

Atom	X	Y	Z
O	0.5299	-1.6269	2.9822
C	-0.1817	-0.7128	2.6275
C	-1.0406	-0.6345	1.3751
C	-2.4381	-0.2688	1.6900
O	-3.2297	0.3326	0.9770
O	-0.2444	0.4593	3.3192
C	0.6065	0.5608	4.4723
H	0.4622	1.5728	4.8549
H	0.3208	-0.1757	5.2296
H	1.6542	0.4099	4.1962
O	-2.8089	-0.7600	2.9034
C	-4.2009	-0.6411	3.2173
H	-4.3231	-1.1094	4.1962
H	-4.5068	0.4095	3.2643
H	-4.8047	-1.1626	2.4702
O	-0.1621	6.6945	1.5555
O	-1.5833	5.4380	3.4479
N	2.4538	2.7535	2.2283
N	-1.7106	2.2728	-0.0776
C	-0.1152	3.8184	1.6349
C	0.9166	4.6755	2.1554
C	0.8492	6.1059	2.2330
C	1.8324	6.8151	2.9116
H	1.7707	7.8928	2.9993
C	2.9465	6.1429	3.4515
H	3.7002	6.7189	3.9813
C	3.1233	4.7891	3.2532
H	4.0195	4.2723	3.5800
C	2.1419	4.0483	2.5551
C	1.6013	2.0526	1.5150
C	0.2644	2.5244	1.2265
C	-0.6051	1.7119	0.4508
C	-2.3762	1.7022	-1.2819
H	-3.4479	1.6772	-1.1040
H	-2.0814	0.6650	-1.3936
C	-2.0101	2.5143	-2.5278
H	-2.2428	3.5731	-2.3643
H	-0.9270	2.4468	-2.6909
C	-2.7672	1.9985	-3.7541
H	-2.4989	2.5744	-4.6467
H	-3.8519	2.0826	-3.6125
C	-2.2556	3.4347	0.4956
C	-3.5486	3.8754	0.1422
H	-4.1054	3.4101	-0.6572
C	-4.1408	4.8884	0.8739
H	-5.1441	5.2110	0.6120
C	-3.5109	5.4562	1.9907
H	-4.0435	6.1768	2.5987
C	-2.2237	5.0559	2.3244
C	-2.1610	6.4563	4.2656
H	-2.3692	7.3634	3.6872
H	-1.4141	6.6742	5.0308
H	-3.0830	6.1029	4.7432
C	-1.5174	4.1307	1.4926
C	-0.2975	0.2455	0.2651
H	-0.6454	-0.1081	-0.7010
C	1.1731	-0.0490	0.3627
H	1.4750	-1.0280	0.0112
C	2.0354	0.7738	0.9755
C	-0.3242	8.1100	1.6337
H	-1.2347	8.3305	1.0733
H	-0.4430	8.4444	2.6707
H	0.5238	8.6324	1.1741

H	-2.5361	0.9454	-3.9508
H	3.0825	0.5181	1.1069
Rh	-1.3994	-2.6882	0.4461
Rh	-1.7844	-4.8870	-0.6198
H	-5.9950	-2.2995	0.2394
C	-5.6976	-3.3370	0.4147
C	-4.2114	-3.5010	0.1869
H	-6.2666	-4.0083	-0.2324
H	-5.9225	-3.5816	1.4601
O	-3.4729	-2.5523	0.6034
O	-3.8208	-4.5726	-0.3736
O	0.6305	-3.0499	0.1592
C	1.0191	-4.1346	-0.3854
O	0.2779	-5.0798	-0.7976
C	2.5139	-4.2868	-0.5564
H	2.7670	-5.2800	-0.9327
H	3.0129	-4.1127	0.4026
H	2.8755	-3.5285	-1.2607
O	-1.8459	-3.8810	-2.4484
C	-1.6896	-2.6240	-2.4787
O	-1.4828	-1.8647	-1.4717
C	-1.7850	-1.9375	-3.8219
H	-2.7798	-1.4858	-3.9164
H	-1.6458	-2.6536	-4.6346
H	-1.0427	-1.1376	-3.8921
O	-1.3943	-3.6987	2.2533
C	-1.5333	-4.9624	2.2761
O	-1.7033	-5.7299	1.2741
C	-1.4549	-5.6137	3.6390
H	-0.4038	-5.6537	3.9497
H	-1.9976	-5.0143	4.3754
H	-1.8546	-6.6298	3.6094
C	-5.2080	-0.8205	1.9332
H	-5.5608	-1.3556	2.8147
H	-5.7108	0.1467	1.8467
H	-5.3804	-1.4170	1.0349
O	1.9039	6.5656	0.2749
O	3.5241	4.7050	1.3622
N	1.4564	2.7893	-2.6497
N	-0.8283	2.7949	1.5796
C	1.1986	3.8207	-0.0122
C	1.8672	4.5851	-1.0166
C	2.3261	5.9368	-0.8475
C	3.0809	6.5493	-1.8333
H	3.4360	7.5646	-1.7067
C	3.3518	5.8665	-3.0422
H	3.9514	6.3649	-3.7994
C	2.8274	4.6202	-3.2917
H	2.9759	4.1142	-4.2402
C	2.0300	3.9721	-2.3087
C	0.6184	2.2155	-1.7883
C	0.4211	2.7223	-0.4506
C	-0.5648	2.1382	0.4018
C	-2.1792	2.7895	2.1856
H	-2.1648	2.2665	3.1466
H	-2.8277	2.2353	1.5185
C	-2.7702	4.2003	2.3099
H	-2.1545	4.8294	2.9596
H	-2.7649	4.6647	1.3158
C	-4.1991	4.1276	2.8560
H	-4.6372	5.1289	2.9335
H	-4.2179	3.6750	3.8554

r-Rh-TS-I1

Energy (POTENTIAL) = -2816.8138509 Eh

Atom	X	Y	Z
O	0.4024	0.1599	2.2791
C	-0.7890	-0.1179	2.3036
C	-1.6748	-0.0451	1.1312
C	-3.1097	-0.0737	1.1746
O	-3.7196	0.5201	0.2083
O	-1.4163	-0.4923	3.4438
C	-0.5543	-0.6798	4.5742
H	-1.1867	-1.0991	5.3593
H	0.2557	-1.3696	4.3297
H	-0.1310	0.2755	4.9035
O	-3.7924	-0.6388	2.1441
C	0.1882	3.5281	2.2027
C	0.1510	3.7502	3.5936
H	-0.6839	3.4034	4.1882
C	1.2311	4.3596	4.2089
H	1.2130	4.5137	5.2841
C	2.3760	4.7234	3.4874
H	3.2333	5.1277	4.0109
C	2.4173	4.5133	2.1124
C	4.6681	5.3184	1.9529
H	4.4195	6.2943	2.3861
H	5.3806	5.4530	1.1370
H	5.1124	4.6761	2.7234
C	1.2737	4.0025	1.4250
C	-1.2602	0.9243	0.0100
H	-2.6325	1.0538	-0.2308
C	-0.9170	0.4051	-1.3082
H	-1.4159	-0.5070	-1.6055

C	-0.0945	1.0387	-2.1829
C	2.4386	7.8478	0.5940
H	2.0574	8.0845	1.5893
H	3.5346	7.8301	0.6163
H	2.0983	8.6104	-0.1177
H	-4.8454	3.5267	2.2039
H	0.0750	0.6565	-3.1851
Rh	-1.4400	-2.5025	0.5322
Rh	-1.0048	-4.8093	-0.1449
H	-4.7985	-4.5257	3.0112
C	-4.7891	-4.8542	1.9666
C	-3.4864	-4.4402	1.3234
H	-5.6189	-4.3546	1.4547
H	-4.9265	-5.9360	1.9170
O	-3.2099	-3.1991	1.3704
O	-2.7743	-5.3492	0.7893
O	0.3552	-2.0030	-0.3537
C	1.0388	-2.8954	-0.9494
O	0.7382	-4.1287	-1.0451
C	2.3372	-2.4405	-1.5697
H	3.1096	-2.4208	-0.7910
H	2.2297	-1.4268	-1.9646
H	2.6525	-3.1280	-2.3578
O	-2.0434	-4.4188	-1.9016
C	-2.5327	-3.2607	-2.0812
O	-2.4664	-2.2840	-1.2618
C	-3.2375	-2.9865	-3.3870
H	-2.5998	-2.3389	-4.0009
H	-4.1735	-2.4514	-3.2013
H	-3.4330	-3.9139	-3.9287
O	-0.4271	-2.9415	2.2872
C	0.0738	-4.0986	2.4581
O	0.0080	-5.0727	1.6421
C	0.8278	-4.3159	3.7483
H	1.0669	-5.3719	3.8882
H	1.7573	-3.7355	3.7185
H	0.2328	-3.9499	4.5905

CF3CH2OH

Energy (POTENTIAL) = -452.807581992 Eh

Atom	X	Y	Z
O	2.3104	4.6566	10.0788
C	1.9490	5.5310	11.1269
H	2.7935	6.1168	11.5091
H	1.1822	6.2123	10.7506
C	1.3526	4.7742	12.3049
F	1.0162	5.6270	13.3071
F	2.2307	3.8718	12.8226
F	0.2350	4.0821	11.9750
H	3.1221	4.1841	10.3221

Ru-I3

Energy (POTENTIAL) = -2542.5975136 Eh

Atom	X	Y	Z
N	-0.2165	-3.2023	2.1310
C	-1.0560	-3.6302	3.1216
C	-0.6243	-3.8995	4.4419
C	0.7438	-3.7101	4.7297
C	1.5862	-3.2748	3.7217
C	1.0716	-3.0281	2.4383
C	-2.4313	-3.7844	2.7719
H	2.6437	-3.1150	3.9040
H	1.7144	-2.6755	1.6407
C	-3.3692	-4.2173	3.7375
C	-4.7115	-4.3500	3.3248
C	-5.0434	-4.0455	2.0160
C	-4.0500	-3.6070	1.1260
N	-2.7696	-3.4830	1.4844
H	-6.0654	-4.1317	1.6621
H	-4.2908	-3.3425	0.1048
Ru	-1.1659	-2.8902	0.2551
C	-0.2149	-4.4825	-1.0074
C	-1.5786	-4.3343	-1.3955
C	-1.7753	-3.0038	-1.8930
C	-0.5067	-2.3330	-1.8098
C	0.4571	-3.2324	-1.2778
H	-2.3459	-5.0937	-1.2990
H	-2.6889	-2.5890	-2.2951
H	-0.3425	-1.2948	-2.0642
H	1.5092	-3.0299	-1.1227
H	0.2391	-5.3782	-0.6017
O	-3.1064	-0.8059	2.7171
C	-1.9820	-0.6615	2.2611
C	-1.5463	-0.6312	0.8365
C	-2.5839	-0.3854	-0.2040
O	-2.3477	0.1529	-1.2832
H	-5.4643	-4.6835	4.0332
H	1.1168	-3.9020	5.7316
C	-1.5941	-4.3346	5.4089
C	-2.9080	-4.4883	5.0709
H	-3.6333	-4.8194	5.8087
H	-1.2553	-4.5399	6.4204
O	-0.9115	-0.4856	3.0968
C	-1.2267	-0.4056	4.4928

H	-0.2667	-0.3255	5.0059
H	-1.8373	0.4799	4.7009
H	-1.7626	-1.2948	4.8339
O	-3.8415	-0.7827	0.1191
C	-4.8257	-0.6104	-0.9075
H	-5.7693	-0.9346	-0.4641
H	-4.8975	0.4365	-1.2179
H	-4.5959	-1.2238	-1.7854
O	0.1630	6.3210	2.6251
O	-0.3577	6.5878	-0.0066
N	-1.7405	2.1938	1.2945
N	2.8502	3.0761	0.1747
C	0.3099	4.0128	0.9354
C	-0.8385	4.3807	1.7285
C	-0.9590	5.5831	2.5040
C	-2.1557	5.8944	3.1365
H	-2.2551	6.8072	3.7100
C	-3.2392	4.9980	3.0740
H	-4.1669	5.2605	3.5744
C	-3.1262	3.7741	2.4466
H	-3.9342	3.0486	2.4596
C	-1.9080	3.4468	1.8189
C	-0.5331	1.6879	0.8833
C	0.5268	2.6314	0.7025
C	1.8159	2.1585	0.2950
C	4.2349	2.5767	0.0503
H	4.3096	1.6718	0.6570
H	4.8989	3.3061	0.5179
C	4.6615	2.2964	-1.3966
H	3.9628	1.5844	-1.8508
H	4.5894	3.2202	-1.9819
C	6.0879	1.7445	-1.4505
H	6.3884	1.5396	-2.4838
H	6.1719	0.8079	-0.8847
C	2.5813	4.4298	0.0618
C	3.5865	5.3396	-0.3402
H	4.5985	5.0107	-0.5270
C	3.2625	6.6657	-0.5567
H	4.0404	7.3509	-0.8815
C	1.9473	7.1382	-0.4381
H	1.7165	8.1606	-0.7085
C	0.9514	6.2671	-0.0215
C	-0.7495	7.9407	-0.2452
H	-0.2433	8.6299	0.4397
H	-1.8250	7.9677	-0.0618
H	-0.5460	8.2335	-1.2820
C	1.2678	4.9171	0.3536
C	1.9900	0.7858	0.1024
H	2.9426	0.3763	-0.2037
C	0.9134	-0.0890	0.2676
H	1.0784	-1.1439	0.0975
C	-0.3669	0.3077	0.6370
C	0.0913	7.5969	3.2631
H	1.0783	8.0441	3.1342
H	-0.6653	8.2342	2.7919
H	-0.1266	7.4937	4.3329
H	6.8067	2.4572	-1.0268
H	-2.5359	1.5680	1.3486

Ru-TS-I1

Energy (POTENTIAL) = -2542.5439884 Eh

Atom	X	Y	Z
N	-0.2398	-3.1315	2.1064
C	-1.0579	-3.5273	3.1268
C	-0.5999	-3.7417	4.4478
C	0.7715	-3.5278	4.7027
C	1.5913	-3.1228	3.6642
C	1.0512	-2.9312	2.3817
C	-2.4382	-3.7051	2.8087
H	2.6498	-2.9444	3.8213
H	1.6748	-2.6010	1.5598
C	-3.3533	-4.1119	3.8066
C	-4.7023	-4.2693	3.4253
C	-5.0627	-4.0142	2.1136
C	-4.0902	-3.5996	1.1898
N	-2.8048	-3.4502	1.5196
H	-6.0905	-4.1211	1.7831
H	-4.3529	-3.3778	0.1634
Ru	-1.2309	-2.9014	0.2343
C	-0.4111	-4.6190	-0.9471
C	-1.7558	-4.3720	-1.3561
C	-1.8354	-3.0585	-1.9187
C	-0.5097	-2.4981	-1.8651
C	0.3651	-3.4463	-1.2764
H	-2.5872	-5.0553	-1.2276
H	-2.7087	-2.5903	-2.3515
H	-0.2349	-1.5085	-2.2048
H	1.4281	-3.3204	-1.1124
H	-0.0348	-5.5319	-0.5028
O	-2.9652	-0.7661	2.7657
C	-1.8808	-0.5763	2.2410
C	-1.5500	-0.6469	0.7975
C	-2.6364	-0.4596	-0.1560
O	-2.3303	0.0841	-1.2727

H	-5.4384	-4.5831	4.1596
H	1.1641	-3.6770	5.7043
C	-1.5471	-4.1511	5.4480
C	-2.8648	-4.3307	5.1398
H	-3.5734	-4.6414	5.9022
H	-1.1878	-4.3150	6.4600
O	-0.7683	-0.2976	2.9754
C	-0.9862	-0.1458	4.3847
H	0.0038	-0.0084	4.8229
H	-1.6098	0.7323	4.5829
H	-1.4679	-1.0303	4.8090
O	-3.8820	-0.7859	0.1120
C	-4.8558	-0.5911	-0.9375
H	-5.8095	-0.8828	-0.4974
H	-4.8815	0.4569	-1.2452
H	-4.6232	-1.2257	-1.7974
O	0.1117	6.2700	2.6367
O	-0.2633	6.6153	0.0014
N	-1.7917	2.1274	1.1637
N	2.8471	3.0243	0.1913
C	0.3135	4.0023	0.9018
C	-0.8523	4.3424	1.6571
C	-1.0073	5.5299	2.4546
C	-2.2218	5.8152	3.0549
H	-2.3449	6.7110	3.6509
C	-3.3026	4.9100	2.9385
H	-4.2455	5.1600	3.4176
C	-3.1580	3.7077	2.2878
H	-3.9526	2.9694	2.2534
C	-1.9167	3.3771	1.6805
C	-0.6054	1.7244	0.7361
C	0.5009	2.6358	0.5959
C	1.7960	2.1450	0.2327
C	4.2292	2.5031	0.1006
H	4.2723	1.5826	0.6862
H	4.8835	3.2117	0.6112
C	4.7017	2.2578	-1.3378
H	4.0181	1.5576	-1.8313
H	4.6480	3.1951	-1.9029
C	6.1294	1.7070	-1.3592
H	6.4614	1.5236	-2.3870
H	6.1974	0.7594	-0.8100
C	2.6210	4.4069	0.1190
C	3.6584	5.2897	-0.2458
H	4.6659	4.9388	-0.4165
C	3.3747	6.6317	-0.4417
H	4.1775	7.3042	-0.7296
C	2.0700	7.1275	-0.3460
H	1.8667	8.1620	-0.5926
C	1.0407	6.2669	0.0231
C	-0.6146	7.9907	-0.1456
H	-0.1040	8.6144	0.5969
H	-1.6925	8.0360	0.0195
H	-0.3832	8.3551	-1.1541
C	1.3200	4.9131	0.3852
C	1.9649	0.7562	0.0050
H	2.9326	0.3484	-0.2504
C	0.8861	-0.0986	0.0803
H	1.0492	-1.1548	-0.0912
C	-0.4479	0.3195	0.3296
C	0.0338	7.4714	3.4006
H	1.0289	7.9170	3.3468
H	-0.7006	8.1692	2.9810
H	-0.2178	7.2608	4.4477
H	6.8340	2.4115	-0.8997
H	-1.1235	0.3663	-0.8752

Ru-I3-O

Energy (POTENTIAL) = -2542.5943976 Eh

Atom	X	Y	Z
N	-0.8726	-2.5862	2.5318
C	-2.0206	-2.2498	3.1871
C	-2.0610	-1.9393	4.5664
C	-0.8458	-2.0035	5.2820
C	0.3086	-2.3657	4.6128
C	0.2580	-2.6478	3.2375
C	-3.2186	-2.2156	2.4076
H	1.2623	-2.4223	5.1260
H	1.1533	-2.9116	2.6893
C	-4.4476	-1.8542	3.0095
C	-5.5958	-1.8520	2.1880
C	-5.4703	-2.1986	0.8541
C	-4.2068	-2.5341	0.3369
N	-3.1037	-2.5456	1.0884
H	-6.3303	-2.2098	0.1929
H	-4.0795	-2.7902	-0.7088
Ru	-1.0990	-2.8767	0.4268
C	-0.3703	-4.9246	0.1630
C	-1.4020	-4.6692	-0.7976
C	-0.9722	-3.6123	-1.6667
C	0.3424	-3.2190	-1.2263
C	0.7250	-4.0241	-0.1221
H	-2.3552	-5.1824	-0.8472
H	-1.5130	-3.2110	-2.5136
H	0.9227	-2.4024	-1.6375
H	1.6584	-3.9579	0.4209
H	-0.3908	-5.6854	0.9335
O	-1.1773	-0.6631	0.1414
C	-1.1546	0.3271	0.9086
C	-0.2411	0.6280	1.9701
C	1.0718	0.0640	1.9920
O	1.9307	0.2803	2.8766
H	-6.5599	-1.5823	2.6094
H	-0.8305	-1.7650	6.3415
C	-3.3171	-1.5611	5.1524
C	-4.4596	-1.5161	4.4062
H	-5.4050	-1.2302	4.8584
H	-3.3358	-1.3073	6.2083
O	-2.0939	1.2969	0.7107
C	-3.1049	1.0300	-0.2686
H	-3.7486	1.9123	-0.2684
H	-2.6676	0.8891	-1.2618
H	-3.6909	0.1434	-0.0080
O	1.4040	-0.7152	0.9248
C	2.7521	-1.1905	0.8986
H	2.8378	-1.7717	-0.0212
H	3.4665	-0.3605	0.8824
H	2.9759	-1.8234	1.7636
O	1.2117	7.2020	5.1102
O	1.8606	5.5869	7.1879
N	1.3108	2.8286	3.3030
N	-2.4946	4.1273	5.9557
C	0.1792	4.5241	5.1782
C	1.3593	4.9370	4.4633
C	1.9549	6.2427	4.5193
C	3.1901	6.4751	3.9318
H	3.6483	7.4549	3.9780
C	3.8423	5.4399	3.2305
H	4.8140	5.6396	2.7874
C	3.2472	4.2112	3.0405
H	3.7092	3.4380	2.4347
C	1.9711	3.9809	3.6008
C	0.0079	2.5810	3.6513
C	-0.5709	3.4468	4.6410
C	-1.9219	3.2351	5.0602
C	-3.9561	4.0903	6.1603
H	-4.4119	3.8281	5.2030
H	-4.2914	5.1046	6.3842
C	-4.3983	3.1117	7.2562
H	-4.0242	2.1086	7.0204
H	-3.9382	3.3995	8.2084
C	-5.9224	3.0876	7.3911
H	-6.2347	2.3859	8.1724
H	-6.3991	2.7791	6.4522
C	-1.7000	4.9592	6.7255
C	-2.2403	5.6685	7.8234
H	-3.2931	5.6147	8.0614
C	-1.4020	6.3963	8.6463
H	-1.8257	6.9252	9.4954
C	-0.0123	6.4143	8.4579
H	0.6212	6.9166	9.1777
C	0.5330	5.7411	7.3734
C	2.7688	6.3155	8.0147
H	2.5562	7.3900	7.9893
H	3.7591	6.1291	7.5955
H	2.7357	5.9568	9.0505
C	-0.3124	5.0970	6.4090
C	-2.6295	2.1689	4.4971
H	-3.6437	1.9437	4.7967
C	-2.0235	1.3500	3.5511
H	-2.6151	0.5377	3.1479

C	-0.7155	1.5144	3.0664
C	1.7664	8.5065	5.2809
H	1.0314	9.0610	5.8671
H	2.7163	8.4689	5.8257
H	1.9149	9.0024	4.3138
H	-6.3136	4.0782	7.6549
H	1.8311	2.0174	2.9380

Ru-I2

Energy (POTENTIAL) = -2542.5710344 Eh

Atom	X	Y	Z
N	-0.2760	-3.2339	2.0817
C	-1.1128	-3.6857	3.0615
C	-0.6727	-4.0017	4.3682
C	0.7012	-3.8400	4.6471
C	1.5410	-3.3857	3.6464
C	1.0169	-3.0888	2.3773
C	-2.4929	-3.8138	2.7188
H	2.6023	-3.2470	3.8232
H	1.6557	-2.7191	1.5851
C	-3.4251	-4.2688	3.6790
C	-4.7726	-4.3762	3.2752
C	-5.1150	-4.0330	1.9795
C	-4.1254	-3.5762	1.0944
N	-2.8429	-3.4650	1.4474
H	-6.1402	-4.1032	1.6319
H	-4.3741	-3.2938	0.0795
Ru	-1.2437	-2.8456	0.2168
C	-0.4380	-4.5217	-1.0028
C	-1.7541	-4.2144	-1.4655
C	-1.7671	-2.8764	-1.9654
C	-0.4261	-2.3615	-1.8267
C	0.3902	-3.3627	-1.2457
H	-2.6119	-4.8736	-1.4031
H	-2.6072	-2.3598	-2.4085
H	-0.0929	-1.3733	-2.1149
H	1.4467	-3.2770	-1.0252
H	-0.1122	-5.4654	-0.5841
O	-2.9171	-0.8830	2.9143
C	-1.8221	-0.6612	2.4298
C	-1.4571	-0.6045	0.9803
C	-2.5413	-0.4474	0.0667
O	-2.3570	0.0932	-1.1346
H	-5.5214	-4.7233	3.9812
H	1.0806	-4.0684	5.6388
C	-1.6385	-4.4536	5.3314
C	-2.9563	-4.5835	5.0002
H	-3.6795	-4.9282	5.7337
H	-1.2928	-4.6921	6.3331
O	-0.7263	-0.4486	3.2007
C	-0.9679	-0.4074	4.6155
H	0.0163	-0.3196	5.0780
H	-1.5831	0.4611	4.8718
H	-1.4692	-1.3162	4.9575
O	-3.7911	-0.7006	0.3639
C	-4.7989	-0.5647	-0.6663
H	-5.7296	-0.8609	-0.1836
H	-4.8595	0.4715	-1.0060
H	-4.5771	-1.2254	-1.5085
O	0.0826	6.3823	2.5666
O	-0.3529	6.5850	-0.0864
N	-1.7207	2.1169	1.3582
N	2.8864	3.1180	0.2121
C	0.3282	4.0374	0.9408
C	-0.8350	4.3805	1.6956
C	-1.0220	5.6093	2.4234
C	-2.2427	5.9053	3.0008
H	-2.3873	6.8305	3.5448
C	-3.3067	4.9698	2.9351
H	-4.2579	5.2306	3.3921
C	-3.1318	3.7344	2.3663
H	-3.9106	2.9781	2.3744
C	-1.8740	3.3851	1.7888
C	-0.5101	1.7220	0.9363
C	0.5580	2.6614	0.7145
C	1.8504	2.1931	0.3038
C	4.2708	2.6286	0.1019
H	4.3457	1.7219	0.7079
H	4.9276	3.3614	0.5764
C	4.7232	2.3498	-1.3384
H	4.0359	1.6339	-1.8039
H	4.6536	3.2727	-1.9255
C	6.1538	1.8073	-1.3737
H	6.4721	1.6050	-2.4024
H	6.2366	0.8711	-0.8067
C	2.6090	4.4787	0.0808
C	3.6082	5.3855	-0.3370
H	4.6259	5.0611	-0.5029
C	3.2749	6.7061	-0.5909
H	4.0499	7.3927	-0.9204
C	1.9545	7.1591	-0.5009
H	1.7095	8.1717	-0.7956
C	0.9645	6.2789	-0.0713
C	-0.7546	7.9282	-0.3447

H	-0.2649	8.6324	0.3376
H	-1.8328	7.9482	-0.1743
H	-0.5434	8.2154	-1.3823
C	1.2951	4.9555	0.3517
C	2.0330	0.8265	0.0937
H	2.9877	0.4286	-0.2221
C	0.9655	-0.0725	0.2777
H	1.1486	-1.1250	0.1029
C	-0.2951	0.3215	0.6799
C	-0.0440	7.6571	3.1890
H	0.9338	8.1328	3.0903
H	-0.8010	8.2743	2.6906
H	-0.2963	7.5596	4.2527
H	6.8615	2.5244	-0.9388
H	-1.4139	0.3582	-1.2056

r-Ru-TS-I0

Energy (POTENTIAL) = -2542.5381775 Eh

Atom	X	Y	Z
N	-0.4328	-1.3391	1.9950
C	-1.2095	-1.4106	3.1168
C	-0.9612	-0.6497	4.2814
C	0.1615	0.2063	4.2716
C	0.9644	0.2427	3.1466
C	0.6388	-0.5447	2.0291
C	-2.3252	-2.2967	3.0684
H	1.8357	0.8856	3.0934
H	1.2478	-0.5199	1.1368
C	-3.1982	-2.4150	4.1744
C	-4.2919	-3.2980	4.0456
C	-4.4664	-3.9877	2.8584
C	-3.5576	-3.8041	1.8036
N	-2.5066	-2.9862	1.9053
H	-5.3011	-4.6666	2.7205
H	-3.6823	-4.3059	0.8526
Ru	-1.0250	-2.6526	0.4394
C	0.8710	-4.0311	0.5253
C	-0.2549	-4.8648	0.3497
C	-0.9056	-4.5084	-0.8809
C	-0.1412	-3.4537	-1.4740
C	0.9363	-3.1281	-0.5977
H	-0.6004	-5.6169	1.0492
H	-1.7715	-4.9928	-1.3118
H	-0.3629	-2.9724	-2.4163
H	1.7041	-2.3872	-0.7767
H	1.5449	-4.0394	1.3731
O	-4.5630	-0.7589	0.1229
C	-3.3549	-0.6758	0.2862
C	-2.3181	-1.3900	-0.5323
C	-2.9173	-2.1078	-1.7216
O	-2.6050	-1.9793	-2.8982
H	-4.9870	-3.4196	4.8712
H	0.3833	0.8176	5.1413
C	-1.8609	-0.7807	5.3951
C	-2.9303	-1.6275	5.3463
H	-3.6033	-1.7193	6.1937
H	-1.6645	-0.1888	6.2846
O	-2.8209	0.0643	1.2806
C	-3.7588	0.6545	2.2005
H	-3.1741	1.3334	2.8203
H	-4.5420	1.1989	1.6705
H	-4.2181	-0.1228	2.8177
O	-3.8421	-3.0132	-1.3381
C	-4.4540	-3.7709	-2.3991
H	-5.1772	-4.4236	-1.9079
H	-4.9599	-3.1066	-3.1056
H	-3.7073	-4.3652	-2.9339
O	-2.0138	6.1520	1.1742
O	-2.9049	4.2786	2.8943
N	1.1684	2.6184	0.6375
N	-3.2565	1.9446	-1.1874
C	-1.5718	3.3757	0.5684
C	-0.5954	4.2560	1.1466
C	-0.8373	5.6095	1.5643
C	0.1363	6.3132	2.2576
H	-0.0492	7.3271	2.5895
C	1.4012	5.7321	2.4929
H	2.1447	6.3019	3.0437
C	1.7196	4.4955	1.9795
H	2.7133	4.0705	2.0777
C	0.7496	3.7623	1.2477
C	0.3141	1.9578	-0.1315
C	-1.0916	2.2843	-0.1825
C	-1.9630	1.5202	-1.0193
C	-4.0949	1.3789	-2.2729
H	-5.1051	1.2441	-1.8914
H	-3.7448	0.3790	-2.5070
C	-4.0679	2.2572	-3.5258
H	-4.4195	3.2647	-3.2721
H	-3.0288	2.3552	-3.8641
C	-4.9320	1.6543	-4.6357
H	-4.9018	2.2777	-5.5361
H	-5.9795	1.5689	-4.3209

C	-3.8198	2.8912	-0.3229
C	-5.1945	3.2109	-0.3880
H	-5.8169	2.8629	-1.1989
C	-5.7686	3.9569	0.6268
H	-6.8281	4.1902	0.5757
C	-5.0351	4.3520	1.7539
H	-5.5367	4.8357	2.5825
C	-3.6726	4.0839	1.7997
C	-3.4530	4.9763	4.0126
H	-3.8273	5.9645	3.7235
H	-2.6273	5.0911	4.7171
H	-4.2589	4.4000	4.4832
C	-3.0105	3.4887	0.6822
C	-1.4700	0.3330	-1.6504
H	-1.9677	-0.0346	-2.5325
C	-0.0458	0.1047	-1.6486
H	0.3205	-0.7182	-2.2519
C	0.8106	0.8628	-0.9256
C	-2.3510	7.4706	1.6015
H	-3.3612	7.6484	1.2285
H	-2.3458	7.5516	2.6947
H	-1.6665	8.2139	1.1743
H	-4.5810	0.6514	-4.9094
H	1.8811	0.6835	-0.9168

Rh-I3

Energy (POTENTIAL) = -2816.8753468 Eh

Atom	X	Y	Z
O	-2.8823	-1.0051	3.1183
C	-1.9115	-0.6905	2.4492
C	-1.8290	-0.5197	0.9756
C	-3.0721	-0.5320	0.2013
O	-3.1957	-0.0015	-0.9171
O	-0.7075	-0.4393	3.0506
C	-0.6279	-0.8072	4.4278
H	0.4081	-0.6252	4.7232
H	-1.2999	-0.2013	5.0457
H	-0.8773	-1.8643	4.5648
O	-4.1114	-1.1816	0.7565
C	-5.2737	-1.3101	-0.0696
H	-5.9966	-1.8625	0.5347
H	-5.6828	-0.3307	-0.3391
H	-5.0387	-1.8677	-0.9794
O	-0.4865	6.7259	-0.0242
O	-0.2526	5.8959	-2.6004
N	-2.1252	2.2855	-0.0896
N	2.6345	2.9729	-0.2094
C	0.0253	3.9482	-0.5711
C	-1.2744	4.5213	-0.3323
C	-1.5592	5.9203	-0.1796
C	-2.8721	6.3669	-0.1345
H	-3.0944	7.4214	-0.0321
C	-3.9337	5.4398	-0.1840
H	-4.9544	5.8108	-0.1591
C	-3.7032	4.0810	-0.1988
H	-4.5155	3.3618	-0.1595
C	-2.3682	3.6195	-0.2071
C	-0.8811	1.7343	0.0894
C	0.2359	2.6022	-0.1800
C	1.5676	2.1059	-0.0175
C	3.9781	2.5623	0.2435
H	3.8493	1.9693	1.1514
H	4.5207	3.4599	0.5456
C	4.7665	1.7756	-0.8112
H	4.1906	0.8919	-1.1094
H	4.8826	2.3908	-1.7110
C	6.1376	1.3577	-0.2753
H	6.6973	0.7944	-1.0301
H	6.0386	0.7221	0.6136
C	2.4645	4.1569	-0.9067
C	3.5782	4.9127	-1.3423
H	4.5878	4.6095	-1.1048
C	3.3800	6.0261	-2.1365
H	4.2431	6.5909	-2.4778
C	2.1023	6.4076	-2.5715
H	1.9947	7.2273	-3.2702
C	0.9970	5.6909	-2.1339
C	-0.5121	7.0292	-3.4284
H	-0.1958	7.9588	-2.9422
H	-1.5937	7.0424	-3.5740
H	-0.0137	6.9321	-4.4005
C	1.1437	4.6209	-1.1896
C	1.7304	0.7841	0.4029
H	2.7129	0.3527	0.5386
C	0.6206	-0.0189	0.6508
H	0.8024	-1.0221	1.0054
C	-0.7124	0.3926	0.5075
C	-0.6823	8.1377	0.0524
H	0.3195	8.5696	0.0820
H	-1.2183	8.5150	-0.8261
H	-1.2280	8.4137	0.9631
H	6.7380	2.2328	0.0039
Rh	-1.2872	-2.8774	0.4131
Rh	-0.7076	-5.2043	-0.1041

O	-2.1729	-5.1720	-1.5671
C	-2.8438	-4.1050	-1.7419
O	-2.7111	-3.0175	-1.0962
C	-3.9256	-4.1337	-2.7969
H	-3.7365	-4.9278	-3.5228
H	-3.9893	-3.1658	-3.3008
H	-4.8890	-4.3275	-2.3097
O	-2.1214	-5.7917	1.2964
C	-2.7593	-4.8888	1.9308
O	-2.6275	-3.6328	1.7938
C	-3.8042	-5.3547	2.9185
H	-3.9458	-4.6104	3.7055
H	-3.5224	-6.3188	3.3494
H	-4.7561	-5.4804	2.3874
O	0.1949	-2.9587	1.8724
C	0.8638	-4.0296	2.0440
O	0.7252	-5.1151	1.3983
C	1.9415	-3.9765	3.1017
H	2.2416	-4.9821	3.4040
H	2.8147	-3.4560	2.6892
H	1.5903	-3.4078	3.9672
O	0.1132	-2.3258	-1.0267
C	0.7825	-3.2211	-1.6349
O	0.6822	-4.4788	-1.4700
C	1.7667	-2.7314	-2.6722
H	1.2341	-2.5863	-3.6204
H	2.1872	-1.7687	-2.3691
H	2.5620	-3.4638	-2.8294
H	-2.8793	1.6229	-0.2930

3g

Energy (POTENTIAL) = -2357.11674371 Eh

Atom	X	Y	Z
O	0.9590	8.4649	4.2519
O	-1.2662	6.9377	4.5839
O	3.8599	0.9977	9.4150
O	5.7187	2.1081	8.7749
O	4.5938	4.9105	10.2167
O	5.1890	5.0400	8.0436
N	2.1636	5.4176	7.7368
N	2.0310	3.8168	3.1553
C	1.2549	5.7348	5.0647
C	1.2687	6.8507	5.9578
C	1.0008	8.2157	5.5856
C	0.8874	9.2011	6.5503
H	0.6816	10.2274	6.2734
C	1.1006	8.8836	7.9178
H	0.9978	9.6728	8.6580
C	1.4927	7.6256	8.3035
H	1.7314	7.3892	9.3357
C	1.6488	6.5949	7.3260
C	2.4429	4.4814	6.8142
C	2.0055	4.6014	5.4499
C	2.3727	3.6012	4.4888
C	2.6771	3.0050	2.1090
H	3.7031	2.8106	2.4326
H	2.7567	3.6186	1.2085
C	1.9566	1.6851	1.7973
H	1.8839	1.0832	2.7098
H	0.9289	1.8961	1.4816
C	2.6905	0.8998	0.7066
H	2.1755	-0.0416	0.4865
H	3.7154	0.6566	1.0130
C	1.0164	4.7110	2.8231
C	0.4476	4.7113	1.5282
H	0.8253	4.0611	0.7522
C	-0.6475	5.5168	1.2582
H	-1.0825	5.5020	0.2627
C	-1.2488	6.2963	2.2553
H	-2.1566	6.8437	2.0373
C	-0.6827	6.3206	3.5283
C	-2.3985	7.7819	4.3653
H	-2.1788	8.5587	3.6244
H	-2.6057	8.2472	5.3304
H	-3.2721	7.2009	4.0457
C	0.5353	5.6275	3.8027
C	3.1316	2.5094	4.9091
H	3.4295	1.7309	4.2209
C	3.5356	2.3984	6.2558
H	4.1338	1.5317	6.5229
C	3.2111	3.3423	7.2078
C	3.6622	3.2977	8.6575
H	2.7891	3.3304	9.3156
C	4.3908	2.0061	9.0022
C	6.5074	0.9495	9.0688
H	6.4915	0.7234	10.1380
C	4.5108	4.5050	9.0749
C	5.9501	6.2140	8.3283
H	5.3528	6.9534	8.8674
C	0.6133	9.7742	3.7991
H	0.5625	9.7043	2.7111
H	-0.3603	10.0882	4.1925
H	1.3786	10.5070	4.0829

H	6.8536	5.9743	8.8958
H	6.1696	0.0816	8.4973
H	2.7486	1.4748	-0.2260
C	7.9247	1.2892	8.6554
C	6.3472	6.7949	6.9864
F	7.0888	7.9156	7.1744
F	5.2758	7.1385	6.2337
F	7.0923	5.9371	6.2476
F	8.4146	2.3638	9.3186
F	8.7406	0.2400	8.9203
F	8.0253	1.5596	7.3322

r-Rh-I2

Energy (POTENTIAL) = -2816.8454447 Eh

Atom	X	Y	Z
O	0.2120	0.0294	2.5210
C	-0.9924	-0.1411	2.4271
C	-1.7449	0.0542	1.1599
C	-3.1299	-0.1118	1.0355
O	-3.7904	0.2626	-0.0612
O	-1.7540	-0.4976	3.4835
C	-1.0252	-0.7668	4.6911
H	-1.7760	-1.0748	5.4211
H	-0.3012	-1.5681	4.5277
H	-0.5049	0.1309	5.0405
O	-3.9000	-0.6002	1.9793
C	-5.2546	-0.9848	1.6465
H	-5.6254	-1.4893	2.5379
H	-5.8592	-0.0998	1.4315
H	-5.2574	-1.6686	0.7970
O	1.9827	6.5886	0.3603
O	3.6764	4.6774	1.2499
N	1.2969	2.9603	-2.7077
N	-0.6512	2.7660	1.7079
C	1.2514	3.8567	0.0002
C	1.8303	4.6693	-1.0170
C	2.3103	6.0139	-0.8239
C	2.9800	6.6760	-1.8349
H	3.3431	7.6860	-1.6889
C	3.1485	6.0562	-3.1006
H	3.6847	6.5955	-3.8773
C	2.6031	4.8276	-3.3676
H	2.6715	4.3687	-4.3491
C	1.8835	4.1215	-2.3542
C	0.5309	2.3340	-1.7968
C	0.4516	2.7720	-0.4294
C	-0.4331	2.1155	0.4884
C	-2.0038	2.8602	2.2899
H	-2.0462	2.3569	3.2628
H	-2.6749	2.3236	1.6235
C	-2.5056	4.3060	2.3956
H	-1.8490	4.8919	3.0474
H	-2.4562	4.7638	1.3993
C	-3.9398	4.3464	2.9302
H	-4.3047	5.3777	2.9957
H	-4.0021	3.9056	3.9332
C	0.4021	3.4750	2.2824
C	0.4540	3.6726	3.6763
H	-0.3491	3.3143	4.3090
C	1.5720	4.2700	4.2358
H	1.6217	4.4072	5.3127
C	2.6707	4.6413	3.4495
H	3.5621	5.0326	3.9235
C	2.6186	4.4616	2.0679
C	4.8518	5.2818	1.7805
H	4.6296	6.2431	2.2590
H	5.5085	5.4465	0.9239
H	5.3517	4.6221	2.5012
C	1.4323	3.9757	1.4450
C	-1.0473	0.9026	0.1337
H	-3.1633	0.6401	-0.7113
C	-0.9223	0.4788	-1.2227
H	-1.3923	-0.4526	-1.5146
C	-0.2032	1.1747	-2.1657
C	2.5372	7.8587	0.6861
H	2.2432	8.0529	1.7198
H	3.6314	7.8503	0.6127
H	2.1351	8.6495	0.0395
H	-4.6246	3.7893	2.2781
H	-0.1451	0.8331	-3.1948
Rh	-1.5037	-2.5562	0.5569
Rh	-1.0390	-4.8328	-0.1448
H	-5.6577	-4.4975	1.4370
C	-4.8182	-4.9564	1.9713
C	-3.5244	-4.5165	1.3306
H	-4.9199	-6.0428	1.9453
H	-4.8508	-4.6027	3.0069
O	-3.2628	-3.2715	1.3869
O	-2.8003	-5.4075	0.7826
O	0.2864	-2.0187	-0.3087
C	0.9835	-2.8918	-0.9175
O	0.6950	-4.1274	-1.0343
C	2.2772	-2.4130	-1.5264
H	2.6039	-3.0856	-2.3226

H	3.0459	-2.3940	-0.7440
H	2.1575	-1.3952	-1.9072
O	-2.0778	-4.4301	-1.8987
C	-2.5614	-3.2685	-2.0742
O	-2.5003	-2.3007	-1.2430
C	-3.2497	-2.9797	-3.3844
H	-4.1831	-2.4383	-3.2043
H	-3.4468	-3.9017	-3.9344
H	-2.5995	-2.3336	-3.9866
O	-0.5119	-2.9953	2.3180
C	0.0171	-4.1425	2.4724
O	-0.0242	-5.1031	1.6375
C	0.7604	-4.3692	3.7657
H	1.5861	-3.6528	3.8353
H	0.0881	-4.1848	4.6100
H	1.1509	-5.3871	3.8197

r-Ru-I1

Energy (POTENTIAL) = -2542.562063 Eh

Atom	X	Y	Z
N	-0.3576	-1.9278	2.0913
C	-1.2223	-2.0356	3.1439
C	-0.9852	-1.4463	4.4074
C	0.2163	-0.7239	4.5724
C	1.0964	-0.6379	3.5086
C	0.7779	-1.2530	2.2862
C	-2.4170	-2.7813	2.9144
H	2.0322	-0.0959	3.5947
H	1.4496	-1.1896	1.4411
C	-3.3715	-2.9383	3.9457
C	-4.5291	-3.6894	3.6490
C	-4.6772	-4.2212	2.3804
C	-3.6858	-4.0036	1.4095
N	-2.5752	-3.3087	1.6650
H	-5.5552	-4.8003	2.1144
H	-3.7914	-4.3810	0.4012
Ru	-0.9849	-2.9396	0.3311
C	0.6186	-4.4860	0.2337
C	-0.6201	-5.0762	-0.1565
C	-1.0862	-4.4234	-1.3458
C	-0.1083	-3.4278	-1.6865
C	0.9442	-3.4630	-0.7322
H	-1.1361	-5.8661	0.3766
H	-1.9764	-4.6672	-1.9078
H	-0.1950	-2.7412	-2.5182
H	1.8350	-2.8481	-0.7343
H	1.2179	-4.7687	1.0904
O	-4.1940	-0.9139	0.9147
C	-3.0351	-0.6176	0.6732
C	-2.1963	-1.0063	-0.4866
C	-2.8567	-1.7400	-1.5945
O	-2.4787	-1.6923	-2.7689
H	-5.2873	-3.8365	4.4126
H	0.4362	-0.2495	5.5244
C	-1.9713	-1.6104	5.4398
C	-3.1138	-2.3247	5.2193
H	-3.8526	-2.4440	6.0068
H	-1.7856	-1.1501	6.4062
O	-2.3345	0.1936	1.5289
C	-3.0845	0.7416	2.6243
H	-2.4036	1.4269	3.1305
H	-3.9655	1.2801	2.2653
H	-3.4027	-0.0465	3.3108
O	-3.9334	-2.4812	-1.2501
C	-4.5753	-3.1791	-2.3268
H	-5.4037	-3.7214	-1.8669
H	-4.9572	-2.4770	-3.0748
H	-3.8916	-3.8797	-2.8150
O	-2.1833	6.4492	0.6326
O	-2.9359	4.9307	2.7164
N	1.1752	3.0363	0.8755
N	-3.1226	1.8858	-0.9187
C	-1.5790	3.6560	0.5894
C	-0.6544	4.6806	0.9838
C	-0.9744	6.0734	1.1036
C	-0.0361	6.9631	1.6113
H	-0.2778	8.0130	1.7217
C	1.2551	6.5134	1.9487
H	1.9702	7.2277	2.3471
C	1.6367	5.2055	1.7270
H	2.6493	4.8633	1.9129
C	0.7061	4.2837	1.1975
C	0.3720	2.1908	0.2679
C	-1.0365	2.4581	0.0712
C	-1.8457	1.5405	-0.6439
C	-3.8836	1.2361	-2.0197
H	-4.7818	0.7752	-1.6039
H	-3.2787	0.4375	-2.4265
C	-4.1895	2.2154	-3.1590
H	-4.7759	3.0670	-2.8005
H	-3.2387	2.6142	-3.5336
C	-4.9410	1.4915	-4.2799
H	-5.1332	2.1703	-5.1177
H	-5.9073	1.1110	-3.9262

C	-3.7801	2.8552	-0.1402
C	-5.1872	2.9421	-0.1481
H	-5.7877	2.3396	-0.8136
C	-5.8162	3.7660	0.7680
H	-6.9009	3.8157	0.7754
C	-5.0946	4.4794	1.7351
H	-5.6258	5.0413	2.4931
C	-3.7073	4.4118	1.7415
C	-3.5206	5.8147	3.6746
H	-4.0488	6.6390	3.1831
H	-2.6845	6.2086	4.2548
H	-4.2078	5.2782	4.3395
C	-3.0150	3.6878	0.7185
C	-1.3157	0.1801	-1.0652
H	-1.4262	0.0790	-2.1538
C	0.1529	0.0321	-0.8251
H	0.5838	-0.8820	-1.2061
C	0.9262	0.9471	-0.2248
C	-2.6026	7.8059	0.7848
H	-3.6245	7.8390	0.4028
H	-2.5950	8.1100	1.8378
H	-1.9697	8.4840	0.1999
H	-4.3633	0.6398	-4.6592
H	1.9928	0.7977	-0.0875

Ru-I4

Energy (POTENTIAL) = -2542.5952434 Eh

Atom	X	Y	Z
N	-1.9619	-2.6408	2.9845
C	-3.0827	-1.9027	3.2423
C	-3.6083	-1.7274	4.5429
C	-2.9344	-2.3675	5.6069
C	-1.8069	-3.1198	5.3343
C	-1.3452	-3.2309	4.0107
C	-3.7072	-1.2648	2.1257
H	-1.2577	-3.6169	6.1263
H	-0.4471	-3.7876	3.7770
C	-4.8385	-0.4383	2.3194
C	-5.3952	0.1755	1.1755
C	-4.8151	-0.0465	-0.0603
C	-3.6900	-0.8839	-0.1600
N	-3.1515	-1.4862	0.9009
H	-5.2135	0.4122	-0.9588
H	-3.2124	-1.0723	-1.1150
Ru	-1.3962	-2.7105	0.9207
C	-1.6650	-4.8129	0.4356
C	-1.9004	-4.0322	-0.7472
C	-0.6968	-3.3461	-1.0920
C	0.2979	-3.7180	-0.1122
C	-0.2789	-4.6312	0.8058
H	-2.8473	-3.9517	-1.2679
H	-0.5480	-2.6908	-1.9403
H	1.3086	-3.3331	-0.0697
H	0.2204	-5.0929	1.6476
H	-2.3759	-5.4716	0.9182
O	-0.4474	-0.6464	1.0424
C	0.4478	0.1425	1.3264
C	1.2355	0.1635	2.6258
C	1.5972	-1.2523	3.0690
O	1.3592	-1.7496	4.1487
H	-6.2666	0.8155	1.2786
H	-3.3015	-2.2545	6.6228
C	-4.7664	-0.8942	4.7150
C	-5.3522	-0.2711	3.6507
H	-6.2231	0.3626	3.7914
H	-5.1644	-0.7672	5.7174
O	0.7667	1.1590	0.5353
C	-0.0153	1.3090	-0.6735
H	0.3990	2.1829	-1.1756
H	0.0842	0.4202	-1.3019
H	-1.0673	1.4720	-0.4239
O	2.2837	-1.8722	2.0934
C	2.7706	-3.1932	2.4133
H	3.2703	-3.5488	1.5122
H	3.4801	-3.1422	3.2441
H	1.9434	-3.8544	2.6804
O	0.5322	7.2338	4.6352
O	1.7629	6.2293	6.8205
N	1.8913	2.8451	3.2930
N	-1.9598	3.3382	6.2112
C	0.3974	4.4199	5.1269
C	1.3272	5.0395	4.2392
C	1.4914	6.4605	4.0692
C	2.5237	6.9617	3.2991
H	2.6530	8.0296	3.1735
C	3.3959	6.0748	2.6164
H	4.2055	6.4943	2.0247
C	3.1949	4.7189	2.6435
H	3.8095	4.0336	2.0682
C	2.1181	4.1688	3.4037
C	0.8123	2.3238	3.8987
C	0.0218	3.0901	4.8319
C	-1.1588	2.5222	5.4170
C	-3.3237	2.8947	6.5417

H	-3.7141	2.3626	5.6696
H	-3.9472	3.7842	6.6569
C	-3.4140	2.0099	7.7932
H	-2.7891	1.1197	7.6585
H	-3.0004	2.5510	8.6518
C	-4.8612	1.5998	8.0752
H	-4.9243	0.9641	8.9654
H	-5.2858	1.0404	7.2320
C	-1.4320	4.4888	6.7958
C	-2.1035	5.1386	7.8550
H	-3.0709	4.7992	8.1984
C	-1.4930	6.1985	8.5058
H	-2.0135	6.6866	9.3252
C	-0.1967	6.6106	8.1792
H	0.2883	7.3814	8.7649
C	0.4662	5.9897	7.1230
C	2.4415	7.3057	7.4627
H	1.9029	8.2521	7.3370
H	3.4137	7.3735	6.9704
H	2.5868	7.1059	8.5318
C	-0.1896	5.0018	6.3283
C	-1.5037	1.2095	5.1004
H	-2.3816	0.7355	5.5156
C	-0.6979	0.4618	4.2279
H	-0.9833	-0.5584	4.0194
C	0.4380	0.9692	3.6320
C	0.6711	8.6508	4.5917
H	-0.1497	9.0452	5.1943
H	1.6267	8.9750	5.0205
H	0.5851	9.0297	3.5650
H	-5.4968	2.4781	8.2452
H	2.1734	0.6919	2.4306

2

Energy (POTENTIAL) = -1188.1402374 Eh

Atom	X	Y	Z
O	1.1109	8.5437	4.2856
O	-1.1639	7.0983	4.4987
N	2.0759	5.3810	7.7520
N	2.0615	3.8878	3.1236
C	1.2882	5.7923	5.0446
C	1.3073	6.8841	5.9633
C	1.0945	8.2654	5.6138
C	0.9741	9.2304	6.5952
H	0.8097	10.2687	6.3342
C	1.1264	8.8759	7.9615
H	1.0190	9.6517	8.7156
C	1.4637	7.6001	8.3306
H	1.6537	7.3314	9.3652
C	1.6265	6.5821	7.3393
C	2.3501	4.4504	6.8178
C	1.9757	4.6223	5.4356
C	2.3332	3.6230	4.4683
C	2.7061	3.0652	2.0879
H	3.7072	2.8127	2.4479
H	2.8530	3.6909	1.2039
C	1.9350	1.7888	1.7209
H	1.7975	1.1746	2.6181
H	0.9308	2.0565	1.3729
C	2.6703	0.9889	0.6427
H	2.1156	0.0813	0.3792
H	3.6676	0.6841	0.9845
C	1.0940	4.8265	2.7719
C	0.5709	4.8726	1.4599
H	0.9505	4.2232	0.6837
C	-0.4824	5.7240	1.1686
H	-0.8818	5.7447	0.1582
C	-1.0874	6.5055	2.1587
H	-1.9663	7.0918	1.9223
C	-0.5671	6.4823	3.4510
C	-2.2567	7.9799	4.2581
H	-1.9914	8.7636	3.5391
H	-2.4822	8.4364	5.2240
H	-3.1391	7.4357	3.8981
C	0.6144	5.7413	3.7515
C	3.0089	2.4802	4.8897
H	3.2931	1.7063	4.1899
C	3.3266	2.3072	6.2546
H	3.8434	1.3974	6.5501
C	3.0105	3.2519	7.2043
C	0.8109	9.8667	3.8548
H	0.7911	9.8240	2.7636
H	-0.1670	10.1990	4.2240
H	1.5820	10.5783	4.1777
H	2.7979	1.5801	-0.2732
H	3.2736	3.1257	8.2500

r-Ru-TS-I4

Energy (POTENTIAL) = -3037.7536827 Eh

Atom	X	Y	Z
N	-0.4264	-2.1888	2.2323
C	-1.1951	-2.3245	3.3515
C	-0.7997	-1.8506	4.6241
C	0.4626	-1.2256	4.7182

C	1.2376	-1.1049	3.5780	N	-3.0341	1.6370	-1.3156
C	0.7601	-1.5888	2.3480	C	-1.4721	3.5568	0.0931
C	-2.4582	-2.9652	3.1841	C	-0.5365	4.5674	0.5522
H	2.2114	-0.6273	3.6092	C	-0.8186	5.9665	0.5678
H	1.3361	-1.4775	1.4392	C	0.0263	6.8483	1.2353
C	-3.3335	-3.1191	4.2829	H	-0.1963	7.9071	1.2771
C	-4.5667	-3.7616	4.0418	C	1.1922	6.3576	1.8347
C	-4.8601	-4.1997	2.7620	H	1.8437	7.0444	2.3674
C	-3.9358	-3.9965	1.7245	C	1.5723	5.0315	1.7029
N	-2.7598	-3.3979	1.9261	H	2.5205	4.7099	2.1044
H	-5.7983	-4.6967	2.5397	C	0.7451	4.1404	0.9941
H	-4.1477	-4.3172	0.7124	C	0.4752	2.0683	-0.1991
Ru	-1.2711	-3.0025	0.4756	C	-0.9021	2.3742	-0.4256
C	0.3316	-4.6459	0.0588	C	-1.7426	1.4151	-1.0944
C	-0.9358	-5.2155	-0.1959	C	-3.6219	2.6990	-0.7088
C	-1.5746	-4.4700	-1.2477	C	-5.0265	2.8469	-0.8741
C	-0.6654	-3.4437	-1.6476	H	-5.5328	2.1638	-1.5485
C	0.5028	-3.5212	-0.8274	C	-5.7073	3.7876	-0.1396
H	-1.3748	-6.0478	0.3419	H	-6.7835	3.8906	-0.2491
H	-2.5352	-4.6820	-1.6977	C	-5.0436	4.5832	0.8246
H	-0.8576	-2.7108	-2.4179	H	-5.6272	5.2353	1.4626
H	1.3760	-2.8854	-0.8858	C	-3.6730	4.4801	0.9860
H	1.0354	-4.9690	0.8161	C	-3.6776	5.9682	2.8660
O	-3.5141	-0.2378	1.7297	H	-4.1527	6.7871	2.3136
C	-2.4562	-0.3278	1.1320	H	-2.9136	6.3737	3.5322
C	-2.1889	-1.2530	-0.0131	H	-4.4351	5.4377	3.4565
C	-3.3315	-1.5918	-0.9348	C	-2.9000	3.6264	0.1233
O	-3.2553	-1.7842	-2.1410	C	-1.1871	0.1324	-1.4566
H	-5.2703	-3.9027	4.8569	H	-1.7220	-0.4447	-2.1994
H	0.8073	-0.8463	5.6758	C	0.2161	-0.0001	-1.4208
C	-1.7041	-2.0167	5.7289	H	0.6605	-0.8615	-1.9051
C	-2.9171	-2.6217	5.5654	C	1.0598	0.9408	-0.8558
H	-3.5936	-2.7398	6.4069	C	-2.3532	7.7062	-0.0244
H	-1.3979	-1.6465	6.7032	H	-3.3004	7.7450	-0.5654
O	-1.3536	0.3453	1.5308	H	-2.5161	7.9915	1.0210
C	-1.5305	1.1987	2.6748	H	-1.6396	8.3987	-0.4875
H	-0.5500	1.6321	2.8699	C	2.5485	0.8909	-1.1645
H	-2.2537	1.9901	2.4616	H	3.0027	1.8462	-0.9119
H	-1.8670	0.6226	3.5401	C	2.7316	0.7978	-2.6899
O	-4.4858	-1.7736	-0.2621	C	3.3492	-0.2226	-0.4942
C	-5.5976	-2.2348	-1.0496	O	2.7878	1.7614	-3.4200
H	-6.4197	-2.3550	-0.3424	O	2.8896	-1.2035	0.0567
H	-5.8594	-1.5000	-1.8163	O	2.7861	-0.4781	-3.1016
H	-5.3682	-3.1902	-1.5315	O	4.6577	0.0211	-0.6186
O	-1.9060	6.3547	-0.1321	C	5.5514	-0.9930	-0.1037
O	-2.9885	5.0783	1.9907	H	5.4176	-1.9253	-0.6587
N	1.1783	2.8346	0.7106	H	6.5553	-0.5969	-0.2555

H	5.3634	-1.1647	0.9586
C	2.8985	-0.6784	-4.5305
H	3.8129	-0.2122	-4.9060
H	2.9318	-1.7588	-4.6692
H	2.0294	-0.2522	-5.0384
C	2.0999	2.1432	1.6673
H	1.7337	2.4028	2.6669
H	1.9076	1.0759	1.5377
C	3.6167	2.3947	1.6166
H	4.0145	2.2747	0.6080
H	3.8615	3.4157	1.9180
C	4.3167	1.4096	2.5616
H	4.0032	1.5667	3.6011
H	4.0879	0.3705	2.2966
H	5.4034	1.5369	2.5171

Ru-I1

Energy (POTENTIAL) = -2542.5675735 Eh

Atom	X	Y	Z
N	-0.0491	-2.8960	1.7058
C	-0.6552	-3.1579	2.9036
C	0.0416	-3.1959	4.1348
C	1.4311	-2.9559	4.1040
C	2.0392	-2.7034	2.8861
C	1.2701	-2.6785	1.7126
C	-2.0614	-3.3937	2.8674
H	3.1064	-2.5187	2.8194
H	1.7301	-2.4755	0.7540
C	-2.7693	-3.6745	4.0590
C	-4.1552	-3.9085	3.9507
C	-4.7484	-3.8526	2.7004
C	-3.9722	-3.5528	1.5707
N	-2.6566	-3.3279	1.6414
H	-5.8117	-4.0289	2.5758
H	-4.4187	-3.4877	0.5871
Ru	-1.3683	-2.8918	0.0382
C	-0.6278	-4.6481	-1.1603
C	-2.0449	-4.5576	-1.2900
C	-2.3737	-3.3119	-1.9180
C	-1.1323	-2.6360	-2.1822
C	-0.0592	-3.4470	-1.7254
H	-2.7575	-5.2956	-0.9400
H	-3.3582	-2.9518	-2.1810
H	-1.0443	-1.6602	-2.6397
H	0.9970	-3.2241	-1.8103
H	-0.0770	-5.4775	-0.7343
O	-2.5853	-0.5223	2.6336
C	-1.7115	-0.3767	1.7962
C	-1.7605	-0.6117	0.3318
C	-3.0916	-0.5527	-0.3364
O	-3.2473	-0.2818	-1.5258
H	-4.7380	-4.1279	4.8406
H	2.0035	-2.9726	5.0269
C	-0.6985	-3.4704	5.3361
C	-2.0433	-3.7004	5.2990
H	-2.5930	-3.9102	6.2124
H	-0.1586	-3.4940	6.2784
O	-0.4545	0.0356	2.1683
C	-0.3004	0.4021	3.5424
H	0.7632	0.6109	3.6748
H	-0.8873	1.2988	3.7703
H	-0.6104	-0.4067	4.2084
O	-4.1492	-0.8474	0.4530
C	-5.4211	-0.8773	-0.2058
H	-6.1460	-1.1268	0.5715
H	-5.6609	0.0957	-0.6454
H	-5.4409	-1.6361	-0.9951
O	-0.3268	5.9377	2.7236
O	0.0191	6.6576	0.1695
N	-2.0961	2.2333	0.2621
N	2.6712	2.7294	0.5130
C	0.1343	3.8959	0.7651
C	-1.1563	4.2721	1.2634
C	-1.4134	5.3580	2.1669
C	-2.7189	5.7035	2.4857
H	-2.9207	6.5308	3.1549
C	-3.7972	4.9473	1.9814
H	-4.8096	5.2395	2.2461
C	-3.5814	3.8183	1.2201
H	-4.3940	3.1787	0.8921
C	-2.2597	3.4368	0.8939
C	-0.8893	1.7679	0.0642
C	0.2830	2.5656	0.3137
C	1.5833	1.9915	0.1881
C	3.9969	2.0734	0.6715
H	3.8241	1.0779	1.0821
H	4.5358	2.6234	1.4435
C	4.8061	2.0013	-0.6285
H	4.2417	1.4356	-1.3787
H	4.9400	3.0095	-1.0346
C	6.1666	1.3440	-0.3848
H	6.7397	1.2846	-1.3163
H	6.0526	0.3246	0.0045
C	2.5849	4.1289	0.6269

C	3.7512	4.9185	0.6720
H	4.7369	4.4775	0.6887
C	3.6372	6.2978	0.6343
H	4.5385	6.9029	0.6606
C	2.3957	6.9280	0.4912
H	2.3507	8.0036	0.3753
C	1.2378	6.1586	0.4535
C	-0.1576	8.0738	0.1063
H	0.1791	8.5578	1.0296
H	-1.2306	8.2281	-0.0183
H	0.3753	8.5009	-0.7518
C	1.3031	4.7425	0.6495
C	1.7129	0.6175	-0.2315
H	2.6929	0.1815	-0.3622
C	0.6244	-0.1281	-0.5150
H	0.7635	-1.1425	-0.8639
C	-0.7779	0.3378	-0.4304
C	-0.4991	7.0724	3.5720
H	0.5097	7.4074	3.8201
H	-1.0371	7.8776	3.0586
H	-1.0311	6.8026	4.4925
H	6.7598	1.9156	0.3395
H	-1.1656	0.3322	-1.4625

Rh-I3-O

Energy (POTENTIAL) = -2816.8803379 Eh

Atom	X	Y	Z
O	-2.3731	-1.4163	0.9192
C	-1.9158	-0.2887	0.6011
C	-1.9861	0.3695	-0.6641
C	-2.8982	-0.0790	-1.6765
O	-2.9349	0.3680	-2.8460
O	-1.2720	0.4298	1.5689
C	-0.9220	-0.2914	2.7578
H	-0.3260	0.4041	3.3538
H	-1.8123	-0.5901	3.3201
H	-0.3334	-1.1806	2.5135
O	-3.8126	-0.9971	-1.2814
C	-4.5500	-1.6342	-2.3238
H	-5.2002	-2.3542	-1.8236
H	-5.1511	-0.9167	-2.8927
H	-3.8747	-2.1603	-3.0056
O	-1.4593	7.5315	-2.4765
O	-0.4665	6.4621	-4.7605
N	-2.4146	2.9045	-2.1872
N	2.1049	4.3554	-1.3670
C	-0.4489	4.8405	-2.4441
C	-1.8350	5.2032	-2.6000
C	-2.3370	6.5408	-2.7450
C	-3.6655	6.7590	-3.0794
H	-4.0456	7.7653	-3.2014
C	-4.5450	5.6657	-3.2216
H	-5.5792	5.8577	-3.4935
C	-4.1377	4.3749	-2.9622
H	-4.8298	3.5391	-2.9913
C	-2.7950	4.1490	-2.5841
C	-1.1860	2.6154	-1.6481
C	-0.1595	3.6060	-1.8102
C	1.1528	3.3461	-1.3041
C	3.3342	4.2267	-0.5601
H	3.0650	3.6961	0.3557
H	3.6400	5.2283	-0.2504
C	4.4774	3.5054	-1.2859
H	4.1352	2.5155	-1.6089
H	4.7355	4.0570	-2.1970
C	5.7062	3.3708	-0.3837
H	6.5228	2.8634	-0.9092
H	5.4755	2.7897	0.5179
C	1.9466	5.4168	-2.2397
C	3.0160	6.3040	-2.5091
H	3.9648	6.2075	-2.0008
C	2.8702	7.2748	-3.4815
H	3.7028	7.9403	-3.6916
C	1.7050	7.3780	-4.2561
H	1.6642	8.0854	-5.0746
C	0.6373	6.5333	-3.9873
C	-0.6598	7.4285	-5.7926
H	-0.6044	8.4501	-5.4001
H	-1.6617	7.2416	-6.1830
H	0.0756	7.3027	-6.5966
C	0.6862	5.6166	-2.8848
C	1.3996	2.1046	-0.7091
H	2.3867	1.8375	-0.3559
C	0.3757	1.1703	-0.5829
H	0.6136	0.2135	-0.1299
C	-0.9540	1.3883	-0.9860
C	-1.8639	8.8879	-2.6611
H	-0.9748	9.4879	-2.4588

H	-2.2010	9.0690	-3.6884
H	-2.6596	9.1633	-1.9581
H	6.0732	4.3539	-0.0628
Rh	-1.3600	-3.1642	-0.0760
Rh	-0.1267	-5.0292	-1.0227
O	-1.9565	-5.9423	-1.3808
C	-3.0270	-5.3357	-1.0524
O	-3.1024	-4.2013	-0.4796
C	-4.3367	-6.0005	-1.4033
H	-4.1933	-7.0654	-1.5982
H	-4.7386	-5.5261	-2.3071
H	-5.0620	-5.8561	-0.5978
O	-0.0761	-5.8491	0.8827
C	-0.6218	-5.2224	1.8482
O	-1.2203	-4.1015	1.7733
C	-0.5868	-5.8866	3.2043
H	-0.5504	-5.1350	3.9970
H	0.2674	-6.5633	3.2811
H	-1.5064	-6.4716	3.3308
O	0.4609	-2.2268	0.2675
C	1.5350	-2.8133	-0.0918
O	1.6154	-3.9681	-0.6178
C	2.8180	-2.0393	0.0957
H	3.6804	-2.7093	0.0948
H	2.9220	-1.3308	-0.7355
H	2.7827	-1.4669	1.0267
O	-1.4005	-2.3753	-1.9826
C	-0.8521	-2.9881	-2.9514
O	-0.2404	-4.1037	-2.8779
C	-0.9512	-2.3324	-4.3072
H	-1.5464	-2.9691	-4.9721
H	-1.4232	-1.3517	-4.2170
H	0.0474	-2.2367	-4.7463
H	-2.9817	2.0845	-2.4461

Rh-TS-I1
Energy (POTENTIAL) = -2816.8262732 Eh

Atom	X	Y	Z
O	-3.2339	-0.6635	2.6071
C	-2.1279	-0.5226	2.1153
C	-1.7731	-0.5342	0.6796
C	-2.8206	-0.2895	-0.2977
O	-2.4526	0.2079	-1.4141
O	-1.0161	-0.3393	2.8810
C	-1.2461	-0.3321	4.2940
H	-0.2654	-0.1923	4.7533
H	-1.9133	0.4885	4.5792
H	-1.6837	-1.2795	4.6247
O	-4.0769	-0.5778	-0.0670
C	-4.9906	-0.4516	-1.1772
H	-5.9563	-0.7783	-0.7906
H	-5.0465	0.5886	-1.5094
H	-4.6712	-1.0941	-1.9996
O	0.0508	6.2667	2.6940
O	-0.1551	6.6861	0.0496
N	-1.9263	2.2324	1.0329
N	2.7846	2.9585	0.2980
C	0.2615	4.0251	0.9173
C	-0.9292	4.3999	1.6151
C	-1.0853	5.5806	2.4226
C	-2.3243	5.9136	2.9420
H	-2.4487	6.8054	3.5437
C	-3.4343	5.0601	2.7364
H	-4.3969	5.3479	3.1507
C	-3.2973	3.8602	2.0810
H	-4.1194	3.1586	1.9816
C	-2.0318	3.4799	1.5565
C	-0.7358	1.7869	0.6598
C	0.4084	2.6585	0.5901
C	1.6958	2.1237	0.2623
C	4.1453	2.3815	0.2445
H	4.1220	1.4342	0.7862
H	4.8038	3.0368	0.8171
C	4.6747	2.1814	-1.1815
H	4.0016	1.5119	-1.7290
H	4.6592	3.1395	-1.7131
C	6.0935	1.6079	-1.1653
H	6.4642	1.4544	-2.1847
H	6.1253	0.6414	-0.6468
C	2.6215	4.3504	0.2628
C	3.7154	5.1978	-0.0116
H	4.7157	4.8073	-0.1315
C	3.5006	6.5555	-0.1831
H	4.3466	7.2006	-0.4027
C	2.2139	7.1035	-0.1500
H	2.0685	8.1514	-0.3800
C	1.1298	6.2785	0.1348
C	-0.4362	8.0791	-0.0780
H	0.0571	8.6594	0.7102
H	-1.5189	8.1698	0.0256
H	-0.1311	8.4591	-1.0609
C	1.3302	4.9053	0.4742
C	1.8153	0.7432	-0.0197
H	2.7729	0.3108	-0.2730

C	0.7078	-0.0805	0.0015
H	0.8489	-1.1268	-0.2204
C	-0.6154	0.3794	0.2438
C	-0.0287	7.4581	3.4723
H	0.9874	7.8563	3.5031
H	-0.6961	8.1962	3.0120
H	-0.3690	7.2443	4.4935
H	6.7892	2.2850	-0.6537
H	-1.2368	0.4312	-0.9789
Rh	-1.4239	-2.8171	0.2481
Rh	-0.9676	-5.1934	-0.1999
O	-2.7918	-5.2822	-1.1834
C	-3.4871	-4.2241	-1.2887
O	-3.1938	-3.0789	-0.8158
C	-4.7784	-4.3123	-2.0689
H	-5.0900	-5.3521	-2.1876
H	-4.6231	-3.8731	-3.0618
H	-5.5609	-3.7375	-1.5655
O	-1.9120	-5.6264	1.5954
C	-2.3847	-4.6706	2.2900
O	-2.3392	-3.4322	2.0019
C	-3.1008	-5.0432	3.5674
H	-3.1749	-4.1815	4.2342
H	-2.5843	-5.8683	4.0656
H	-4.1136	-5.3808	3.3153
O	0.3835	-2.7825	1.2744
C	1.1002	-3.8340	1.3365
O	0.8295	-4.9612	0.8170
C	2.3788	-3.7205	2.1338
H	3.0667	-4.5313	1.8844
H	2.8513	-2.7506	1.9540
H	2.1328	-3.7809	3.2010
O	-0.4732	-2.3982	-1.5537
C	-0.0049	-3.3543	-2.2562
O	-0.0542	-4.5900	-1.9701
C	0.6541	-2.9570	-3.5565
H	-0.1153	-2.6042	-4.2534
H	1.3467	-2.1278	-3.3815
H	1.1829	-3.8018	-4.0023

r-Ru-TS-I1

Energy (POTENTIAL) = -2542.5300979 Eh

Atom	X	Y	Z
N	-0.3243	-1.3776	1.7015
C	-1.0260	-1.3760	2.8727
C	-0.7269	-0.5145	3.9528
C	0.3556	0.3779	3.7899
C	1.0572	0.3715	2.5983
C	0.6878	-0.5186	1.5748
C	-2.1308	-2.2772	2.9600
H	1.8898	1.0467	2.4318
H	1.2140	-0.5285	0.6290
C	-2.9318	-2.3152	4.1242
C	-4.0264	-3.2074	4.1288
C	-4.2724	-3.9763	3.0055
C	-3.4318	-3.8658	1.8850
N	-2.3768	-3.0502	1.8627
H	-5.1111	-4.6634	2.9688
H	-3.6167	-4.4333	0.9815
Ru	-0.9731	-2.8433	0.3012
C	0.7550	-4.2238	0.4584
C	-0.4294	-4.9950	0.2564
C	-0.9816	-4.6640	-1.0219
C	-0.1099	-3.6839	-1.6145
C	0.9596	-3.4162	-0.7220
H	-0.8573	-5.6891	0.9702
H	-1.8499	-5.1146	-1.4821
H	-0.2688	-3.2106	-2.5747
H	1.7820	-2.7328	-0.8915
H	1.4012	-4.2618	1.3266
O	-4.3023	-1.0380	0.8339
C	-3.2144	-0.6337	0.4537
C	-2.4155	-1.1582	-0.6799
C	-3.0149	-2.1345	-1.5629
O	-2.6295	-2.1002	-2.7910
H	-4.6659	-3.2724	5.0043
H	0.6199	1.0585	4.5939
C	-1.5428	-0.5806	5.1338
C	-2.5973	-1.4440	5.2169
H	-3.2108	-1.4822	6.1125
H	-1.3022	0.0790	5.9625
O	-2.5847	0.4080	1.0569
C	-3.3118	1.0542	2.1146
H	-2.6871	1.8929	2.4244
H	-4.2771	1.4182	1.7535
H	-3.4706	0.3722	2.9535
O	-3.9667	-2.9622	-1.1871
C	-4.5177	-3.8429	-2.1943
H	-5.2443	-4.4589	-1.6644
H	-5.0123	-3.2589	-2.9748
H	-3.7376	-4.4656	-2.6369
O	-3.1674	6.1543	-2.4241
O	-2.9266	5.7272	0.2224
N	0.7074	3.3459	-2.0433

N	-3.6685	1.3991	-1.6064
C	-2.0744	3.6734	-1.5464
C	-1.2904	4.7850	-1.9813
C	-1.8202	6.0762	-2.3275
C	-0.9678	7.1280	-2.6180
H	-1.3626	8.1041	-2.8712
C	0.4307	6.9216	-2.6357
H	1.0787	7.7638	-2.8632
C	0.9714	5.6746	-2.4258
H	2.0384	5.4913	-2.4992
C	0.1240	4.5693	-2.1449
C	-0.0730	2.2800	-1.8924
C	-1.4972	2.3871	-1.6825
C	-2.3053	1.2126	-1.6250
C	-4.5922	0.4422	-2.2625
H	-5.2214	-0.0510	-1.5161
H	-3.9782	-0.3152	-2.7326
C	-5.4196	1.0940	-3.3788
H	-6.0661	1.8816	-2.9810
H	-4.7303	1.5712	-4.0866
C	-6.2663	0.0366	-4.0932
H	-6.8606	0.4920	-4.8929
H	-6.9594	-0.4528	-3.3975
C	-4.1974	2.5598	-1.0262
C	-5.5103	2.5579	-0.5144
H	-6.1409	1.6840	-0.6096
C	-5.9636	3.6645	0.1835
H	-6.9684	3.6576	0.5959
C	-5.1329	4.7659	0.4294
H	-5.4929	5.5815	1.0440
C	-3.8375	4.7774	-0.0789
C	-3.3447	6.8940	0.9294
H	-4.1642	7.4034	0.4094
H	-2.4703	7.5468	0.9586
H	-3.6525	6.6502	1.9538
C	-3.3787	3.7106	-0.9119
C	-1.6985	-0.1078	-1.5702
H	-2.0246	-0.9970	-2.6434
C	-0.2489	-0.1253	-1.6794
H	0.2181	-1.0977	-1.6155
C	0.5173	0.9736	-1.8967
C	-3.7735	7.4284	-2.6321
H	-4.8497	7.2547	-2.5740
H	-3.4741	8.1439	-1.8573
H	-3.5227	7.8325	-3.6208
H	-5.6371	-0.7406	-4.5446
H	1.5911	0.9038	-2.0406

Rh-I2

Energy (POTENTIAL) = -2816.8564804 Eh

Atom	X	Y	Z
O	-3.0068	-1.1692	3.1488
C	-1.9175	-0.8941	2.6758
C	-1.5973	-0.5827	1.2603
C	-2.6784	-0.2608	0.3885
O	-2.4522	0.0521	-0.8757
O	-0.7901	-0.8694	3.4351
C	-0.9729	-1.2411	4.8074
H	0.0153	-1.1703	5.2657
H	-1.6687	-0.5619	5.3103
H	-1.3525	-2.2649	4.8862
O	-3.9268	-0.2800	0.7480
C	-4.9503	-0.0877	-0.2593
H	-5.8857	-0.3258	0.2465
H	-4.9491	0.9543	-0.5892
H	-4.7833	-0.7592	-1.1006
O	-0.1677	6.5520	2.1845
O	-0.2883	6.4379	-0.5059
N	-1.8652	2.1679	1.2984
N	2.8522	3.0319	0.5616
C	0.2373	4.0253	0.8854
C	-0.9999	4.4572	1.4535
C	-1.2572	5.7636	2.0036
C	-2.5333	6.1235	2.3943
H	-2.7298	7.1059	2.8053
C	-3.5946	5.1846	2.3137
H	-4.5901	5.4967	2.6193
C	-3.3682	3.8910	1.9223
H	-4.1515	3.1391	1.9271
C	-2.0537	3.4770	1.5452
C	-0.6198	1.7200	1.0668
C	0.4749	2.6324	0.8529
C	1.7984	2.1212	0.6432
C	4.2332	2.5360	0.6725
H	4.2267	1.7082	1.3866
H	4.8391	3.3223	1.1294
C	4.8488	2.0866	-0.6606
H	4.2242	1.3022	-1.1035
H	4.8403	2.9249	-1.3670
C	6.2782	1.5757	-0.4668
H	6.7125	1.2518	-1.4193
H	6.3053	0.7213	0.2212
C	2.6076	4.3651	0.2420
C	3.6595	5.2187	-0.1606

H	4.6847	4.8761	-0.1708
C	3.3750	6.5006	-0.6014
H	4.1911	7.1451	-0.9167
C	2.0585	6.9596	-0.7149
H	1.8609	7.9308	-1.1507
C	1.0152	6.1330	-0.3038
C	-0.6396	7.7635	-0.8923
H	-0.2141	8.5065	-0.2079
H	-1.7296	7.8049	-0.8432
H	-0.3154	7.9822	-1.9176
C	1.2769	4.8684	0.3034
C	1.9846	0.7425	0.6159
H	2.9649	0.3126	0.4590
C	0.8959	-0.1360	0.7845
H	1.0923	-1.1982	0.7551
C	-0.3943	0.3005	1.0031
C	-0.3456	7.8641	2.7080
H	0.6465	8.3201	2.7038
H	-1.0223	8.4610	2.0842
H	-0.7294	7.8352	3.7361
H	6.9266	2.3579	-0.0518
H	-1.5666	-0.2966	-1.1440
Rh	-1.3929	-2.8327	0.3546
Rh	-1.0559	-4.9751	-0.7762
O	-2.9397	-4.7631	-1.6117
C	-3.6233	-3.7312	-1.3275
O	-3.2609	-2.7819	-0.5573
C	-4.9808	-3.5877	-1.9715
H	-5.3069	-4.5347	-2.4060
H	-4.9176	-2.8337	-2.7655
H	-5.7094	-3.2389	-1.2343
O	-1.8916	-5.8784	0.8886
C	-2.2632	-5.1547	1.8686
O	-2.1870	-3.8855	1.9407
C	-2.8825	-5.8669	3.0467
H	-2.8371	-5.2436	3.9422
H	-2.3780	-6.8214	3.2194
H	-3.9346	-6.0753	2.8167
O	0.4760	-3.1348	1.2165
C	1.1622	-4.1651	0.9110
O	0.8060	-5.0898	0.1164
C	2.5291	-4.2652	1.5429
H	2.9613	-5.2555	1.3870
H	3.1833	-3.5103	1.0904
H	2.4607	-4.0471	2.6129
O	-0.5965	-1.8980	-1.3234
C	-0.2130	-2.6116	-2.3190
O	-0.2427	-3.8746	-2.3661
C	0.3007	-1.8530	-3.5156
H	-0.5388	-1.3340	-3.9936
H	1.0191	-1.0941	-3.1904
H	0.7639	-2.5288	-4.2366

r-Ru-I2

Energy (POTENTIAL) = -2542.5587806 Eh

Atom	X	Y	Z
N	-0.3928	-1.5330	1.8482
C	-1.1394	-1.6727	2.9815
C	-0.9068	-0.9226	4.1566
C	0.1559	0.0068	4.1323
C	0.8999	0.1471	2.9756
C	0.5951	-0.6379	1.8496
C	-2.2209	-2.6035	2.9274
H	1.7167	0.8583	2.9145
H	1.1548	-0.5316	0.9291
C	-3.0691	-2.7759	4.0447
C	-4.1345	-3.6934	3.9139
C	-4.3027	-4.3630	2.7158
C	-3.4163	-4.1248	1.6516
N	-2.3954	-3.2728	1.7501
H	-5.1126	-5.0710	2.5772
H	-3.5357	-4.6261	0.6998
Ru	-0.9295	-2.8738	0.2840
C	0.7448	-4.2851	0.5020
C	-0.3795	-5.0074	-0.0126
C	-0.7112	-4.4828	-1.2942
C	0.2394	-3.4367	-1.5882
C	1.1371	-3.3212	-0.5016
H	-0.9145	-5.7921	0.5089
H	-1.4874	-4.8399	-1.9550
H	0.2597	-2.8444	-2.4935
H	1.9620	-2.6241	-0.4254
H	1.2454	-4.4670	1.4447
O	-4.3094	-1.0883	0.9561
C	-3.2205	-0.6345	0.6490
C	-2.3745	-1.0255	-0.5235
C	-2.8781	-2.0253	-1.3911
O	-2.4522	-2.1323	-2.6537
H	-4.8093	-3.8588	4.7485
H	0.3700	0.6046	5.0134
C	-1.7695	-1.1259	5.2869
C	-2.8057	-2.0134	5.2335
H	-3.4566	-2.1562	6.0911
H	-1.5803	-0.5500	6.1882

O	-2.6189	0.3376	1.3718
C	-3.3879	0.8629	2.4666
H	-2.7594	1.6320	2.9166
H	-4.3198	1.3046	2.1031
H	-3.6120	0.0802	3.1959
O	-3.9225	-2.7737	-1.1045
C	-4.4128	-3.7219	-2.0803
H	-5.2712	-4.1915	-1.6001
H	-4.7245	-3.2062	-2.9915
H	-3.6527	-4.4703	-2.3122
O	-3.3106	6.1301	-2.7116
O	-2.8097	6.0441	-0.0504
N	0.6645	3.4590	-2.3707
N	-3.6329	1.5239	-1.2804
C	-2.0733	3.8021	-1.6446
C	-1.3650	4.8529	-2.2915
C	-1.9558	6.0911	-2.7317
C	-1.1657	7.1147	-3.2165
H	-1.6082	8.0485	-3.5419
C	0.2357	6.9294	-3.3460
H	0.8359	7.7521	-3.7261
C	0.8252	5.7267	-3.0542
H	1.8861	5.5546	-3.2062
C	0.0387	4.6373	-2.5677
C	-0.0801	2.4005	-2.0065
C	-1.4738	2.5206	-1.6743
C	-2.2449	1.3553	-1.3656
C	-4.5526	0.5957	-1.9767
H	-5.2004	0.0846	-1.2569
H	-3.9321	-0.1590	-2.4532
C	-5.3706	1.2720	-3.0835
H	-6.0157	2.0492	-2.6618
H	-4.6791	1.7705	-3.7748
C	-6.2201	0.2406	-3.8313
H	-6.8079	0.7179	-4.6233
H	-6.9197	-0.2633	-3.1526
C	-4.1232	2.7356	-0.7862
C	-5.3899	2.7872	-0.1738
H	-6.0094	1.8988	-0.1329
C	-5.8061	3.9674	0.4229
H	-6.7768	4.0076	0.9095
C	-4.9723	5.0927	0.4686
H	-5.2908	5.9753	1.0092
C	-3.7229	5.0500	-0.1492
C	-3.1883	7.2786	0.5527
H	-4.0666	7.7131	0.0612
H	-2.3328	7.9436	0.4195
H	-3.3914	7.1557	1.6241
C	-3.3199	3.8988	-0.8874
C	-1.6087	0.1198	-1.1534
H	-1.7705	-1.4466	-2.8188
C	-0.2169	0.0469	-1.4385
H	0.2710	-0.9018	-1.2595
C	0.5214	1.1173	-1.8851
C	-3.9675	7.3658	-2.9757
H	-5.0256	7.1860	-2.7748
H	-3.5996	8.1618	-2.3173
H	-3.8426	7.6710	-4.0227
H	-5.5930	-0.5297	-4.2984
H	1.5778	1.0187	-2.1156

I0-Ru

Energy (POTENTIAL) = -1354.4007495 Eh

Atom	X	Y	Z
N	-0.1608	-2.9999	0.5237
C	-0.1272	-3.7615	1.6545
C	1.0203	-4.4771	2.0636
C	2.1659	-4.3791	1.2441
C	2.1188	-3.5969	0.1032
C	0.9372	-2.9138	-0.2288
C	-1.3120	-3.7863	2.4443
H	2.9834	-3.4941	-0.5432
H	0.8744	-2.2825	-1.1069
C	-1.3535	-4.5277	3.6455
C	-2.5530	-4.4826	4.3889
C	-3.6111	-3.7228	3.9219
C	-3.4838	-3.0126	2.7165
N	-2.3650	-3.0487	1.9920
H	-4.5431	-3.6588	4.4725
H	-4.2909	-2.4016	2.3303
Ru	-2.0127	-2.0555	0.1659
C	-2.4102	-3.1372	-1.9332
C	-3.5652	-3.3307	-1.1564
C	-4.0846	-2.0346	-0.7974
C	-3.2621	-1.0471	-1.4201
C	-2.1921	-1.7230	-2.0861
H	-3.9625	-4.2839	-0.8307
H	-4.9893	-1.8460	-0.2336
H	-3.4191	0.0222	-1.3989
H	-1.4092	-1.2580	-2.6703
H	-1.7596	-3.9172	-2.3098
O	-0.8019	-0.6944	3.2821
C	-0.4425	-0.5134	2.1347
C	-1.3767	-0.4598	0.9672

C	-1.6937	0.9224	0.5006
O	-1.3613	1.3731	-0.5803
H	-2.6313	-5.0359	5.3201
H	3.0718	-4.9111	1.5193
C	0.9536	-5.2396	3.2799
C	-0.1831	-5.2642	4.0364
H	-0.2209	-5.8382	4.9573
H	1.8347	-5.7950	3.5872
O	0.8341	-0.4047	1.7388
C	1.8364	-0.6057	2.7638
H	2.7929	-0.5618	2.2434
H	1.7744	0.1829	3.5184
H	1.7021	-1.5814	3.2382
O	-2.3826	1.5999	1.4301
C	-2.7508	2.9561	1.0805
H	-3.2899	3.3407	1.9462
H	-1.8567	3.5547	0.8880
H	-3.3938	2.9590	0.1960

3a

Energy (POTENTIAL) = -1683.3729485 Eh

Atom	X	Y	Z
O	0.8734	8.4898	4.1938
O	-1.2854	6.9172	4.5557
O	3.8509	1.0992	9.4900
O	5.6929	2.1233	8.6777
O	4.4949	4.9448	10.2708
O	5.2541	5.0938	8.1506
N	2.2082	5.5003	7.6849
N	2.0559	3.8506	3.1212
C	1.2667	5.7758	5.0182
C	1.2636	6.8980	5.9027
C	0.9465	8.2515	5.5273
C	0.8199	9.2374	6.4877
H	0.5767	10.2549	6.2072
C	1.0709	8.9385	7.8519
H	0.9613	9.7312	8.5876
C	1.5044	7.6965	8.2380
H	1.7719	7.4707	9.2656
C	1.6683	6.6617	7.2674
C	2.4940	4.5609	6.7690
C	2.0443	4.6622	5.4071
C	2.4155	3.6549	4.4551
C	2.6787	3.0135	2.0832
H	3.7107	2.8248	2.3909
H	2.7419	3.6045	1.1661
C	1.9468	1.6894	1.8222
H	1.8941	1.1127	2.7528
H	0.9116	1.8987	1.5291
C	2.6472	0.8717	0.7344
H	2.1217	-0.0728	0.5537
H	3.6792	0.6312	1.0196
C	1.0181	4.7197	2.7913
C	0.4287	4.6881	1.5073
H	0.8090	4.0350	0.7346
C	-0.6877	5.4641	1.2436
H	-1.1379	5.4248	0.2554
C	-1.2888	6.2440	2.2374
H	-2.2119	6.7688	2.0273
C	-0.7046	6.2996	3.5007
C	-2.4729	7.6777	4.3526
H	-2.3315	8.4557	3.5931
H	-2.6866	8.1466	5.3151
H	-3.3151	7.0350	4.0657
C	0.5337	5.6395	3.7648
C	3.1873	2.5784	4.8831
H	3.4878	1.7948	4.2012
C	3.5919	2.4793	6.2312
H	4.1899	1.6157	6.5087
C	3.2658	3.4277	7.1757
C	3.6667	3.3639	8.6380
H	2.7684	3.3842	9.2608
C	4.3879	2.0674	8.9945
C	6.4643	0.9407	8.9772
H	6.4306	0.7243	10.0487
H	7.4839	1.1720	8.6675
C	4.4977	4.5610	9.1181
C	6.0283	6.2457	8.5327
H	6.5766	6.5407	7.6370
H	5.3676	7.0556	8.8564
C	0.4184	9.7620	3.7453
H	0.3237	9.6765	2.6608
H	-0.5568	10.0143	4.1788
H	1.1387	10.5545	3.9864
H	6.7221	5.9956	9.3405
H	6.0808	0.0814	8.4199
H	2.6835	1.4221	-0.2145

Ter2

Energy (POTENTIAL) = -1904.26272385 Eh

Atom	X	Y	Z
O	0.8051	8.5559	4.0830
O	-1.4359	7.0326	3.8722
O	1.0207	1.5217	10.0334

O	4.0888	4.9352	9.2294
O	3.1604	3.9203	11.0359
N	1.1405	5.4754	7.7300
N	2.0837	3.9004	3.2320
C	0.9031	5.8183	4.9184
C	0.6971	6.9250	5.7975
C	0.5185	8.2930	5.3836
C	0.1611	9.2656	6.2997
H	0.0214	10.2942	5.9918
C	0.0284	8.9310	7.6735
H	-0.2644	9.7097	8.3729
C	0.3240	7.6709	8.1312
H	0.3005	7.4243	9.1880
C	0.7320	6.6533	7.2138
C	1.6414	4.5502	6.8963
C	1.5374	4.6795	5.4638
C	2.0987	3.6746	4.6047
C	2.9348	3.0701	2.3608
H	3.8494	2.8461	2.9158
H	3.2422	3.6810	1.5090
C	2.2689	1.7724	1.8815
H	1.9482	1.1840	2.7482
H	1.3609	2.0135	1.3179
C	3.2247	0.9500	1.0130
H	2.7463	0.0264	0.6697
H	4.1276	0.6723	1.5707
C	1.1886	4.8120	2.6748
C	0.9432	4.8284	1.2823
H	1.4927	4.1858	0.6092
C	-0.0560	5.6418	0.7705
H	-0.2438	5.6397	-0.2995
C	-0.8741	6.4125	1.6067
H	-1.7054	6.9654	1.1881
C	-0.6218	6.4233	2.9772
C	-2.4843	7.8811	3.4005
H	-2.0952	8.6642	2.7400
H	-2.9158	8.3376	4.2929
H	-3.2562	7.3049	2.8762
C	0.4972	5.7237	3.5223
C	2.7081	2.5544	5.1755
H	3.1351	1.7715	4.5645
C	2.7840	2.4286	6.5738
H	3.2710	1.5509	6.9901
C	2.2811	3.3872	7.4367
C	2.4244	3.2578	8.9126
C	1.6775	2.3459	9.5341
C	3.3042	4.1272	9.6909
C	4.0132	4.6994	11.8748
H	5.0665	4.5584	11.6187
C	0.5984	9.8751	3.5762
H	0.8116	9.8157	2.5073
H	-0.4367	10.2030	3.7255
H	1.2832	10.5922	4.0456
H	3.7575	5.7612	11.8254
H	3.5399	1.5135	0.1261
C	3.7917	4.2102	13.2906
F	4.5733	4.9175	14.1443
F	4.1083	2.9010	13.4424
F	2.5091	4.3572	13.7007

Ru-I5

Energy (POTENTIAL) = -3037.7973817 Eh

Atom	X	Y	Z
N	-0.5992	-1.9385	2.2615
C	-1.4966	-2.0997	3.2792
C	-1.3444	-1.4973	4.5494
C	-0.2051	-0.6883	4.7525
C	0.6945	-0.5267	3.7150
C	0.4664	-1.1686	2.4855
C	-2.6434	-2.9007	2.9984
H	1.5777	0.0928	3.8130
H	1.1580	-1.0356	1.6664
C	-3.6318	-3.1057	3.9886
C	-4.7406	-3.9070	3.6412
C	-4.8086	-4.4421	2.3671
C	-3.7871	-4.1759	1.4403
N	-2.7247	-3.4268	1.7417
H	-5.6455	-5.0623	2.0634
H	-3.8276	-4.5638	0.4317
Ru	-1.0849	-2.9985	0.4841
C	0.5910	-4.4682	0.4815
C	-0.6075	-5.1321	0.0873
C	-1.0737	-4.5473	-1.1388
C	-0.1390	-3.5200	-1.4929
C	0.8938	-3.4688	-0.5147
H	-1.0998	-5.9236	0.6401
H	-1.9409	-4.8466	-1.7108
H	-0.2423	-2.8725	-2.3537
H	1.7603	-2.8209	-0.5236
H	1.1793	-4.6869	1.3642
O	-4.2198	-0.9470	1.0792
C	-3.0786	-0.6366	0.7778
C	-2.2702	-1.0817	-0.3830
C	-2.9549	-1.8570	-1.4480

O	-2.5823	-1.8711	-2.6256
H	-5.5232	-4.0919	4.3713
H	-0.0538	-0.1997	5.7110
C	-2.3592	-1.7188	5.5423
C	-3.4550	-2.4882	5.2737
H	-4.2180	-2.6471	6.0304
H	-2.2360	-1.2530	6.5160
O	-2.3713	0.2406	1.5608
C	-3.0955	0.8239	2.6551
H	-2.4027	1.5252	3.1216
H	-3.9852	1.3503	2.2987
H	-3.3972	0.0584	3.3736
O	-4.0475	-2.5544	-1.0659
C	-4.6895	-3.3161	-2.0978
H	-5.5327	-3.8100	-1.6110
H	-5.0497	-2.6645	-2.9001
H	-4.0114	-4.0617	-2.5240
O	-2.0542	6.4259	0.4088
O	-2.7071	5.0017	2.5890
N	1.2593	2.9746	0.5528
N	-3.1522	1.8120	-0.8946
C	-1.4918	3.6168	0.4417
C	-0.5311	4.6450	0.7239
C	-0.8230	6.0466	0.8120
C	0.1598	6.9381	1.2236
H	-0.0612	7.9948	1.3109
C	1.4660	6.4823	1.4873
H	2.2154	7.2002	1.8086
C	1.8156	5.1621	1.2870
H	2.8355	4.8120	1.4068
C	0.8366	4.2385	0.8591
C	0.4126	2.1119	0.0463
C	-1.0029	2.3843	-0.0508
C	-1.8716	1.4504	-0.6657
C	-3.9848	1.1275	-1.9213
H	-4.8706	0.7120	-1.4367
H	-3.4208	0.2941	-2.3180
C	-4.3285	2.0582	-3.0901
H	-4.8609	2.9480	-2.7401
H	-3.3908	2.4018	-3.5439
C	-5.1761	1.3085	-4.1217
H	-5.3979	1.9515	-4.9803
H	-6.1301	0.9835	-3.6884
C	-3.7454	2.8329	-0.1308
C	-5.1485	2.9550	-0.0727
H	-5.7950	2.3408	-0.6820
C	-5.7104	3.8306	0.8389
H	-6.7918	3.9090	0.8987
C	-4.9233	4.5627	1.7385
H	-5.4018	5.1687	2.4973
C	-3.5393	4.4618	1.6772
C	-3.2310	5.9143	3.5559
H	-3.7666	6.7396	3.0738
H	-2.3625	6.3030	4.0900
H	-3.8965	5.4029	4.2616
C	-2.9178	3.6820	0.6504
C	-1.3902	0.0635	-1.0405
H	-1.5527	-0.0810	-2.1187
C	0.0898	-0.0926	-0.9006
H	0.4812	-1.0170	-1.2974
C	0.9362	0.8420	-0.4410
C	-2.4486	7.7906	0.5563
H	-3.4933	7.8269	0.2426
H	-2.3667	8.1180	1.5990
H	-1.8501	8.4481	-0.0858
H	-4.6542	0.4175	-4.4918
C	2.4391	0.6520	-0.5362
H	2.6488	-0.4187	-0.6392
C	3.1803	1.0753	0.7382
C	2.9987	1.3489	-1.7785
O	2.9359	0.5997	1.8293
O	2.4892	2.2912	-2.3466
O	4.1629	1.9496	0.5043
O	4.1352	0.7527	-2.1708
C	4.8049	1.3566	-3.2970
H	5.0676	2.3942	-3.0718
H	5.7054	0.7616	-3.4537
H	4.1678	1.3253	-4.1856
C	4.8760	2.4136	1.6683
H	5.3940	1.5835	2.1567
H	5.5944	3.1453	1.2979
H	4.1826	2.8786	2.3744

Ter-TS1

Energy (POTENTIAL) = -2357.08215170 Eh

Atom	X	Y	Z
O	1.3452	8.4571	4.2097
O	-0.9124	7.0853	4.7196
O	3.3052	2.2518	10.4094
O	1.7997	2.3599	8.7079
O	6.2084	3.4663	7.5614
O	5.7751	3.2243	9.7587
N	2.7345	5.2301	7.4080
N	1.9173	3.7107	2.9031

C	1.5410	5.6911	4.8969
C	1.7375	6.7748	5.8255
C	1.5310	8.1652	5.5191
C	1.6128	9.1301	6.5113
H	1.4439	10.1744	6.2817
C	1.9775	8.7632	7.8288
H	2.0282	9.5358	8.5912
C	2.3275	7.4694	8.1408
H	2.6780	7.1910	9.1293
C	2.2691	6.4701	7.1336
C	2.8201	4.3054	6.4357
C	2.2435	4.4953	5.1574
C	2.4116	3.4627	4.1806
C	2.3262	2.8259	1.7970
H	3.3560	2.5165	1.9917
H	2.3593	3.4198	0.8813
C	1.4244	1.5969	1.6153
H	1.4098	1.0162	2.5443
H	0.3942	1.9242	1.4353
C	1.9103	0.7249	0.4546
H	1.2683	-0.1534	0.3286
H	2.9339	0.3702	0.6265
C	0.9669	4.7067	2.6977
C	0.2604	4.7834	1.4738
H	0.4872	4.1170	0.6542
C	-0.7816	5.6851	1.3339
H	-1.3227	5.7235	0.3927
C	-1.2038	6.4960	2.3970
H	-2.0851	7.1146	2.2865
C	-0.5009	6.4533	3.5972
C	-2.0194	7.9883	4.6534
H	-1.8470	8.7768	3.9123
H	-2.0941	8.4309	5.6479
H	-2.9492	7.4561	4.4199
C	0.6827	5.6593	3.7280
C	3.1133	2.3049	4.5373
H	3.2580	1.5029	3.8263
C	3.6313	2.1381	5.8438
H	4.1504	1.2129	6.0791
C	3.4910	3.1174	6.8071
C	3.9355	3.1716	8.2452
C	3.0666	2.5392	9.2497
C	0.7199	2.1522	9.6043
H	0.0409	1.4185	9.1651
C	5.3878	3.2751	8.4523
C	7.1695	3.3999	9.9867
H	7.7653	2.6302	9.4883
C	1.0428	9.8013	3.8284
H	0.8400	9.7626	2.7568
H	0.1585	10.1747	4.3569
H	1.8945	10.4660	4.0169
H	7.5115	4.3890	9.6687
H	1.0545	1.8183	10.5891
H	1.9041	1.2834	-0.4896
C	-0.0288	3.4658	9.7612
C	7.3782	3.2763	11.4819
F	8.6931	3.4530	11.7770
F	7.0209	2.0609	11.9626
F	6.6825	4.2016	12.1867
F	-0.5266	3.9220	8.5845
F	-1.0778	3.3189	10.6115
F	0.7618	4.4498	10.2576
H	3.4210	4.4916	8.3039

4aa

Energy (POTENTIAL) = -2178.5974674 Eh

Atom	X	Y	Z
O	8.4804	15.1984	9.3623
O	9.0124	13.3656	10.5722
O	7.0818	14.2303	6.7849
O	9.2769	14.0386	6.2917
O	6.9158	6.0636	9.8886
O	5.4456	6.0606	7.6103
N	3.9135	9.9361	9.9710
N	8.1517	10.0707	7.6136
C	6.1099	8.5621	8.7994
C	4.8597	7.9010	8.9752
C	4.5486	6.5845	8.4811
C	3.3610	5.9676	8.8243
H	3.1287	4.9744	8.4599
C	2.4040	6.6560	9.6143
H	1.4795	6.1461	9.8725
C	2.6034	7.9564	10.0006
H	1.8533	8.5175	10.5490
C	3.8108	8.6311	9.6472
C	4.9879	10.6148	9.5435
C	5.0295	12.0331	9.7321
C	6.1215	12.7269	9.2796
H	6.1585	13.8036	9.4049
C	7.2312	12.1160	8.6373
C	8.4279	12.9973	8.3107
H	9.3322	12.3881	8.2468
C	8.6483	13.9982	9.4526
C	8.3288	13.8053	7.0138

C	6.8969	15.0477	5.6107
H	7.4776	15.9703	5.6974
H	7.2028	14.5034	4.7134
H	5.8300	15.2698	5.5779
C	7.2034	10.7444	8.4016
C	8.5246	10.6055	6.2759
H	9.1984	11.4584	6.3673
H	9.0842	9.8186	5.7702
C	7.3086	10.9681	5.4120
H	7.7035	11.3827	4.4750
H	6.7320	11.7691	5.8877
C	8.3997	8.7194	7.8921
C	7.4123	7.9466	8.5551
C	6.1069	9.9711	8.9169
C	9.6351	8.1432	7.5385
H	10.3874	8.7366	7.0314
C	9.9113	6.8366	7.9092
C	9.0090	6.0932	8.6799
H	9.2842	5.1045	9.0250
C	7.7858	6.6575	9.0379
C	7.1487	4.7171	10.2931
H	6.2728	4.4322	10.8795
H	7.2414	4.0482	9.4293
H	8.0471	4.6356	10.9179
C	5.2730	4.7178	7.1692
H	5.2144	4.0220	8.0150
H	4.3747	4.6126	6.5474
H	6.1569	4.4881	6.5705
C	6.3991	9.7722	5.1180
H	6.9571	8.9609	4.6337
H	5.9557	9.3700	6.0352
H	5.5777	10.0615	4.4523
C	9.0874	14.1856	11.7600
H	8.1083	14.6251	11.9722
H	9.3809	13.5078	12.5618
H	9.8304	14.9780	11.6361
H	10.8701	6.3980	7.6465
C	3.8549	12.6825	10.4384
H	3.6513	12.1416	11.3657
C	4.1800	14.1165	10.8478
C	2.5412	12.6372	9.6515
O	4.6350	14.4317	11.9269
O	3.9471	14.9807	9.8461
O	2.7314	12.5421	8.3305
O	1.4511	12.7106	10.1822
C	1.5351	12.5102	7.5285
H	0.9520	13.4244	7.6727
H	1.8777	12.4361	6.4957
H	0.9230	11.6419	7.7895
C	4.3158	16.3540	10.0994
H	4.0498	16.8981	9.1927
H	3.7643	16.7460	10.9580
H	5.3907	16.4282	10.2875

Ru-TS-I4

Energy (POTENTIAL) = -3037.7760432 Eh

Atom	X	Y	Z
N	-0.8999	-0.8568	2.2827
C	-1.8705	-0.8390	3.2434
C	-1.9350	0.1392	4.2616
C	-0.9190	1.1189	4.2869
C	0.0957	1.0522	3.3492
C	0.0769	0.0513	2.3630
C	-2.8512	-1.8714	3.1788
H	0.9062	1.7721	3.3431
H	0.8680	-0.0211	1.6296
C	-3.8962	-1.9292	4.1286
C	-4.8463	-2.9634	3.9847
C	-4.7279	-3.8463	2.9250
C	-3.6667	-3.7079	2.0149
N	-2.7389	-2.7553	2.1448
H	-5.4505	-4.6410	2.7737
H	-3.5681	-4.3562	1.1524
Ru	-1.0407	-2.4712	0.9288
C	0.9044	-3.5481	1.6510
C	-0.0648	-4.5459	1.4035
C	-0.4390	-4.4889	0.0192
C	0.3361	-3.4477	-0.5866
C	1.1472	-2.8439	0.4169
H	-0.4896	-5.2142	2.1434
H	-1.1361	-5.1466	-0.4813
H	0.2978	-3.1626	-1.6288
H	1.8478	-2.0310	0.2847
H	1.3629	-3.3263	2.6070
O	-4.6605	-1.3880	-0.5202
C	-3.5796	-1.0552	-0.0562
C	-2.2419	-1.5723	-0.5015
C	-2.3840	-2.5141	-1.6770
O	-1.8339	-2.4226	-2.7651
H	-5.6647	-3.0459	4.6940
H	-0.9360	1.8973	5.0436
C	-3.0147	0.0736	5.2088
C	-3.9501	-0.9192	5.1499
H	-4.7580	-0.9624	5.8749

H	-3.0637	0.8321	5.9850
O	-3.4894	-0.1764	0.9643
C	-4.7412	0.2364	1.5424
H	-4.4903	1.0205	2.2555
H	-5.4194	0.6199	0.7798
H	-5.2173	-0.6060	2.0511
O	-3.1729	-3.5695	-1.3753
C	-3.3236	-4.5545	-2.4134
H	-3.9589	-5.3313	-1.9853
H	-3.8030	-4.1187	-3.2951
H	-2.3540	-4.9717	-2.7005
O	-1.7697	5.8760	1.4565
O	-2.5116	3.8934	3.1290
N	1.3316	2.4077	0.3498
N	-3.2278	1.8519	-1.0527
C	-1.3864	3.1385	0.6415
C	-0.3551	4.0022	1.1415
C	-0.5516	5.3276	1.6618
C	0.4981	6.0041	2.2667
H	0.3481	6.9933	2.6810
C	1.7893	5.4350	2.2975
H	2.5930	5.9873	2.7766
C	2.0504	4.2346	1.6756
H	3.0528	3.8242	1.6108
C	0.9958	3.5252	1.0472
C	0.4006	1.7454	-0.3110
C	-0.9993	2.0653	-0.1865
C	-1.9608	1.3426	-0.9545
C	-4.1376	1.4173	-2.1411
H	-5.1376	1.2937	-1.7279
H	-3.8424	0.4273	-2.4732
C	-4.1210	2.4055	-3.3097
H	-4.3911	3.4042	-2.9463
H	-3.0975	2.4724	-3.6985
C	-5.0828	1.9669	-4.4163
H	-5.0634	2.6766	-5.2506
H	-6.1144	1.9107	-4.0468
C	-3.6969	2.7740	-0.1081
C	-5.0506	3.1779	-0.0773
H	-5.7339	2.9342	-0.8779
C	-5.5287	3.8532	1.0328
H	-6.5755	4.1410	1.0636
C	-4.7193	4.1049	2.1502
H	-5.1523	4.5383	3.0431
C	-3.3669	3.7872	2.0887
C	-2.9662	4.4856	4.3449
H	-3.3737	5.4888	4.1768
H	-2.0845	4.5567	4.9841
H	-3.7228	3.8563	4.8301
C	-2.8069	3.2558	0.8890
C	-1.5573	0.1251	-1.6097
H	-2.1254	-0.1960	-2.4695
C	-0.1357	-0.1054	-1.7662
H	0.1442	-0.9320	-2.4086
C	0.8133	0.6484	-1.1620
C	-2.0703	7.1471	2.0312
H	-3.1208	7.3317	1.7995
H	-1.9300	7.1364	3.1184
H	-1.4537	7.9388	1.5887
H	-4.8121	0.9787	-4.8081
C	2.2866	0.4524	-1.4538
H	2.4353	-0.5628	-1.8378
C	3.1615	0.5336	-0.1967
C	2.7648	1.4301	-2.5313
O	2.9883	-0.1717	0.7809
O	2.2790	2.5173	-2.7592
O	4.1764	1.3900	-0.3250
O	3.7990	0.9071	-3.2089
C	4.3882	1.7654	-4.2076
H	4.7718	2.6793	-3.7450
H	5.2038	1.1886	-4.6448
H	3.6516	2.0254	-4.9731
C	5.0286	1.5344	0.8294
H	5.5589	0.5996	1.0317
H	5.7343	2.3247	0.5719
H	4.4365	1.8180	1.7036

Rh-I5

Energy (POTENTIAL) = -3312.0716087 Eh

Atom	X	Y	Z
O	0.4665	-1.6291	3.0935
C	-0.2194	-0.7187	2.6870
C	-1.0859	-0.6772	1.4374
C	-2.4926	-0.3861	1.7887
O	-3.3136	0.2343	1.1283
O	-0.2699	0.4846	3.3276
C	0.5668	0.6236	4.4870
H	0.4289	1.6515	4.8273
H	0.2626	-0.0791	5.2688
H	1.6166	0.4495	4.2331
O	-2.8241	-0.9657	2.9734
C	-4.2208	-0.9756	3.2872
H	-4.3060	-1.5096	4.2357
H	-4.6126	0.0417	3.3936

H	-4.7807	-1.5009	2.5076	H	-0.2679	8.4387	2.7926
O	-0.1032	6.6880	1.6564	H	0.6042	8.6103	1.2364
O	-1.4369	5.4471	3.6186	H	-2.6472	0.8347	-3.6864
N	2.4641	2.6939	2.1220	Rh	-1.3052	-2.7334	0.4747
N	-1.8477	2.2976	0.1046	Rh	-1.6050	-4.9331	-0.6191
C	-0.1126	3.8061	1.7163	H	-5.7098	-2.0034	-0.5476
C	0.9668	4.6415	2.1737	C	-5.5209	-3.0795	-0.5294
C	0.9366	6.0725	2.2619	C	-4.0383	-3.3547	-0.4134
C	1.9799	6.7551	2.8760	H	-5.9332	-3.5557	-1.4224
H	1.9485	7.8332	2.9729	H	-6.0245	-3.5014	0.3494
C	3.1145	6.0570	3.3330	O	-3.3549	-2.4832	0.2139
H	3.9155	6.6139	3.8110	O	-3.6041	-4.4363	-0.9189
C	3.2483	4.7011	3.1155	O	0.7214	-3.2163	0.6244
H	4.1548	4.1624	3.3710	C	1.0932	-4.4026	0.3486
C	2.2036	3.9892	2.4846	O	0.3912	-5.3041	-0.2120
C	1.5593	2.0135	1.4612	C	2.4893	-4.8032	0.7662
C	0.2144	2.5080	1.2751	H	2.4057	-5.3952	1.6867
C	-0.7194	1.7181	0.5585	H	3.1095	-3.9292	0.9654
C	-2.5815	1.7427	-1.0676	H	2.9488	-5.4377	0.0027
H	-3.6478	1.7817	-0.8624	O	-1.1149	-4.0017	-2.4274
H	-2.3494	0.6892	-1.1572	C	-0.9394	-2.7473	-2.4470
C	-2.2010	2.4943	-2.3448	O	-0.9688	-1.9663	-1.4369
H	-2.4533	3.5568	-2.2437	C	-0.7078	-2.0878	-3.7873
H	-1.1139	2.4333	-2.4814	H	-1.6670	-1.7066	-4.1600
C	-2.9191	1.8896	-3.5541	H	-0.3156	-2.8065	-4.5105
H	-2.6519	2.4239	-4.4725	H	-0.0238	-1.2413	-3.6824
H	-4.0085	1.9432	-3.4375	O	-1.6901	-3.6936	2.2698
C	-2.3257	3.4751	0.7028	C	-2.0071	-4.9249	2.2688
C	-3.6202	3.9575	0.4163	O	-2.0839	-5.6916	1.2556
H	-4.2292	3.5139	-0.3569	C	-2.2961	-5.5357	3.6223
C	-4.1416	4.9859	1.1797	H	-1.3485	-5.6825	4.1544
H	-5.1458	5.3420	0.9699	H	-2.9106	-4.8544	4.2182
C	-3.4387	5.5280	2.2652	H	-2.7968	-6.5005	3.5159
H	-3.9176	6.2618	2.9012	C	3.4458	0.4404	0.8776
C	-2.1479	5.0891	2.5302	H	3.8071	0.4879	1.9073
C	-1.9436	6.4741	4.4721	C	4.2300	1.4804	0.0662
H	-2.1568	7.3915	3.9120	C	3.7924	-0.9560	0.3684
H	-1.1529	6.6663	5.1992	O	4.1659	-1.8665	1.0766
H	-2.8494	6.1411	4.9934	O	3.7623	2.2298	-0.7628
C	-1.5137	4.1485	1.6586	O	3.6363	-1.0315	-0.9627
C	-0.4340	0.2578	0.3274	O	5.5321	1.4216	0.3875
H	-0.8407	-0.0661	-0.6254	C	3.8559	-2.3191	-1.5797
C	1.0324	-0.0486	0.2922	H	3.9221	-2.1216	-2.6501
H	1.2811	-1.0075	-0.1429	H	4.7790	-2.7732	-1.2128
C	1.9621	0.7439	0.8445	H	3.0060	-2.9733	-1.3703
C	-0.2249	8.1070	1.7488	C	6.3961	2.3233	-0.3362
H	-1.1643	8.3524	1.2501	H	6.1010	3.3605	-0.1543

H	7.3992	2.1398	0.0502
H	6.3555	2.1139	-1.4089

r-3a

Energy (POTENTIAL) = -1683.3633069 Eh

Atom	X	Y	Z
O	-0.5733	-0.9761	-3.2974
C	-1.4308	-1.4635	-2.5921
C	-1.7724	-1.0162	-1.1684
C	-1.4825	-2.1764	-0.2130
O	-0.5820	-2.9797	-0.3570
O	-2.2368	-2.4685	-2.9653
C	-2.0035	-3.0060	-4.2847
H	-2.7246	-3.8154	-4.4028
H	-2.1667	-2.2357	-5.0438
H	-0.9828	-3.3896	-4.3652
O	-2.3285	-2.1629	0.8240
C	-2.1019	-3.1722	1.8328
H	-2.8653	-3.0011	2.5923
H	-2.2083	-4.1719	1.4023
H	-1.1030	-3.0626	2.2638
O	-2.4568	6.4011	0.2188
O	-3.1995	5.0410	2.4385
N	1.1927	3.3746	0.9662
N	-2.9341	1.7300	-0.9698
C	-1.6027	3.6982	0.5020
C	-0.7890	4.8274	0.8036
C	-1.2376	6.1962	0.7768
C	-0.4159	7.2158	1.2165
H	-0.7532	8.2450	1.1982
C	0.9063	6.9281	1.6451
H	1.5307	7.7476	1.9919
C	1.4159	5.6577	1.5745
H	2.4446	5.4351	1.8401
C	0.6045	4.5804	1.1009
C	0.4760	2.3788	0.4162
C	-0.9279	2.5114	0.1368
C	-1.6379	1.4465	-0.5114
C	-3.3170	1.3994	-2.3620
H	-4.2289	0.7912	-2.3740
H	-2.5146	0.7948	-2.7813
C	-3.4746	2.6401	-3.2499
H	-4.2722	3.2833	-2.8624
H	-2.5442	3.2202	-3.2030
C	-3.7805	2.2412	-4.6961
H	-3.8875	3.1271	-5.3321
H	-4.7147	1.6687	-4.7613
C	-3.7059	2.6504	-0.2534
C	-5.1106	2.6190	-0.3458
H	-5.5969	1.9160	-1.0121
C	-5.8635	3.4563	0.4628
H	-6.9481	3.4236	0.4037
C	-5.2566	4.2979	1.4040
H	-5.8714	4.8801	2.0793
C	-3.8655	4.3478	1.4846
C	-3.9249	5.9241	3.2887
H	-4.5024	6.6554	2.7110
H	-3.1709	6.4448	3.8822
H	-4.5979	5.3732	3.9580
C	-3.0605	3.6066	0.5713
C	-1.0122	0.2163	-0.7084
C	0.3715	0.0976	-0.4000
H	0.8542	-0.8571	-0.5855
C	1.1031	1.1338	0.1244
C	-3.0302	7.7026	0.2783
H	-4.0437	7.5979	-0.1148
H	-3.0755	8.0753	1.3088
H	-2.4718	8.4152	-0.3426
H	-2.9782	1.6214	-5.1161
H	2.1633	1.0306	0.3332
H	-2.8484	-0.8343	-1.1239

r-Rh-I5

Energy (POTENTIAL) = -3312.0665731 Eh

Atom	X	Y	Z
O	-3.3012	-0.4736	2.5384
C	-2.4033	-0.1665	1.7733
C	-2.2527	-0.4422	0.3314
C	-3.4388	-0.7059	-0.5163
O	-3.4333	-0.5916	-1.7384
O	-1.2945	0.5098	2.2200
C	-1.1454	0.6280	3.6330
H	-0.1535	1.0582	3.7838
H	-1.9128	1.2827	4.0619
H	-1.2074	-0.3535	4.1144
O	-4.5301	-1.1441	0.1477
C	-5.6279	-1.5342	-0.6834
H	-6.3926	-1.9122	-0.0009
H	-6.0211	-0.6807	-1.2469
H	-5.3244	-2.3184	-1.3815
O	-2.7187	6.8955	-0.0237
O	-1.9856	6.4427	-2.5669
N	-3.1621	2.1375	-0.4494
N	1.1620	4.0464	0.0771

C	-1.4807	4.3919	-0.6618
C	-2.8865	4.5781	-0.4841
C	-3.5325	5.8404	-0.2472
C	-4.9160	5.9103	-0.1746
H	-5.4101	6.8604	-0.0130
C	-5.6941	4.7368	-0.2596
H	-6.7755	4.8215	-0.1990
C	-5.1034	3.4957	-0.3622
H	-5.6820	2.5779	-0.3631
C	-3.6952	3.3952	-0.4370
C	-1.8655	1.9745	-0.4177
C	-0.9601	3.0921	-0.4548
C	0.4225	2.9169	-0.1188
C	2.1628	4.1465	1.1767
H	3.1563	4.3547	0.7700
H	2.1828	3.1872	1.6778
C	1.7515	5.1797	2.2349
H	1.7524	6.1907	1.8170
H	0.7249	4.9591	2.5521
C	2.7045	5.1056	3.4316
H	2.4129	5.8295	4.2002
H	3.7361	5.3306	3.1331
C	0.8147	5.2283	-0.6032
C	1.7891	6.2139	-0.8393
H	2.7981	6.0954	-0.4663
C	1.4529	7.3161	-1.6083
H	2.2045	8.0739	-1.8086
C	0.1830	7.4458	-2.1870
H	-0.0238	8.2846	-2.8399
C	-0.7858	6.4744	-1.9521
C	-2.4052	7.5739	-3.3311
H	-2.3726	8.4925	-2.7340
H	-3.4361	7.3629	-3.6205
H	-1.7900	7.6960	-4.2308
C	-0.5119	5.3928	-1.0624
C	0.9595	1.5808	0.0489
C	0.1160	0.5150	-0.0354
H	0.5356	-0.4736	0.0663
C	-1.3205	0.5615	-0.3862
C	-3.2887	8.1931	0.1409
H	-2.4410	8.8759	0.2224
H	-3.9024	8.4717	-0.7236
H	-3.8923	8.2495	1.0552
H	2.6946	4.1078	3.8868
H	-1.3346	0.2191	-1.4388
Rh	-1.3742	-2.5943	0.3439
Rh	-0.3270	-4.8268	0.2291

O	-1.9489	-5.4630	-0.8977
C	-2.8763	-4.6315	-1.1542
O	-2.9188	-3.4112	-0.8001
C	-4.0555	-5.1279	-1.9618
H	-3.9366	-6.1815	-2.2228
H	-4.1482	-4.5296	-2.8747
H	-4.9755	-4.9937	-1.3826
O	-1.3499	-5.2982	1.9776
C	-2.1143	-4.4238	2.4982
O	-2.3277	-3.2463	2.0673
C	-2.8786	-4.8262	3.7402
H	-2.9881	-3.9697	4.4106
H	-2.3798	-5.6514	4.2540
H	-3.8828	-5.1542	3.4440
O	0.2411	-2.0269	1.5253
C	1.1881	-2.8519	1.7423
O	1.2403	-4.0605	1.3573
C	2.3780	-2.3221	2.5045
H	2.8370	-3.1139	3.1018
H	3.1118	-1.9694	1.7708
H	2.0855	-1.4814	3.1352
O	-0.3478	-2.1238	-1.3966
C	0.4119	-2.9941	-1.9326
O	0.6252	-4.1717	-1.5084
C	1.1008	-2.5982	-3.2179
H	0.6772	-3.1829	-4.0429
H	0.9612	-1.5352	-3.4215
H	2.1667	-2.8327	-3.1524
C	2.4603	1.3104	0.1668
H	3.0269	2.2007	-0.1131
C	2.9075	0.2208	-0.8177
C	2.9244	0.9490	1.5849
O	3.5350	0.7697	-1.8637
O	4.2038	0.5565	1.5611
O	2.2661	1.0767	2.5962
O	2.7202	-0.9705	-0.6726
C	4.0259	-0.1461	-2.8700
H	3.1895	-0.5785	-3.4233
H	4.6477	0.4576	-3.5317
H	4.6145	-0.9419	-2.4070
C	4.8343	0.3362	2.8444
H	4.8122	1.2570	3.4340
H	4.3310	-0.4639	3.3905
H	5.8616	0.0532	2.6147

I0-Rh

Energy (POTENTIAL) = -1628.6419941 Eh

Atom	X	Y	Z
O	-1.2056	0.1310	2.3950
C	-1.0481	0.0185	1.1911
C	-1.9579	-0.8851	0.4696
C	-3.2320	-0.3454	-0.0321
O	-3.2357	0.1522	-1.1450
O	-0.1086	0.5793	0.4485
C	0.8662	1.3870	1.1620
H	1.5378	1.7668	0.3930
H	0.3689	2.2098	1.6811
H	1.4092	0.7625	1.8755
O	-4.2655	-0.5115	0.7761
C	-5.5549	-0.0930	0.2522
H	-6.2639	-0.2890	1.0554
H	-5.5368	0.9709	0.0042
H	-5.8012	-0.6838	-0.6337
Rh	-1.4892	-2.7739	0.1736
Rh	-0.9149	-5.1303	-0.2064
O	-2.7518	-5.3072	-1.1685
C	-3.5184	-4.3016	-1.2541
O	-3.2816	-3.1302	-0.7941
C	-4.8296	-4.4650	-1.9816
H	-5.0363	-5.5190	-2.1758
H	-4.7741	-3.9245	-2.9338
H	-5.6390	-4.0220	-1.3936
O	-1.8237	-5.5863	1.5937
C	-2.3242	-4.6448	2.2780
O	-2.3300	-3.4065	1.9578
C	-2.9905	-4.9853	3.5871
H	-2.5905	-4.3460	4.3802
H	-2.8355	-6.0364	3.8374
H	-4.0642	-4.7814	3.5056
O	0.3351	-2.5542	1.1194
C	1.1110	-3.5682	1.2168
O	0.8702	-4.7384	0.7925
C	2.4449	-3.3134	1.8730
H	2.8743	-4.2456	2.2458
H	3.1245	-2.8860	1.1253
H	2.3366	-2.5902	2.6854
O	-0.5758	-2.3924	-1.6477
C	-0.0556	-3.3447	-2.3251
O	-0.0390	-4.5675	-1.9954
C	0.5932	-2.9446	-3.6260
H	-0.1372	-2.4221	-4.2524
H	1.4137	-2.2486	-3.4208
H	0.9758	-3.8213	-4.1517

Rh-TS-I3-O

Energy (POTENTIAL) = -2816.8504111 Eh

Atom	**X**	**Y**	**Z**
O	-2.4642	-1.3387	0.8015
C	-1.8419	-0.3103	0.4859
C	-1.5853	0.2222	-0.8463
C	-1.9130	-0.5526	-2.0625
O	-1.3303	-0.4227	-3.1333
O	-1.3593	0.5024	1.4532
C	-1.5209	0.0446	2.8088
H	-1.0377	0.8041	3.4263
H	-2.5805	-0.0319	3.0716
H	-1.0360	-0.9252	2.9451
O	-2.9991	-1.3417	-1.9275
C	-3.3041	-2.1648	-3.0588
H	-4.1963	-2.7252	-2.7818
H	-3.4963	-1.5559	-3.9482
H	-2.4798	-2.8540	-3.2635
O	-1.3498	7.3671	-0.8734
O	-1.0185	6.8508	-3.4786
N	-2.0973	2.6842	-1.1590
N	2.4473	4.2457	-1.5150
C	-0.3260	4.8153	-1.6473
C	-1.7028	5.0700	-1.3066
C	-2.2512	6.3699	-1.0243
C	-3.6143	6.5378	-0.8404
H	-4.0280	7.5182	-0.6410
C	-4.4755	5.4171	-0.8521
H	-5.5409	5.5761	-0.7088
C	-3.9862	4.1385	-0.9788
H	-4.6300	3.2670	-0.9174
C	-2.5924	3.9426	-1.1517
C	-0.7701	2.4688	-1.1849
C	0.1576	3.5111	-1.4111
C	1.5506	3.1866	-1.3913
C	3.8667	4.0158	-1.1952
H	3.9000	3.2876	-0.3809
H	4.2779	4.9423	-0.7875
C	4.7027	3.5256	-2.3851
H	4.2618	2.6046	-2.7839
H	4.6585	4.2662	-3.1918
C	6.1555	3.2789	-1.9726
H	6.7499	2.9260	-2.8227
H	6.2190	2.5216	-1.1810
C	2.0304	5.4586	-2.0551
C	2.9763	6.4310	-2.4535
H	4.0336	6.2826	-2.2848

C	2.5571	7.5666	-3.1246
H	3.2978	8.2974	-3.4373
C	1.2159	7.7630	-3.4770
H	0.9366	8.6106	-4.0896
C	0.2687	6.8305	-3.0686
C	-1.4987	7.9737	-4.2153
H	-1.3365	8.9105	-3.6700
H	-2.5698	7.8046	-4.3400
H	-1.0222	8.0365	-5.2015
C	0.6362	5.7302	-2.2312
C	1.9293	1.8566	-1.1819
H	2.9737	1.5741	-1.1715
C	0.9615	0.8400	-1.0063
H	1.2982	-0.1842	-0.8708
C	-0.3898	1.1210	-1.0022
C	-1.8154	8.7020	-0.6880
H	-0.9204	9.3272	-0.6903
H	-2.4798	9.0115	-1.5030
H	-2.3362	8.8128	0.2713
H	6.6229	4.1971	-1.5949
Rh	-1.4925	-3.4006	0.4879
Rh	-0.3511	-5.5139	0.1996
O	-1.8913	-6.1072	-1.0501
C	-2.8668	-5.3081	-1.2349
O	-2.9900	-4.1476	-0.7283
C	-3.9627	-5.7629	-2.1680
H	-3.9792	-6.8525	-2.2416
H	-3.7708	-5.3467	-3.1644
H	-4.9318	-5.3906	-1.8260
O	-1.4065	-6.2104	1.8408
C	-2.2084	-5.4289	2.4480
O	-2.4599	-4.2217	2.1318
C	-2.9010	-5.9682	3.6763
H	-3.9307	-5.6030	3.7203
H	-2.3739	-5.6008	4.5656
H	-2.8848	-7.0601	3.6837
O	0.0777	-2.8051	1.7088
C	1.0333	-3.6195	1.9301
O	1.1313	-4.8006	1.4662
C	2.1515	-3.1151	2.8096
H	2.8453	-3.9192	3.0618
H	2.6913	-2.3223	2.2786
H	1.7346	-2.6776	3.7221
O	-0.4374	-2.7024	-1.1338
C	0.3836	-3.4654	-1.7381
O	0.6302	-4.6767	-1.4342
C	1.1420	-2.8573	-2.8913
H	1.3068	-3.6025	-3.6743
H	0.5973	-1.9950	-3.2817
H	2.1230	-2.5249	-2.5291
H	-2.4204	1.4277	-0.9551

Ter-1

Energy (POTENTIAL) = -2357.11648794 Eh

Atom	X	Y	Z
O	1.1177	8.4254	4.1320
O	-1.0598	6.9276	4.7514
O	3.2957	2.6519	10.5614
O	1.7741	2.7500	8.8530
O	6.2722	3.1810	7.5306
O	5.8944	2.7408	9.7165
N	2.5831	5.3684	7.4421
N	1.9957	3.7678	2.9461
C	1.4710	5.6948	4.9288
C	1.5912	6.8309	5.8107
C	1.3210	8.1944	5.4476
C	1.3492	9.1994	6.4062
H	1.1293	10.2251	6.1394
C	1.7113	8.8948	7.7345
H	1.7211	9.6921	8.4722
C	2.1067	7.6240	8.0997
H	2.4491	7.3994	9.1048
C	2.0950	6.6029	7.1247
C	2.8044	4.3734	6.5298
C	2.2466	4.5477	5.2254
C	2.4859	3.5482	4.2277
C	2.4949	2.9306	1.8362
H	3.5396	2.6960	2.0503
H	2.5026	3.5429	0.9323
C	1.6872	1.6443	1.6185
H	1.6838	1.0563	2.5428
H	0.6431	1.8999	1.4068
C	2.2710	0.8170	0.4699
H	1.6949	-0.1020	0.3184
H	3.3099	0.5315	0.6755
C	0.9824	4.6858	2.7296
C	0.2890	4.7313	1.4951
H	0.5696	4.0927	0.6700
C	-0.8086	5.5617	1.3571
H	-1.3433	5.5741	0.4115
C	-1.2960	6.3350	2.4241
H	-2.2155	6.8943	2.3082
C	-0.6067	6.3288	3.6291
C	-2.2128	7.7725	4.6831

H	-2.0785	8.5676	3.9415
H	-2.3118	8.2107	5.6772
H	-3.1124	7.1919	4.4479
C	0.6222	5.6019	3.7677
C	3.2527	2.4290	4.5713
H	3.4607	1.6474	3.8538
C	3.7252	2.2773	5.8792
H	4.2862	1.3798	6.1225
C	3.5123	3.2116	6.8945
C	4.0202	3.0145	8.2811
C	3.1046	2.7961	9.3542
C	0.7255	2.7395	9.7972
H	-0.0207	1.9992	9.4961
C	5.4434	2.9953	8.4342
C	7.3040	2.7088	9.8584
H	7.7637	1.9100	9.2681
C	0.7567	9.7392	3.6947
H	0.5585	9.6455	2.6259
H	-0.1451	10.0932	4.2061
H	1.5782	10.4471	3.8549
H	7.7724	3.6622	9.5952
H	1.0834	2.5228	10.8072
H	2.2585	1.3807	-0.4712
C	0.0504	4.0998	9.8149
C	7.5844	2.4346	11.3207
F	8.9284	2.3769	11.5294
F	7.0668	1.2561	11.7473
F	7.0980	3.3982	12.1422
F	-0.4861	4.4371	8.6133
F	-0.9632	4.1218	10.7209
F	0.9080	5.0994	10.1490
H	2.9441	5.2171	8.3790

9g

Energy (POTENTIAL) = -1904.30419299 Eh

Atom	X	Y	Z
O	1.1225	8.3577	4.2611
O	-1.0069	6.8360	4.8705
O	3.7029	5.2840	9.5819
O	5.4812	1.4646	8.3122
O	5.2882	2.9015	10.0565
N	2.9079	5.2252	7.3463
N	1.9790	3.7113	2.8529
C	1.5523	5.6381	4.8949
C	1.7116	6.7196	5.8566
C	1.3878	8.0841	5.5585
C	1.4269	9.0611	6.5481
H	1.1558	10.0850	6.3257
C	1.8657	8.7236	7.8376
H	1.8842	9.4919	8.6056
C	2.3375	7.4563	8.1378
H	2.7485	7.2191	9.1081
C	2.3181	6.4636	7.1385
C	3.0355	4.3487	6.3013
C	2.3856	4.5151	5.0798
C	2.5850	3.5070	4.0835
C	2.3898	2.8683	1.7098
H	3.4477	2.6362	1.8452
H	2.3275	3.4718	0.8028
C	1.5680	1.5800	1.5712
H	1.6403	1.0030	2.5000
H	0.5103	1.8343	1.4404
C	2.0595	0.7412	0.3888
H	1.4739	-0.1794	0.2940
H	3.1117	0.4578	0.5137
C	0.9530	4.6309	2.7193
C	0.1842	4.6797	1.5281
H	0.4197	4.0462	0.6855
C	-0.9228	5.5020	1.4557
H	-1.5107	5.5141	0.5425
C	-1.3561	6.2665	2.5546
H	-2.2882	6.8133	2.4938
C	-0.6038	6.2592	3.7183
C	-2.1703	7.6706	4.8687
H	-2.0805	8.4735	4.1286
H	-2.2237	8.0980	5.8710
H	-3.0745	7.0828	4.6726
C	0.6446	5.5474	3.7875
C	3.4114	2.4112	4.3953
H	3.5803	1.6267	3.6696
C	4.0082	2.2753	5.6612
H	4.6135	1.4004	5.8705
C	3.8215	3.2477	6.6539
C	4.2172	3.4484	8.0303
C	3.6554	4.6875	8.5022
C	5.0274	2.5236	8.7592
C	6.0962	1.9966	10.7952
H	5.6153	1.0215	10.9175
C	0.7091	9.6759	3.8890
H	0.4673	9.6157	2.8268
H	-0.1779	9.9883	4.4510
H	1.5190	10.3994	4.0395
H	7.0818	1.8591	10.3405
H	1.9708	1.2943	-0.5542

C	6.2864	2.6094	12.1668
F	7.0497	1.7880	12.9365
F	5.1171	2.7989	12.8258
F	6.9143	3.8100	12.1228

r-Ru-I5

Energy (POTENTIAL) = -3037.7829812 Eh

Atom	X	Y	Z
N	-0.4372	-2.6938	2.0624
C	-1.1912	-3.0394	3.1475
C	-0.7644	-2.8433	4.4813
C	0.5048	-2.2570	4.6771
C	1.2569	-1.9009	3.5699
C	0.7533	-2.1299	2.2797
C	-2.4700	-3.6058	2.8729
H	2.2326	-1.4381	3.6718
H	1.3164	-1.8370	1.4060
C	-3.3212	-3.9902	3.9340
C	-4.5721	-4.5446	3.5927
C	-4.9012	-4.6810	2.2545
C	-3.9983	-4.2619	1.2650
N	-2.8042	-3.7377	1.5565
H	-5.8544	-5.1014	1.9511
H	-4.2428	-4.3466	0.2143
Ru	-1.3155	-3.1433	0.1909
C	0.0261	-4.8038	-0.5372
C	-1.3079	-5.0617	-0.9684
C	-1.7245	-4.0070	-1.8447
C	-0.6197	-3.0956	-1.9543
C	0.4570	-3.5742	-1.1582
H	-1.9184	-5.9014	-0.6566
H	-2.6726	-3.9174	-2.3560
H	-0.6171	-2.1897	-2.5452
H	1.4309	-3.1139	-1.0542
H	0.6148	-5.4259	0.1257
O	-3.3405	-0.8682	2.2453
C	-2.4177	-0.5953	1.4968
C	-2.1937	-1.0030	0.0882
C	-3.3823	-1.3254	-0.7528
O	-3.3807	-1.2762	-1.9819
H	-5.2589	-4.8543	4.3751
H	0.8706	-2.0865	5.6857
C	-1.6462	-3.2389	5.5459
C	-2.8682	-3.7882	5.2833
H	-3.5259	-4.0838	6.0960
H	-1.3159	-3.0900	6.5701
O	-1.3624	0.1662	1.9396
C	-1.4721	0.6701	3.2743
H	-0.5246	1.1724	3.4797
H	-2.2972	1.3866	3.3495
H	-1.6323	-0.1380	3.9919
O	-4.4887	-1.6996	-0.0726
C	-5.6235	-2.0382	-0.8796
H	-6.4089	-2.3176	-0.1746
H	-5.9463	-1.1824	-1.4810
H	-5.4028	-2.8768	-1.5480
O	-1.9091	6.4680	-0.6386
O	-2.8666	5.7826	1.7851
N	1.2031	3.1812	0.7661
N	-3.1208	1.6472	-0.5988
C	-1.4803	3.7960	0.2184
C	-0.5208	4.8632	0.3696
C	-0.7990	6.2362	0.0885
C	0.0758	7.2271	0.5167
H	-0.1408	8.2733	0.3412
C	1.2596	6.8597	1.1697
H	1.9293	7.6361	1.5273
C	1.6343	5.5336	1.3080
H	2.5895	5.3037	1.7515
C	0.7808	4.5223	0.8253
C	0.4449	2.2551	0.1137
C	-0.9425	2.5204	-0.0774
C	-1.8347	1.4576	-0.4678
C	-3.6820	2.8286	-0.1999
C	-5.0893	2.9296	-0.2795
H	-5.6372	2.1076	-0.7279
C	-5.7234	4.0292	0.2585
H	-6.8056	4.1120	0.2107
C	-4.9961	5.0257	0.9405
H	-5.5334	5.8267	1.4326
C	-3.6124	4.9538	1.0215
C	-3.5071	6.8401	2.4998
H	-4.0410	7.5159	1.8220
H	-2.7019	7.3841	2.9969
H	-4.2028	6.4454	3.2504
C	-2.9050	3.9049	0.3388
C	-1.3281	0.0481	-0.6771
H	-1.5305	-0.1948	-1.7353
C	0.1418	-0.0462	-0.5854
H	0.5627	-0.9961	-0.8790
C	0.9936	0.9791	-0.3392
C	-2.3541	7.8151	-0.8125
H	-3.3188	7.7405	-1.3168
H	-2.4802	8.3187	0.1524

H	-1.6558	8.3838	-1.4381
C	2.4439	0.8619	-0.8084
H	2.8562	1.8622	-0.9323
C	2.4553	0.2967	-2.2389
C	3.3994	0.0623	0.0773
O	2.3817	0.9843	-3.2318
O	3.1032	-0.5293	1.0959
O	2.5198	-1.0445	-2.2422
O	4.6337	0.1192	-0.4304
C	5.6521	-0.6366	0.2665
H	5.3805	-1.6950	0.2950
H	6.5643	-0.4907	-0.3117
H	5.7780	-0.2559	1.2827
C	2.4588	-1.6784	-3.5425
H	3.3090	-1.3643	-4.1529
H	2.5006	-2.7490	-3.3464
H	1.5245	-1.4166	-4.0452
C	2.3271	2.7421	1.6602
H	2.1037	3.1992	2.6289
H	2.2043	1.6673	1.7951
C	3.7828	3.0629	1.2787
H	4.0516	2.6424	0.3091
H	3.9439	4.1384	1.1876
C	4.7141	2.4948	2.3571
H	4.5365	2.9748	3.3271
H	4.5647	1.4163	2.4851
H	5.7612	2.6636	2.0851

Ter-TS2

Energy (POTENTIAL) = -2357.07472788 Eh

Atom	X	Y	Z
O	1.0698	8.3830	4.1981
O	-1.1913	6.8730	4.4453
O	3.1592	3.0226	11.0678
O	1.2597	3.7415	9.5039
O	5.9367	2.9698	7.7388
O	5.6800	2.6958	9.9641
N	2.0157	5.3418	7.7489
N	2.1154	3.6949	3.1929
C	1.2945	5.6482	5.0447
C	1.2632	6.7776	5.9249
C	1.0141	8.1377	5.5300
C	0.8092	9.1210	6.4836
H	0.6148	10.1455	6.1925
C	0.8989	8.7987	7.8609
H	0.7221	9.5826	8.5923
C	1.2669	7.5413	8.2791
H	1.4154	7.3083	9.3289
C	1.5255	6.5261	7.3144
C	2.4033	4.3963	6.8592
C	2.0221	4.5129	5.4742
C	2.3905	3.4915	4.5406
C	2.7927	2.8541	2.1875
H	3.7899	2.6274	2.5721
H	2.9449	3.4606	1.2916
C	2.0460	1.5577	1.8424
H	1.9063	0.9624	2.7514
H	1.0439	1.7996	1.4721
C	2.8120	0.7454	0.7943
H	2.2784	-0.1787	0.5476
H	3.8097	0.4690	1.1569
C	1.1439	4.6089	2.8044
C	0.6454	4.6196	1.4802
H	1.0496	3.9625	0.7237
C	-0.4218	5.4406	1.1565
H	-0.8059	5.4317	0.1403
C	-1.0643	6.2286	2.1224
H	-1.9541	6.7848	1.8578
C	-0.5646	6.2486	3.4212
C	-2.3211	7.7071	4.1749
H	-2.0742	8.4857	3.4448
H	-2.5766	8.1692	5.1297
H	-3.1723	7.1167	3.8156
C	0.6305	5.5375	3.7581
C	3.0743	2.3685	5.0110
H	3.3558	1.5617	4.3488
C	3.4142	2.2694	6.3660
H	3.9627	1.3888	6.6853
C	3.1220	3.2499	7.3104
C	3.7327	3.1207	8.6670
C	3.1298	3.1399	9.8948
C	0.5784	4.1564	10.6637
H	0.8891	3.5503	11.5209
C	5.1890	2.9268	8.7077
C	7.0930	2.5444	10.0510
H	7.4453	1.6857	9.4727
C	0.7321	9.6852	3.7156
H	0.7506	9.6084	2.6272
H	-0.2695	9.9841	4.0450
H	1.4666	10.4309	4.0431
H	7.6188	3.4468	9.7273
H	0.7496	5.2143	10.8953
H	2.9397	1.3154	-0.1344
C	-0.9145	3.9579	10.4695

C	7.4067	2.3005	11.5127
F	8.7453	2.1389	11.6720
F	6.8068	1.1856	11.9964
F	7.0288	3.3300	12.3085
F	-1.2447	2.6620	10.2476
F	-1.5984	4.3633	11.5722
F	-1.3941	4.6711	9.4178
H	1.5113	4.5652	8.8820

2

Energy = -1187.9084659 h

Symbol	X	Y	Z
O	2.374781	-1.170253	1.304038
O	1.842356	-1.658823	-1.323265
N	1.198898	3.121276	-0.466474
N	-2.319339	-0.124708	0.397133
C	0.407326	0.421016	-0.033588
C	1.767500	0.812768	0.158438
C	2.800109	-0.024190	0.714073
C	4.121076	0.385846	0.709193
H	4.899550	-0.244886	1.119258
C	4.460777	1.671794	0.212216
H	5.506106	1.968809	0.208089
C	3.493593	2.555465	-0.197716
H	3.739393	3.565971	-0.509269
C	2.114703	2.174624	-0.174171
C	-0.107958	2.812850	-0.333474
C	-0.549145	1.457517	-0.108954
C	-1.947846	1.178503	0.064502
C	-3.683728	-0.373601	0.895716
H	-3.976765	0.494099	1.491681
H	-3.642079	-1.216660	1.589158
C	-4.719362	-0.635667	-0.206755
H	-4.737694	0.212396	-0.899956
H	-4.416120	-1.511092	-0.791400
C	-6.111997	-0.858291	0.388292
H	-6.849825	-1.045683	-0.399221
H	-6.443230	0.019118	0.957198
C	-1.464188	-1.191102	0.132630
C	-1.935354	-2.524491	0.163962
H	-2.950509	-2.753075	0.454977
C	-1.102539	-3.557337	-0.237004
H	-1.477594	-4.576672	-0.217444
C	0.184897	-3.312568	-0.732059
H	0.776928	-4.129716	-1.122968
C	0.667346	-2.005427	-0.743678
C	2.729846	-2.687476	-1.773796
H	2.974527	-3.382384	-0.963622
H	3.634152	-2.170425	-2.098005
H	2.300674	-3.238889	-2.618666
C	-0.099826	-0.935913	-0.189997
C	-2.866610	2.224875	-0.023174
H	-3.926979	2.053697	0.099770
C	-2.419440	3.537764	-0.285092
H	-3.161855	4.328215	-0.358274
C	-1.083049	3.841372	-0.442920
C	3.344596	-2.101501	1.792204
H	2.774559	-2.969666	2.126655
H	4.040190	-2.401391	1.000878
H	3.904631	-1.683447	2.637394
H	-6.118506	-1.720036	1.066982
H	-0.747920	4.857373	-0.627097

3a

Energy = -1683.022553 h

Symbol	X	Y	Z
O	3.354820	-2.022400	-1.154661
O	3.649047	-1.276130	1.449119
O	-4.882706	0.930947	2.224917
O	-5.471549	1.018891	0.046341
O	-3.676622	-1.006669	-1.741337
O	-4.837575	-2.145096	-0.172938
N	-1.128945	-1.631059	0.478981
N	1.380017	2.405085	-0.530323
C	1.368698	-0.347717	0.047171
C	1.257740	-1.766295	-0.082474
C	2.294989	-2.637552	-0.571941
C	2.146795	-4.011993	-0.515790
H	2.927406	-4.669070	-0.877779
C	0.937519	-4.574620	-0.030324
H	0.847470	-5.656703	0.013285
C	-0.127194	-3.781546	0.320470
H	-1.079803	-4.204629	0.623813
C	-0.016795	-2.358667	0.243318
C	-1.079847	-0.299543	0.289685
C	0.163624	0.388873	0.057152
C	0.169087	1.802001	-0.195766
C	1.368427	3.754816	-1.122423
H	0.477962	3.826646	-1.751808
H	2.223074	3.833656	-1.798080
C	1.388631	4.895458	-0.095524
H	0.532463	4.794708	0.580406
H	2.288103	4.812896	0.524335
C	1.352080	6.259414	-0.789390
H	1.369267	7.073133	-0.056458
H	0.443595	6.370623	-1.393948
C	2.585875	1.793688	-0.197872
C	3.800978	2.516566	-0.232864
H	3.833791	3.540158	-0.577583
C	4.964820	1.925913	0.233843
H	5.890685	2.493700	0.212345

C	4.963552	0.642569	0.796536
H	5.869084	0.244686	1.235848
C	3.777088	-0.087509	0.811251
C	4.816610	-1.920491	1.968898
H	5.568232	-2.069863	1.186662
H	4.474912	-2.888622	2.337941
H	5.250658	-1.345916	2.795449
C	2.593921	0.424806	0.197442
C	-1.042250	2.491996	-0.183088
H	-1.088895	3.555514	-0.370946
C	-2.245294	1.809248	0.087960
H	-3.159507	2.396277	0.091112
C	-2.295460	0.451965	0.334233
C	-3.575259	-0.311401	0.608523
H	-3.402940	-0.998447	1.441192
C	-4.708711	0.602397	1.065980
C	-6.568047	1.909612	0.374954
H	-7.256999	1.418608	1.066437
H	-7.061422	2.119846	-0.573415
C	-4.009497	-1.170941	-0.584539
C	-5.363228	-3.021976	-1.198964
H	-6.008547	-3.726573	-0.674675
H	-5.937773	-2.447407	-1.930033
C	4.475283	-2.809965	-1.567779
H	5.226954	-2.095000	-1.905892
H	4.875392	-3.398117	-0.734751
H	4.206394	-3.477081	-2.395373
H	-4.546999	-3.549791	-1.698529
H	-6.183220	2.829981	0.820587
H	2.215027	6.387586	-1.454334

r-3a

Energy (POTENTIAL) = -1683.3633069 Eh

Atom	X	Y	Z
O	-0.5733	-0.9761	-3.2974
C	-1.4308	-1.4635	-2.5921
C	-1.7724	-1.0162	-1.1684
C	-1.4825	-2.1764	-0.2130
O	-0.5820	-2.9797	-0.3570
O	-2.2368	-2.4685	-2.9653
C	-2.0035	-3.0060	-4.2847
H	-2.7246	-3.8154	-4.4028
H	-2.1667	-2.2357	-5.0438
H	-0.9828	-3.3896	-4.3652
O	-2.3285	-2.1629	0.8240
C	-2.1019	-3.1722	1.8328
H	-2.8653	-3.0011	2.5923
H	-2.2083	-4.1719	1.4023
H	-1.1030	-3.0626	2.2638
O	-2.4568	6.4011	0.2188
O	-3.1995	5.0410	2.4385
N	1.1927	3.3746	0.9662
N	-2.9341	1.7300	-0.9698
C	-1.6027	3.6982	0.5020
C	-0.7890	4.8274	0.8036
C	-1.2376	6.1962	0.7768
C	-0.4159	7.2158	1.2165
H	-0.7532	8.2450	1.1982
C	0.9063	6.9281	1.6451
H	1.5307	7.7476	1.9919
C	1.4159	5.6577	1.5745
H	2.4446	5.4351	1.8401
C	0.6045	4.5804	1.1009
C	0.4760	2.3788	0.4162
C	-0.9279	2.5114	0.1368
C	-1.6379	1.4465	-0.5114
C	-3.3170	1.3994	-2.3620
H	-4.2289	0.7912	-2.3740
H	-2.5146	0.7948	-2.7813
C	-3.4746	2.6401	-3.2499
H	-4.2722	3.2833	-2.8624
H	-2.5442	3.2202	-3.2030
C	-3.7805	2.2412	-4.6961
H	-3.8875	3.1271	-5.3321
H	-4.7147	1.6687	-4.7613
C	-3.7059	2.6504	-0.2534
C	-5.1106	2.6190	-0.3458
H	-5.5969	1.9160	-1.0121
C	-5.8635	3.4563	0.4628
H	-6.9481	3.4236	0.4037
C	-5.2566	4.2979	1.4040
H	-5.8714	4.8801	2.0793
C	-3.8655	4.3478	1.4846
C	-3.9249	5.9241	3.2887
H	-4.5024	6.6554	2.7110
H	-3.1709	6.4448	3.8822
H	-4.5979	5.3732	3.9580
C	-3.0605	3.6066	0.5713
C	-1.0122	0.2163	-0.7084
C	0.3715	0.0976	-0.4000
H	0.8542	-0.8571	-0.5855
C	1.1031	1.1338	0.1244
C	-3.0302	7.7026	0.2783
H	-4.0437	7.5979	-0.1148
H	-3.0755	8.0753	1.3088
H	-2.4718	8.4152	-0.3426
H	-2.9782	1.6214	-5.1161

H	2.1633	1.0306	0.3332
H	-2.8484	-0.8343	-1.1239

4aa

Energy = -2178.1293437 h

Symbol	X	Y	Z
O	4.425549	-1.964989	1.203285
O	2.569236	-2.481708	2.384042
O	3.597693	-3.864951	-1.197952
O	3.413306	-1.792455	-2.079269
O	-4.329507	-0.238979	1.850664
O	-4.723068	0.373501	-0.784008
N	-0.607571	2.570540	0.322007
N	-0.651401	-2.207608	-0.444946
C	-2.051213	0.125986	0.182142
C	-2.729473	1.368644	0.014892
C	-4.113602	1.508036	-0.359918
C	-4.721877	2.750332	-0.360207
H	-5.763619	2.857715	-0.634962
C	-3.964719	3.910399	-0.050904
H	-4.466501	4.874202	-0.051706
C	-2.614452	3.838834	0.188914
H	-2.016767	4.728357	0.361565
C	-1.950372	2.574261	0.173910
C	0.048759	1.405063	0.198683
C	1.480635	1.399593	0.204289
C	2.142416	0.198682	0.100562
H	3.227859	0.182981	0.127780
C	1.481744	-1.050038	-0.034457
C	2.311204	-2.321992	0.037109
H	1.661251	-3.184036	0.200918
C	3.241272	-2.243781	1.253676
C	3.162647	-2.598338	-1.204880
C	4.455297	-4.261881	-2.297920
H	3.924276	-4.155109	-3.246937
H	5.362292	-3.652530	-2.306077
H	4.698155	-5.307491	-2.110969
C	0.089371	-1.066814	-0.117287
C	-0.239714	-3.078013	-1.576274
H	0.729157	-2.719306	-1.918054
H	-0.113403	-4.109782	-1.234297
C	-1.206467	-3.002039	-2.765407
H	-2.182085	-3.415316	-2.488671
H	-0.795845	-3.659206	-3.542418
C	-1.940949	-2.335388	0.076427
C	-2.645062	-1.172212	0.484898
C	-0.641116	0.151021	0.088129
C	-2.531698	-3.608377	0.201984
H	-2.010790	-4.489583	-0.151860
C	-3.762732	-3.726355	0.829913
C	-4.410017	-2.612174	1.384437
H	-5.330081	-2.747098	1.938639
C	-3.839044	-1.347752	1.244639
C	-5.608143	-0.301215	2.491296
H	-5.830340	0.721804	2.798463
H	-6.380457	-0.653078	1.799072
H	-5.577983	-0.949954	3.374515
C	-6.124131	0.401941	-1.071401
H	-6.698415	0.755968	-0.208262
H	-6.336643	1.034222	-1.941662
H	-6.397635	-0.630235	-1.295334
C	-1.373801	-1.579952	-3.307828
H	-1.829232	-0.921081	-2.560951
H	-0.405535	-1.147912	-3.588420
H	-2.017561	-1.574656	-4.194081
C	3.313433	-2.330736	3.620802
H	3.687575	-1.307763	3.708835
H	2.598272	-2.547198	4.413680
H	4.145395	-3.038257	3.649855
H	-4.213253	-4.708544	0.941471
C	2.186886	2.735035	0.327480
H	1.708373	3.310223	1.126457
C	3.659664	2.588696	0.696540
C	2.074732	3.567291	-0.952900
O	4.592532	2.753920	-0.068798
O	3.796000	2.237286	1.980900
O	2.204282	4.873051	-0.676295
O	1.905099	3.120829	-2.070282
C	2.165001	5.779191	-1.805864
H	1.205788	5.696596	-2.322893
H	2.285189	6.775664	-1.381277
H	2.981728	5.556505	-2.497206
C	5.148410	1.994530	2.445148
H	5.742541	2.908631	2.371256
H	5.043697	1.688842	3.485703
H	5.611094	1.199004	1.856382

5

Energy = -2673.2442238 h

Symbol	X	Y	Z
O	3.049981	-3.329388	1.748411
O	4.023916	-4.568753	0.133989
O	3.943505	-1.240570	-2.260932
O	5.304253	-1.846997	-0.561789
O	0.872963	4.262675	-1.509979
O	0.483132	4.411595	1.193453
N	-1.841138	0.436662	-0.128751
N	2.984678	0.361346	0.243142
C	0.615935	1.835599	-0.037804
C	-0.597236	2.500895	0.303264
C	-0.687749	3.843035	0.817338
C	-1.921148	4.444348	0.983841

H	-2.000163	5.456546	1.359537
C	-3.109932	3.718469	0.717982
H	-4.063969	4.219432	0.861987
C	-3.087848	2.394405	0.348920
C	-1.819728	1.743625	0.174040
C	-0.687690	-0.244441	-0.166182
C	-0.703477	-1.665095	-0.331569
C	0.495183	-2.332984	-0.436236
H	0.483039	-3.405215	-0.613341
C	1.760387	-1.699357	-0.323326
C	2.972306	-2.582054	-0.581611
H	2.692026	-3.251293	-1.404340
C	3.345708	-3.505902	0.581138
C	4.212617	-1.845340	-1.099018
C	5.020116	-0.471025	-2.851273
H	5.359018	0.298337	-2.154621
H	5.851214	-1.128507	-3.118780
H	4.591211	-0.015171	-3.743204
C	1.800539	-0.329744	-0.066213
C	3.767844	-0.057597	1.436361
H	3.618094	-1.124064	1.577486
H	4.830443	0.094018	1.237206
C	3.333025	0.682146	2.705819
H	2.264489	0.497863	2.874035
H	3.455220	1.762430	2.563600
C	3.075500	1.706415	-0.148402
C	1.893365	2.450246	-0.391903
C	0.580985	0.422371	-0.086623
C	4.339317	2.306343	-0.302224
H	5.239265	1.748805	-0.072594
C	4.422390	3.595543	-0.808236
C	3.276255	4.294652	-1.215264
H	3.379102	5.265212	-1.683456
C	2.020511	3.713380	-1.041456
C	0.903891	5.589090	-2.047655
H	-0.135177	5.840059	-2.265711
H	1.309447	6.301353	-1.321072
H	1.491472	5.627789	-2.972330
C	0.490920	5.785307	1.594210
H	0.066753	6.426498	0.813765
H	-0.061390	5.927033	2.530786
H	1.540763	6.039236	1.747628
C	4.145981	0.220116	3.918756
H	4.018249	-0.854718	4.095239
H	5.216245	0.410357	3.771489
H	3.833310	0.748965	4.825796
C	4.479301	-5.514070	1.134170
H	3.625890	-5.940388	1.667057
H	5.008678	-6.287842	0.579005
H	5.151803	-5.019680	1.839599
H	5.397350	4.054989	-0.944412
C	-2.039116	-2.380246	-0.407789
H	-2.823205	-1.665315	-0.141197
C	-2.394213	-2.864040	-1.811895
C	-2.074128	-3.524875	0.596600
O	-1.771289	-2.608092	-2.825606
O	-3.534419	-3.565344	-1.791629
O	-2.625139	-3.129958	1.752008
O	-1.617285	-4.637736	0.401000
C	-2.634727	-4.094710	2.832169
H	-1.612122	-4.378695	3.093668
H	-3.115546	-3.586452	3.667411
H	-3.205802	-4.980906	2.544164
C	-4.047009	-4.008262	-3.071243
H	-3.324029	-4.662329	-3.564950
H	-4.961662	-4.554775	-2.842335
H	-4.265815	-3.146112	-3.706437
C	-4.379831	1.611791	0.208263
H	-5.185351	2.323875	-0.000061
C	-4.405549	0.620778	-0.958130
C	-4.821826	0.875209	1.476509
O	-3.813835	0.273323	2.108691
O	-4.004306	1.205432	-2.093920
O	-5.979929	0.852149	1.858807
O	-4.830801	-0.519152	-0.891497
C	-4.149026	-0.504978	3.280728
H	-4.605698	0.132154	4.042667
H	-3.202515	-0.908680	3.636904
H	-4.832763	-1.313627	3.012550
C	-3.939982	0.354481	-3.262818
H	-4.916780	-0.088866	-3.470899
H	-3.196680	-0.431590	-3.109957
H	-3.636370	1.008599	-4.080122

6

Energy (POTENTIAL) = -3168.3567226 h

Symbol	X	Y	Z
O	-1.207198	3.711560	-1.913766
O	-2.606382	4.864183	-0.567604
O	-0.369448	4.559596	1.498947
O	-2.041503	3.592908	2.670886
O	-1.146738	-4.118441	-1.478400
O	-0.538076	-4.467455	1.156479
N	2.416474	-1.012554	-0.341998
N	-2.206358	0.034606	0.678509
C	-0.254706	-1.872239	0.022352
C	0.815971	-2.789385	0.184278
C	0.671647	-4.148868	0.638248
C	1.748971	-5.011102	0.602197
H	1.649996	-6.039037	0.927630
C	3.021253	-4.544019	0.180766
H	3.847198	-5.250381	0.157633

C	3.248956	-3.226242	-0.137429
C	2.149205	-2.302443	-0.091773
C	1.438939	-0.106535	-0.205486
C	1.746566	1.290475	-0.275258
C	0.735314	2.205320	-0.106901
H	0.968537	3.262103	-0.151740
C	-0.611567	1.834449	0.162096
C	-1.653852	2.919605	0.361104
H	-2.642299	2.467657	0.466536
C	-1.764246	3.861422	-0.843652
C	-1.403688	3.720893	1.642341
C	0.013039	5.333030	2.663893
H	-0.814043	5.974257	2.978022
H	0.301383	4.664714	3.478774
H	0.863375	5.933576	2.342543
C	-0.934678	0.487257	0.239975
C	-2.459420	0.209946	2.146508
H	-2.513469	1.277256	2.366261
H	-3.439833	-0.219493	2.361165
C	-1.414633	-0.462216	3.044143
H	-0.431279	-0.013400	2.861674
H	-1.337184	-1.523863	2.780980
C	-2.629397	-1.209084	0.134691
C	-1.670178	-2.186315	-0.214575
C	0.076076	-0.496963	0.001716
C	-4.004215	-1.403896	-0.113826
C	-4.400831	-2.597763	-0.711566
C	-3.472024	-3.552202	-1.133014
H	-3.821830	-4.433433	-1.655279
C	-2.107427	-3.327971	-0.944337
C	-1.521794	-5.344593	-2.116352
H	-0.583039	-5.831993	-2.383149
H	-2.088457	-5.987350	-1.433892
H	-2.107586	-5.156294	-3.023642
C	-0.813827	-5.835261	1.475935
H	-0.651834	-6.482432	0.607076
H	-0.195358	-6.177719	2.313912
H	-1.865652	-5.862786	1.764357
C	-1.786612	-0.312124	4.522306
H	-1.859460	0.744910	4.805847
H	-2.752979	-0.784222	4.737797
H	-1.033419	-0.780231	5.165530
C	-2.843776	5.824023	-1.627878
H	-3.280282	5.325206	-2.496343
H	-3.544003	6.546554	-1.209959
H	-1.908018	6.314141	-1.907797
H	-5.449093	-2.781625	-0.926360
C	3.191582	1.708644	-0.467818

H	3.765048	0.849799	-0.823591
C	3.292663	2.767669	-1.560758
C	3.852756	2.167396	0.834377
O	3.412130	2.508660	-2.743815
O	3.176939	4.010742	-1.070417
O	5.166183	2.356064	0.641659
O	3.291261	2.330857	1.900203
C	5.924645	2.843146	1.775462
H	5.820903	2.165265	2.624754
H	6.959976	2.876612	1.437165
H	5.580855	3.843217	2.052936
C	3.168251	5.094171	-2.034258
H	3.085439	6.004837	-1.441806
H	4.095985	5.093887	-2.611166
H	2.309920	4.991792	-2.702969
C	4.651268	-2.746846	-0.464349
H	5.223769	-3.606449	-0.829453
C	5.439996	-2.230208	0.742563
C	4.737840	-1.703211	-1.579522
O	6.588761	-2.565013	0.978077
O	5.465897	-0.726030	-1.550172
O	3.996864	-2.052994	-2.636603
O	4.737544	-1.387490	1.501797
C	3.962741	-1.103812	-3.730055
H	4.954881	-1.002701	-4.177914
H	3.260674	-1.521076	-4.451916
H	3.615973	-0.130843	-3.372020
C	5.425984	-0.804754	2.632470
H	4.701814	-0.135629	3.095494
H	5.729903	-1.584194	3.336111
H	6.303057	-0.248390	2.294053
C	-4.970160	-0.245227	0.098040
H	-4.879327	0.164619	1.105276
C	-6.434276	-0.654350	-0.043280
C	-4.697181	0.935309	-0.840566
O	-4.892577	2.094692	-0.519830
O	-7.161269	-0.349637	-0.970955
O	-4.253929	0.550901	-2.038032
O	-6.819783	-1.392189	1.004217
C	-4.003715	1.597336	-3.008292
H	-4.907100	2.191017	-3.166902
H	-3.187606	2.238271	-2.669234
H	-3.720933	1.077694	-3.923485
C	-8.186879	-1.877719	0.986883
H	-8.349989	-2.511729	0.111945
H	-8.297456	-2.455097	1.904151
H	-8.884881	-1.037157	0.975628

References

[1] F. Zinna, T. Bruhn, C. A. Guido, J. Ahrens, M. Bröring, L. Di Bari, G. Pescitelli, *Chem. Eur. J.* **2016**, *22*, 16089-16098.

[2] M. J. Frisch, G. W. Trucks, H. B. Schlegel, G. E. Scuseria, M. A. Robb, J. R. Cheeseman, G. Scalmani, V. Barone, B. Mennucci, G. A. Petersson, H. Nakatsuji, M. Caricato, X. Li, H. P. Hratchian, A. F. Izmaylov, J. Bloino, G. Zheng, J. L. Sonnenberg, M. Hada, M. Ehara, K. Toyota, R. Fukuda, J. Hasegawa, M. Ishida, T. Nakajima, Y. Honda, O. Kitao, H. Nakai, T. Vreven, J. A. Montgomery, J. E. Peralta, F. Ogliaro, M. Bearpark, J. J. Heyd, E. Brothers, K. N. Kudin, V. N. Staroverov, R. Kobayashi, J. Normand, K. Raghavachari, A. Rendell, J. C. Burant, S. S. Iyengar, J. Tomasi, M. Cossi, N. Rega, J. M. Millam, M. Klene, J. E. Knox, J. B. Cross, V. Bakken, C. Adamo, J. Jaramillo, R. Gomperts, R. E. Stratmann, O. Yazyev, A. J. Austin, R. Cammi, C. Pomelli, J. W. Ochterski, R. L. Martin, K. Morokuma, V. G. Zakrzewski, G. A. Voth, P. Salvador, J. J. Dannenberg, S. Dapprich, A. D. Daniels, Farkas, J. B. Foresman, J. V. Ortiz, J. Cioslowski and D. J. Fox, Gaussian 09, Revision D. 01, 2009, Gaussian. *Inc., Wallingford CT* **2009**.

[3] a) A. D. Becke, *J. Chem. Phys.* **1993**, *98*, 5648-5652; b) C. Lee, W. Yang, R. G. Parr, *Phys. Rev. B* **1988**, *37*, 785-789; c) P. J. Stephens, F. J. Devlin, C. F. Chabalowski, M. J. Frisch, *J. Phys. Chem.* **1994**, *98*, 11623-11627.

[4] S. Grimme, J. Antony, S. Ehrlich, H. Krieg, *J. Chem. Phys.* **2010**, *132*, 154104.

[5] a) M. M. Francl, W. J. Pietro, W. J. Hehre, J. S. Binkley, M. S. Gordon, D. J. DeFrees, J. A. Pople, *J. Chem. Phys.* **1982**, *77*, 3654-3665; b) P. C. Hariharan, J. A. Pople, *Theor. Chim. Acta* **1973**, *28*, 213-222; c) W. J. Hehre, R. Ditchfield, J. A. Pople, *J. Chem. Phys.* **1972**, *56*, 2257-2261.

[6] P. J. Hay, W. R. Wadt, *J. Chem. Phys.* **1985**, *82*, 270-283.

[7] R. Krishnan, J. S. Binkley, R. Seeger, J. A. Pople, *J. Chem. Phys.* **1980**, *72*, 650-654.

[8] L. E. Roy, P. J. Hay, R. L. Martin, *J. Chem. Theory Comput.* **2008**, *4*, 1029-1031.

[9] A. V. Marenich, C. J. Cramer, D. G. Truhlar, *J. Phys. Chem. B* **2009**, *113*, 6378-6396.

[10] W. Hehre, P. Klunzinger, B. Deppmeier, A. Driessen, N. Uchida, M. Hashimoto, E. Fukushi, Y. Takata, *J. Nat. Prod.* **2019**, *82*, 2299-2306.

[11] T. Yanai, D. P. Tew, N. C. Handy, *Chem. Phys. Lett.* **2004**, *393*, 51-57.

[12] F. Weigend, R. Ahlrichs, *Phys. Chem. Chem. Phys.* **2005**, *7*, 3297-3305.

[13] J. Tomasi, B. Mennucci, R. Cammi, *Chem. Rev.* **2005**, *105*, 2999-3094.

[14] G. Sheldrick, *Acta Crystallogr. C* **2015**, *71*, 3-8.

[15] L. J. Bourhis, O. V. Dolomanov, R. J. Gildea, J. A. K. Howard, H. Puschmann, *Acta Crystallogr. A* **2015**, *71*, 59-75.

[16] C. C. Scott, S. Vossio, J. Rougemont, J. Gruenberg, *eLife* **2018**, *7*, e36330.

[17] Q. Laurent, R. Martinent, D. Moreau, N. Winssinger, N. Sakai, S. Matile, *Angew. Chem. Int. Ed.* **2021**, *60*, 19102-19106.

[18] a) H. M. Davies, W. R. Cantrell Jr, K. R. Romines, J. S. Baum, *Org. Synth.* **2003**, *70*, 93-93; b) L. S. Campbell-Verduyn, L. Mirfeizi, R. A. Dierckx, P. H. Elsinga, B. L. Feringa, *Chem. Commun.* **2009**, 2139-2141.

[19] A. Wallabregue, P. Sherin, J. Guin, C. Besnard, E. Vauthey, J. Lacour, *Eur. J. Org. Chem.* **2014**, *2014*, 6431-6438.

[20] For the isolation of **r-3a**, a reaction time of 9h only was selected. The isolated yield is higher as less of this mono-functionalized material is consumed and transformed into **4aa** as reported in the main manuscript for a reaction of 16h.

[21] Even though the aromaticity is lost upon insertion of the carbene, **I1** is almost isoenergetic to **I0** for Ru and very exergonic for Rh, in agreement with the expected higher reactivity of the later carbene.

[22] J. Viñas-Lóbez, G. Levitre, A. de Aguirre, C. Besnard, A. I. Poblador-Bahamonde, J. Lacour, *ACS Org. Inorg. Au* **2021**, *1*, 11-17.

[23] Efforts were made to find this transition state but were unsuccessful probably due to the barrierless nature of it.

[24] During the mechanism search, two alternative pathways were considered. On one hand, a direct transfer from **I1** to intermediate **I3** or product **3a** and, on the other, a concerted rebound mechanism. Associated transition states for these mechanisms could not be located.
[25] B. Laleu, C. Herse, B. W. Laursen, G. Bernardinelli, J. Lacour, *J. Org. Chem.* **2003**, *68*, 6304-6308.
[26] X. Xie, J. Zhai, Z. Jarolímová, E. Bakker, *Anal. Chem.* **2016**, *88*, 3015-3018.
[27] N. Ye, K. Wygladacz, E. Bakker, *Anal. Chim. Acta* **2007**, *596*, 195-200.
